# JMJD1A/NR4A1 Signaling Regulates the Procession of Renal Tubular Epithelial Interstitial Fibrosis Induced by AGEs in HK-2

**DOI:** 10.3389/fmed.2021.807694

**Published:** 2022-02-03

**Authors:** Shaoting Wang, Anna Zuo, Weiqiang Jiang, Jiarun Xie, Haoyu Lin, Wei Sun, Min Zhao, Jinjin Xia, Junqiao Shao, Xiaoshan Zhao, Donghui Liang, Aicheng Yang, Jia Sun, Ming Wang

**Affiliations:** ^1^School of Traditional Chinese Medicine, Southern Medical University, Guangzhou, China; ^2^Zhujiang Hospital, Southern Medical University, Guangzhou, China; ^3^The Affiliated Jiangmen Traditional Chinese Medicine Hospital, Jinan University, Guangzhou, China

**Keywords:** diabetic kidney disease, renal fibrosis, epigenetics, JMJD1A, NR4A1

## Abstract

Diabetic kidney disease (DKD) is one of the most serious complications of diabetic patients. Advanced glycation end products (AGEs) induce epithelial-mesenchymal transformation (EMT) of renal tubular epithelial cells (HK-2), resulting in renal tubulointerstitial fibrosis. However, the underlying epigenetic mechanisms remain to be further investigated. In this work, we investigated the functional role of JMJD1A involved in DKD progression. The molecular mechanism study was performed in AGEs-induced HK-2 cells by gene expression analysis, RNA sequencing (RNA-seq), and JMJD1A lentiviral knockdown and overexpression particle transfection. The results showed that AGEs could upregulate JMJD1A, and the expressions of related fibrotic factor were also increased. At the same time, in the DKD animal model induced by unilateral nephrectomy plus streptozotocin (STZ), IHC immunohistochemical staining showed that compared with the control group, the expressions of JMJD1A, FN, and COL1 in the model group were all increased, masson staining results also show that the model group has typical fibrotic changes. This is consistent with the results of our *in vitro* experiments. In order to determine the downstream pathway, we screened out JMJD1A downstream transcription factors by RNA-seq. Further analysis showed that JMJD1A overexpression could accelerate the progression of AGEs-induced renal fibrosis by reducing the expression of NR4A1 in HK-2 cells. Meanwhile, NR4A1 inhibitor can promote the expression of fibrosis-related factors such as VIM, a-SMA in HK-2 cells, and aggravate the process of fibrosis. Taken together, JMJD1A/NR4A1 signaling can regulate the procession of renal tubular epithelial interstitial fibrosis induced by AGEs in HK-2.

## Introduction

Diabetes is a metabolic disease characterized by hyperglycemia, mainly divided into type 1 diabetes, type 2 diabetes, gestational diabetes, and special type diabetes (1, 2). Diabetic kidney disease (DKD), known as diabetic glomerulosclerosis, is one of the most common microvascular complications of diabetes mellitus (DM) (3, 4). It is also the most common and serious chronic complication of type 2 diabetes mellitus (T2DM), which often leads to end-stage renal failure in patients with diabetes (5, 6).

Advanced glycation end products (AGEs) are the end products of non-enzymatic glycation reaction (Maillard reaction), which refer to the heterogeneous molecules formed by the reaction of proteins, lipids, or nucleic acids with glucose or other reducing monosaccharides without the participation of enzymes (7, 8). AGEs cause oxidative stress, trigger excessive reactive oxygen species, promote the production and release of inflammatory cytokines, and aggravate the development of diabetes, chronic renal failure, cardiovascular diseases, and neurological diseases (9–12). In addition, AGEs are the main cause of diabetic microvascular disease (13). The continuous stimulation of risk factors usually causes damage to HK-2 cells in renal tubular epithelial cells, including high glucose levels, proteinuria, and age-modifying proteins that all lead to extracellular matrix (ECM) deposition and abnormal synthesis and degradation of epithelial proteins, leading to epithelial-mesenchymal transition (EMT) and even interstitial fibrosis (14–17).

In recent years, a large number of studies have found that epigenetic modification plays an important role in the pathogenesis and treatment of many major diseases, in which the difference of histone modification sites may have an important impact on some diseases (18, 19). Histone methylation is the most stable modification method, which is most conducive to transmitting and preserving stable epigenetic information (20). It plays an important role in activating and inhibiting gene expression and has an important impact on growth and development (21, 22). Studies have shown that epigenetic remodeling can regulate fibroblast activation, differentiation and apoptosis, collagen synthesis, and fibrogenic gene transcription. Epigenetic histone modifications play an important role in regulating renal gene expression in diabetic patients (23, 24). Histone demethylase JMJD1A, also known as KDM3A, JHDM2A, TSGA, and KIAA0742, is a demethylation of H3 lysine 9 (H3K9) dimethyl and monomethyl (me2/1) in histone alkaline enzyme (25). JMJD1A may play a role in homodimer formation in transcriptional regulation (26). In addition to being highly expressed in male germ cells, JMJD1A is also expressed in varying degrees in heart, ovary, kidney, lung, brain, liver, skeletal muscle, pancreas, spleen, and skin (27, 28). It was found that JMJD1A can inhibit the activity of TGIF1 and promote the activation of TGF-β1/Smad2/3 signals, thereby promoting the process of fibrosis, and the silencing of JMJD1A can inhibit the kidney damage caused by HG (29, 30).

Nuclear receptor subfamily 4 Group A member 1 (NR4A1), also known as Nur77, nerve growth factor IB (NGFI-B) and TR3, is a member of the NR4A nuclear receptor superfamily (31). JMJD1A inhibits the activity of transforming growth factor β inducible factor 1 (TGIF1) and promotes the activation of TGF-β1/Smad2/3 signals (29). The reduction of NR4A1 expression is related to glucose metabolism disorders and renal fibrosis, and histone acetylation can help increase the expression of NR4A1 in patients with DKD (32, 33). We speculate that JMJD1A may regulate the activity of NR4A1 through the demethylation pathway, thereby upregulate the expression levels of fibrosis-related factors through the signal pathway mediated by TGF-β1, and accelerate the pathological progress of diabetic renal fibrosis. In this work, HK-2 treated with AGEs was used as an *in vitro* diabetic renal fibrosis model. The effects of JMJD1A-NR4A1 on fibrosis-related proteins VIM, TGF-β, and CTGF were systematically studied, and through the correlation between JMJD1A-NR4A1 and TGF-β mediated between profibrosis pathways to determine whether JMJD1A-NR4A1 demethylation is a new pathogenic factor involved in the development of diabetic renal fibrosis. Therefore, looking for strategies to regulate the balance of JMJD1A-NR4A1 signaling pathways may be a practical solution for clinical treatment of DKD.

## Materials and Methods

### Animals

Male CD-1 mice weighing approximately 30 to 35 g were purchased from Zhuhai Bastone Biotechnology Co. (Experimental unit use license number: SYXK (Guangdong) 2016-0167). We used unilateral nephrectomy 1 week after injection of streptozotocin (STZ; USP grade, 98.5%; Source Leaf Bio) to establish a diabetic nephropathy model. Mice received 60 mg/kg body weight of STZ intraperitoneally for 5 consecutive days, and blood glucose was measured after 1 week for 12 weeks. Mice were killed 12 weeks after STZ and analyzed for pathological changes in the kidneys, and the results were as follows. This study was approved by the Animal Protection and Utilization Committee of Southern Medical University Experimental Animal Ethics Committee, in accordance with our institutional regulations.

### Masson and Immunohistochemistry Staining

Paraffin-embedded kidney tissue sections were deparaffinized and stained with Masson trichrome. The extent of renal fibrosis was assessed based on the amount of collagen deposition using an inverted microscope (DMi8, Leica, Germany), and collagen quantification was analyzed using ImageJ software. The area of fibrotic lesions was expressed as a percentage of fibrotic area relative to the whole percentage of fibrotic area relative to the whole area. For immunohistochemistry (IHC) staining, paraffin-embedded kidney tissues were baked at 65°C for 2 h and deparaffinized three times in xylene, then rehydrated with alcohol, and washed three times with PBS solution for 5 min each. The sections were placed in sodium citrate liquid for antigenic thermal repair. The sections were closed at room temperature for 1.5 h, then incubated with primary antibody overnight at 4°C, and washed three times with PBS for 5 min each. Primary antibodies were used as follows: rabbit monoclonal antibody to FN (1:400 dilution, Abcam), COL1 (1:1000 dilution, Abcam), and JMJD1A (1:200 dilution, Proteintech). Secondary antibodies were incubated for 20 min at 37°C and washed three times with PBS. Immunostaining was performed using 3,3-diaminobenzidine (DAB), and the staining was observed in real time under the microscope. Sections were counterstained with hematoxylin and finally sealed with neutral gum. Sections (*n* = 5 per animal) were photographed at 200 × magnification using a light microscope (DMi8, Leica, Germany) and analyzed with the ImageJ analysis software.

### Cell Culture and Experimental Design

Renal tubular epithelial cells from an immortalized human proximal tubular epithelial cell line were obtained from the ATCC, which was grown in the MEM and supplemented with 10% fetal bovine serum (FBS; Invitrogen, Carlsbad, Calif., USA). The cells were placed in a CO_2_ incubator at 37 °C and will be taken for the subsequent experimental logarithmic phase. HK-2 cells were grown to 80–90% confluence, digesting with 0.125% trypsin-0.1% EDTA, and seeding on six-well-tissue culture plates. The confluent cells were cultured in 400 μg/ml AGEs after 72 h in serum-free MEM medium for 24 h.

### Real-Time Quantitative Reverse Transcription-Polymerase Chain Reaction

RNAiso Plus (AG RNAex Pro Reagent,China) was used to extract total RNA from HK-2 cells according to the instructions provided by the manufacturer. Subsequently, the PrimeScript™ RT Reagent Kit (TaKaRa Biotech) was used for reverse transcription into cDNA. The RT-qPCR analysis was performed using the Roche LightCycler 96. In all PCR experiments, the expression of β-actin was used as the internal reference.

### Western Blot Analysis

Renal tubular epithelial cells were washed twice with PBS, then lysed on ice for 10 min in 80–100 μl RIPA cell lysis buffer supplemented with protease and phosphatase inhibitors (Roche). Then protein concentration was measured using a BCA protein assay kit. Protein samples (20 μg) were separated by SDS-PAGE and then by a polyvinylidene fluoride membrane (Millipore, IPVH00010). Each membrane was blocked with 5% skim milk for 1 h at room temperature and incubated overnight with the following primary antibody at 4°C, diluted at JMJD1A (Proteintech, 12835-1-AP, 1:2000), VIM (Proteintech, 10366-1-AP, 1:5000), E-cad (Proteintech, 20874-1AP, 1:25000), Collagen-1 (Proteintech, 14695-1-AP, 1:2000), CTGF (Proteintech, 23936-1-AP, 1:5000), a-SMA (Proteintech, 14395-1-AP, 1:5000), and β-actin/GAPDH (Proteintech, 66009-1-lg /60004-1-Ig) as loading control. The membranes were washed out four times in the TBST solution and incubated with their respective horseradish peroxidase–conjugated secondary antibodies for 90 min at room temperature. The ECL Western blotting system (Santa Cruz Biotechnology) was used for detection. The levels of proteins were analyzed using Image J Software.

### Immunofluorescence Staining

Renal tubular epithelial cells were stimulated by AGEs for 72 h and then washed with PBS three times. Cells were fixed with 4% paraformaldehyde (PFA) for 15 min and then permeabilized using 0.1% Triton X-100 for 20 min after washing the plates with PBS and blocking with 5% BSA in PBS for 2 h at room temperature and then incubating with antibody against JMJD1A(Proteintech,12835-1-AP,1:300), VIM (Proteintech, 10366-1-AP,1:300), and a-SMA (Proteintech, 14395-1-AP, 1:400) overnight at 4 °C. On the second day, slides were washed in PBS and the sections were incubated with the appropriate secondary antibodies (Proteintech, SA00013-4,1:200) (Proteintech, SA00013-3,1:200) for 1 h at room temperature. In the final step, the coverslips were washed and mounted on the slides with Antifade Mounting Medium with DAPI. The images were captured using fluorescent inverted microscope (DMi8, Leica, Germany).

### RNA Isolation, Library Construction, and Sequencing RNA

RNAex Pro Reagent (AG, Hunan, China) was used to extract total RNA from HK-2 cells according to the instructions provided by the manufacturer. A total amount of 2 μg isolated RNA per sample was used for DNA library preparation. Use NEBNext^®^ UltraTM RNA Library Preparation Kit for Illumina^®^ to generate a sequencing library (Neb, Ipswich, Massachusetts, USA) in accordance with the manufacturer's instructions. The libraries were pooled in equimolar amounts and sequenced using a 2 ×132 bp chemical method (Nova seq, Illumina). The RNA sequencing (RNA seq) data reported in this study has been saved in NCBI's comprehensive gene expression database (accession number: GSE193192). Quality control of RNA-seq raw paired data was performed using FastQC (http://www.bioinformatics.babraham.ac.uk/projects/fastqc) and MultiQC. Trim Galore (https://github.com/FelixKrueger/TrimGalore) was used to trim adapters, reads with low quality (<50), and short length (<75 bp). RNA-seq reads were mapped to *Homo sapiens* genome Ensembl GRCh38 using Hisat2 (version 2.1.0) with default codes. Sam files were transformed into bam files using samtools (version 1.9). The read counts of each gene were summarized using featureCounts (version 1.6.5). Raw read counts were imported into R studio (version 3.6.1) and analyzed by using R package of DESeq2 (version 1.26.0). Genes with foldchange > 2 and false discovery rate *p* < 0.05 that were adjusted by using ClusterProfile method were considered as differentially expressed genes (DEGs).

### Lentivirus-Mediated Knockdown and Overexpression of JMJD1A in HK-2

Renal tubular epithelial cells were subjected to lentiviral transduction lentiviral particles (shRNA Lentiviral Transduction; Umine Biotechnology Co, LTD Guangzhou) with sequence targeting human JMJD1A (clone ID humanNM_018433, sequence, GATCCCCCTAATAACTGTTCAGGAAACTTCCTGTCAGATTTCCTGAACAGTTATTAGGGTTTTTG) through the selection of green fluorescent protein and RT-qPCR technology to select the appropriate MOI value for cell, and to prepare for the follow-up experiment. The human JMJD1A the empty lentiviral vector (vector group) and lentiviral vector (JMJD1A group) were constructed at Hanbio company (China). HK-2 were transfected with lentivirus medium at a multiplicity of infection (MOI=50), and 2μg/ml polybrene was added to promote efficiency. After 48 h of infection transfection efficiency was determined by RT-qPCR.

### Statistical Analysis

According to unpaired t tests and one-way ANOVA or two-way ANOVA to analyze statistics using by GraphPad Prism 7, data were considered statistically significant at *p* < 0.05.

## Results

### Expression of JMJD1A in the Kidney Structure of DKD Mice

**Figure 1:**
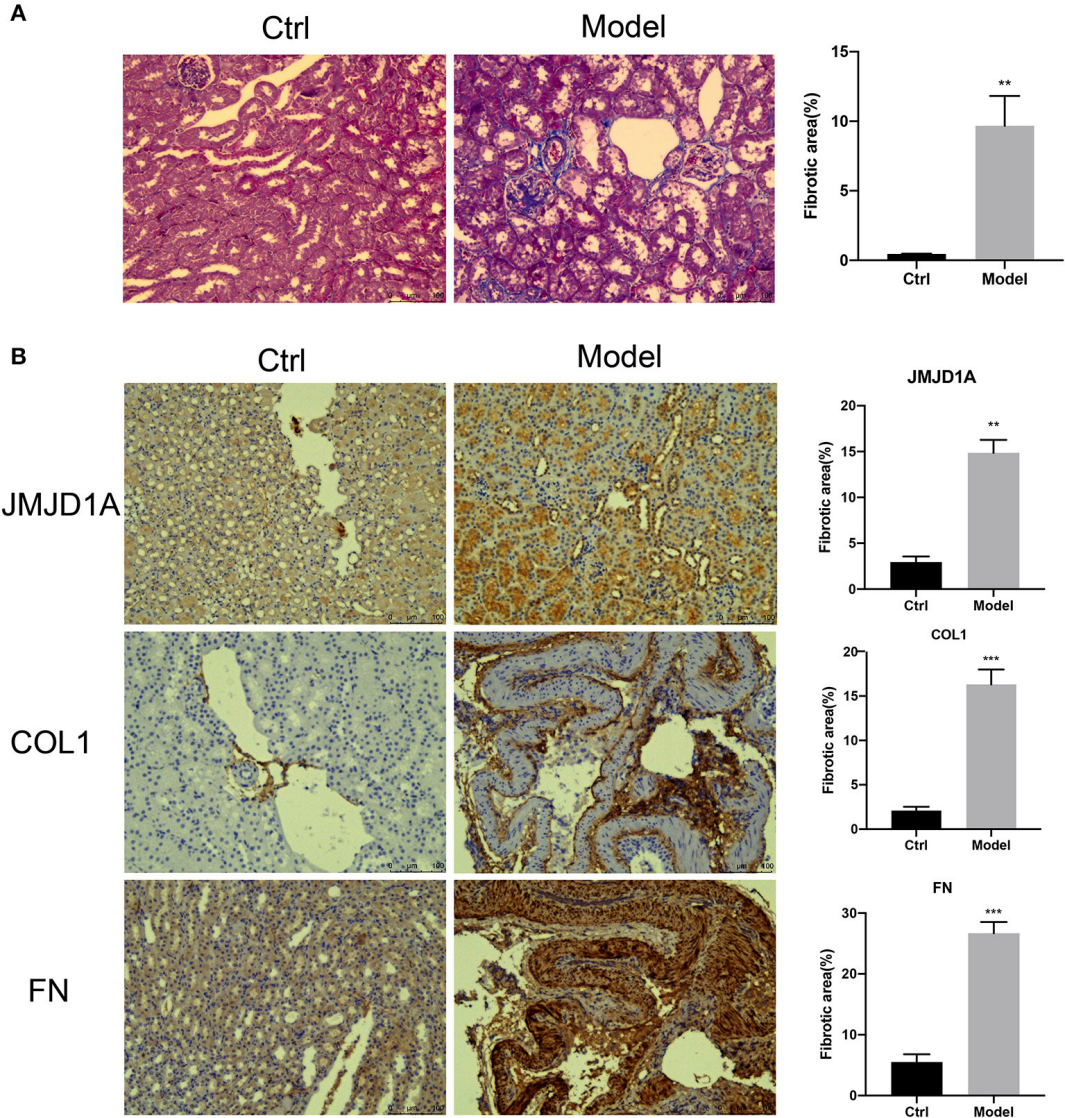
**(A)** Representative images of unilateral nephrectomized mice with diabetic nephropathy combined with STZ vs. normal control mice showing glomerular collagen deposition (Masson trichrome staining) (scale bar, 100 μm, ×200, *n* = 5, **p < 0.001). **(B)** Quantification was performed using ImageJ analysis software. Kidney sections from both groups of mice were stained by IHC to detect the expression of JMJD1A, FN, COL1 (scale bar, 100 μm, ×200, *n* = 5, **p < 0.001, ***p < 0.0001).

Collagen-specific Masson trichrome staining showed glomerular changes with a significant increase in collagen deposition (Figure 1A). Immunohistochemical methods were used to detect the protein expression of FN and COL1 in DKD kidneys. The expression of FN, JMJD1A, and COL1 were significantly increased in the DKD group compared with the control group (Figure 1B).

### AGEs Dose-Dependently Accelerate the HK-2 Cell Damage

**Figure 2:**
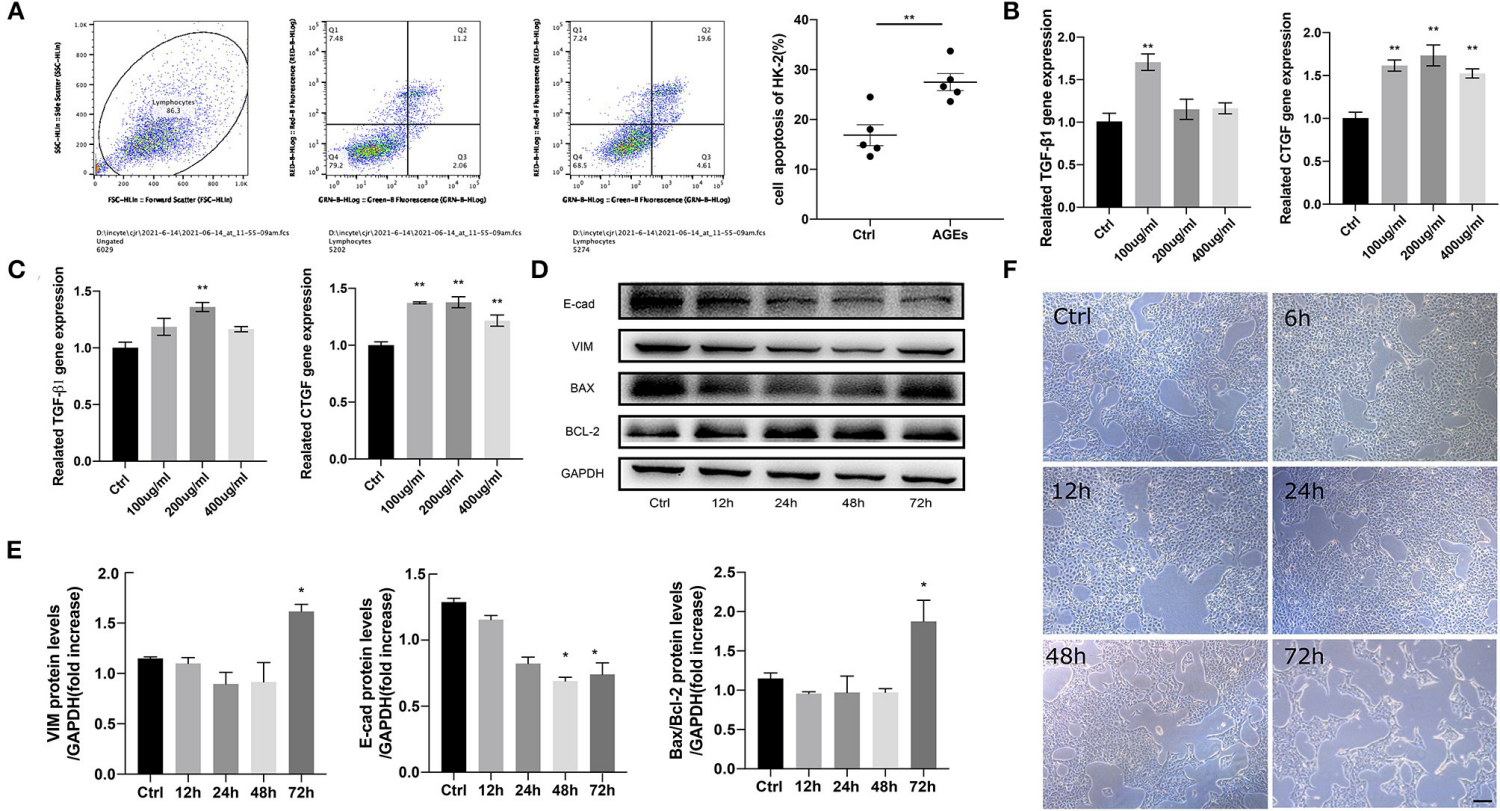
AGEs accelerates the injury of HK-2 cells in a dose-dependent or time-dependent manner. **(A)** Flow cytometry analysis of apoptosis of HK-2 cells. **(B,C)** Reverse transcription-quantitative polymerase chain reaction (RT-qPCR) was used to detect the mRNA expression of 48, 72 h fibrosis related factors TGF-β1 and CTGF in HK-2 cells (*n* = 3, **p < 0.001). **(D,E)** Western blot was used to detect the protein expression of fibrosis and apoptosis related factors in HK-2 cells stimulated by 400 μg/ml AGEs for 12, 24, 48, and 72 h (*n* = 3, *p < 0.05). **(F)** The morphological changes of HK-2 cells in Ctrl group and 400 μg/ml AGEs stimulation group 72 h later (scale bar, 100 μm).

To observe the effects of different concentrations of AGEs on HK-2 cells, they were divided into Ctrl group and AGEs group including 100, 200, and 400 μg/ml. The morphological changes of HK-2 cells were observed after 72 h. The results showed that HK-2 cells in the Ctrl group were oval and spindle-shaped, but in HK-2 cells that were treated with 100, 200, and 400 μg/ml AGEs group, and especially at 400 μg/ml, the cell morphology changed significantly (Figure 2F). We found that the cell body of HK-2 cells was elongated, the cell gap was enlarged, showing a long spindle shape, and there were many cell fragments in the cell supernatant. Therefore, 400 μg/ml AGEs group significantly destroyed the morphology of HK-2 cells and may induce apoptosis. Therefore, we analyzed the level of apoptosis by flow cytometry and found that the level of apoptosis increased after 72 h of AGEs stimulation (Figure 2A). Moreover, RT-qPCR was used to detect in HK-2 cells mRNA expression level at 48 and 72 h, and observed that the expression of fibrosis-related factors TGF-β1 and CTGF in tissues was enhanced (Figures 2B,C). Furthermore, stimulation of HK-2 cells with 400 μg/ml AGEs of 12, 24, 48, and 72 h clearly resulted in related-fibrosis proteins along with apoptosis proteins, which increased at 72 h significantly (Figures 2D,E).

### Sequencing of the Transcriptome of HK-2 Induced by AGEs

**Figure 3:**
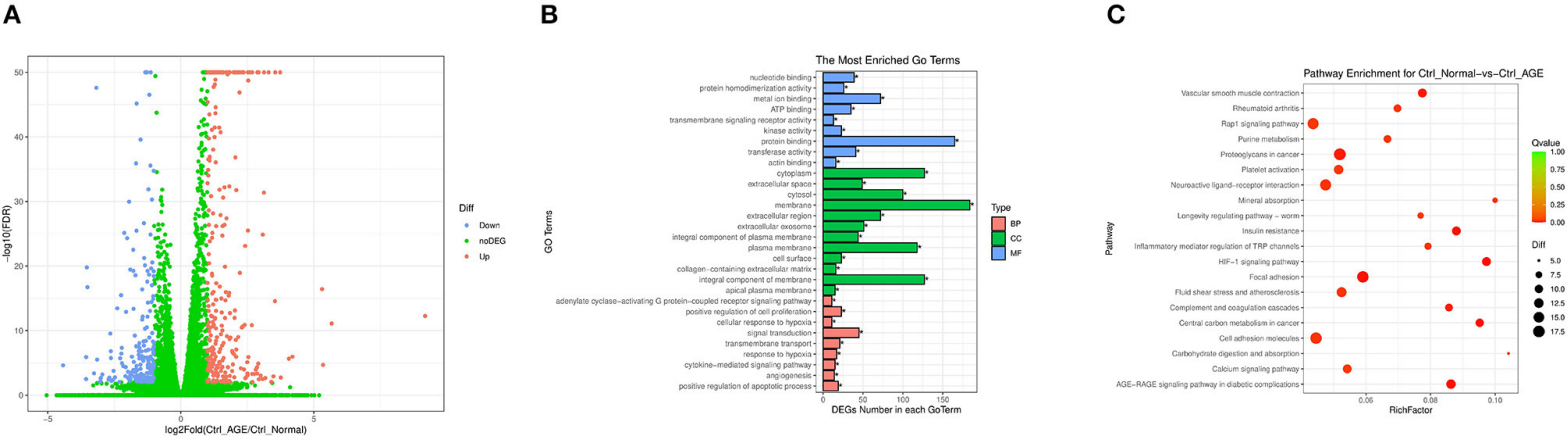
**(A)** Cluster map and volcano map of differentially expressed genes in Ctrl group and AGEs group in HK-2 cells; **(B)** Gene ontology analysis enriches the number distribution position of differential genes; **(C)** Kyoto Encyclopedia of Genes and Gene Pathways (KEGG) analyzes metabolic pathways that are significantly enriched in differentially expressed genes in the Ctrl group and the AGEs group.

To reveal that JMJD1A is a potential mechanism to promote renal tubular fibrosis, we analyzed the gene expression profile of HK-2 stimulated by AGEs by high-throughput RNA sequencing. We selected Ctrl group and AGEs group and then analyzed the DEGs with *p* < 0.05 under the screening condition of fold change ≥2. We obtained 284 upregulated genes and 193 downregulated genes, and plotted the cluster and volcano plots of the DEGs, from which can it be seen that there are obvious gene differences between Ctrl group and AGEs group (Figure 3A). Gene ontology gene ontology analysis (GO) is done by GOseq software to intuitively reflect the number distribution of differential genes enriched in biological process (BP), molecular function (MF), and cellular component (CC). The top 10 GO terms with the highest enrichment factor are shown in Figure 3B. It is obtained from BP results, differential genes can be enriched in extracellular matrix organization and lipid metabolic processes (Figure 3B). In addition, in order to explore the effect of AGEs on cell pathways, we used Kyoto Encyclopedia of genes and genes pathways (KEGG) to analyze the metabolic pathways significantly enriched by DEGs in Ctrl group and AGEs group. A total of 276 pathways were established, of which 46 pathways had *p* < 0.05, and the top 20 pathways were shown in the Figure 3C. The top ten pathways are HIF-1 signaling pathway, AGE-RAGE signaling pathway in diabetic complications, insulin resistance, central carbon metabolism in cancer, focal adhesion, vascular smooth muscle contract, proteoglycans in cancer, complement and coagulation cascades, inflammatory mediator regulation of TRP channels, and rheumatoid arthritis.

### RNA Sequencing Revealed That JMJD1A May Be a Potential Epigenetic Regulator in the Progression of Fibrosis Induced by AGEs in HK-2 Cells

**Figure 4:**
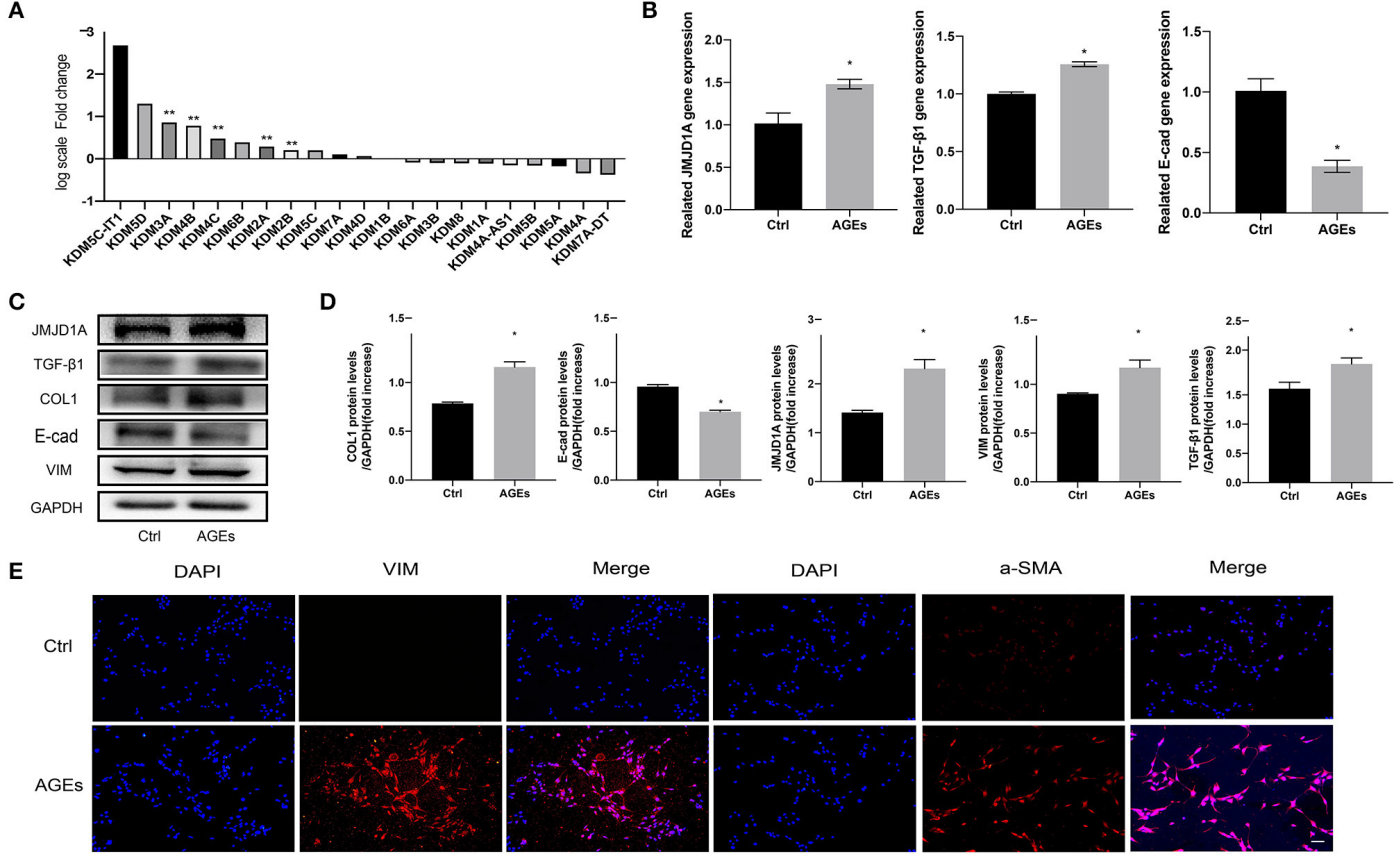
JMJD1A may be an epigenetic regulator of AGEs-induced fibrosis in HK-2 cells. **(A)** Quantitative gene expression changes in JMJD families detected by RNA sequencing (*n* = 3, **p < 0.001). **(B)** HK-2 cells were stimulated with 400 μg/ml AGEs for 72 h, and the expression of JMJD1A, TGF-β1, and E-cad was detected by RT-qPCR (*n* = 3, *p < 0.05). **(C,D)** Western blot was used to detect the expression of fibrosis-related factors (JMJD1A, TGF-β1, COL1, E-cad, VIM) in HK-2 (*n* = 3, *p < 0.05). **(E)** Immunofluorescence was used to detect the expression of fibrosis-related factors a-SMA and VIM (scale bar, 100μm).

Moreover, we detected the transcriptional profile of H3K9 demethylase KDM family by RNA sequencing. We counted the expression of all genes in KDM family and found that the top three genes were KDM5C-LT1, KDM5D, and KDM3A, but the former two were not statistically significant. KDM3A showed the greatest correlation in RNA-seq and had statistical significance, *p* < 0.001 (Figure 4A). This has also been proved to play a key regulatory role in fibrosis of diabetic nephropathy. To explore whether JMJD1A will affect the expression of HK-2 fibrosis related indicators, we induced it with AGEs to further confirm the difference of JMJD1A expression. We stimulated HK-2 cells with AGEs at a concentration of 400 μg/ml for 72 h. Compared with the Ctrl group, RT-qPCR showed that JMJD1A was significantly upregulated at 72 h, whereas TGF-β1 and other related expression levels were significantly increased (Figure 4B). In addition, Western blot to assess the expression of fibrosis-related factors (TGF-β1, COL1, E-cad, VIM) in HK-2. We found that the expression level of AGEs group was significantly increased (Figures 4C,D). Immunofluorescence assay results showed that fibrosis-associated factors a-SMA and VIM were significantly increased in the AGEs group compared with the Ctrl group (Figure 4E).

### Knockout of JMJD1A Inhibited the Expression of Fibrosis-Related Factors in HK-2 Cells Stimulated by AGEs

**Figure 5:**
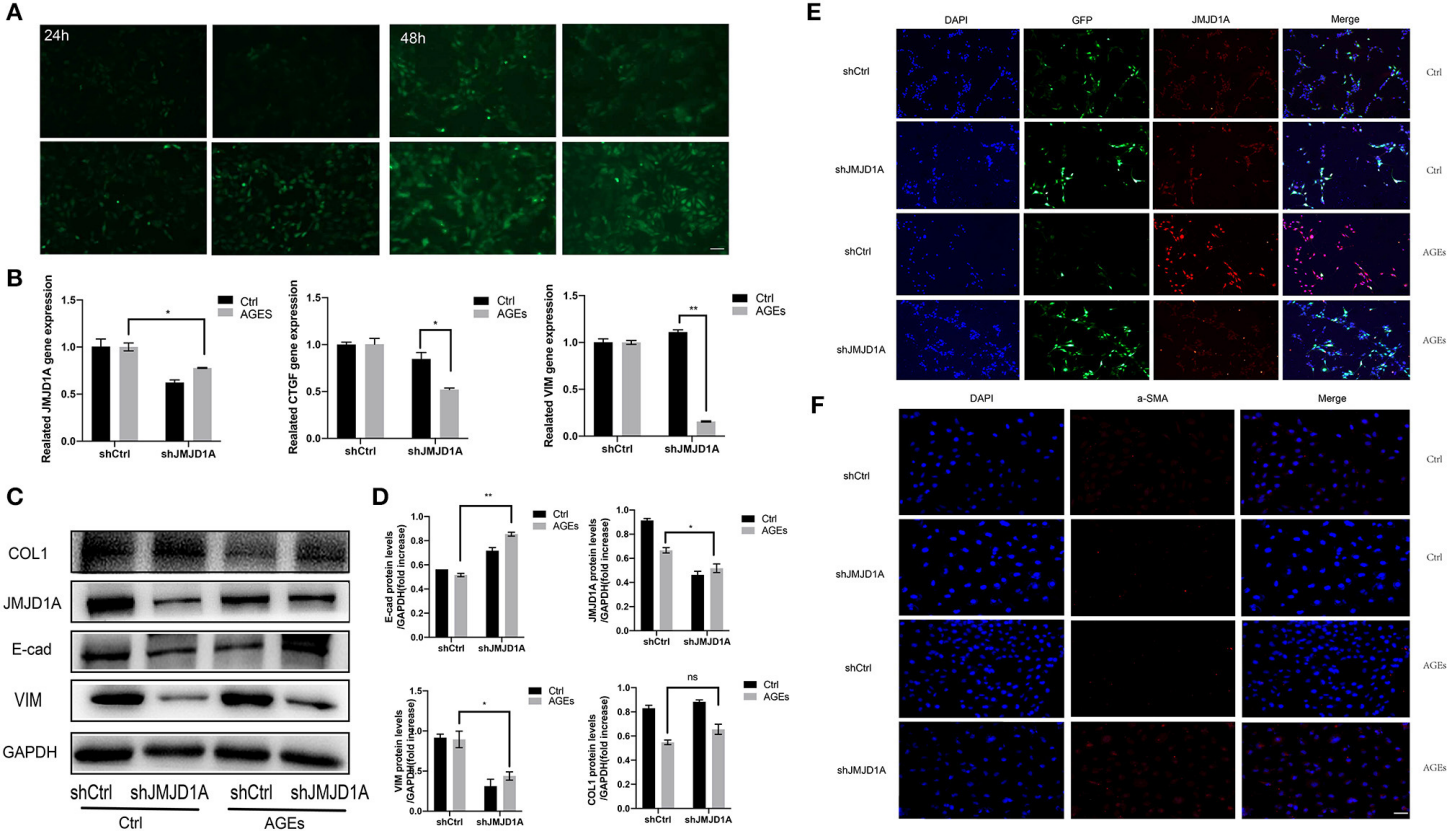
Knockout of JMJD1A inhibited the expression of fibrosis-related factors in HK-2 cells stimulated by AGEs. **(A)** The JMJD1A knockout lentivirus vector was successfully constructed and stably transfected into HK-2 cells. The virus infection efficiency was observed by inverted fluorescence microscope. **(B)** After cultured with 400 μg/ml AGEs for 72 h, the expressions of JMJD1A, CTGF, and VIM in HK-2 cells stably transduced by shJMJD1A and shCtrl were detected by RT-qPCR (*n* = 3, *p < 0.05, **p < 0.001). **(C,D)** After being cultured with 400 μg/ml AGEs for 72 h, the expressions of COL1, JMJD1A, E-cad, and VIM in HK-2 cells stably transduced by shJMJD1A and shCtrl were detected by Western blot (*n* = 3, *p < 0.05, **p < 0.001). **(E,F)** Immunofluorescence was used to detect the expression of fibrosis-related factors JMJD1A and a-SMA, compared with the AGEs shCtrl group, the fibrosis related factors a-SMA and JMJD1A in AGEs shJMJD1A group were significantly lower (scale bar, 100 μm).

Although the role of fibrosis kidney injury in diabetic vascular disease has been most frequently studied, the role of histone H3K9 demethylation of JMJD1A in this process is less well-studied. Therefore, we aimed to analyze whether JMJD1A plays a major regulatory role in the progression of renal fibrosis. We successfully constructed lentivirus vector by introducing green fluorescent protein (GFP) scrambling control and GFP-shJMJD1A into cells (Figure 5A). The virus infection efficiency was observed by inverted fluorescence microscope (Figure 5A). The fluorescence expression of transfected cells was the strongest at 48 h and MOI = 50. At the same time, the expression of JMJD1A gene in shCtrl group and shJMJD1A group stimulated by AGEs was detected by fluorescence quantitative PCR. The picture shows that the expression of JMJD1A gene in shJMJD1A group was lower than that in shCtrl group, suggesting that JMJD1A silencing HK-2 strain was successfully constructed (Figure 5B). In order to further verify the role of JMJD1A in fibrosis induced by AGEs, we detected the expression levels of related inflammatory markers VIM, COL1, E-cad, CTGF, and TGF-β1 by RT-qPCR, Western blot, and immunofluorescence. The results showed that JMJD1A silencing can significantly reduce the expression level of related fibrosis indexes induced by AGEs (Figures 5B–D). Moreover, immunofluorescence assay results showed that fibrosis associated factors a-SMA and JMJD1A were significantly decreased in the AGEs shJMJD1A group compared with the shCtrl group (Figures 5E,F). Combined with previous studies, knockout of JMJD1A can reduce AGEs induced fibrosis factors.

### Overexpression of JMJD1A Accelerates the Expression of Fibrosis Related Factors in AGEs Stimulated HK-2 Cells

**Figure 6:**
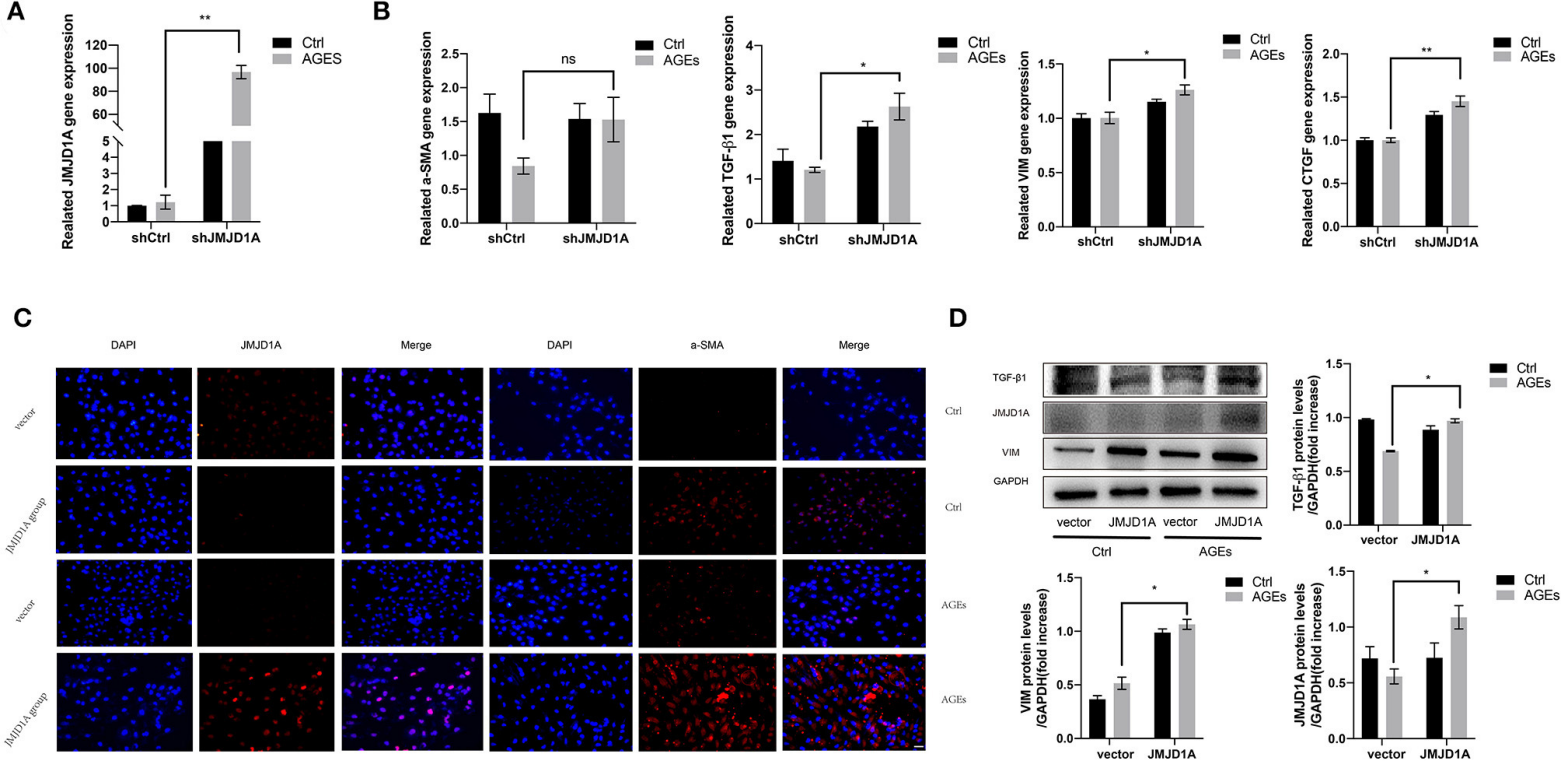
Overexpression of JMJD1A accelerates the expression of fibrosis related factors in AGEs stimulated HK-2 cells. The JMJD1A lentiviral vector was constructed and transfected into HK-2 cells to obtained the HK-2 cell line stably transfected with overexpression of JMJD1A. **(A,B)** After being cultured with 400 μg/ml AGEs for 72 h, RT-qPCR detection of the mRNA expressions of JMJD1A, a-SMA, TGF-β1, VIM, and CTGF in the JMJD1A group increased compared with vector group (*n* = 3, *p < 0.05, **p < 0.001). **(C)** The expression level of a-SMA in the AGEs JMJD1A group detected by immunofluorescence increase compared with AGEs vector group (scale bar, 100 μm). **(D)** The protein expression level of TGF-β1, VIM, and JMJD1A in the AGEs JMJD1A group detected by Western blot increased compared with AGEs vector group (*n* = 3, *p < 0.05).

To further confirm the effect of upregulated JMJD1A in DKD on the expression level of the above-mentioned fibrosis markers, we constructed JMJD1A lentiviral vector and transfected it into HK-2 cells and obtained HK-2 cell line stably transfected with overexpression of JMJD1A. The mRNA level of JMJD1A increased significantly (Figure 6A). Through RT-qPCR and Western blotting analysis, the overexpression of JMJD1A enhanced the expression of CTGF, VIM, and TGF-β1 in HK-2 cells compared with control vector transfection (Figures 6B,D). Immunofluorescence assay results showed that fibrosis associated factors a-SMA and JMJD1A were significantly increased in the AGEs JMJD1A group compared with the AGEs vector group (Figure 6C). Therefore, we inferred that JMJD1A plays an important role in the stimulation of AGEs to induce fibrotic injury by the detection of knockdown and overexpression of JMJD1A and fibrosis related factors.

### RNA-Seq Revealed That JMJD1A Knockdown May Ameliorate the Kidney Fibrosis *via* NR4A1

**Figure 7:**
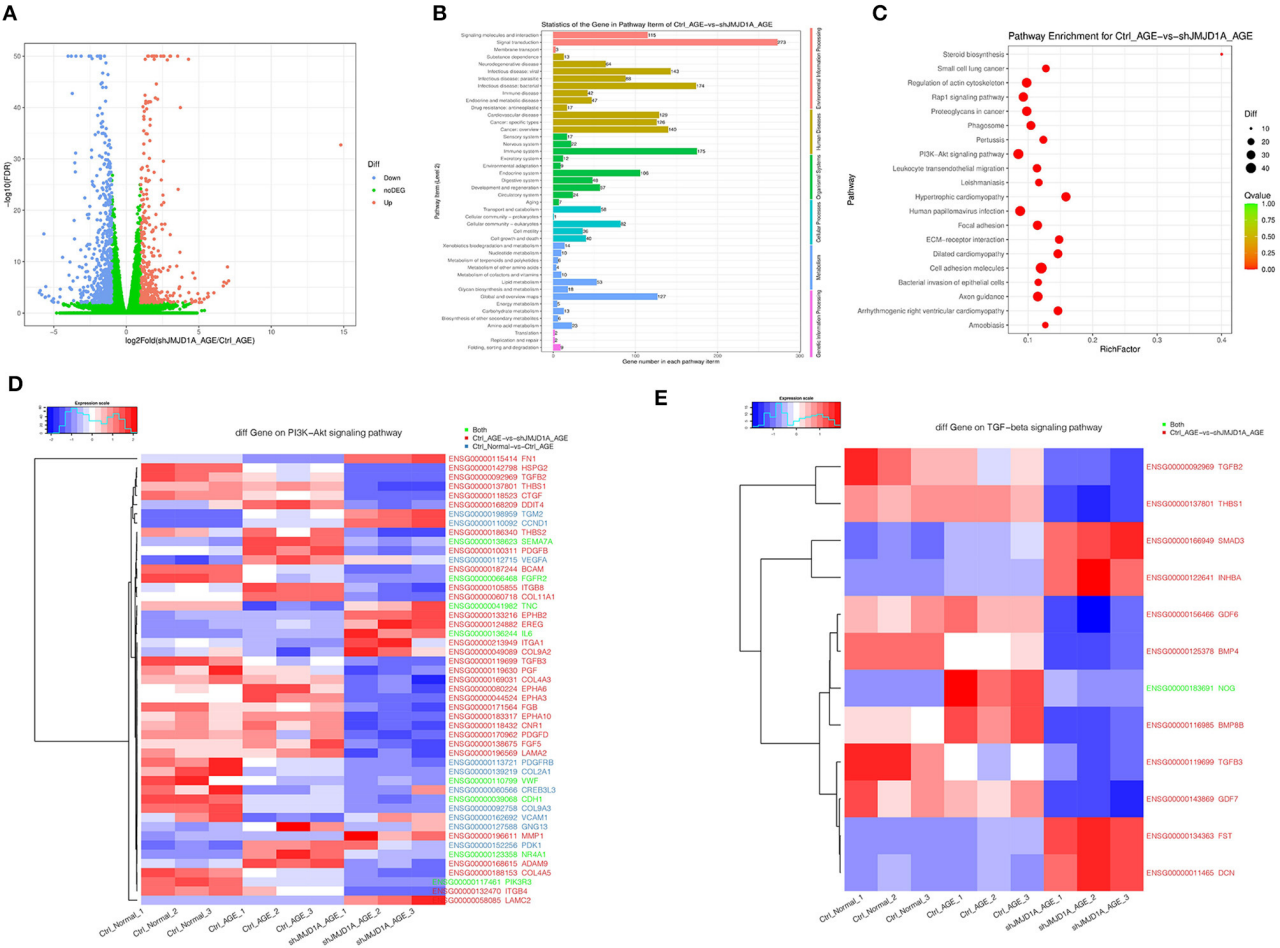
Gene expression and heatmaps of HK-2 cultured with 400 μg/ml AGEs for 72 h by RNA-seq. **(A)** Volcano plot was used to show the difference in gene expression values between the Ctrl_AGEs group and the shJMJD1A_AGEs group. Green dotted line indicates the threshold for *p* < 0.05 and log FC <2. Blue and red points represent downregulated and upregulated DEGs, respectively. **(B,C)** GO and KEGG enrichment analysis of differentially expressed genes (correct *p* < 0.05). Red bubbles show KEGG pathways. **(D,E)** Heatmaps of differentially expressed genes related to the PI3K-Akt and TGF- β signaling pathway.

We studied transcription factors involved in JMJD1A induction under AGEs stimulation. In RNA-seq analysis, we further analyzed that after silencing JMJD1A, compared with the empty vector transfection group, 376 genes were upregulated and 617 genes were downregulated (Figure 7A). Then, gene ontology analysis (GO) of gene related biological process was carried out (Figure 7B). The results showed that the top 10 response process of HK-2 after silencing JMJD1A gene were mainly related to membrane, integral component of membrane, extractive region, plasma membrane, protein binding and extractive space, extracellular exosome, integral component of plasma membrane, collagen containing extracellular matrix, and extracellular matrix.

Besides, we performed KEGG pathway enrichment analysis to further understand the function of the gene and its interaction with cells containing JMJD1A gene knockout. Then 313 pathway items were found, including 64 pathway items, *p* < 0.05 (Figure 7C). It can be enriched to hypertrophic cardiomyopathy, cell adhesion molecules, arrhythmogenic right ventricular cardiomyopathy, ECM-receptor interaction, steroid biosynthesis, axon guidance, focal adhesion, small cell lung cancer, pertussis.

**Figure 8:**
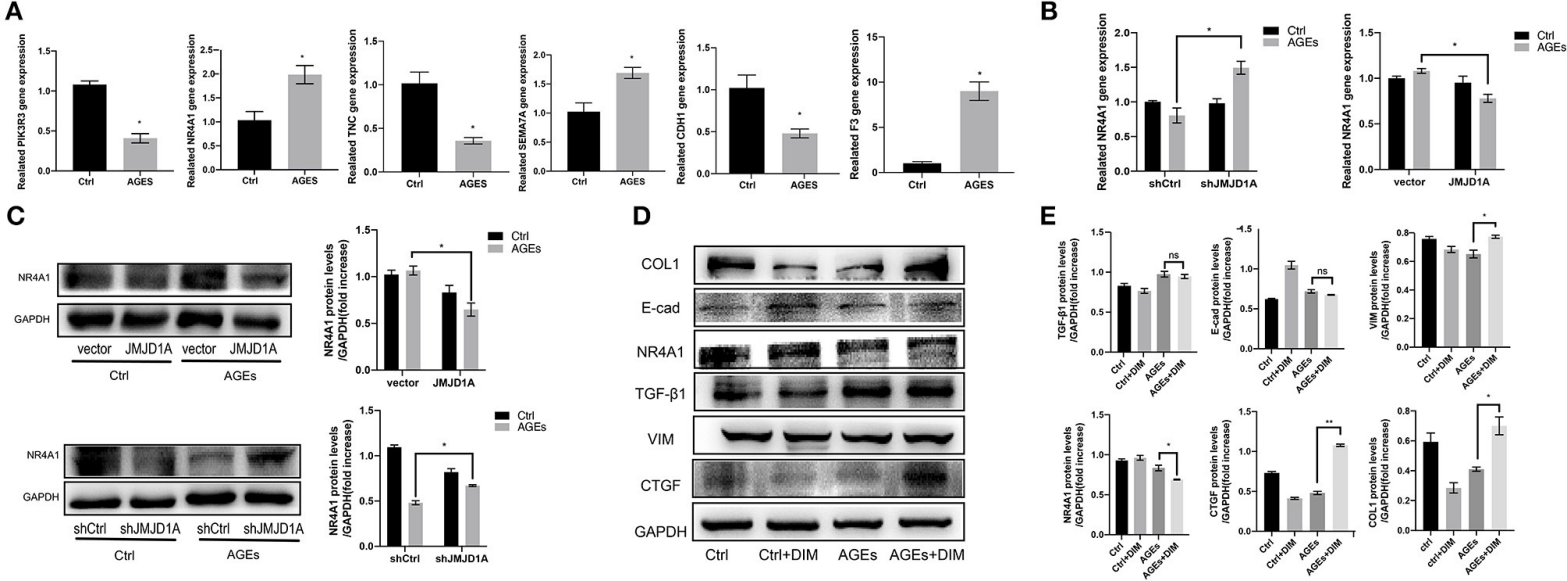
NR4A1 may be the downstream signal factor of JMJD1A regulating HK-2 cell fibrosis. **(A)** The expression of transcriptome predictors (PIK3R3, NR4A1, TNC, SEMA7A, CDH1, and F3) after JMJD1A knockout in HK-2 cells by RT-qPCR(*n* = 3, *p < 0.05). **(B,C)** RT-qPCR and Western blot were used to detect the mRNA and protein expression of NR4A1 in shJMJD1A and JMJD1A group under AGEs stimulation, compared with shCtrl or vector group, NR4A1 was upregulated in shJMJD1A group, but down regulated in JMJD1A group. **(D,E)** NR4A1 was inhibited by the inhibitor DIM-C-pPhCO2Me (20 μM), Western blot detected the related fibrosis, and the expression levels of VIM, TGF-β1, CTGF, and COL1, which were higher in AGEs + DIM group compared with AGEs group (*n* = 3, *p < 0.05, **p < 0.001).

To further explore the mechanism of silencing JMJD1A to reverse the damage of HK-2 treated by AGEs, we selected gene sets related to the JMJD1A downstream pathway through Ctrl group, AGEs group, and shJMJD1A AGEs to reverse the damage. KEGG result analysis is mainly distributed in AGE-RAGE signaling pathway in diabetic complications, Rap1 signaling pathway, PI3K-Akt signaling pathway, VEGF signaling, and TGF-β signaling pathway. Our assays for PI3K-Akt and TGF- β the differential genes among these two pathways are shown (Figures 7D,E). Next, the predicted transcription factors were detected by RT-qPCR. The results showed that the gene expression levels of F3, SEMA7A, NR4A1, CDH1,PIK3R3, and TNC were consistent with the transcriptome sequencing results. The gene expression levels were significantly increased after AGEs stimulation, and the differences were statistically significant (Figure 8A).

### RNA-Seq Revealed That JMJD1A Knockdown May Ameliorate the Kidney Fibrosis *via* NR4A1

We evaluated NR4A1 expression by RT-qPCR and Western blot in JMJD1A knockdown and overexpression stimulated by AGEs. We found that NR4A1 was upregulated at the mRNA or protein level when JMJD1A was knocked down, but downregulated when JMJD1A was overexpressed (Figures 8B,C). These implies that NR4A1 shows an opposite trend to the expression of fibrosis related factors, which is compatible with previous studies. To explore whether NR4A1 can participate in JMJD1A-mediated and AGES-induced renal tubular fibrosis injury, we inhibited NR4A1 with the inhibitor DIM-C-pPhCO2Me (20 μM). In addition, we detected the related fibrosis and the expression levels of VIM, TGF-β1, CTGF, and COL1 by Western blot (Figure 8D). The results showed that the related fibrosis expression level in DIM group by AGEs was higher than that in AGEs group (Figures 8D,E). Combined with previous research results, downregulation of NR4A1 can promote renal tubular fibrosis and EMT related indicators, and accelerate the process of fibrosis.

## Discussion

Diabetic kidney disease, one of the common clinical microvascular complications of diabetes, is an important cause of death in patients with end-stage renal disease and diabetes (34, 35). Renal fibrosis is the main pathological feature in the development stage of DKD (36). Many factors can lead to renal fibrosis in DKD, such as AGEs, mitochondrial dysfunction, autophagy dysfunction, activation of inflammatory pathway and so on (37–39). AGEs is considered to be an important factor in the process of DKD renal fibrosis, which can promote the process by stimulating the secretion of oxygen free radicals, cytokines, chemokines, adhesion molecules, TGF-β, CTGF, and other mediators (15, 40). Therefore, the current study is conducted to reveal the mechanism of renal tubulointerstitial fibrosis induced by AGEs with the conclusion pronouncing that AGEs reduce the fibrosis progression in DKD through the JMJD1A/NR4A1 axis.

In this work, HK-2 cells were stimulated with 400 μg/ml AGEs for 72 h to successfully induce *in vitro* diabetic renal fibrosis model. The gene and protein expression levels of profibrosis factors such as TGF-β1, CTGF, and VIM were detected by fluorescence quantitative PCR and Western Blot, which were consistent with the characteristics of the progression of DKD to renal fibrosis. This indicates that the renal fibrosis model of HK-2 cell DKD was successfully established. JMJD1A, a member of the JMJD (containing JmjC domain) protein family, can promote glycolysis by coactivating glucose through hypoxia-inducible factor-1α (41, 42). Recently, it has been found that miR-101a can prevent renal fibrosis by inhibiting the expression of JMJD1A (43). In addition, JMJD1A has been confirmed to activate TIMP1 and secrete it into the ECM of cardiomyocytes, thereby promoting myocardial fibrosis (44). This suggests that there may be a correlation between JMJD1A and fibrosis. Our *in vitro* experiments showed that the expression of JMJD1A gene and protein in HK-2 cells increased significantly after HK-2 was stimulated with AGEs for 72 h. However, the relationship between JMJD1A and renal fibrosis has rarely been studied. Through the analysis of RNA-Seq data, we found that most of the gene expression of JMJD family changed during the fibrosis of HK-2 cells stimulated by AGEs, among which the expression level of JMJD1A gene was the most significant, about 1.8 times higher than that of the control group.

Furthermore, we used lentivirus to construct shJMJD1A HK-2 model to explore the role of JMJD1A in DKD renal fibrosis under AGEs stimulation. The results showed that compared with the HK-2 model transfected with the empty lentiviral vector (JMJD1A vector group), the gene and protein expression levels of TGF-β, CTGF, and VIM in HK-2 cells in shJMJD1A group decreased, which slowed down the fibrosis process of HK-2 cells to a certain extent. In the subsequent experiments, we successfully transfected HK-2 cells with JMJD1A overexpression lentivirus. Compared with the JMJD1A vector group, the gene and protein expression levels of TGF-β, CTGF, and VIM genes in HK-2 cells of lentivirus group increased, which in turn promotes the fibrosis process of HK-2 cells. This further confirmed that JMJD1A plays a crucial role in AGEs-mediated diabetic renal fibrosis in HK-2 cells. In addition, we used CD-1 mice to induce a DKD model by intraperitoneal injection of STZ 1 week after unilateral nephrectomy. Compared with the normal group, the Masson staining results of the model group showed an increase in the area of collagen fiber deposition in the kidney, and IHC immunohistochemical staining showed that the expressions of JMJD1A, FN, and COL1 in kidney tissue sections were all increased, which is consistent with the results of our *in vitro* experiments.

Some studies have found that higher levels of NR4A1 are closely related to glucose metabolism disorder, renal insufficiency, renal hypertrophy, and fibrosis, and contribute to the occurrence and development of DKD (45, 46). To further explore the downstream mechanism of JMJD1A involved in fibrosis, we used high-throughput sequencing to detect the related genes of HK-2 cells stimulated by AGEs after JMJD1A knockout and obtained the relevant downstream transcription factors, in which NR4A1 was selected for follow-up verification. In chronic kidney disease, the deficiency of NR4A1 has been proved to be involved in the process of renal injury and renal dysfunction. NR4A1 deficient rats [tawny hypertensive rats (FHH)] developed under the genetic background of being susceptible to renal injury showed that compared with the FHH control group, the severity of renal tubular atrophy, renal tubule morphology, and interstitial fibrosis were significantly increased, accompanied by a significant increase in macrophage infiltration and upregulation of inflammatory pathway (33). In the experiment, we found that both JMJD1A and NR4A1 were upregulated under the stimulation of AGEs alone, which may be due to the temporary upregulation of NR4A1 expression induced by the related fibrosis factor TGF-β, resulting in a negative feedback loop, which is consistent with previous studies (47). However, after the knockout of JMJD1A, NR4A1 still showed an upward trend, and the related fibrosis factors were downregulated; after JMJD1A overexpression, NR4A1 factors were downregulated and fibrosis factors were upregulated. Next, we silenced the expression of NR4A1 with inhibitors and detected the expression of related profibrosis factors by Western Blot technology. The results showed that the gene and protein expression levels of TGF-β, CTGF, and VIM were all increased, and the degree of cell fibrosis was increased. Based on these findings, we speculate that JMJD1A regulates renal tubular epithelial fibrosis induced by AGEs through the function of NR4A1.

## Conclusion

To sum up, AGEs may regulate the fibrosis in the progression of DKD through the JMJD1A/NR4A1 axis. In addition, this regulation is accomplished by reducing the expression of JMJD1A and then upregulating the level of NR4A1, thus inhibiting the fibrosis process of renal tubular epithelial cells. These results suggest that JMJD1A/NR4A1 pathway may play an important role in renal fibrosis and provide a new therapeutic strategy for the treatment of renal diseases. However, the regulatory role of JMJD1A/NR4A1 pathway *in vivo* needs to be further verified in future.

## Data Availability Statement

The datasets presented in this study can be found in online repositories. The names of the repository/repositories and accession number(s) can be found in the article/Supplementary Material.

## Ethics Statement

The animal study was reviewed and approved by Southern Medical University Experimental Animal Ethics Committee.

## Author Contributions

SW, AZ, and WJ conducted experiments to ensure the integrity of the entire research and contributed to the conception and design of this research. SW, AZ, JXie, and HL collected, analyzed data, drafted, and polished the manuscript. MW conceived the research. MW, JSu, and AY participated in the design of this research. XZ and MZ contributed to data and statistical analysis. WS, JXia, and JSh provided supplementary illustrations. MW, JSu, and DL revised the manuscript. All authors have read and approved the final manuscript.

## Funding

This work was supported by National Natural Science Foundation of China, Grant Nos. 82074207, 81774035, and 81974117 and Natural Science Foundation of Guangdong Province, Grant Nos. 2021A1515011502 and 2019A1515010665. Innovation team of chronic kidney disease with integrated traditional Chinese and Western Medicine, Grant No. 2019KCXTD014.

## Conflict of Interest

The authors declare that the research was conducted in the absence of any commercial or financial relationships that could be construed as a potential conflict of interest.

## Publisher's Note

All claims expressed in this article are solely those of the authors and do not necessarily represent those of their affiliated organizations, or those of the publisher, the editors and the reviewers. Any product that may be evaluated in this article, or claim that may be made by its manufacturer, is not guaranteed or endorsed by the publisher.
