# Supplementary material for: JMJD1A/NR4A1 Signaling Regulates the Procession of Renal Tubular Epithelial Interstitial Fibrosis Induced by AGEs in HK-2
Source: Front Med (Lausanne). 2022 Feb 3;8:807694. doi: 10.3389/fmed.2021.807694 (PMC8850412; doi:10.3389/fmed.2021.807694)
Supplement: Supplementary file 2 [file Table_2.DOCX]

Supplementary Material

Supplementary Table 1.Primers used for quantitative RT-PCR.

| **Gene name** | **Forward (5'-3')** | **Reverse (5'-3')** |
| --- | --- | --- |
| CTGF | CAGCATGGACGTTCGTCTG | AACCACGGTTTGGTCCTTGG |
| α-SMA | AAAAGACAGCTACGTGGGTGA | GCCATGTTCTATCGGGTACTTC |
| E-cad | ACAGGATGGCTGAAGGTGAC | GGATGACACAGCGTGAGAGA |
| COL1 | GAGGGCCAAGACGAAGACATC | CAGATCACGTCATCGCACAAC |
| TGF-β1 | CAATTCCTGGCGATACCTCAG | GCACAACTCCGGTGACATCAA |
| F3 | CCCAAACCCGTCAATCAAGTC | CCAAGTACGTCTGCTTCACAT |
| SEMA7A | CACCAAGACCAGGCTTACGAT | ACACGGGACACATTGAGAGGA |
| VIM | GCTGCGAGAGAAATTGCAGGA | CCACTTTCCGTTCAAGGTCAAG |
| JMJD1A | GTGTGTGGAATTTGATGGG | CAGCTTTGTCCAACAGAGG |
| PIK3KR3 | TACAATACGGTGTGGAGTATGGA | TCATTGGCTTAGGTGGCTTTG |
| TNC | TCCCAGTGTTCGGTGGATCT | TTGATGCGATGTGTGTGTGGAGACA |
| CDH1 | ATTTTTCCCTCGACACCCGAT | TCCCAGGCGTAGACCAAGA |
| NR4A1 | ATGCCCTGTATCCAAGCCC | GTGTAGCCGTCCATGAAGGT |
