# Supplementary figures and images for: JMJD1A/NR4A1 Signaling Regulates the Procession of Renal Tubular Epithelial Interstitial Fibrosis Induced by AGEs in HK-2 (part 1 of 2)
Source: Front Med (Lausanne). 2022 Feb 3;8:807694. doi: 10.3389/fmed.2021.807694 (PMC8850412; doi:10.3389/fmed.2021.807694)

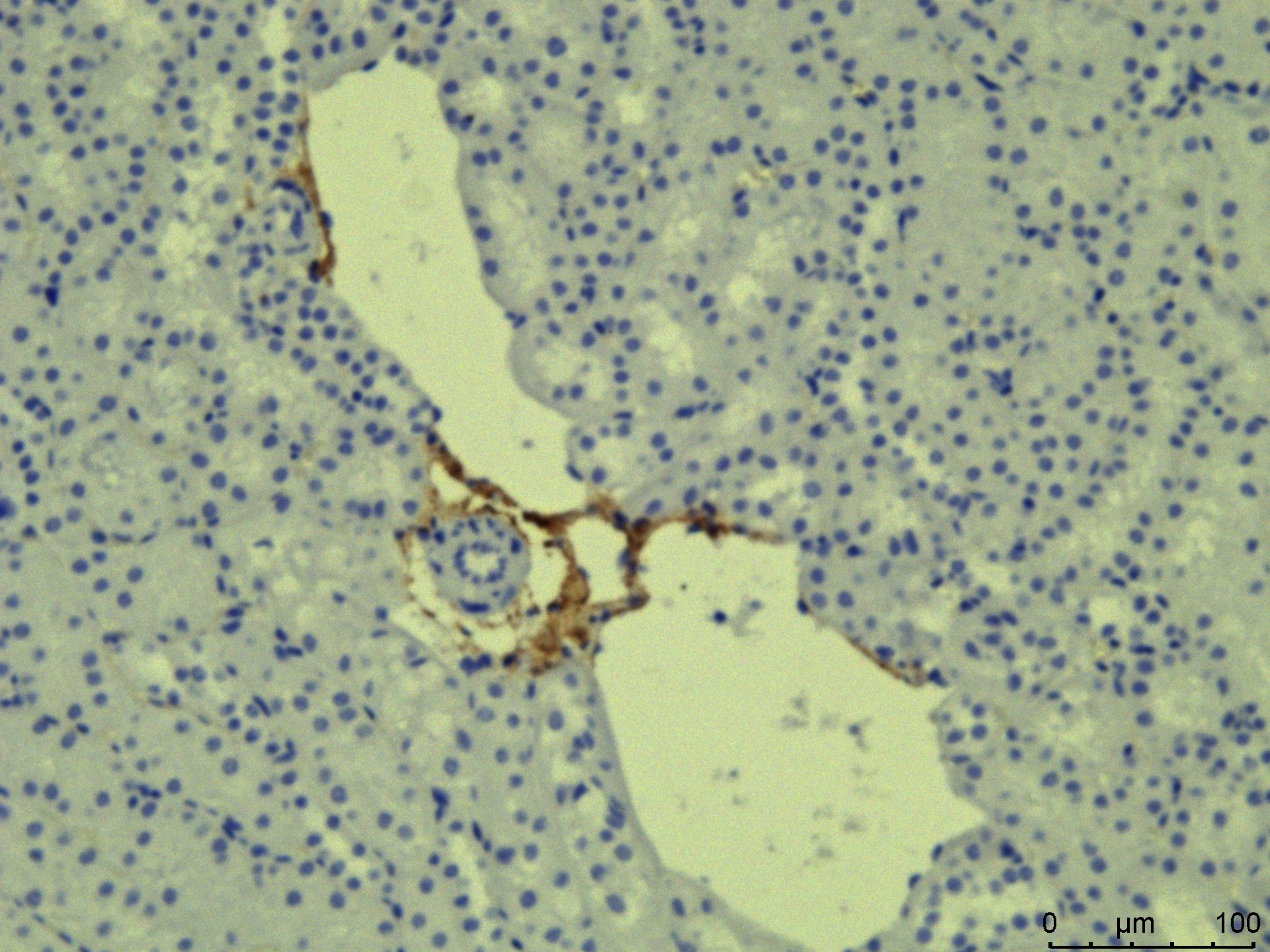

Supplement: Supplementary file 6 [file Data_Sheet_2.ZIP › fig1-1/IHC-COL-CTRL_Image008_ch00.jpg]

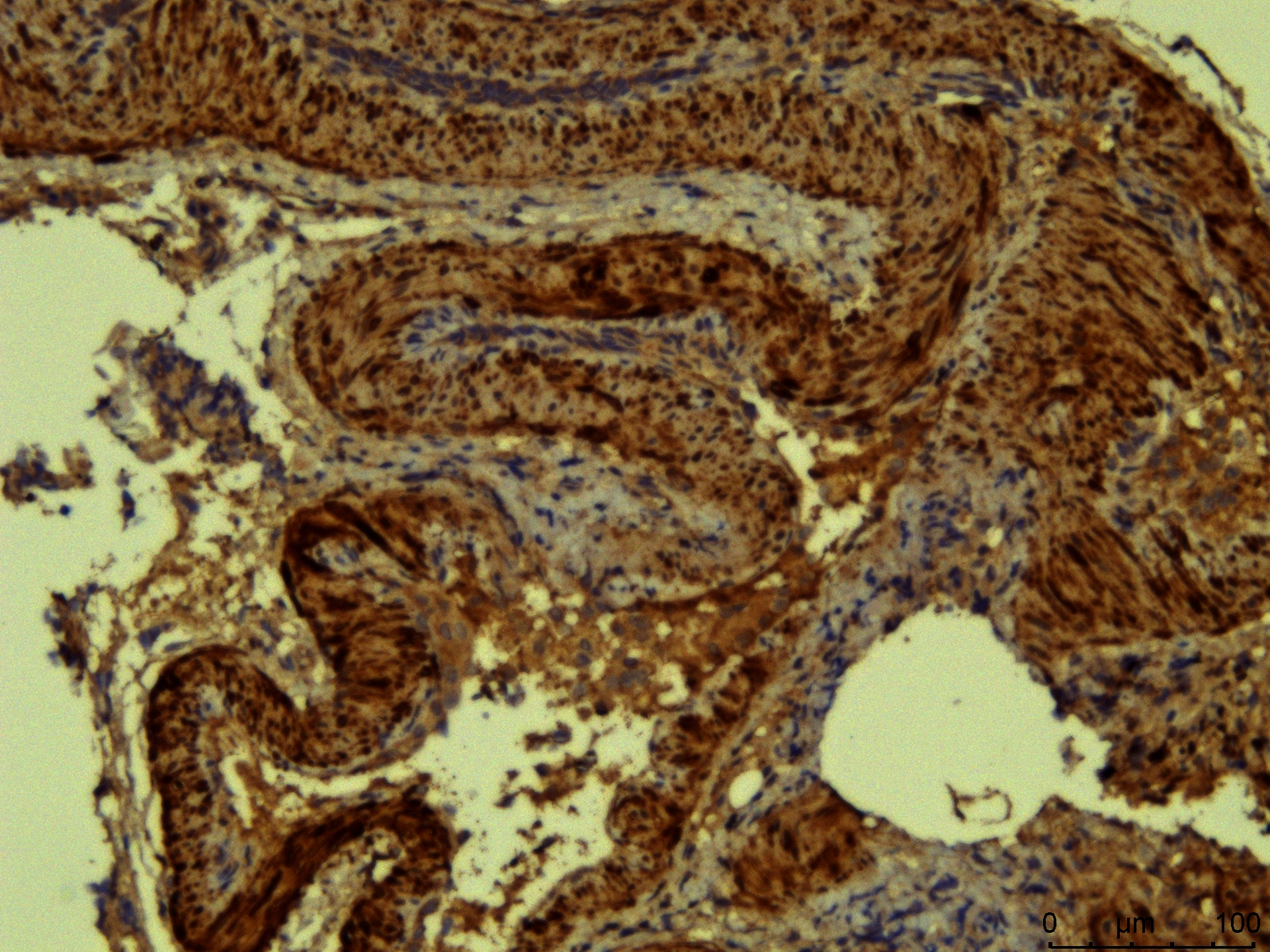

Supplement: Supplementary file 6 [file Data_Sheet_2.ZIP › fig1-1/IHC-FN-MODEL_Image010_ch00.jpg]

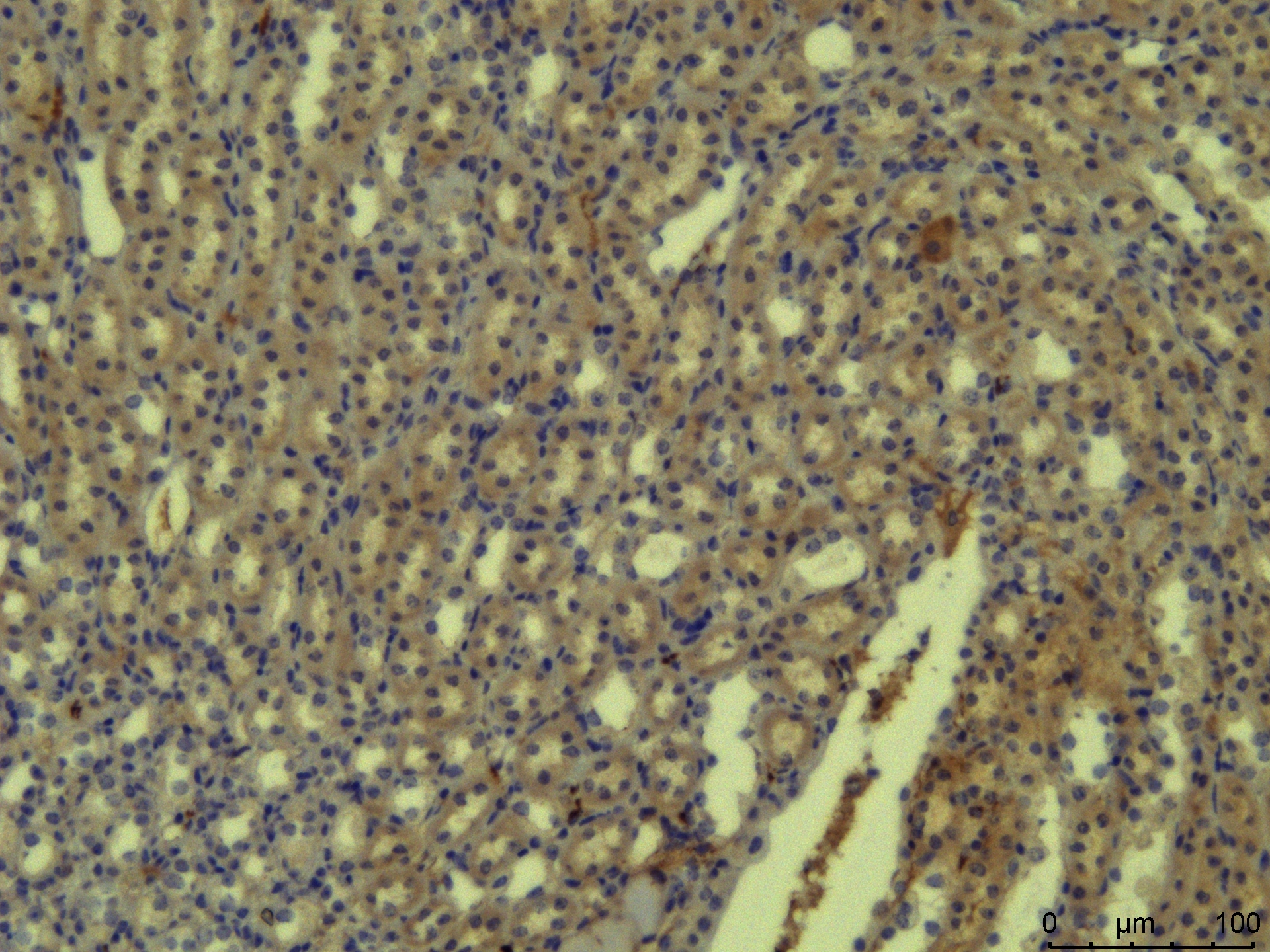

Supplement: Supplementary file 6 [file Data_Sheet_2.ZIP › fig1-1/IHC-FN-CTRL_Image009_ch00.jpg]

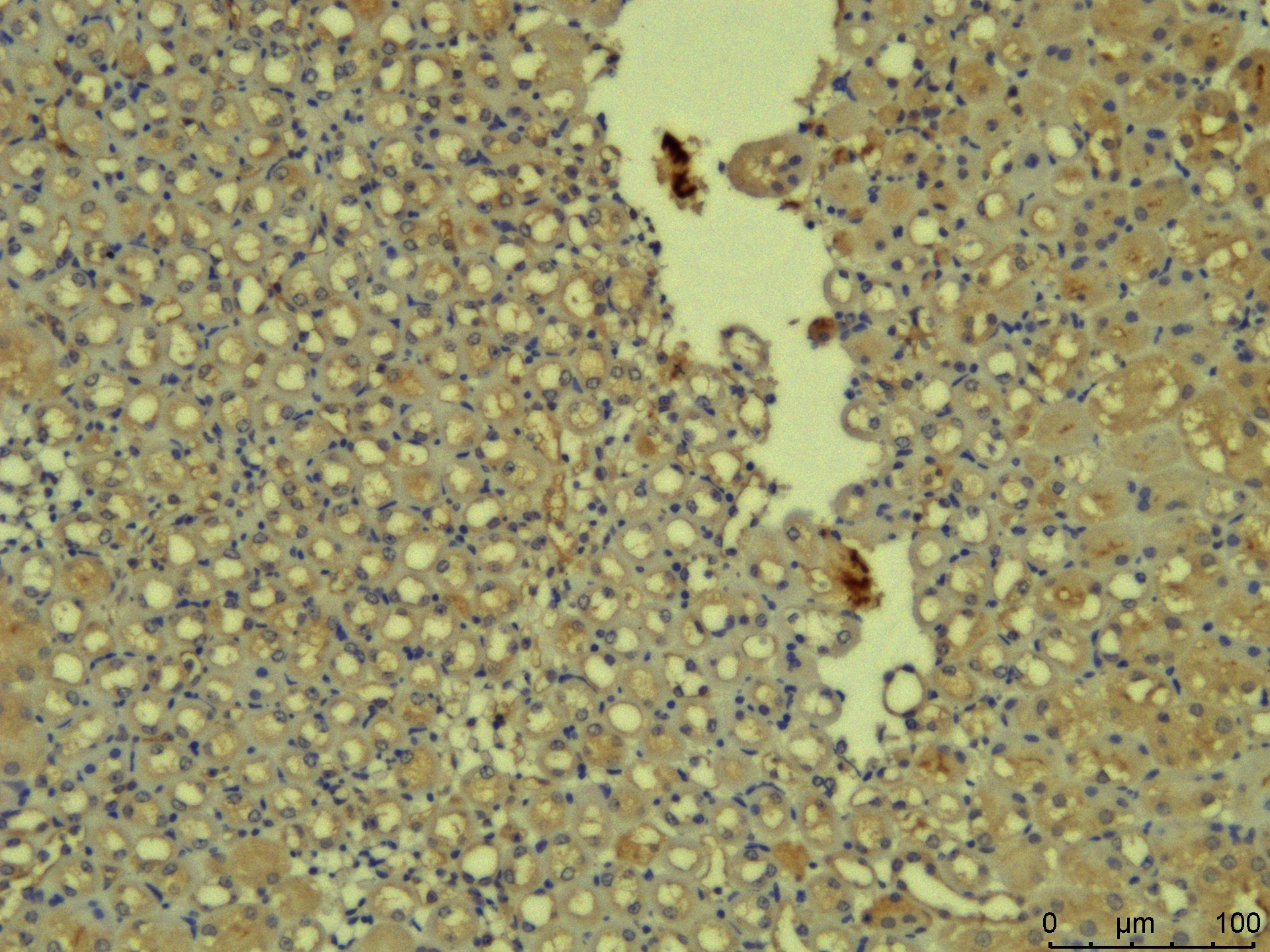

Supplement: Supplementary file 6 [file Data_Sheet_2.ZIP › fig1-1/IHC-JMJD1A-CTRL_Image003_ch00.jpg]

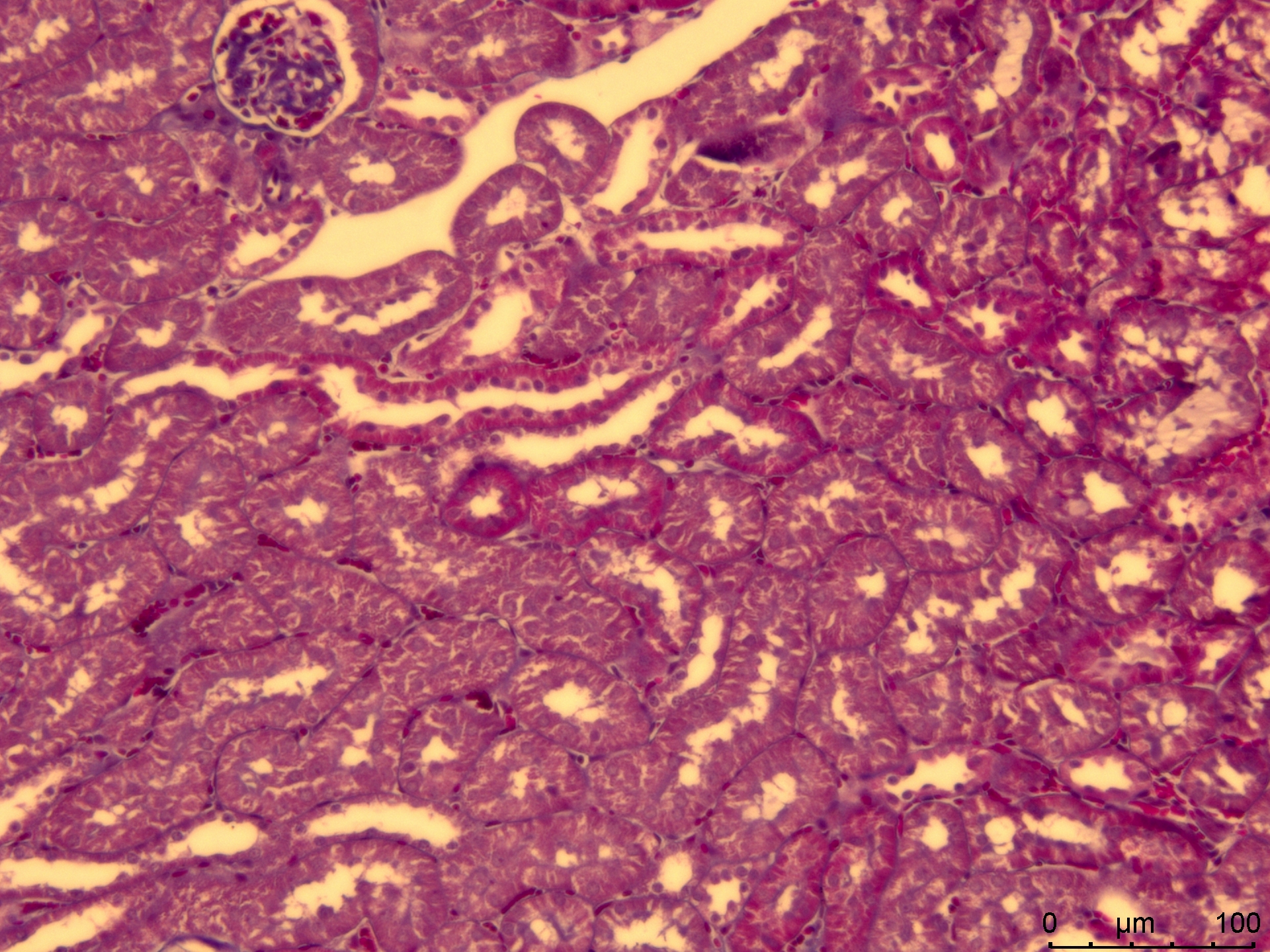

Supplement: Supplementary file 6 [file Data_Sheet_2.ZIP › fig1-1/CTRL-MASSION_Image010_ch00.jpg]

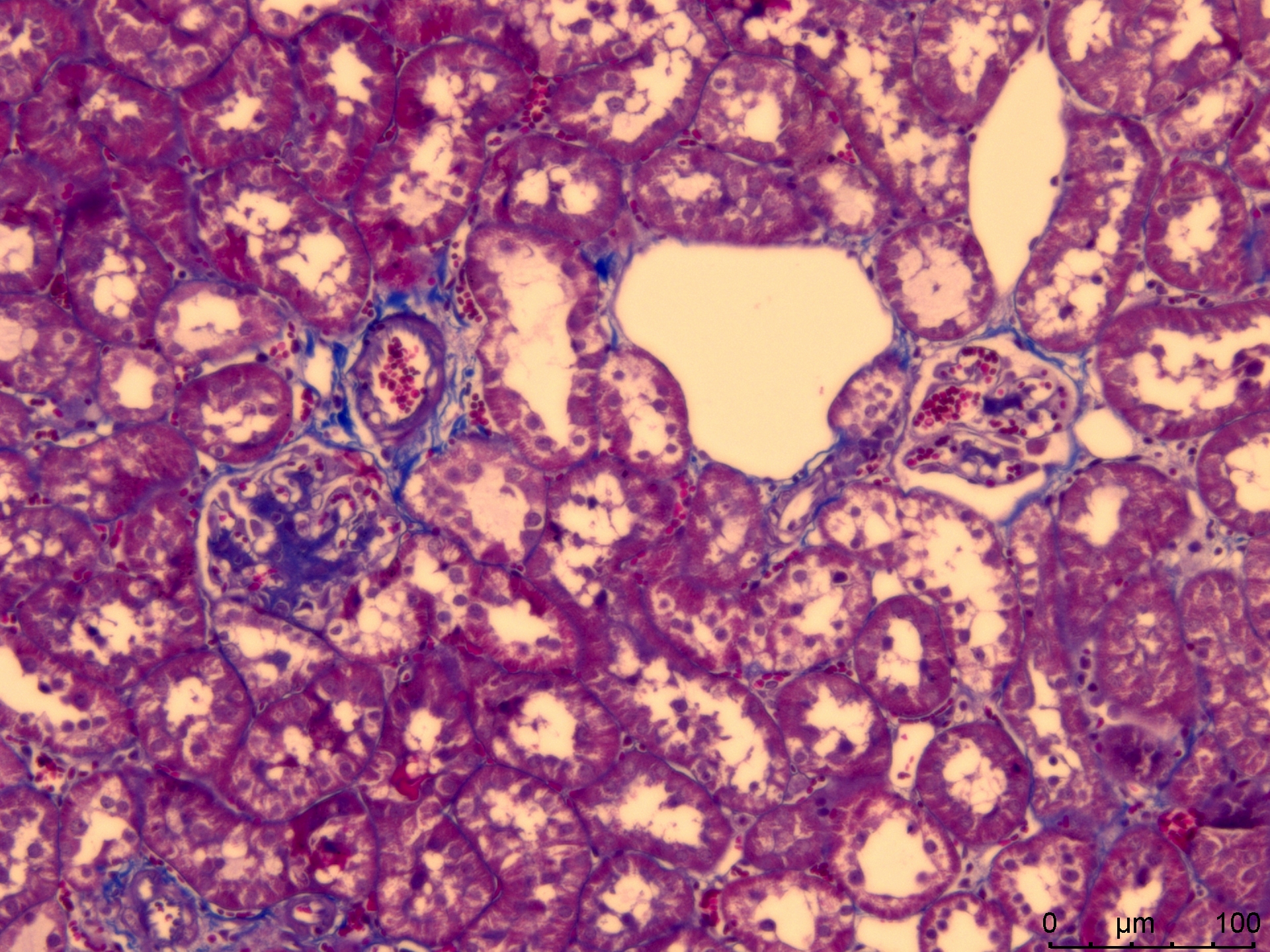

Supplement: Supplementary file 6 [file Data_Sheet_2.ZIP › fig1-1/model-MASSION_Image005_ch00.jpg]

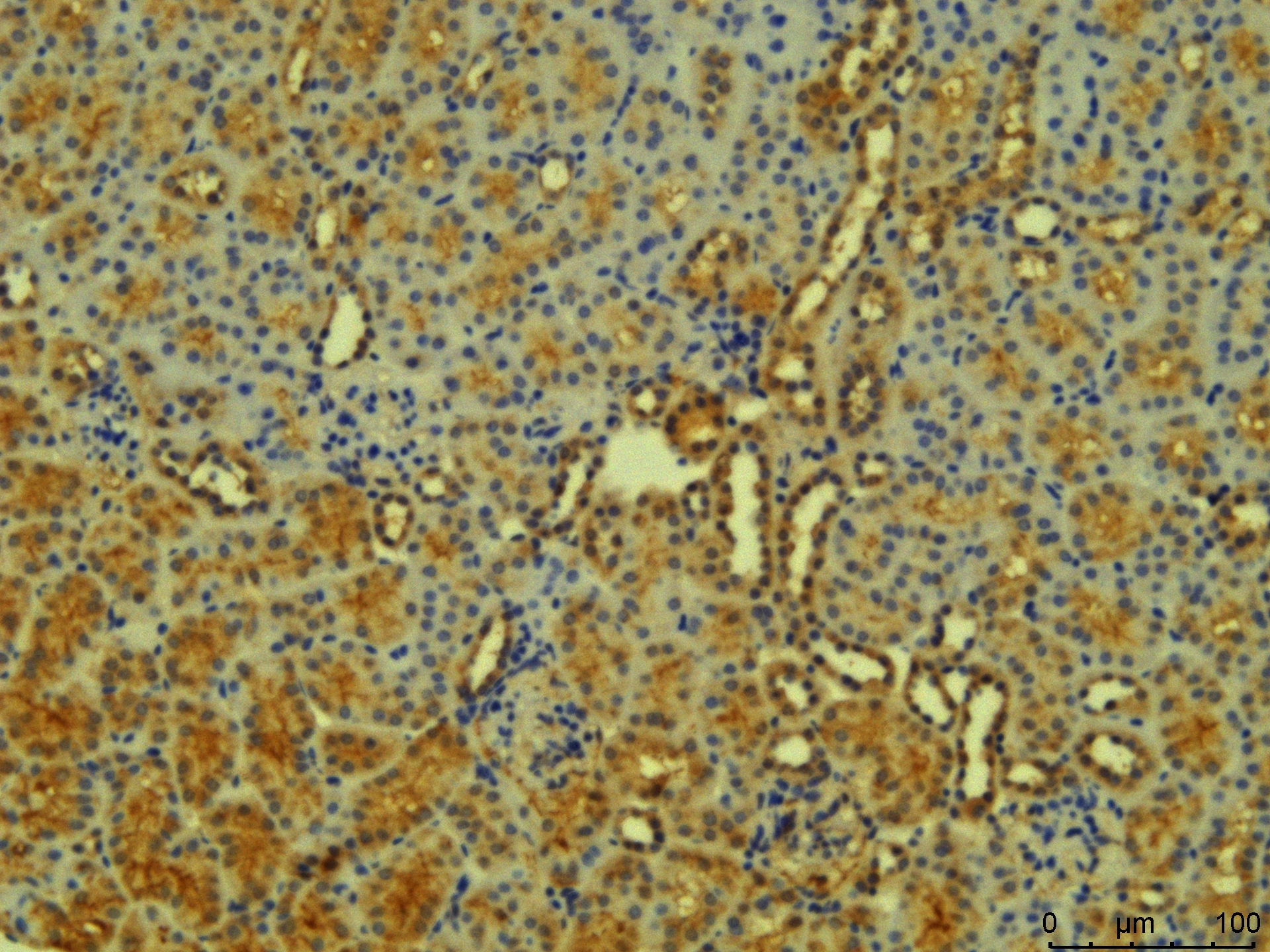

Supplement: Supplementary file 6 [file Data_Sheet_2.ZIP › fig1-1/IHC-JMJD1A-MODEL_Image003_ch00.jpg]

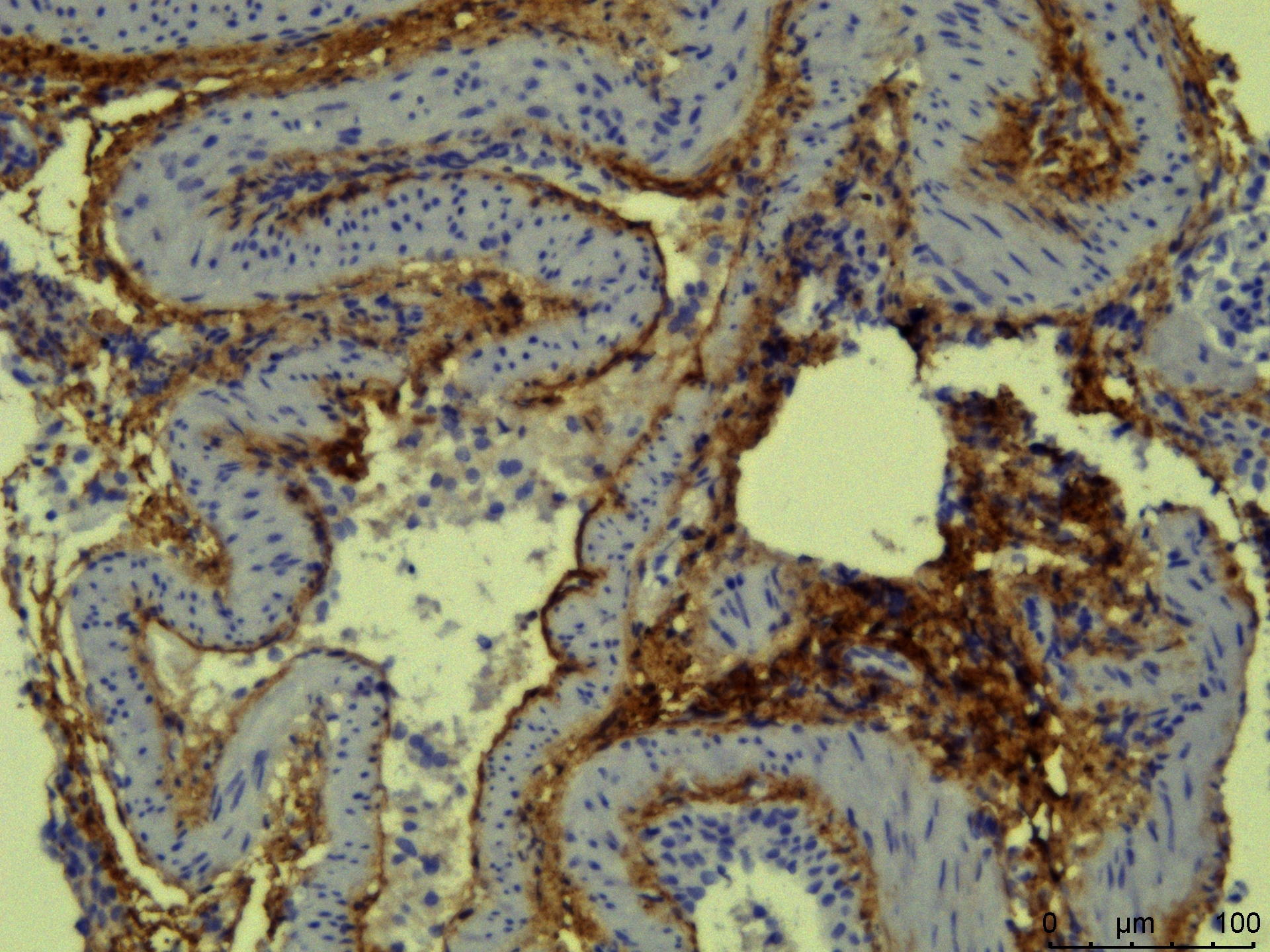

Supplement: Supplementary file 6 [file Data_Sheet_2.ZIP › fig1-1/IHC-COL1-MODEL_Image001_ch00.jpg]

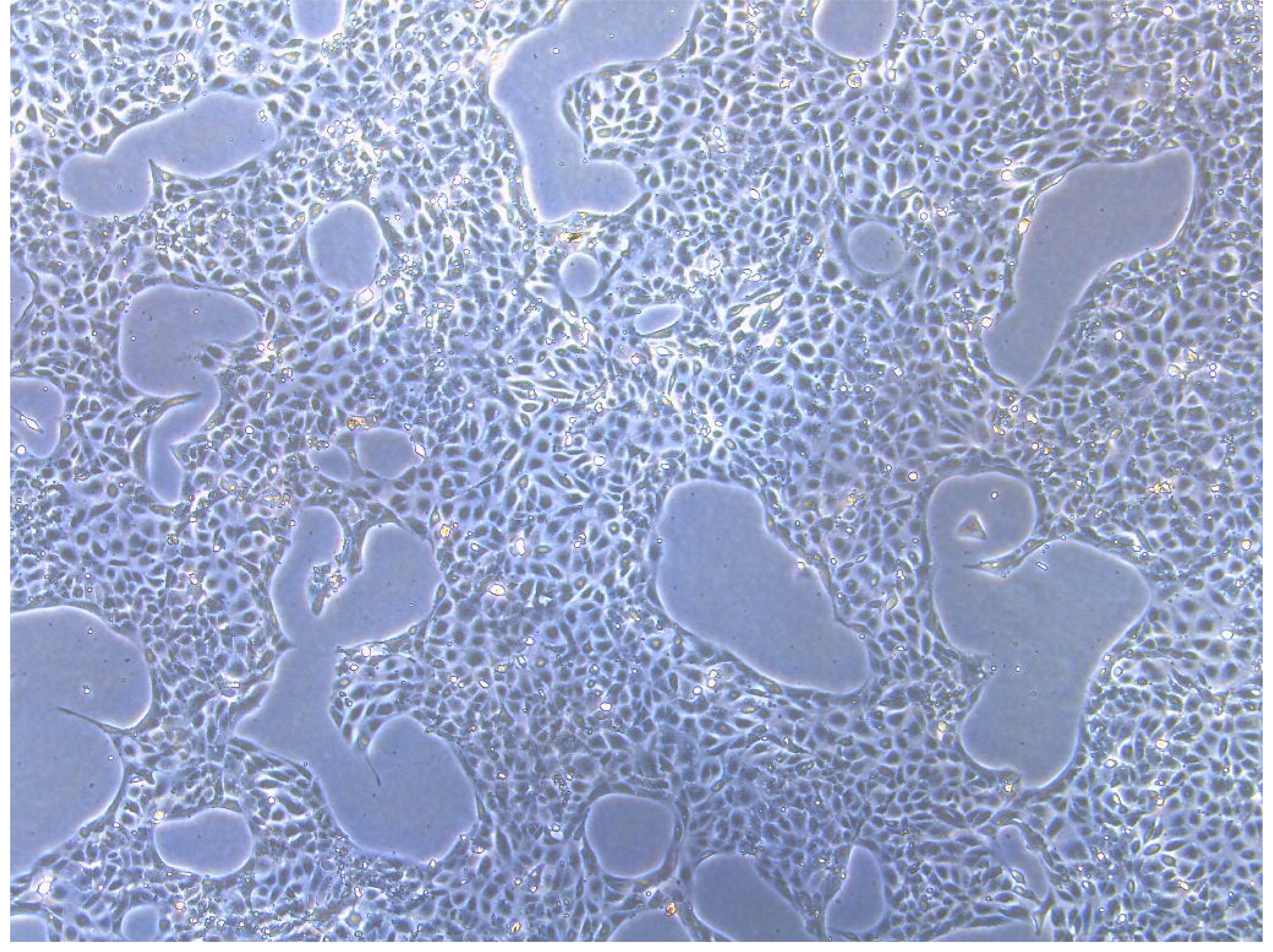

Supplement: Supplementary file 7 [file Data_Sheet_3.ZIP › fig1-2/Ctrl.jpg]

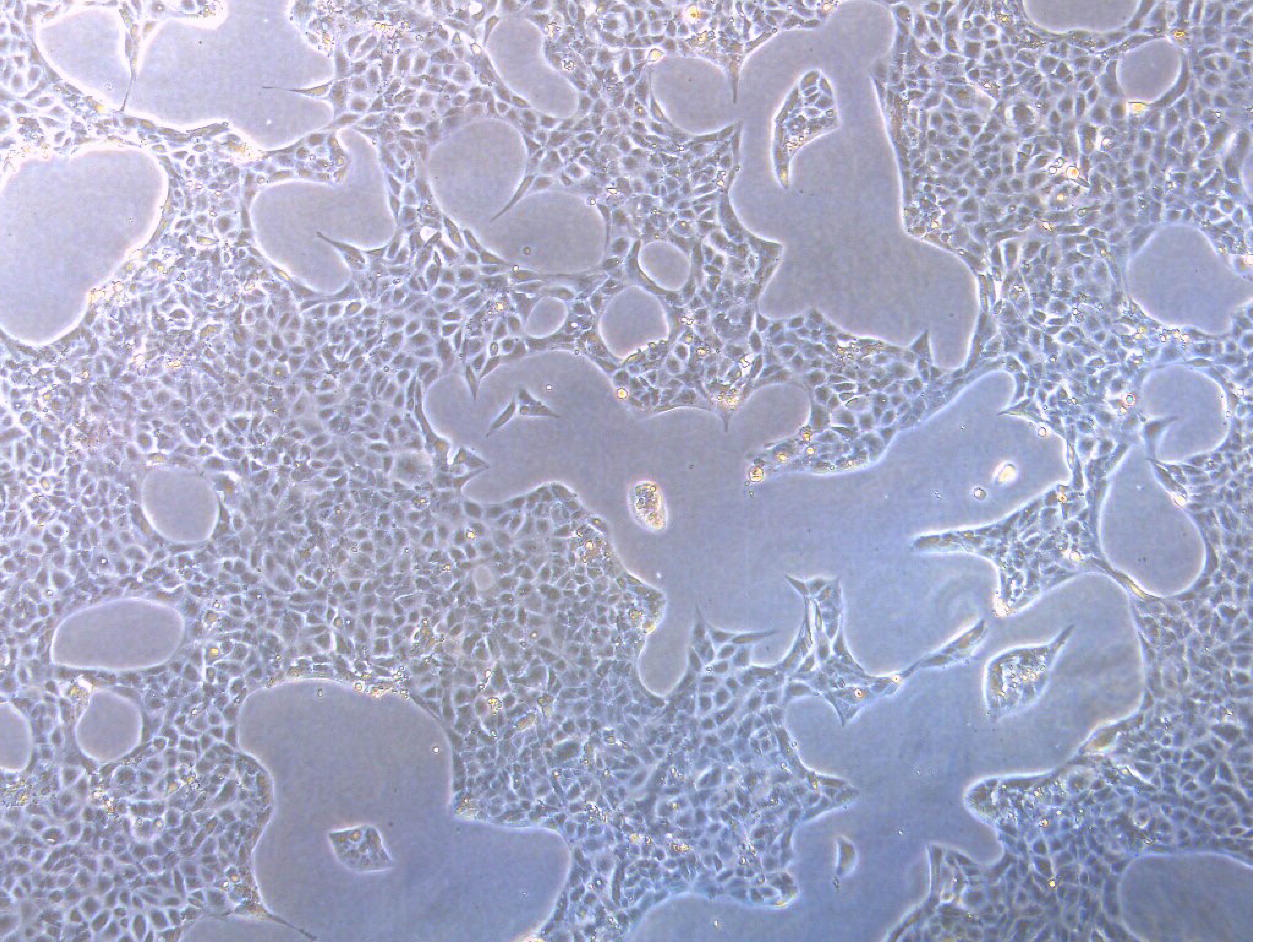

Supplement: Supplementary file 7 [file Data_Sheet_3.ZIP › fig1-2/48h.jpg]

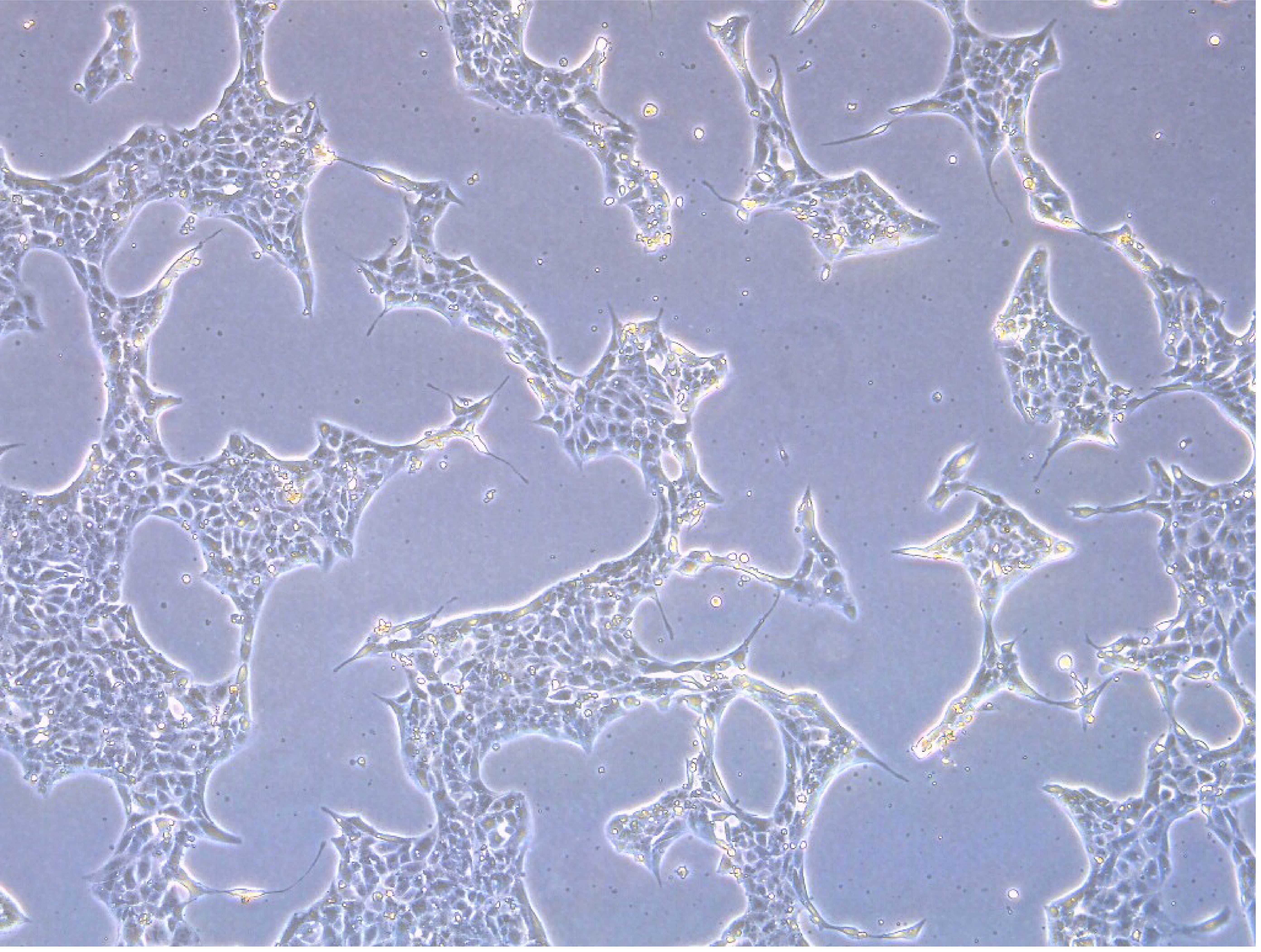

Supplement: Supplementary file 7 [file Data_Sheet_3.ZIP › fig1-2/72h.jpg]

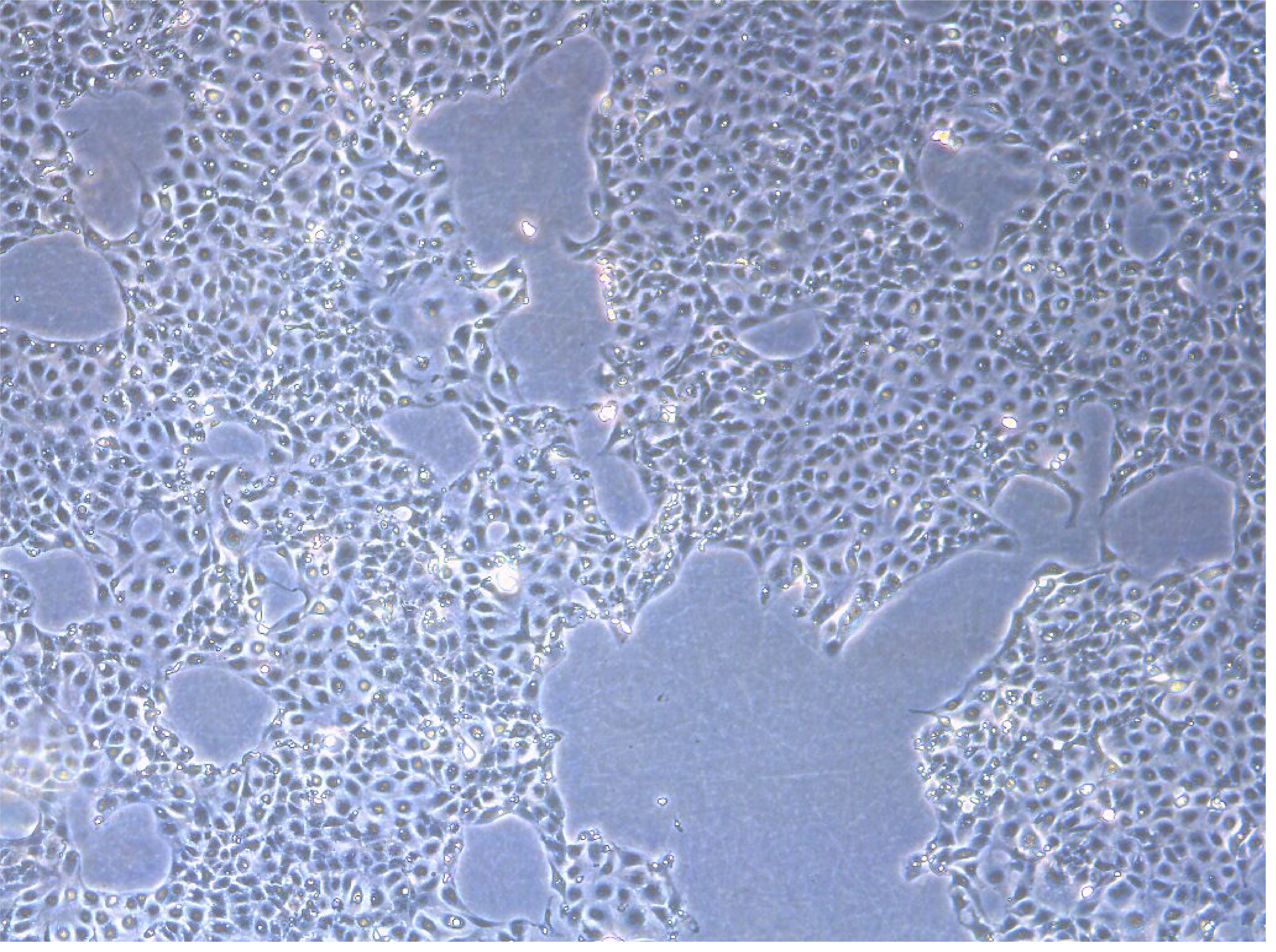

Supplement: Supplementary file 7 [file Data_Sheet_3.ZIP › fig1-2/12h.jpg]

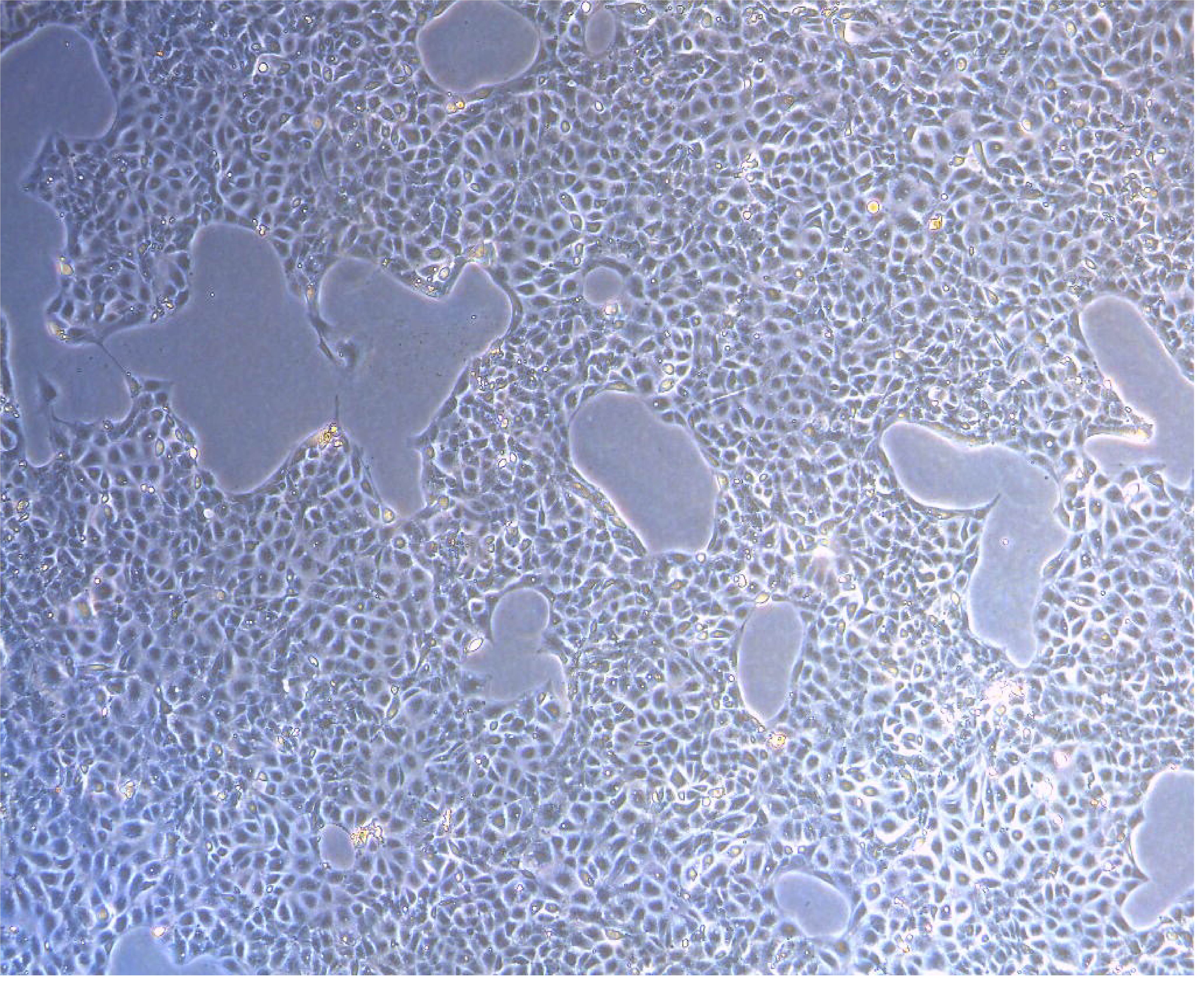

Supplement: Supplementary file 7 [file Data_Sheet_3.ZIP › fig1-2/24h.jpg]

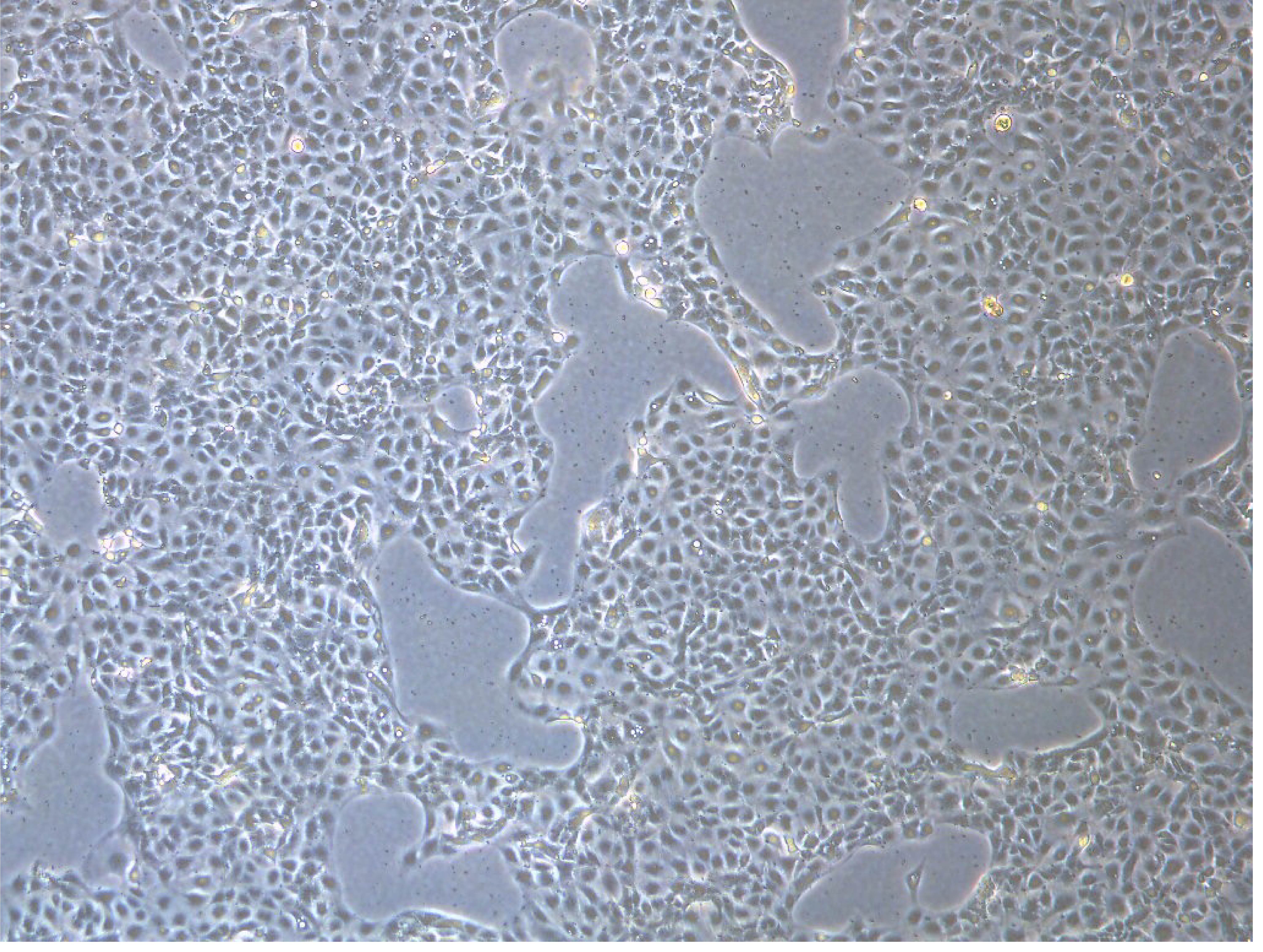

Supplement: Supplementary file 7 [file Data_Sheet_3.ZIP › fig1-2/6h.jpg]

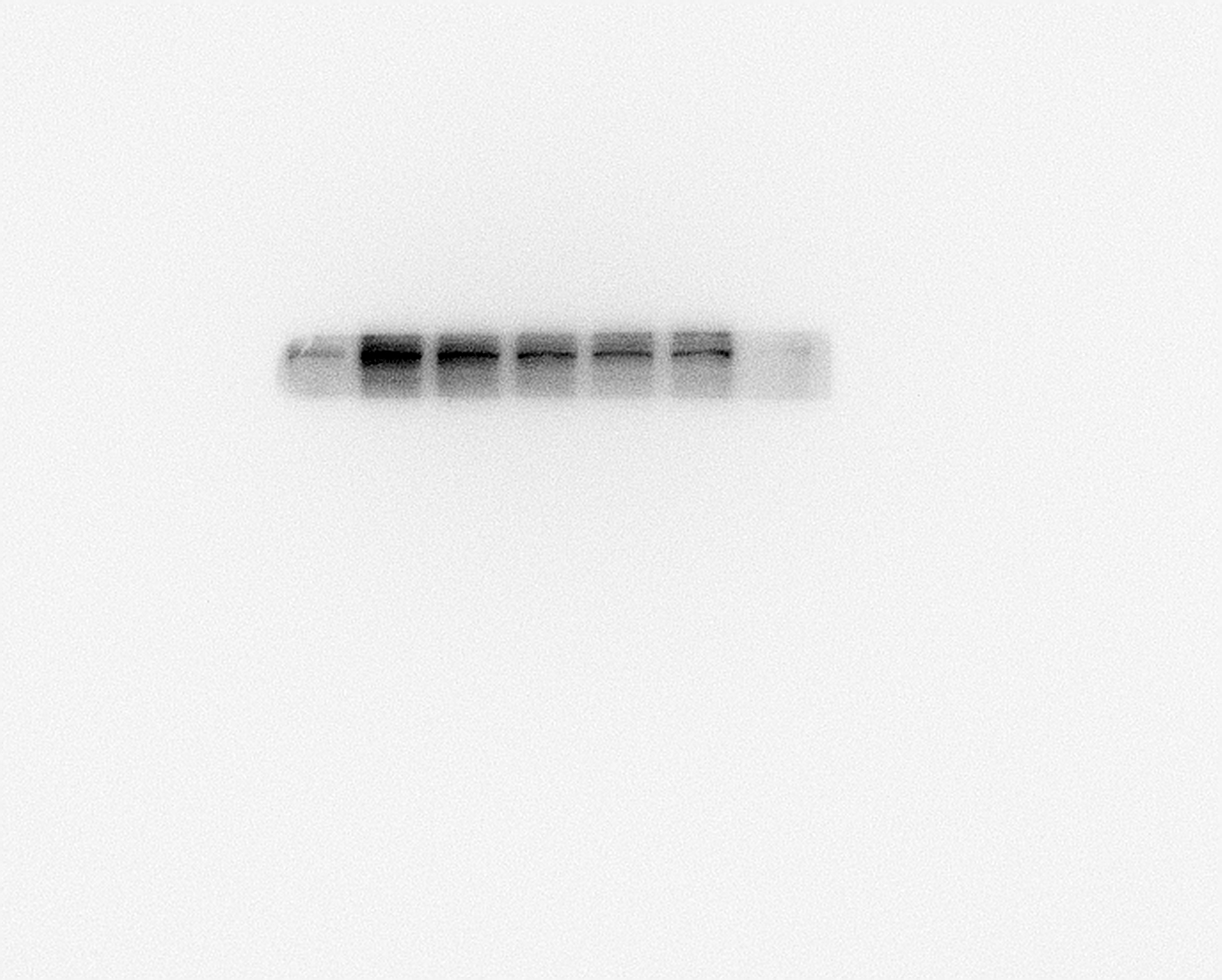

Supplement: Supplementary file 8 [file Data_Sheet_4.ZIP › Fig.2/E-cad.jpg]

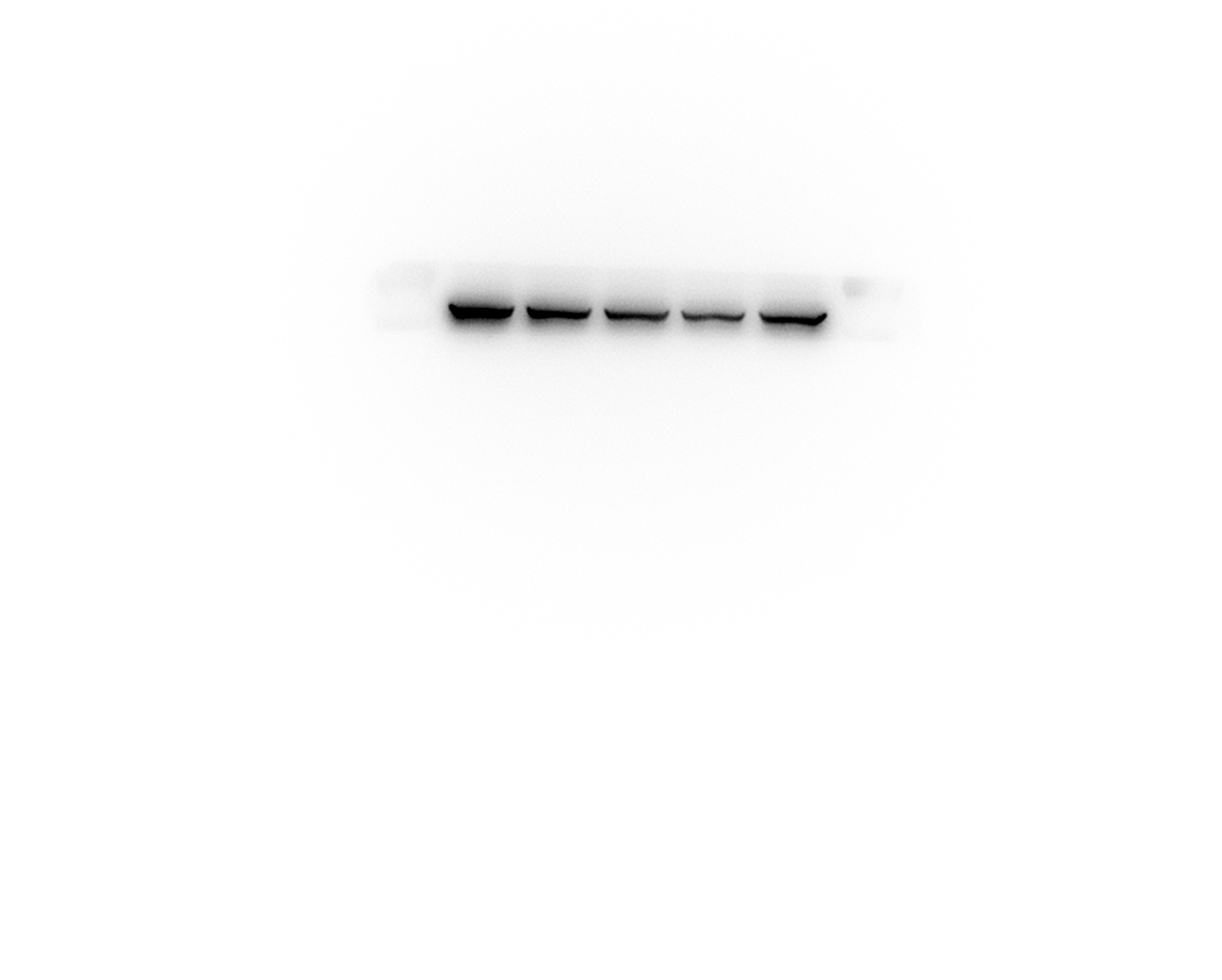

Supplement: Supplementary file 8 [file Data_Sheet_4.ZIP › Fig.2/vim.jpg]

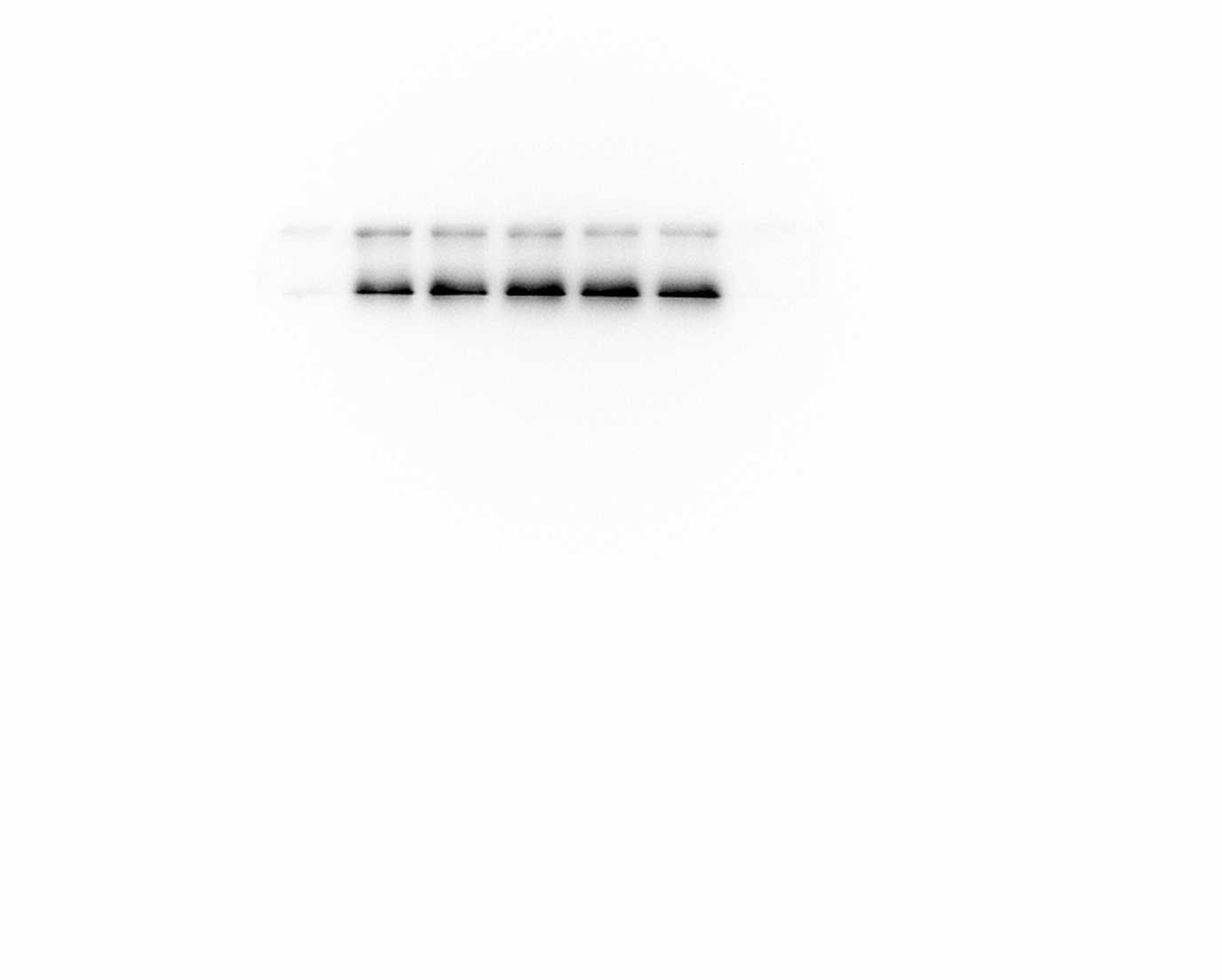

Supplement: Supplementary file 8 [file Data_Sheet_4.ZIP › Fig.2/BCL-2.jpg]

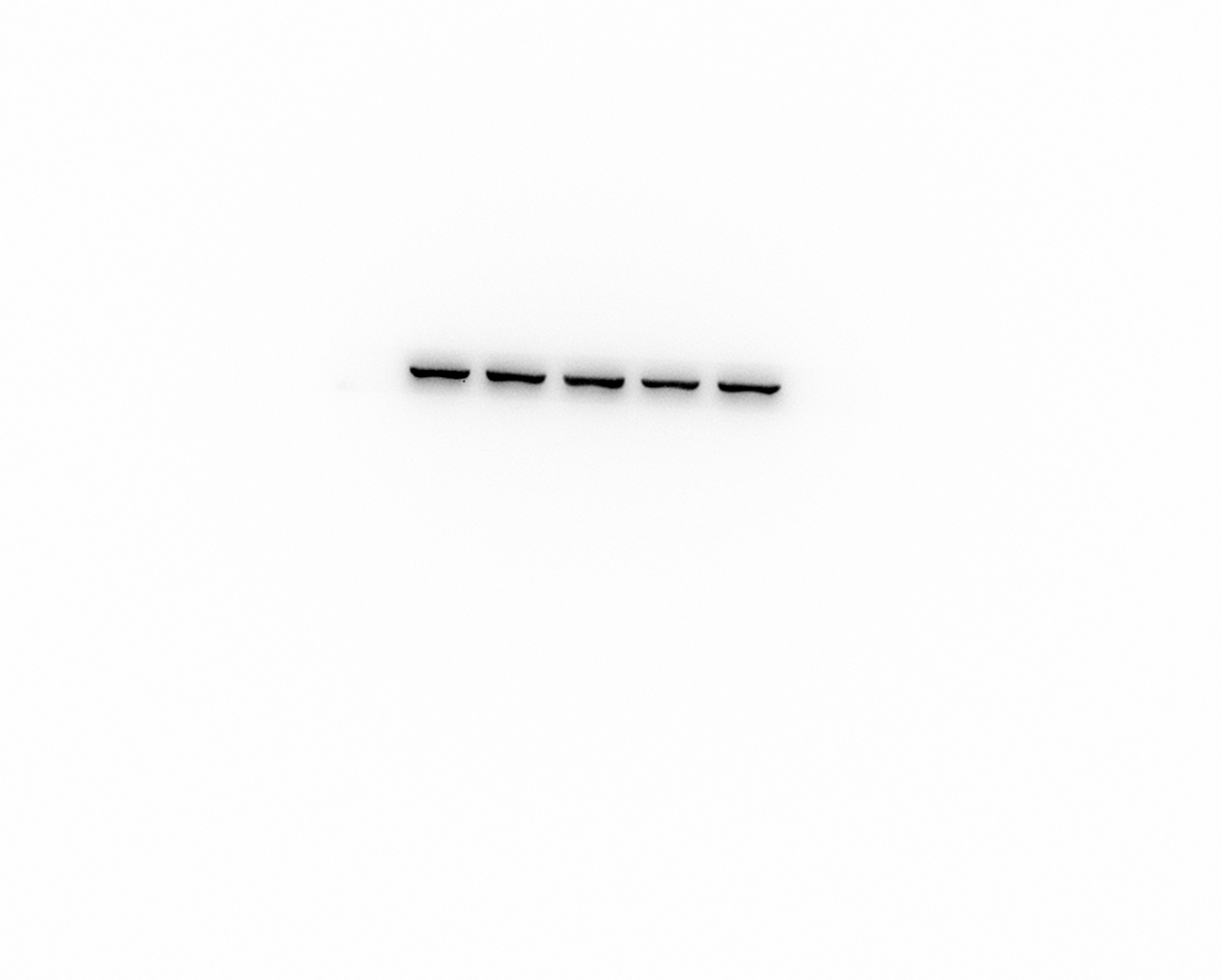

Supplement: Supplementary file 8 [file Data_Sheet_4.ZIP › Fig.2/GAPDH.jpg]

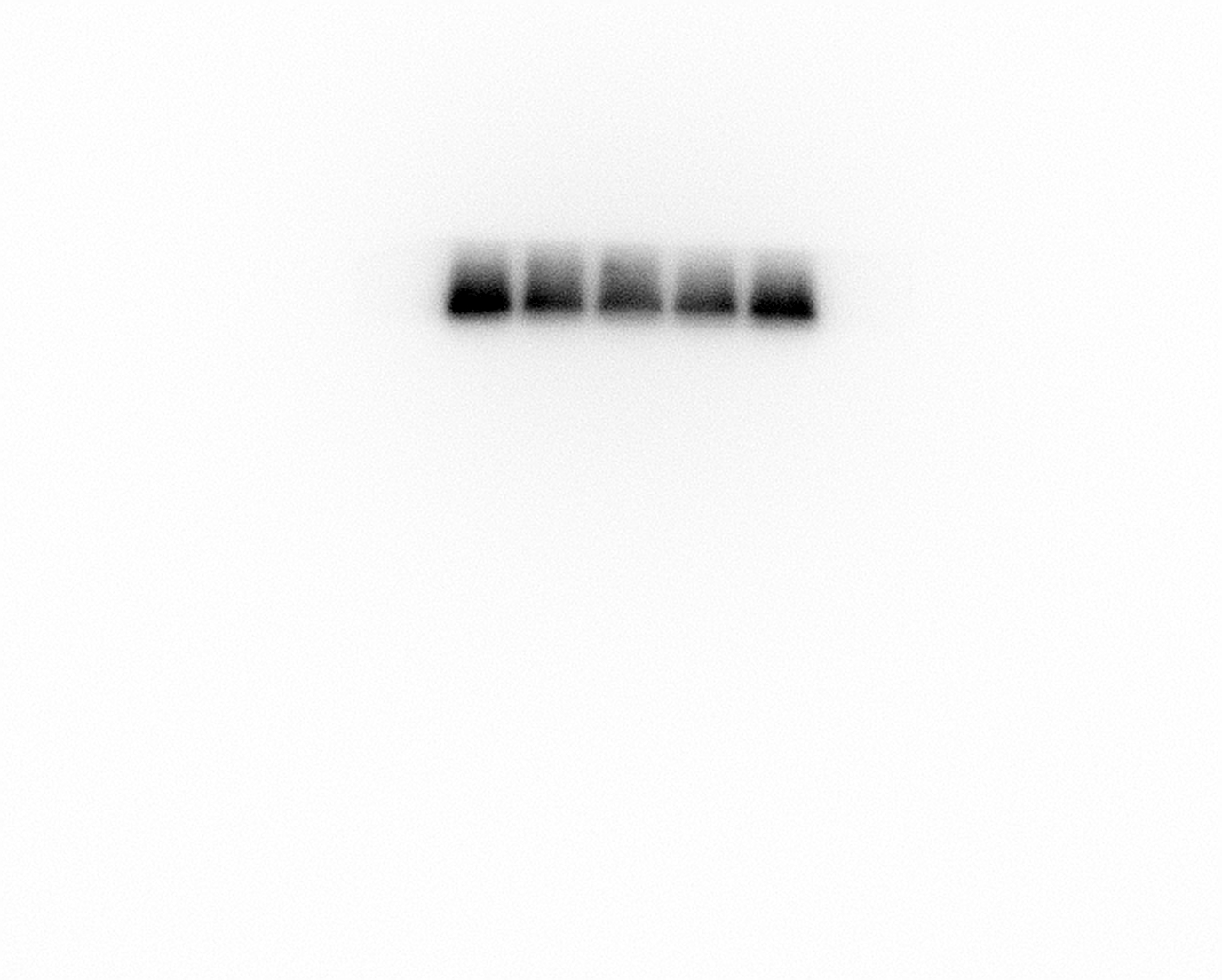

Supplement: Supplementary file 8 [file Data_Sheet_4.ZIP › Fig.2/BAX-0.3S.jpg]

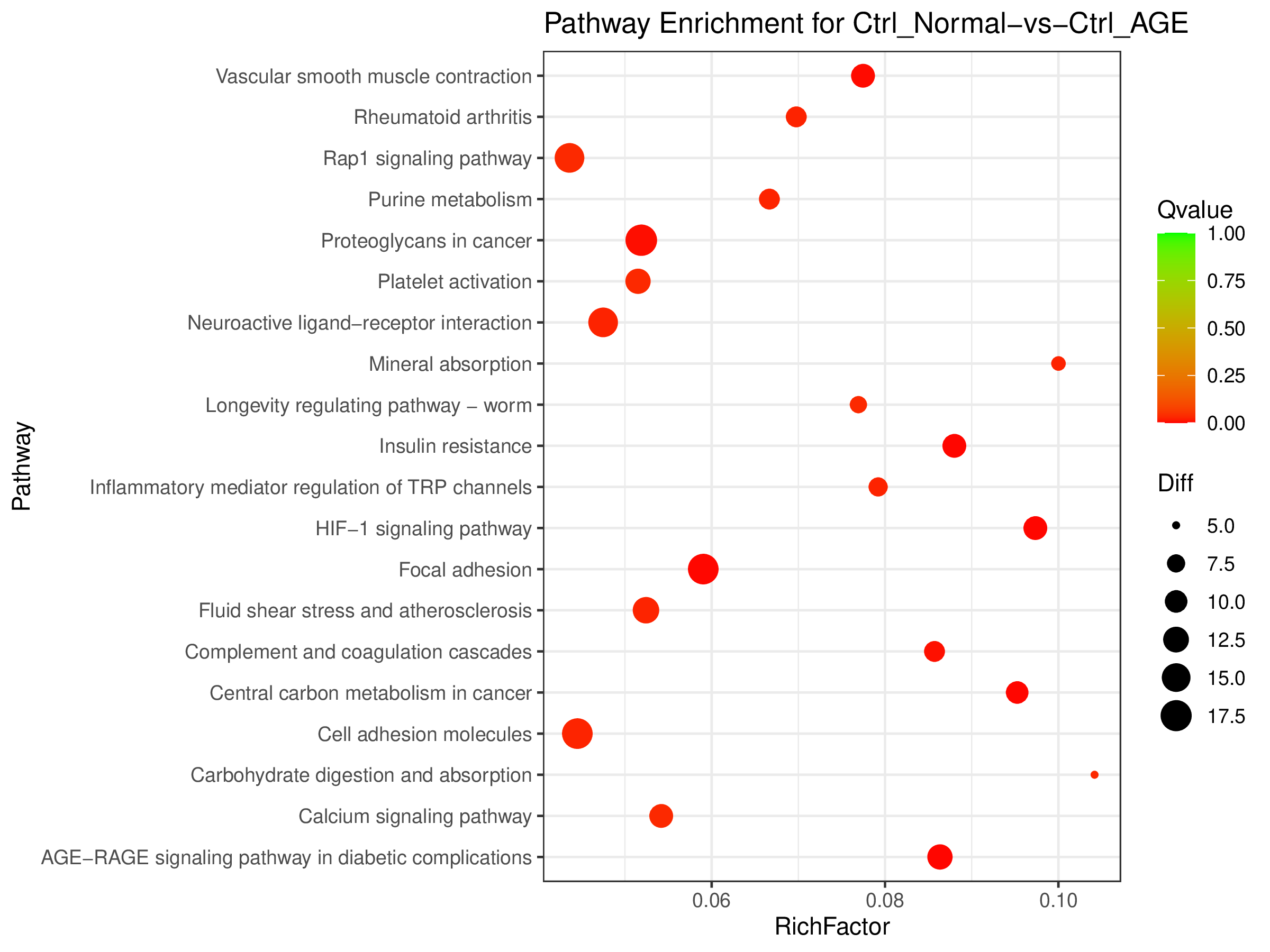

Supplement: Supplementary file 9 [file Data_Sheet_5.ZIP › Fig.3/Ctrl_Normal-vs-Ctrl_AGE.Pathenrich.png]

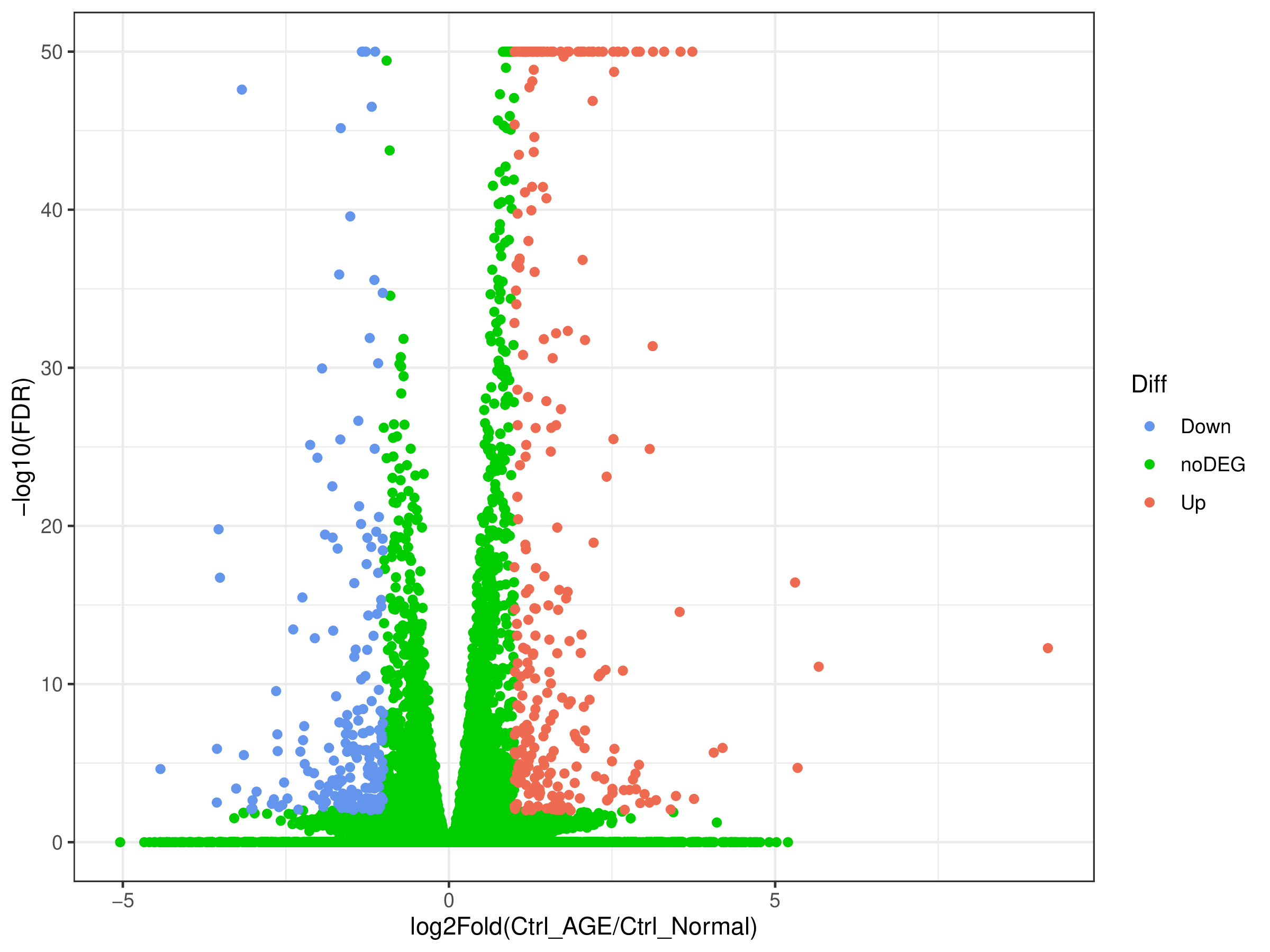

Supplement: Supplementary file 9 [file Data_Sheet_5.ZIP › Fig.3/Ctrl_Normal-vs-Ctrl_AGE.Volcano.png]

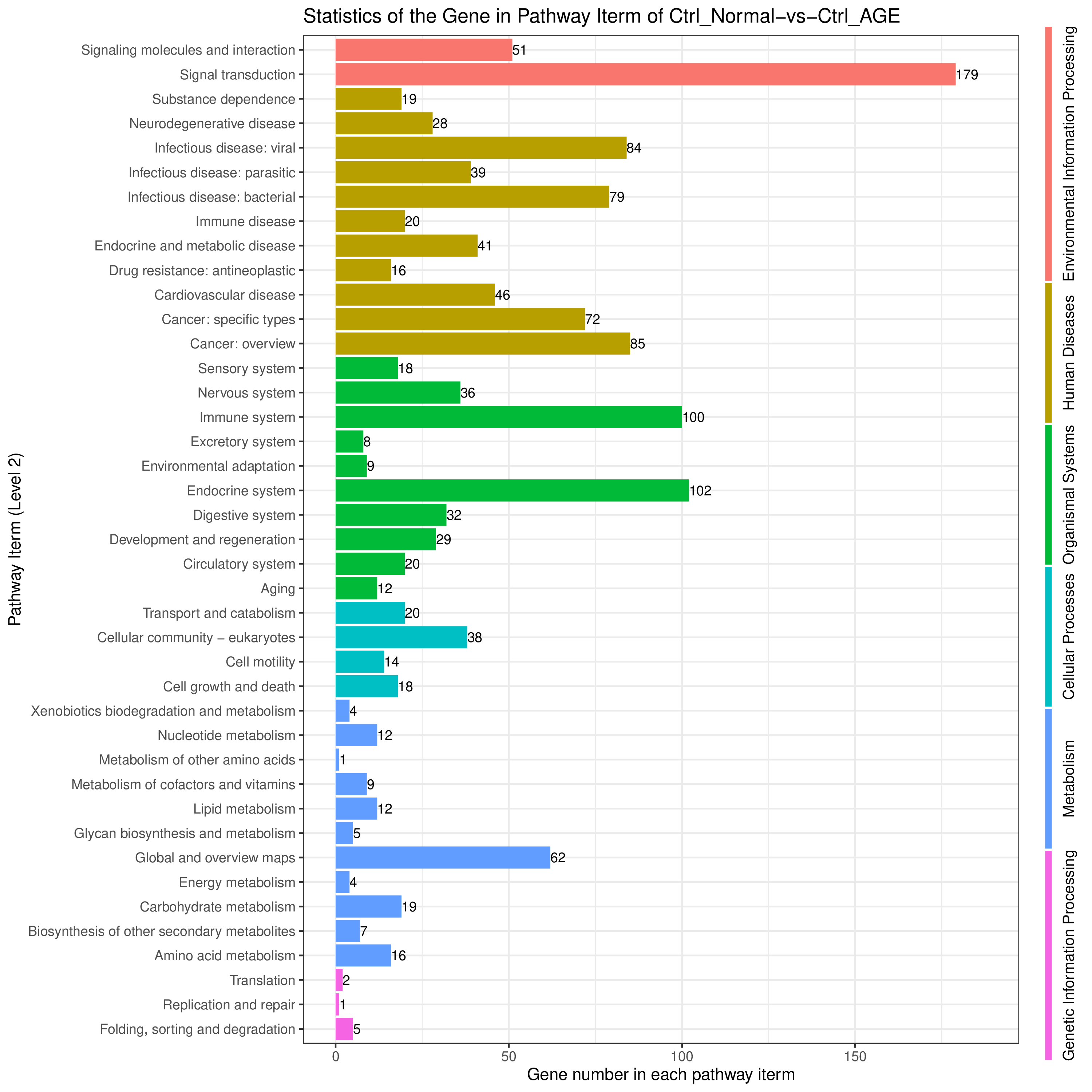

Supplement: Supplementary file 9 [file Data_Sheet_5.ZIP › Fig.3/Ctrl_Normal-vs-Ctrl_AGE.path_class.png]

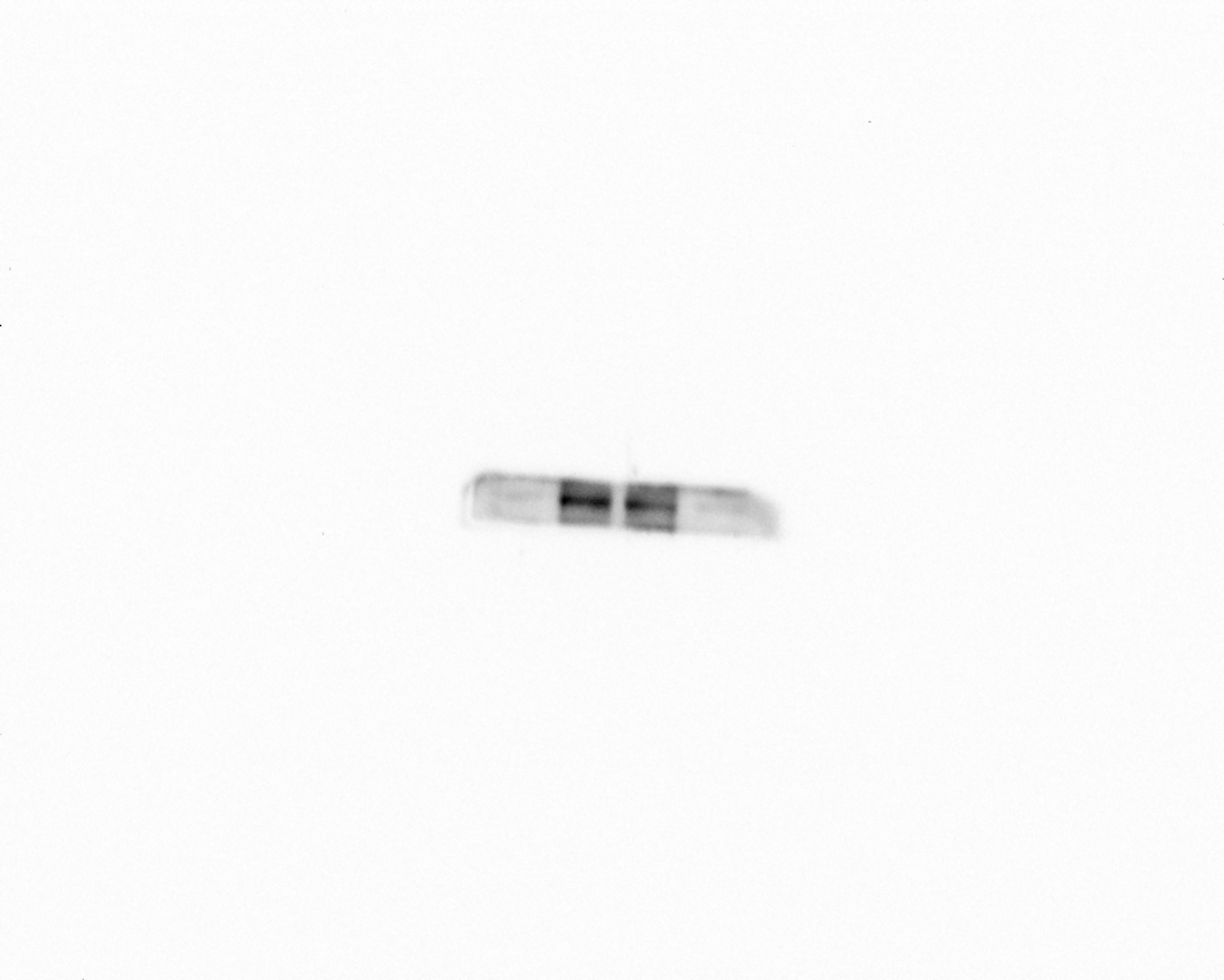

Supplement: Supplementary file 10 [file Data_Sheet_6.ZIP › Fig.4/E-cad.jpg]

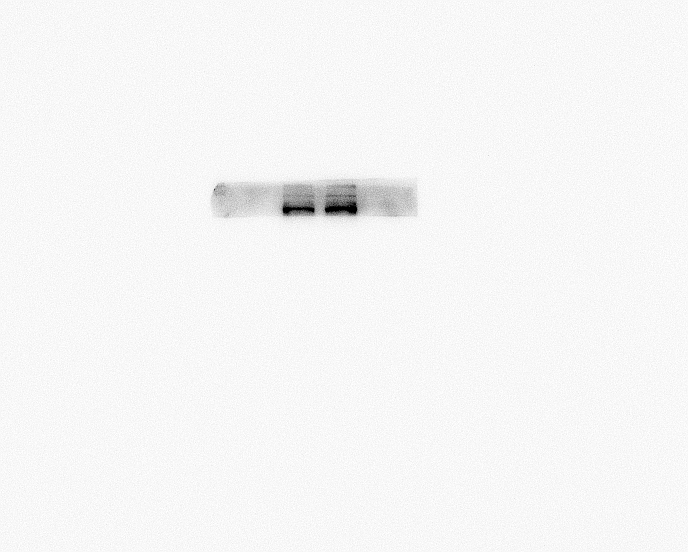

Supplement: Supplementary file 10 [file Data_Sheet_6.ZIP › Fig.4/jmjd1a-1.jpg]

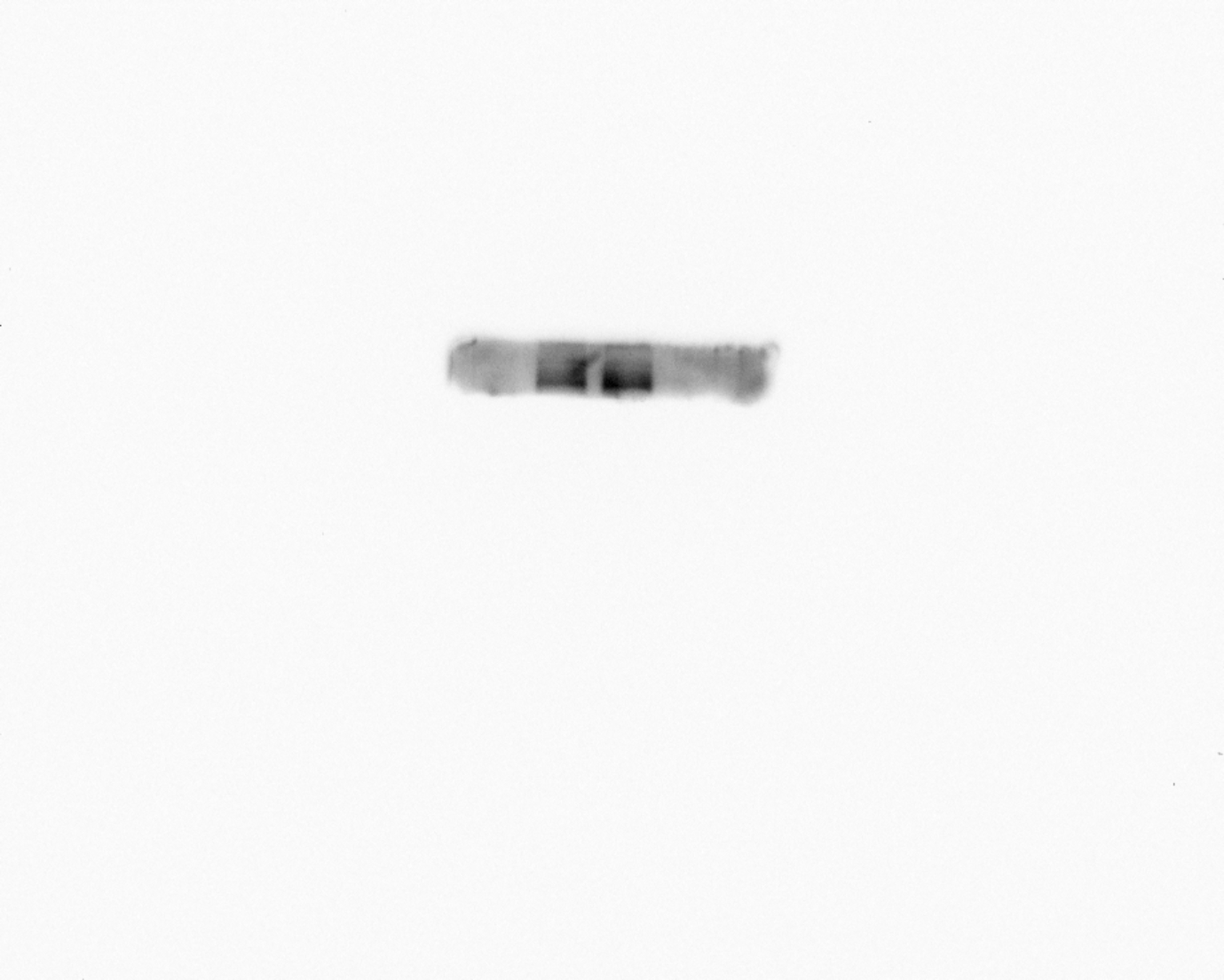

Supplement: Supplementary file 10 [file Data_Sheet_6.ZIP › Fig.4/Collagen1.jpg]

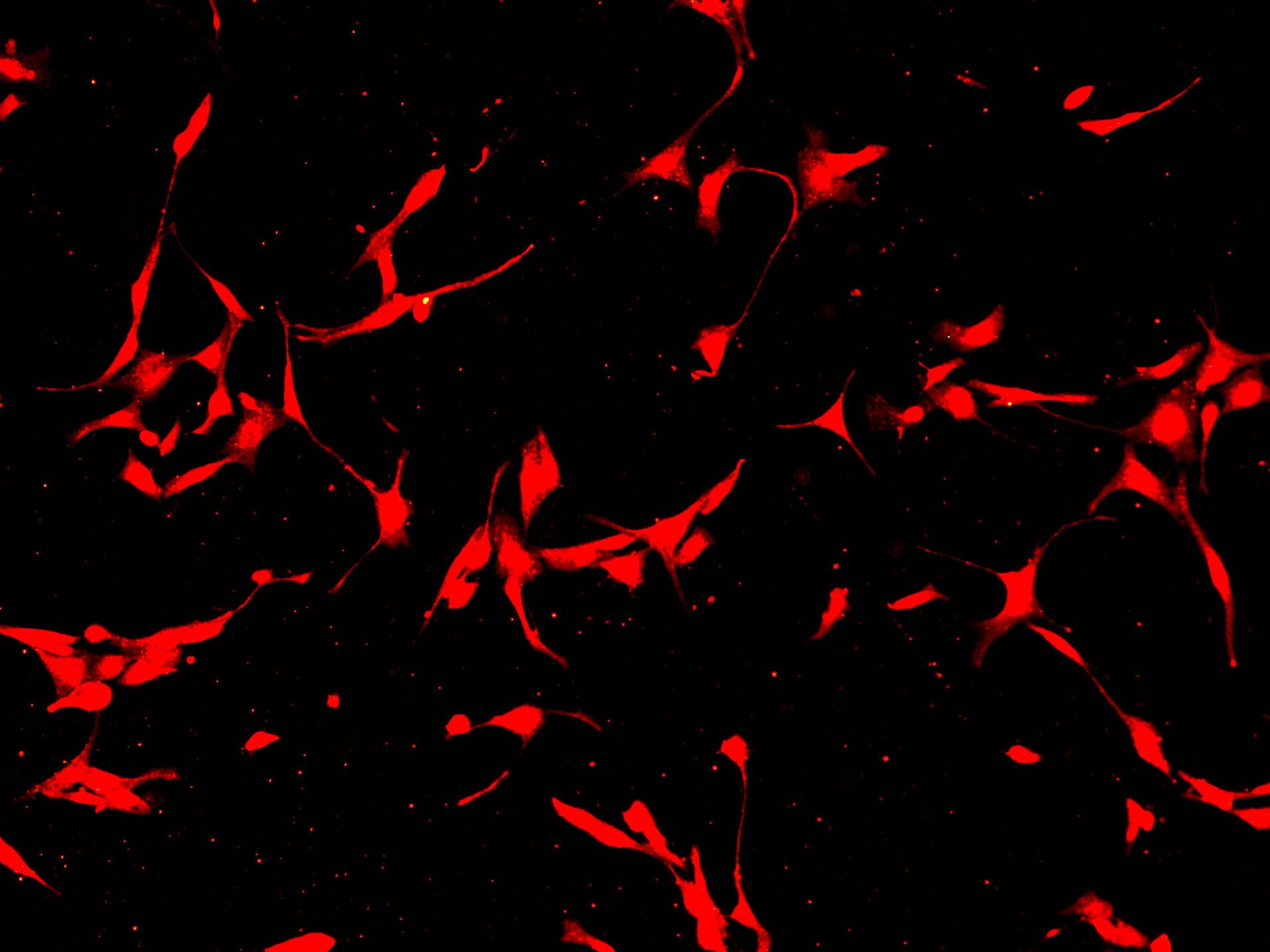

Supplement: Supplementary file 10 [file Data_Sheet_6.ZIP › Fig.4/AGEs-a-SMA.tif]

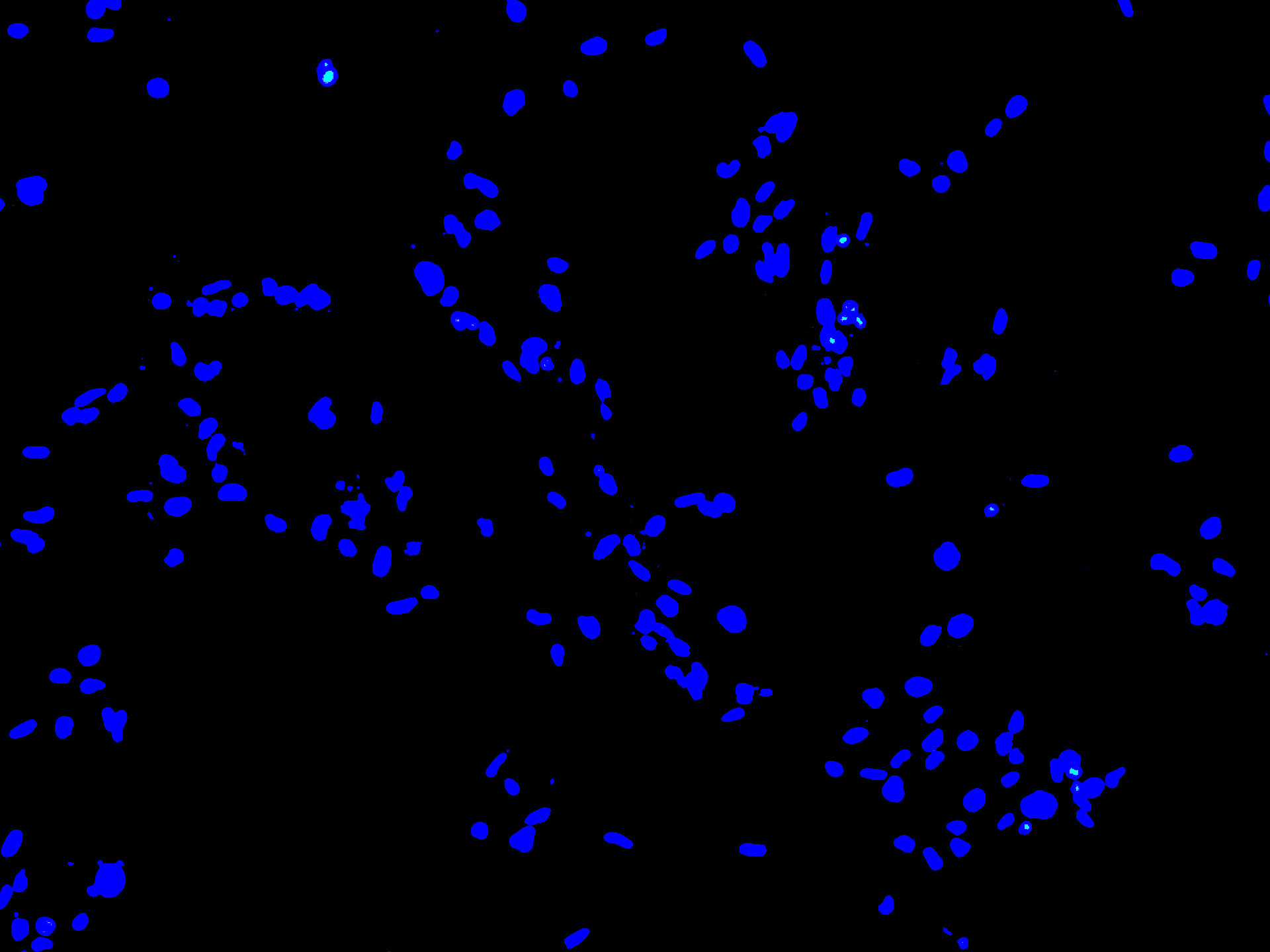

Supplement: Supplementary file 10 [file Data_Sheet_6.ZIP › Fig.4/AGEs-VIM-DAPI.tif]

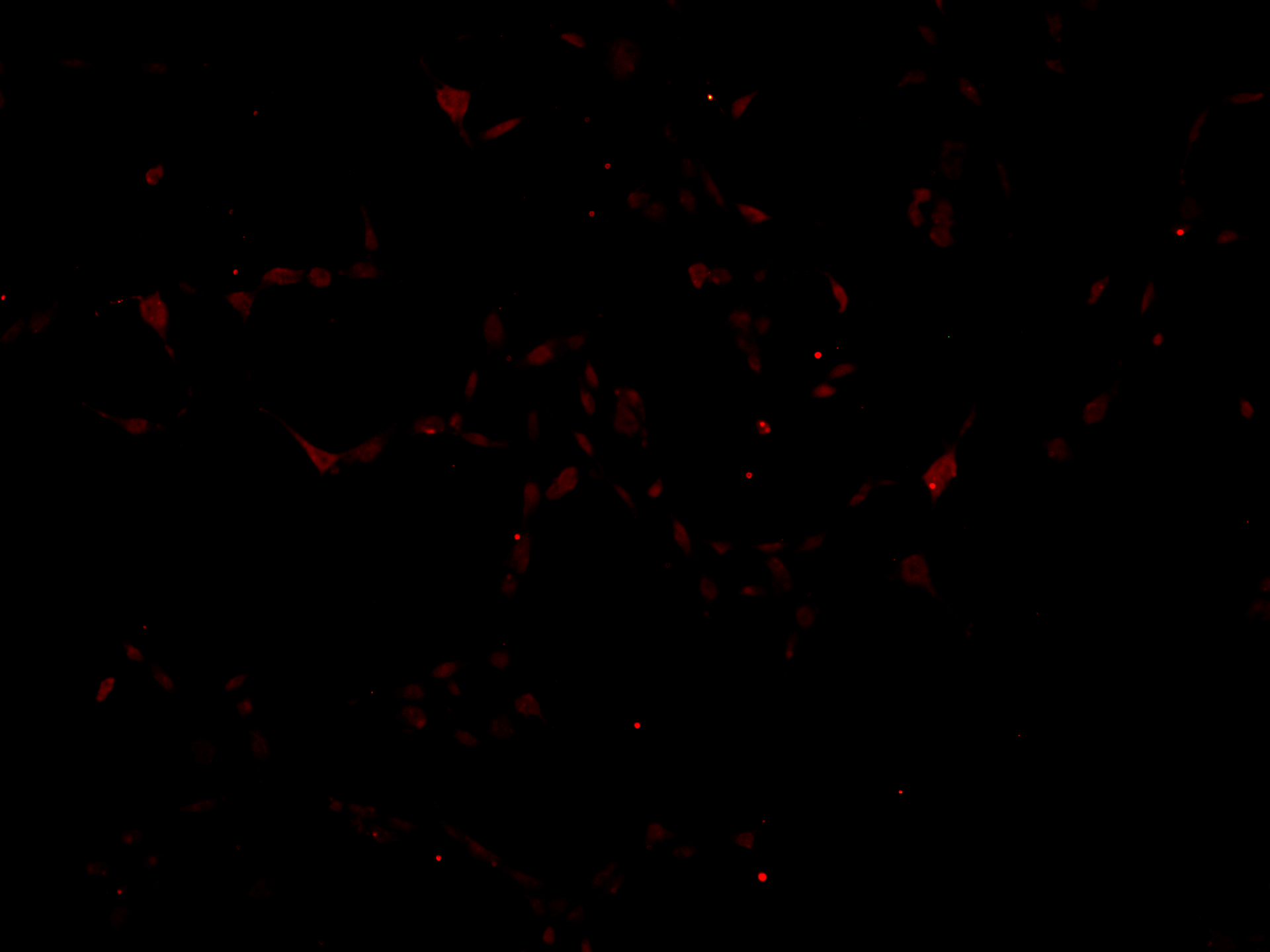

Supplement: Supplementary file 10 [file Data_Sheet_6.ZIP › Fig.4/CTRL-a-SMA.tif]

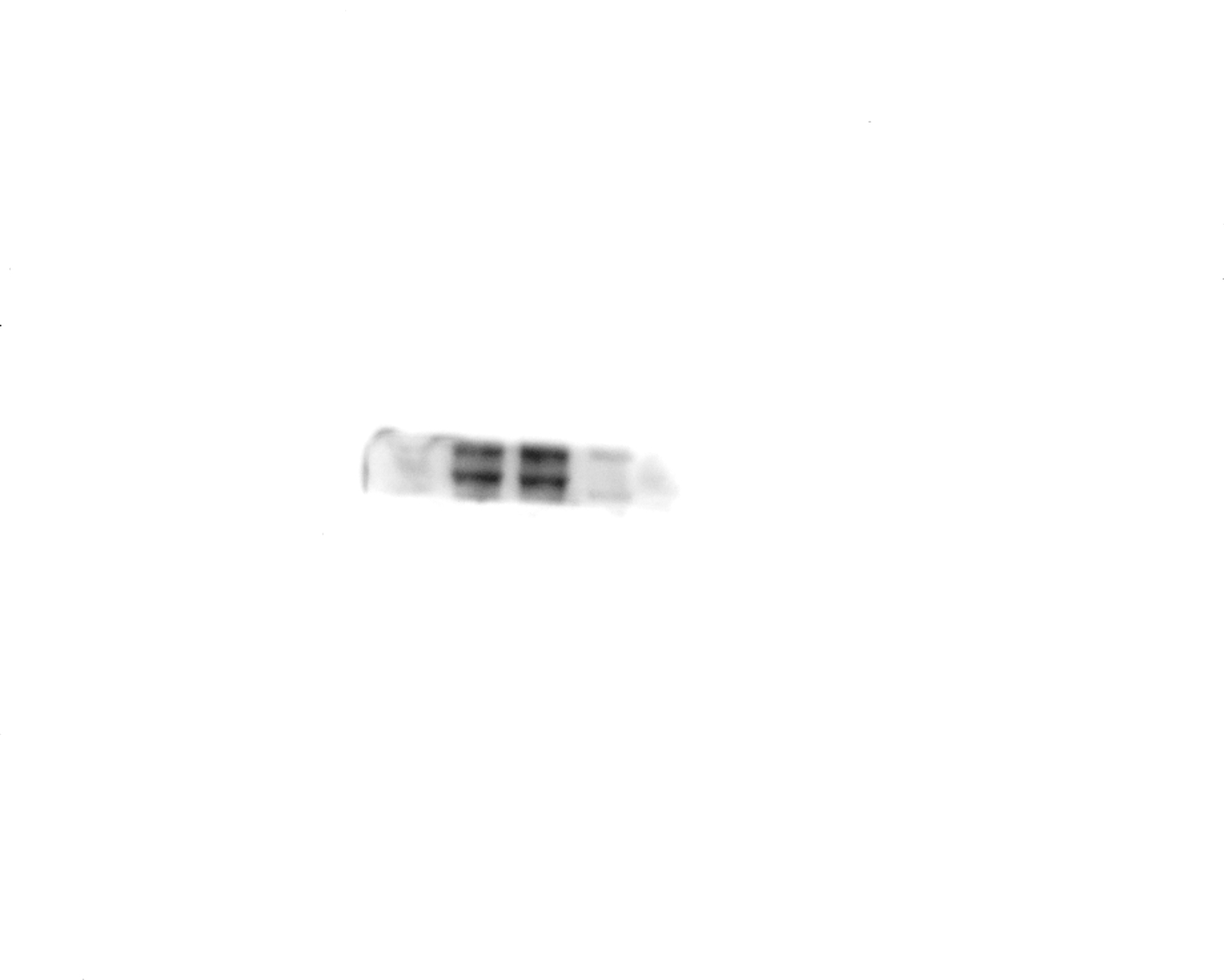

Supplement: Supplementary file 10 [file Data_Sheet_6.ZIP › Fig.4/TGF-b1.jpg]

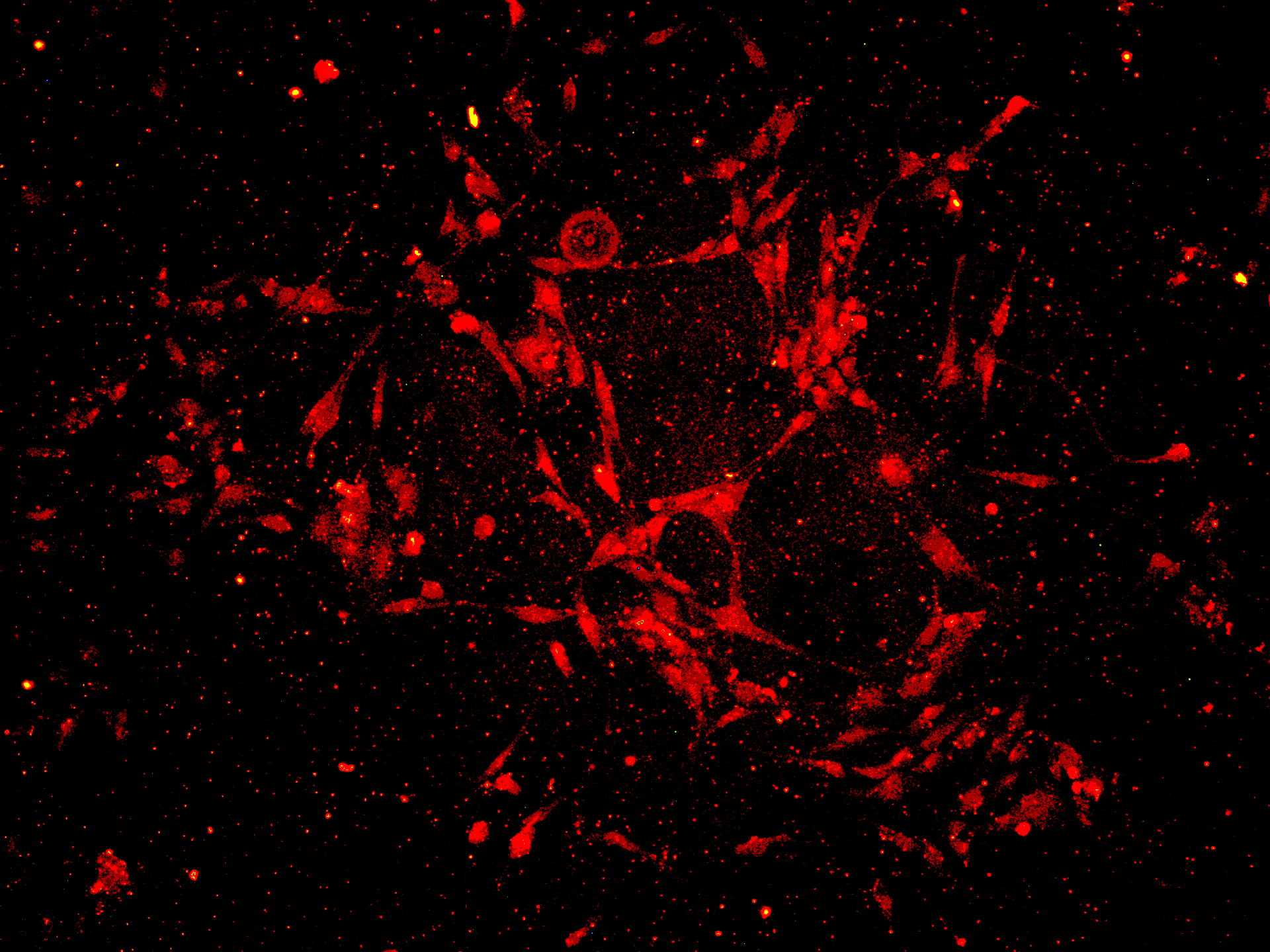

Supplement: Supplementary file 10 [file Data_Sheet_6.ZIP › Fig.4/AGEs-VIM.tif]

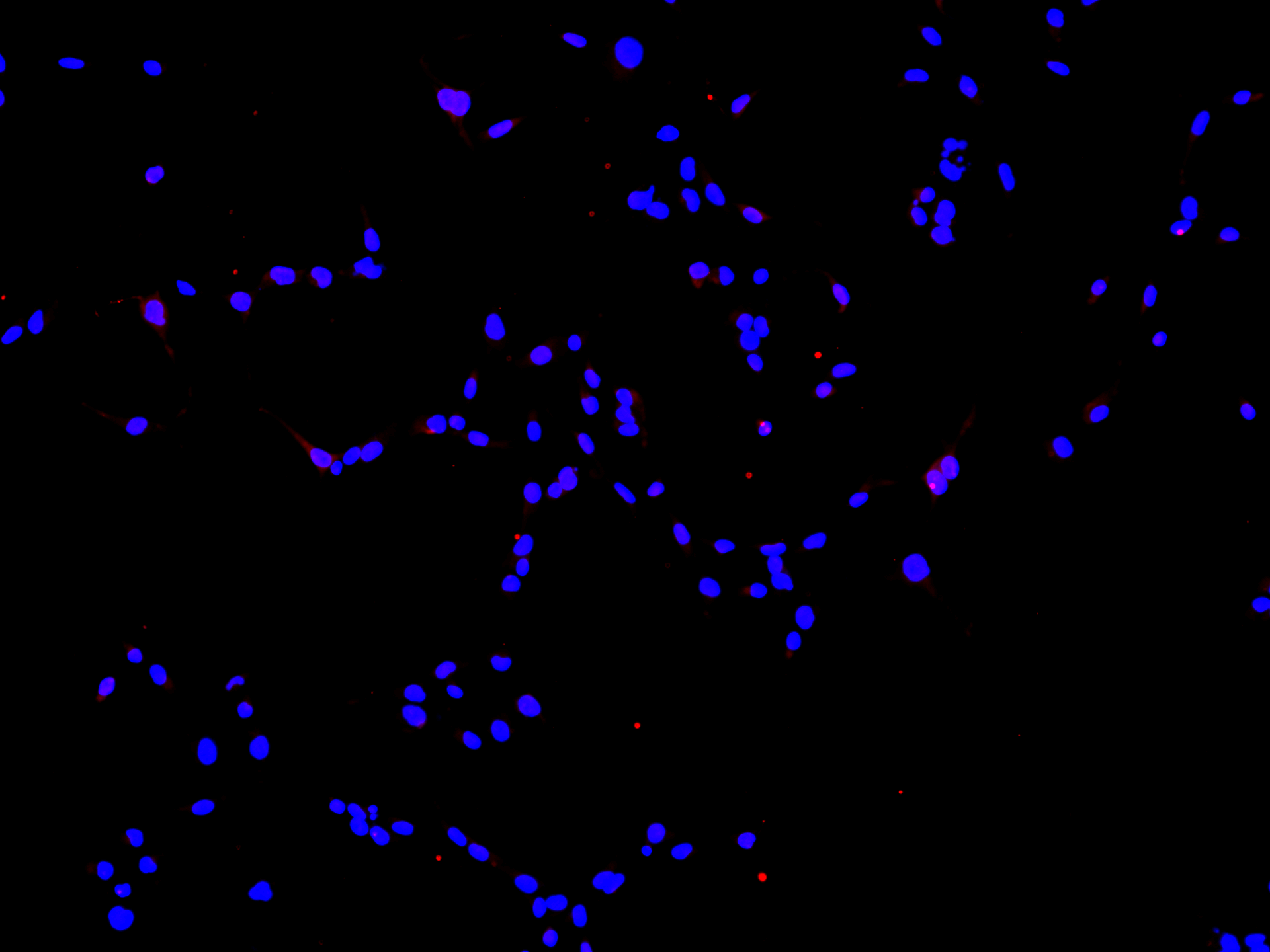

Supplement: Supplementary file 10 [file Data_Sheet_6.ZIP › Fig.4/CTRL-a-SMA-DAPI.tif]

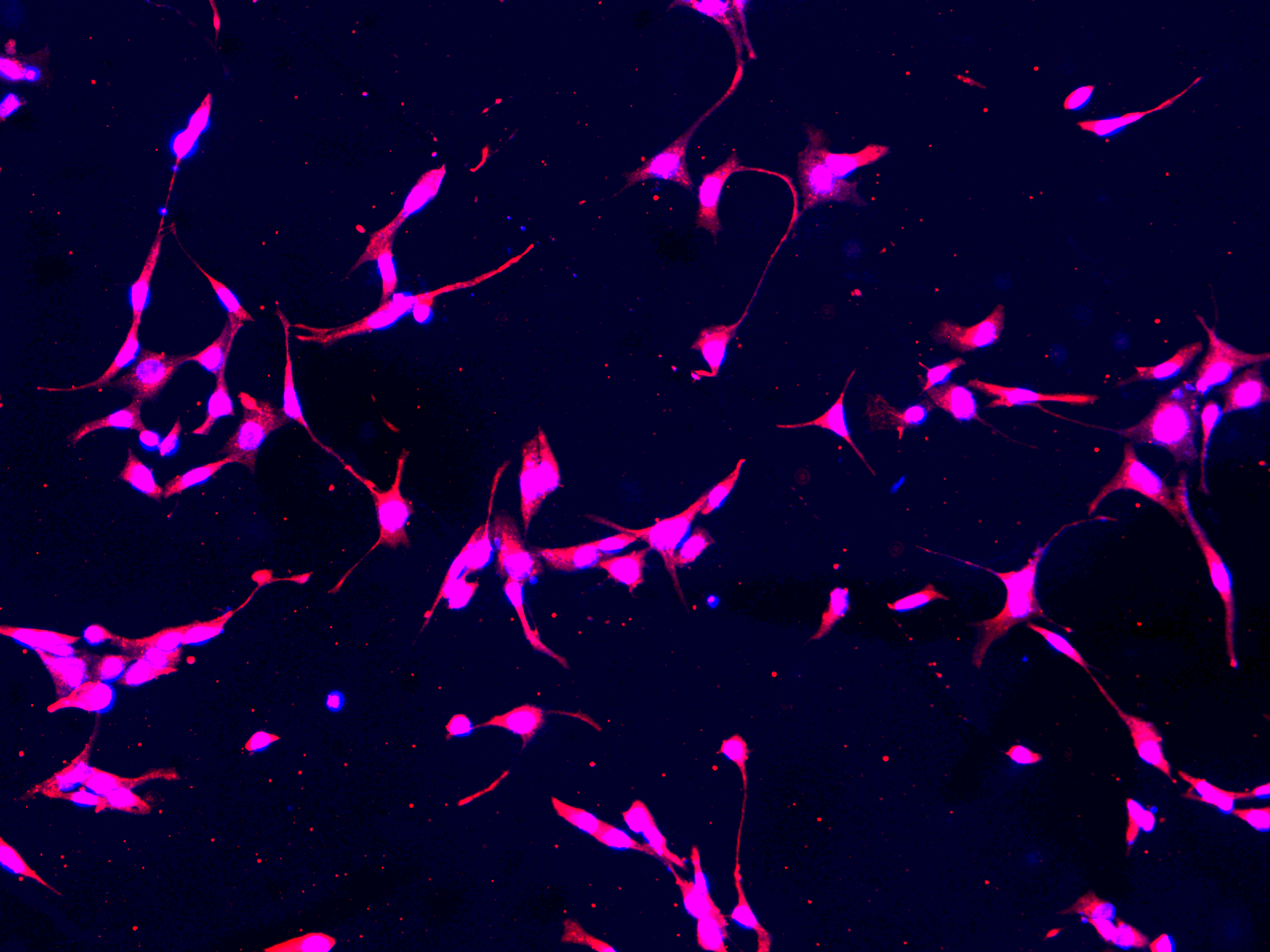

Supplement: Supplementary file 10 [file Data_Sheet_6.ZIP › Fig.4/AGEs-a-SMA-Merge.tif]

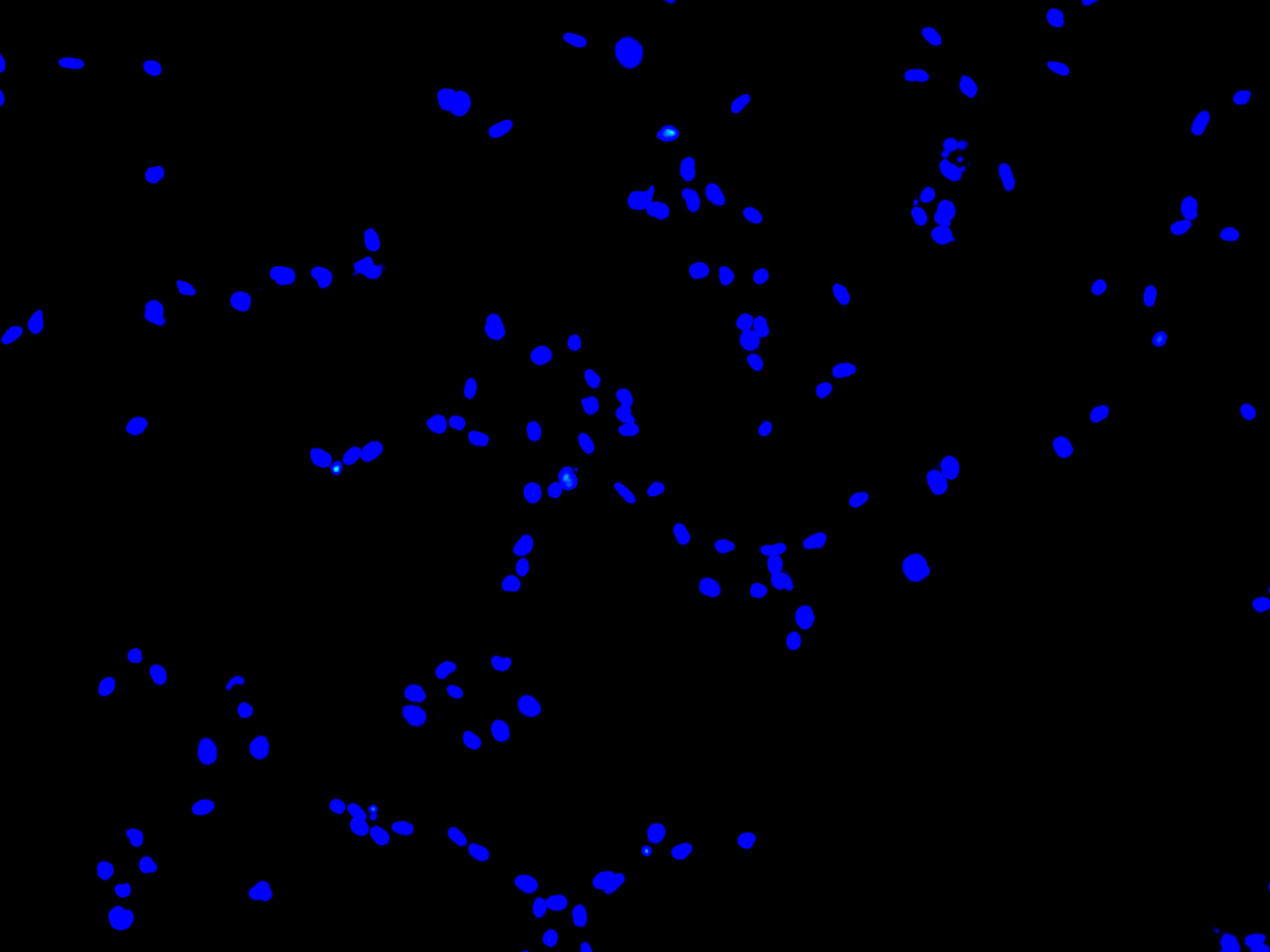

Supplement: Supplementary file 10 [file Data_Sheet_6.ZIP › Fig.4/CTRL-a-SMA-Merge.tif]

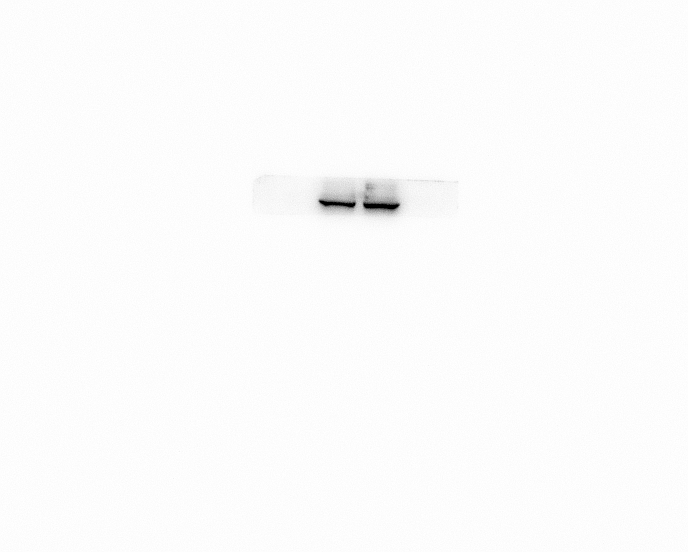

Supplement: Supplementary file 10 [file Data_Sheet_6.ZIP › Fig.4/VIM-1-20S.jpg]

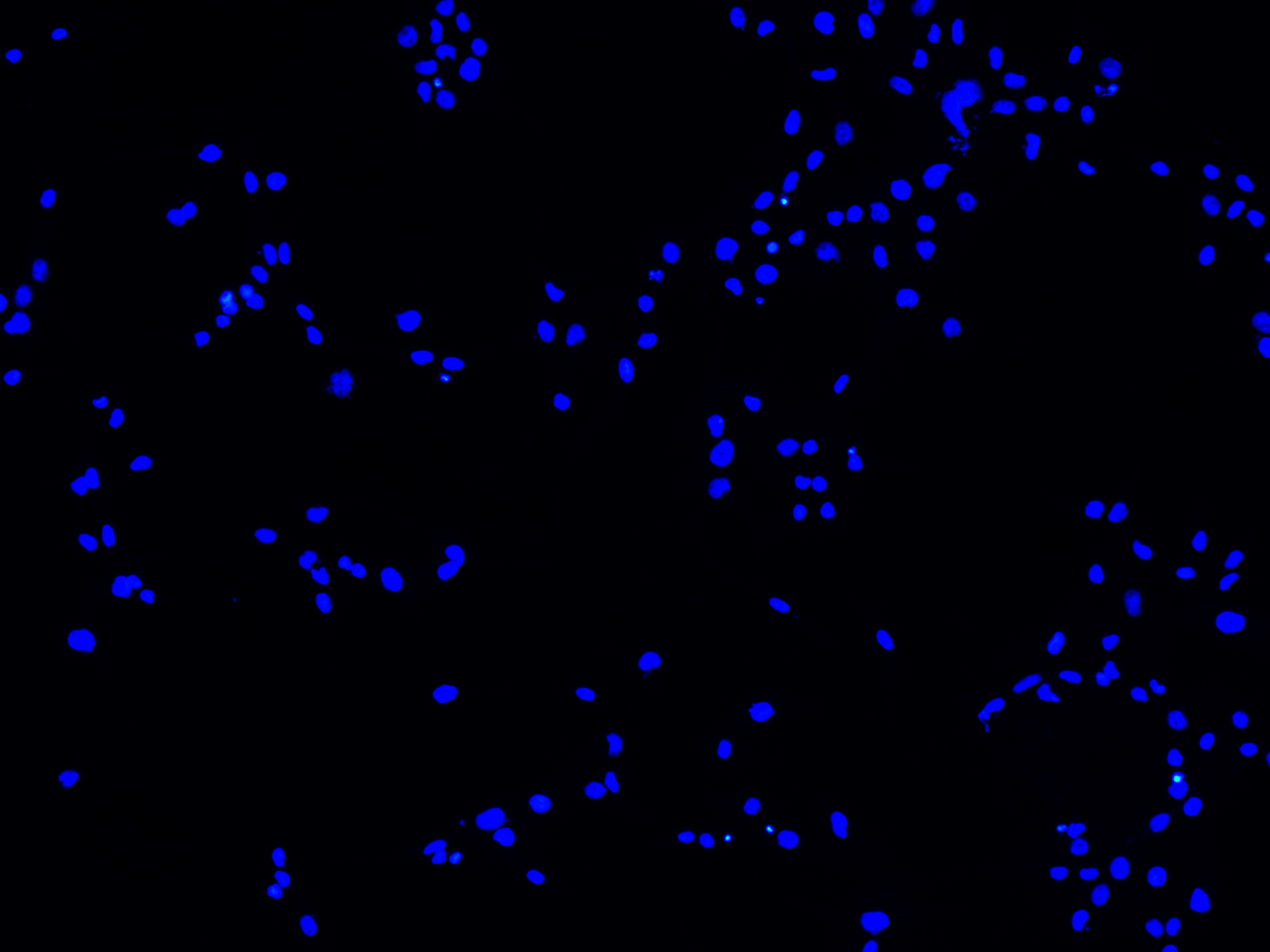

Supplement: Supplementary file 10 [file Data_Sheet_6.ZIP › Fig.4/CTRL-VIM-DAPI.tif]

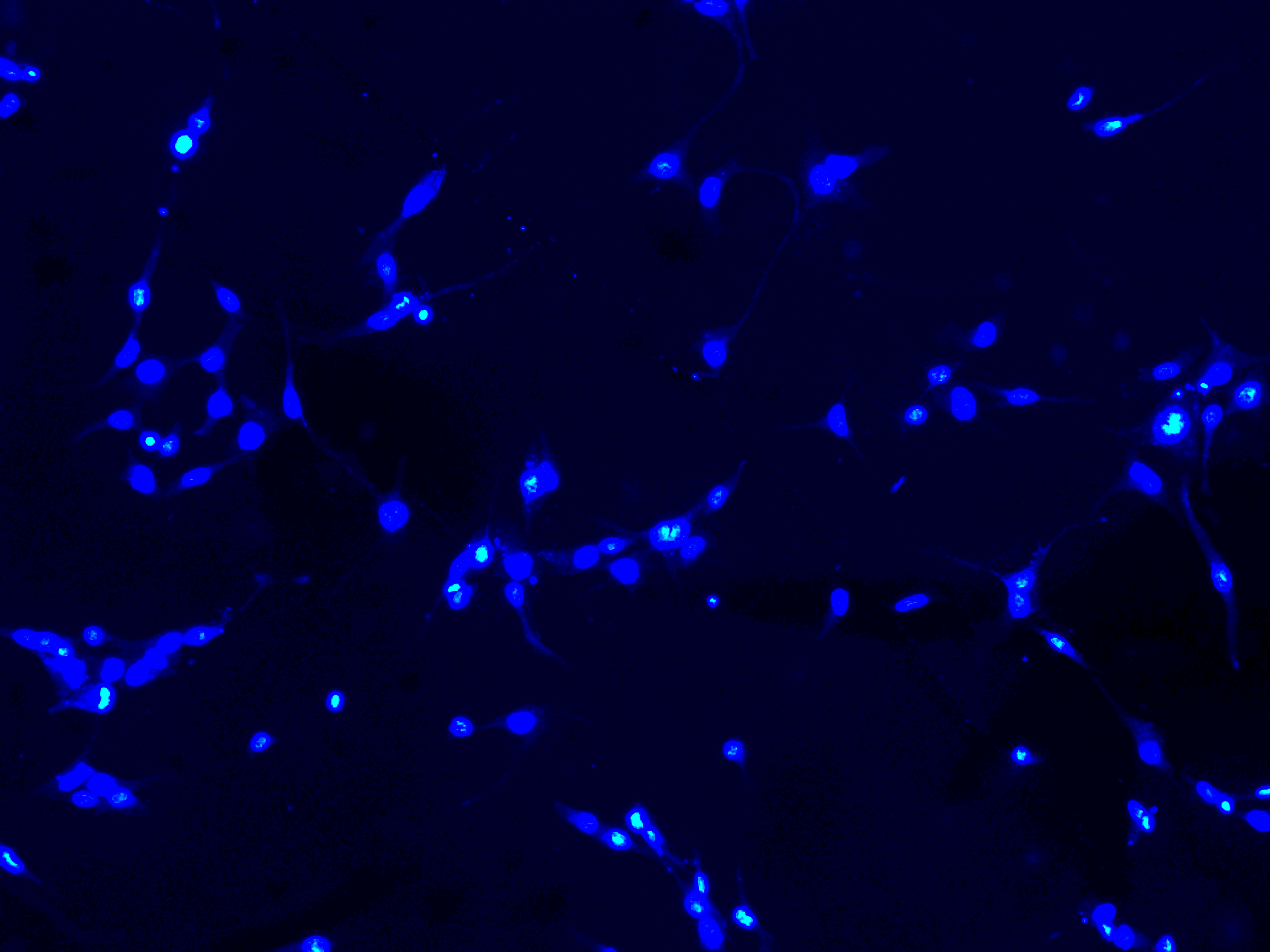

Supplement: Supplementary file 10 [file Data_Sheet_6.ZIP › Fig.4/AGEs-a-SMA-DAPI.tif]

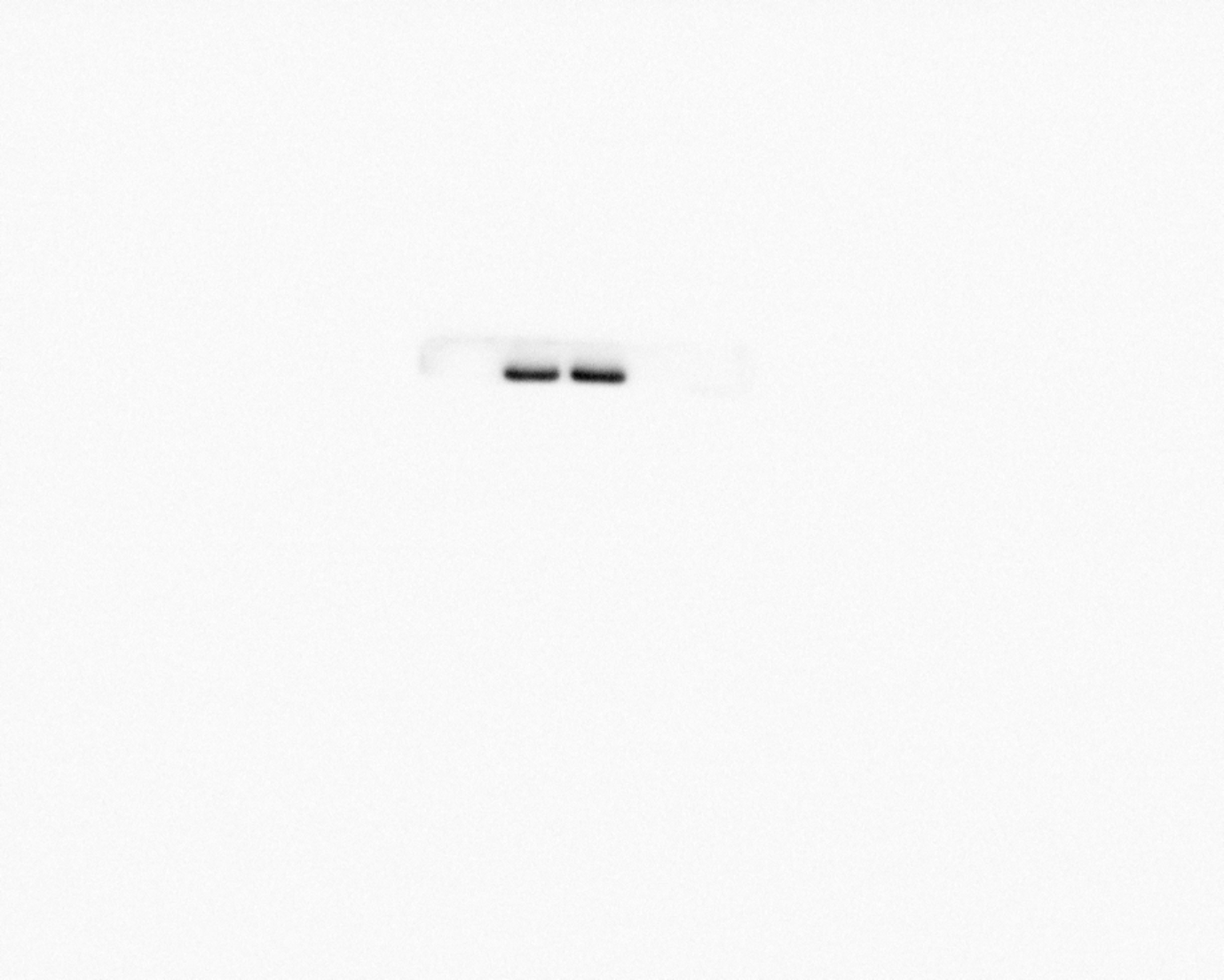

Supplement: Supplementary file 10 [file Data_Sheet_6.ZIP › Fig.4/GAPDH.tif]

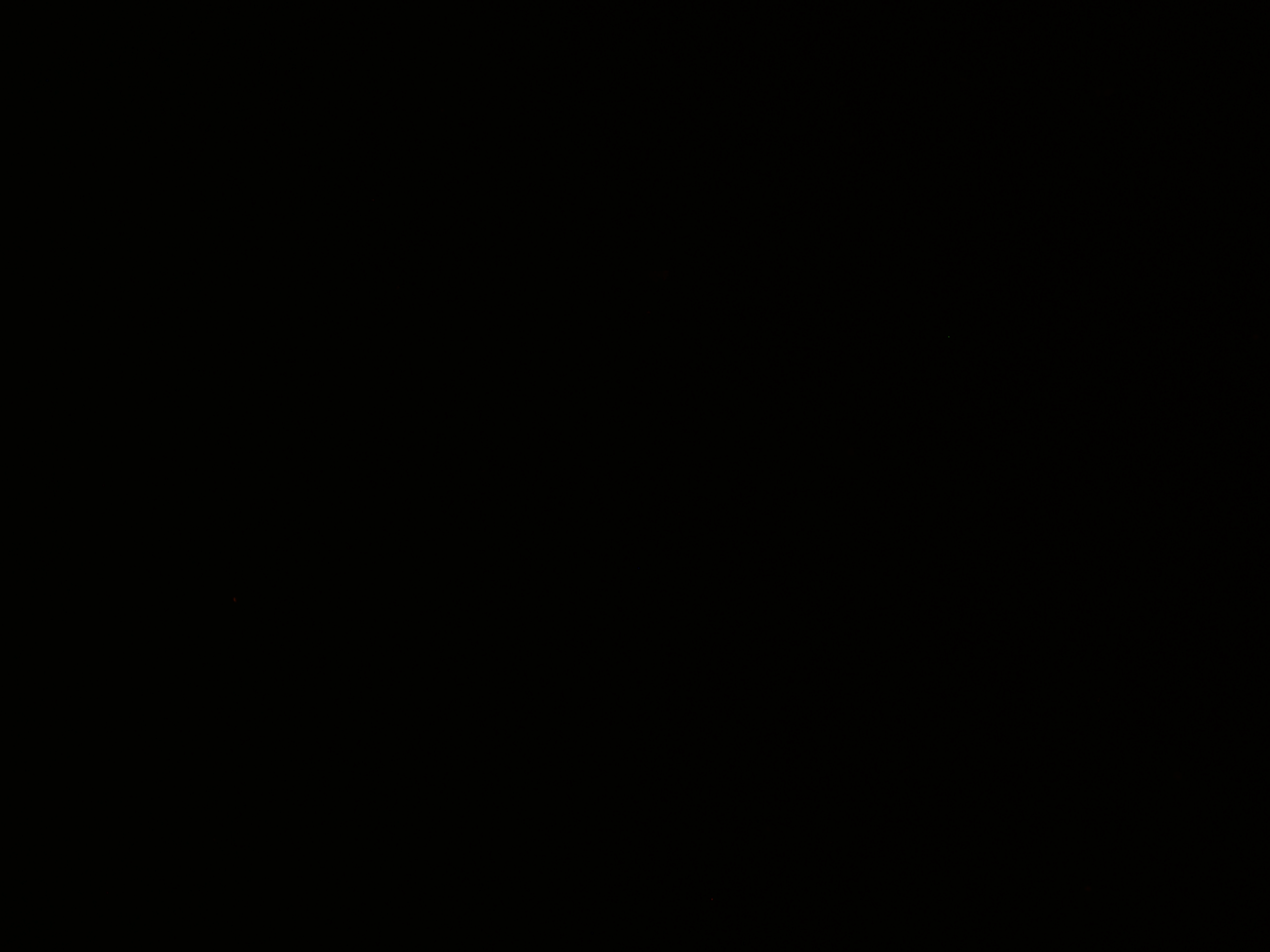

Supplement: Supplementary file 10 [file Data_Sheet_6.ZIP › Fig.4/CTRL-VIM.tif]

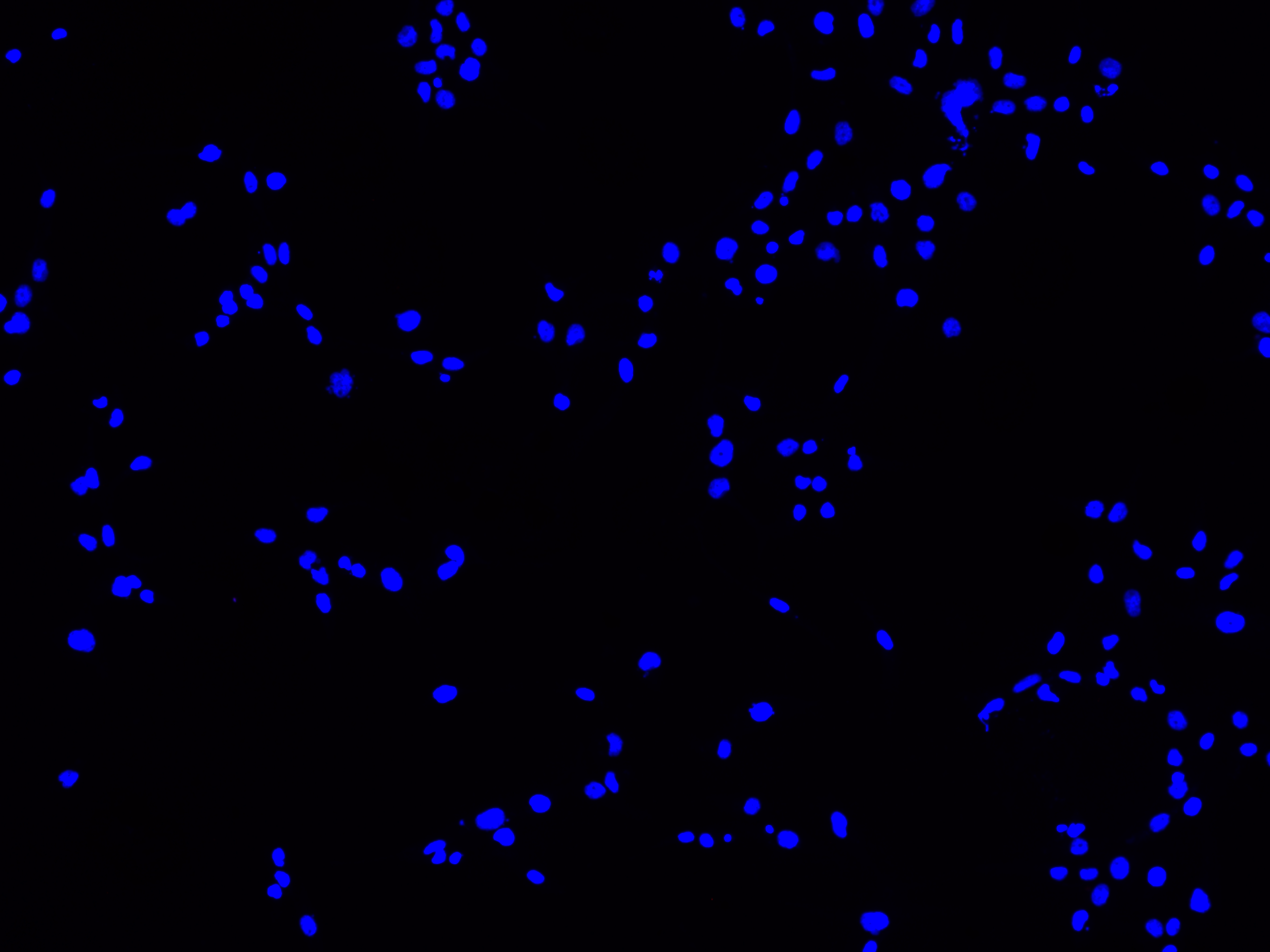

Supplement: Supplementary file 10 [file Data_Sheet_6.ZIP › Fig.4/CTRL-VIM-Merge.tif]

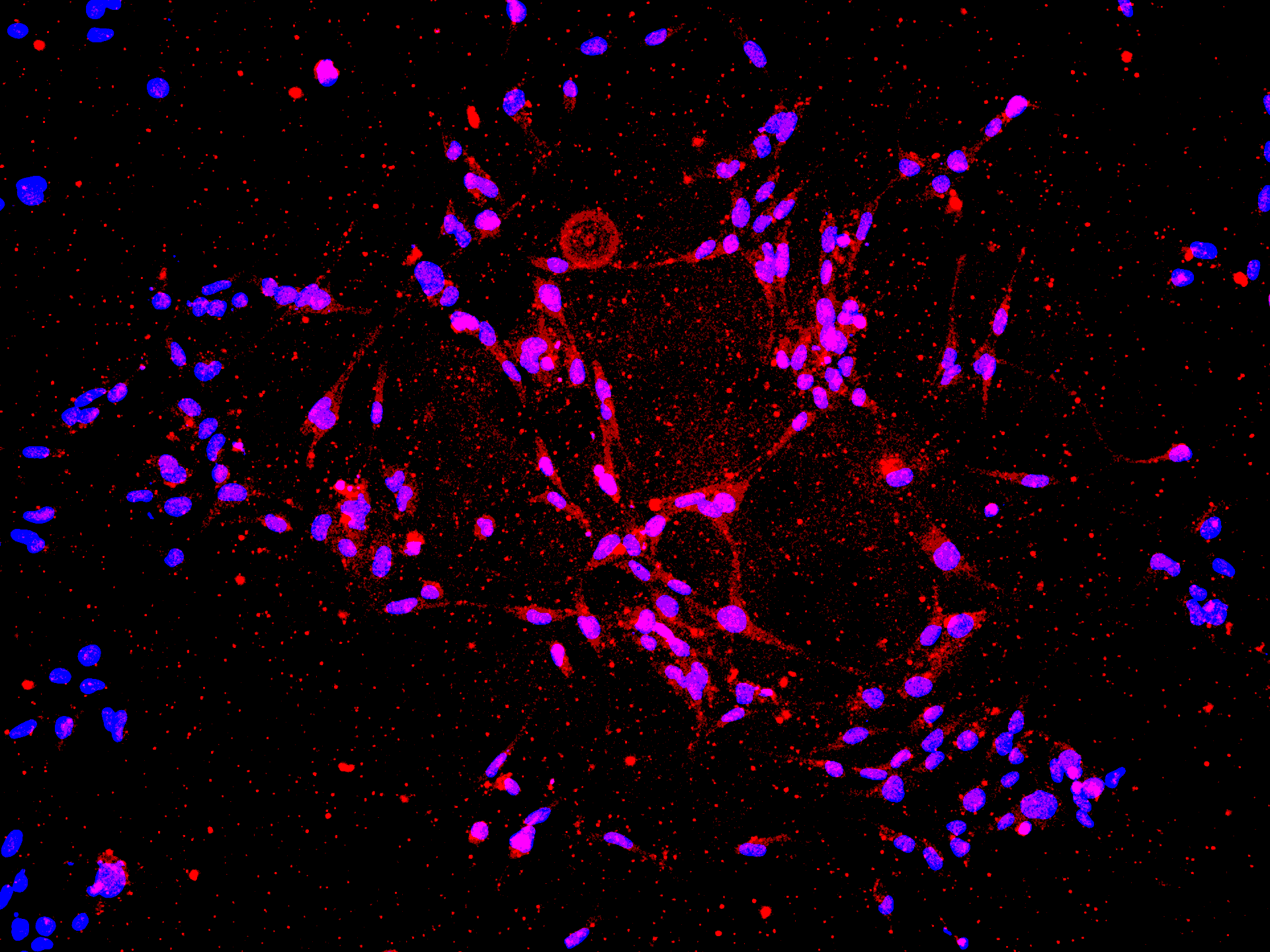

Supplement: Supplementary file 10 [file Data_Sheet_6.ZIP › Fig.4/AGEs-VIM-Merge.tif]

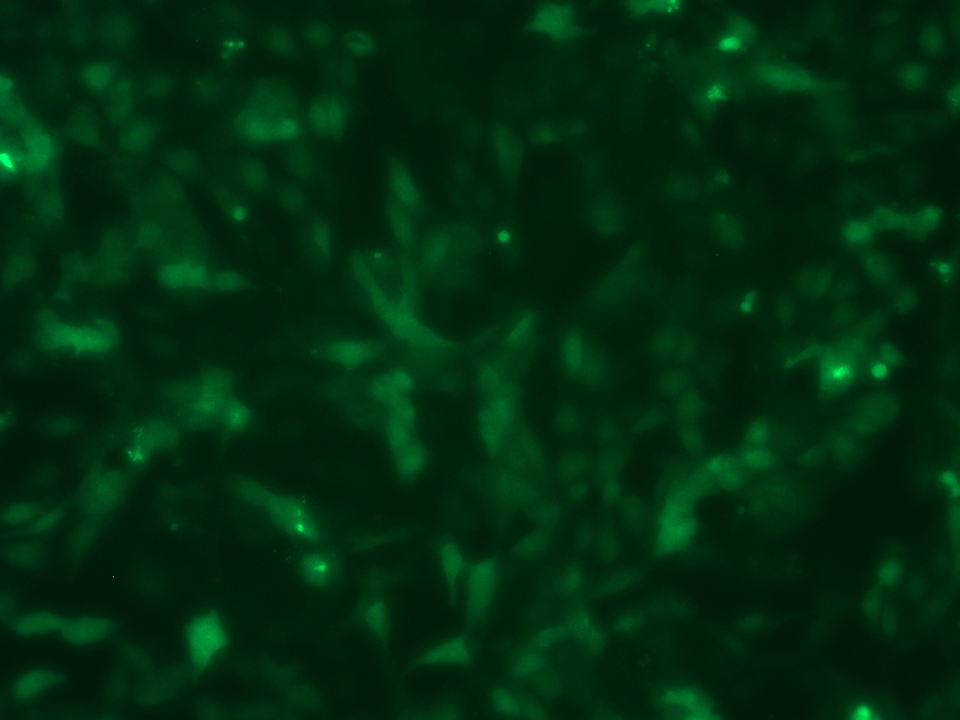

Supplement: Supplementary file 11 [file Data_Sheet_7.ZIP › Fig.5/48h-MOI=40.tif]

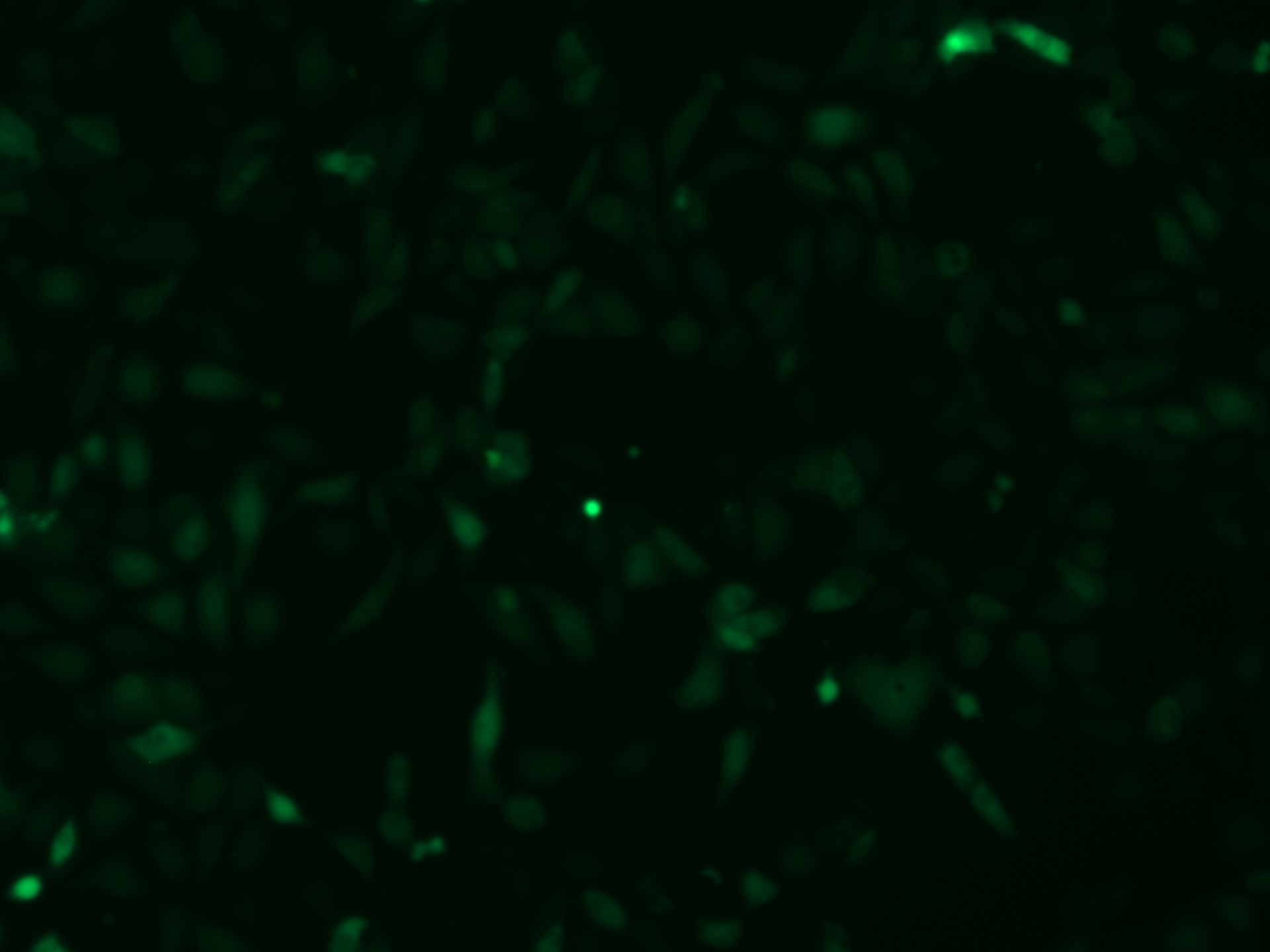

Supplement: Supplementary file 11 [file Data_Sheet_7.ZIP › Fig.5/24h-MOI=40.tif]

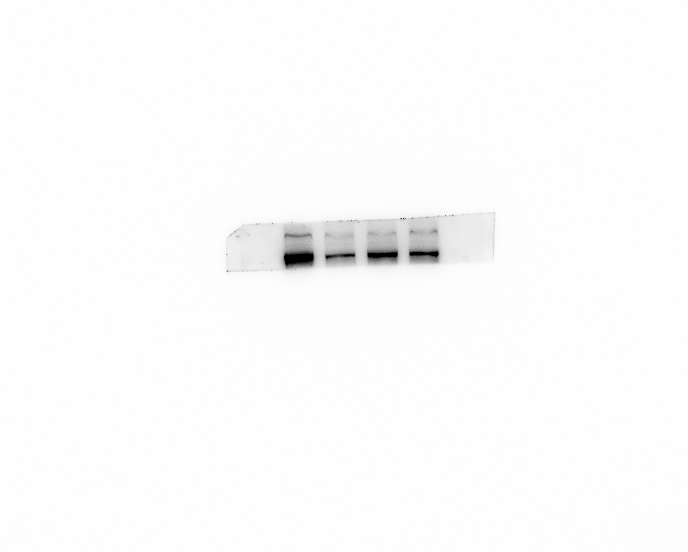

Supplement: Supplementary file 11 [file Data_Sheet_7.ZIP › Fig.5/JMJD1A.jpg]

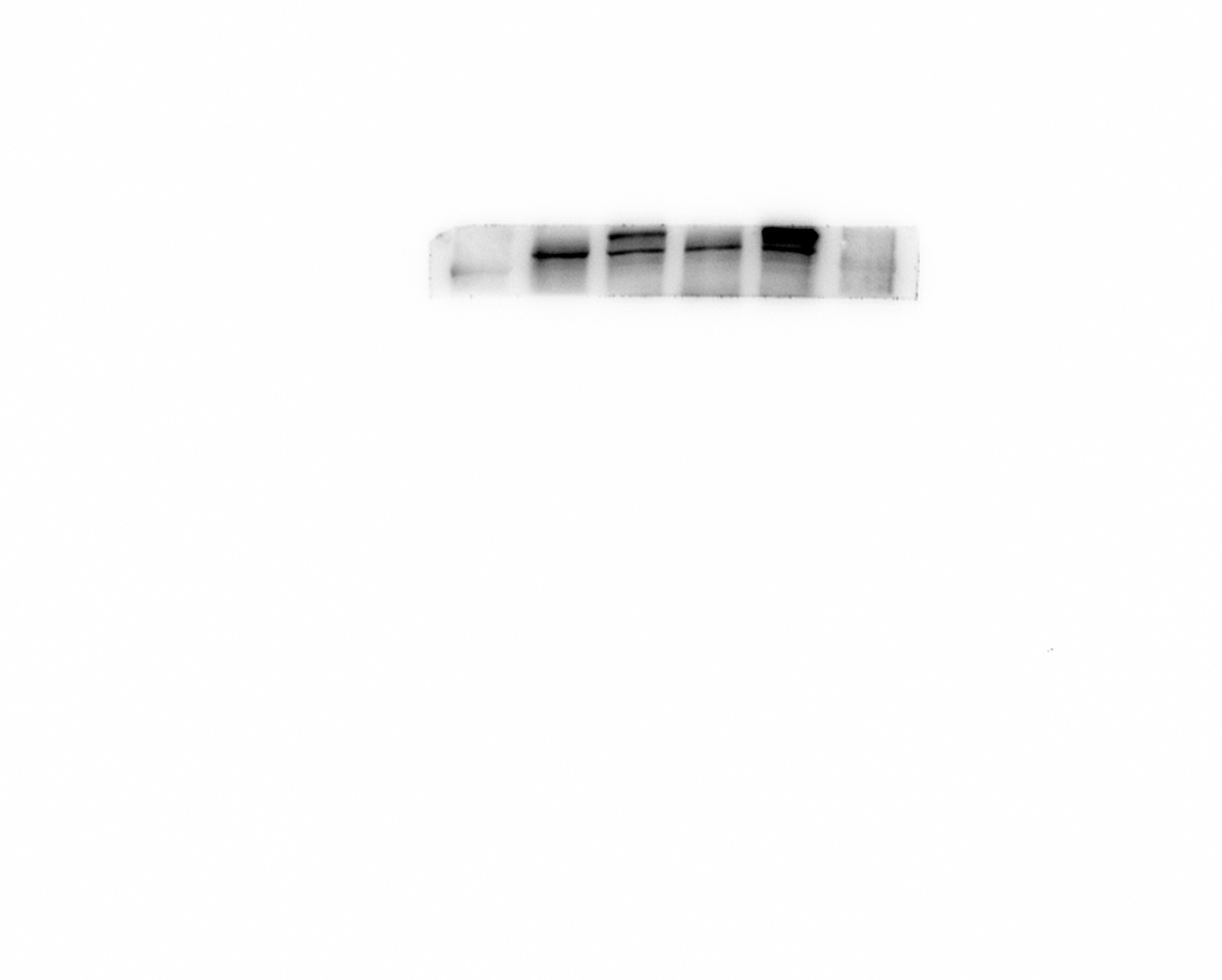

Supplement: Supplementary file 11 [file Data_Sheet_7.ZIP › Fig.5/ECAD.jpg]

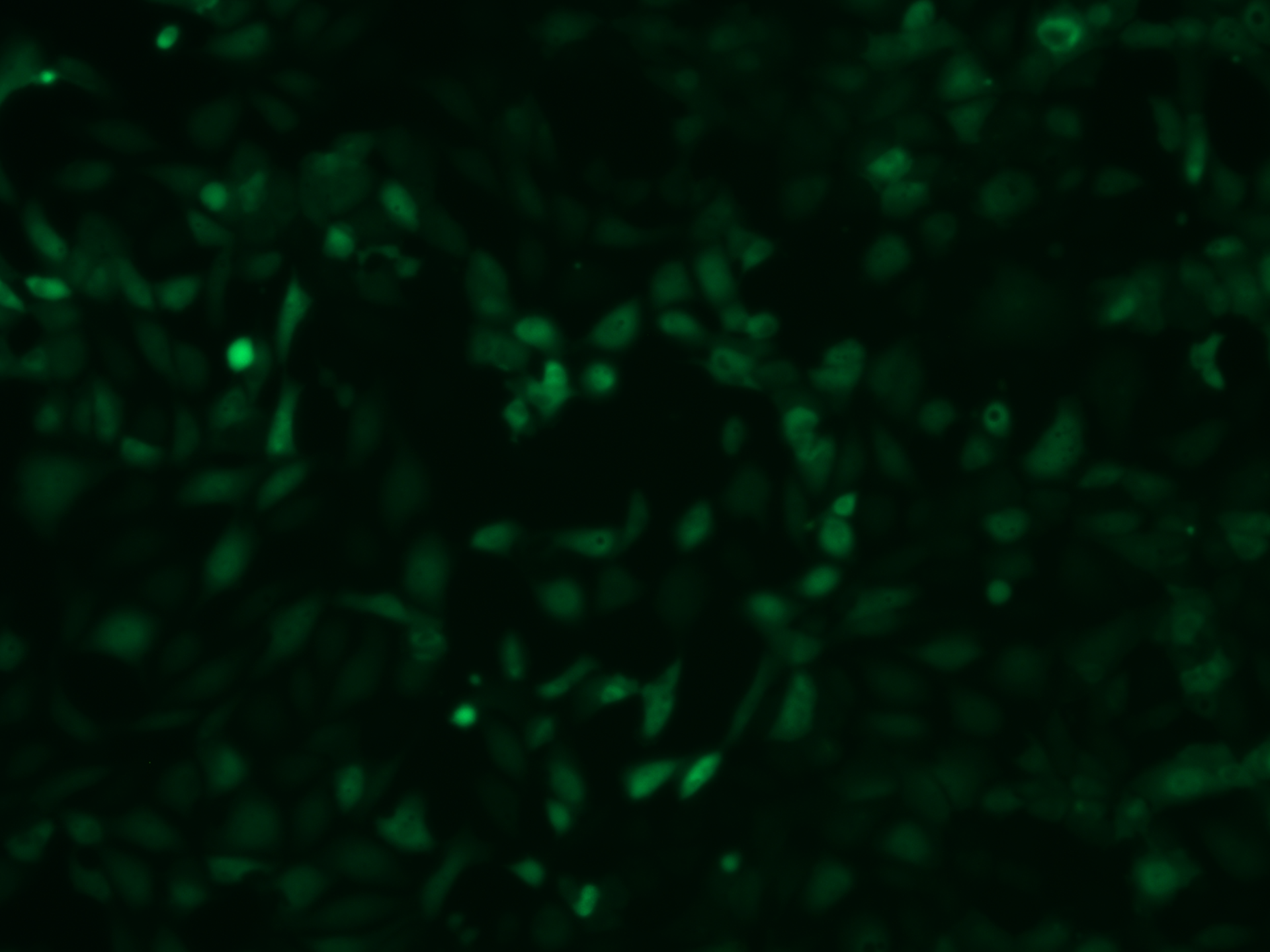

Supplement: Supplementary file 11 [file Data_Sheet_7.ZIP › Fig.5/24h-MOI=50.tif]

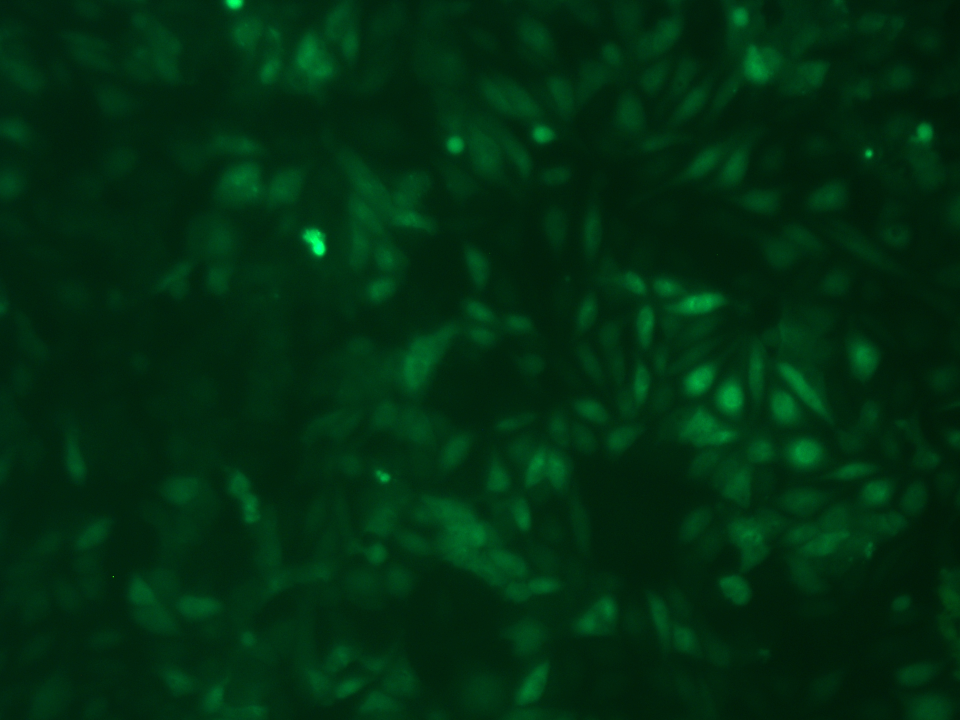

Supplement: Supplementary file 11 [file Data_Sheet_7.ZIP › Fig.5/48h-MOI=50.tif]

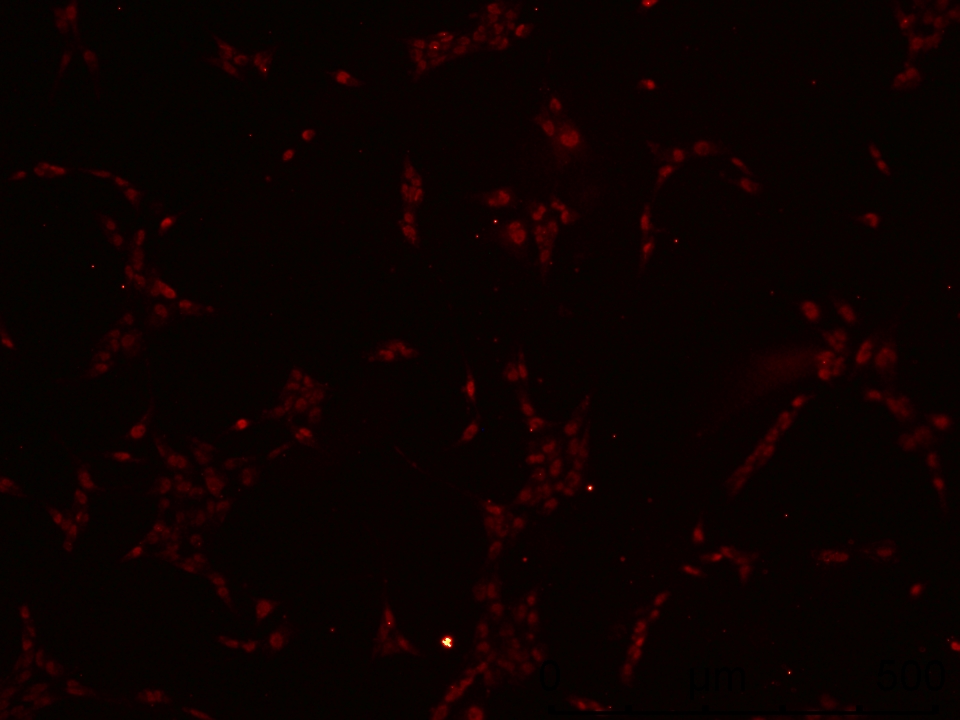

Supplement: Supplementary file 11 [file Data_Sheet_7.ZIP › Fig.5/shCtrl-Ctrl-JMJD1A.jpg]

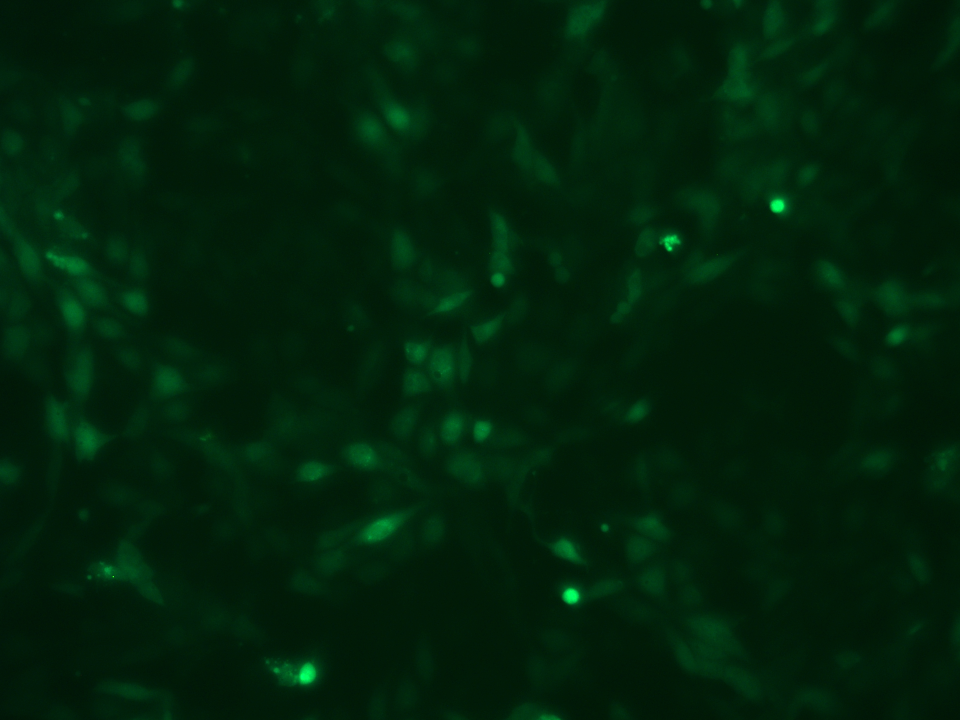

Supplement: Supplementary file 11 [file Data_Sheet_7.ZIP › Fig.5/48h-MOI=20.tif]

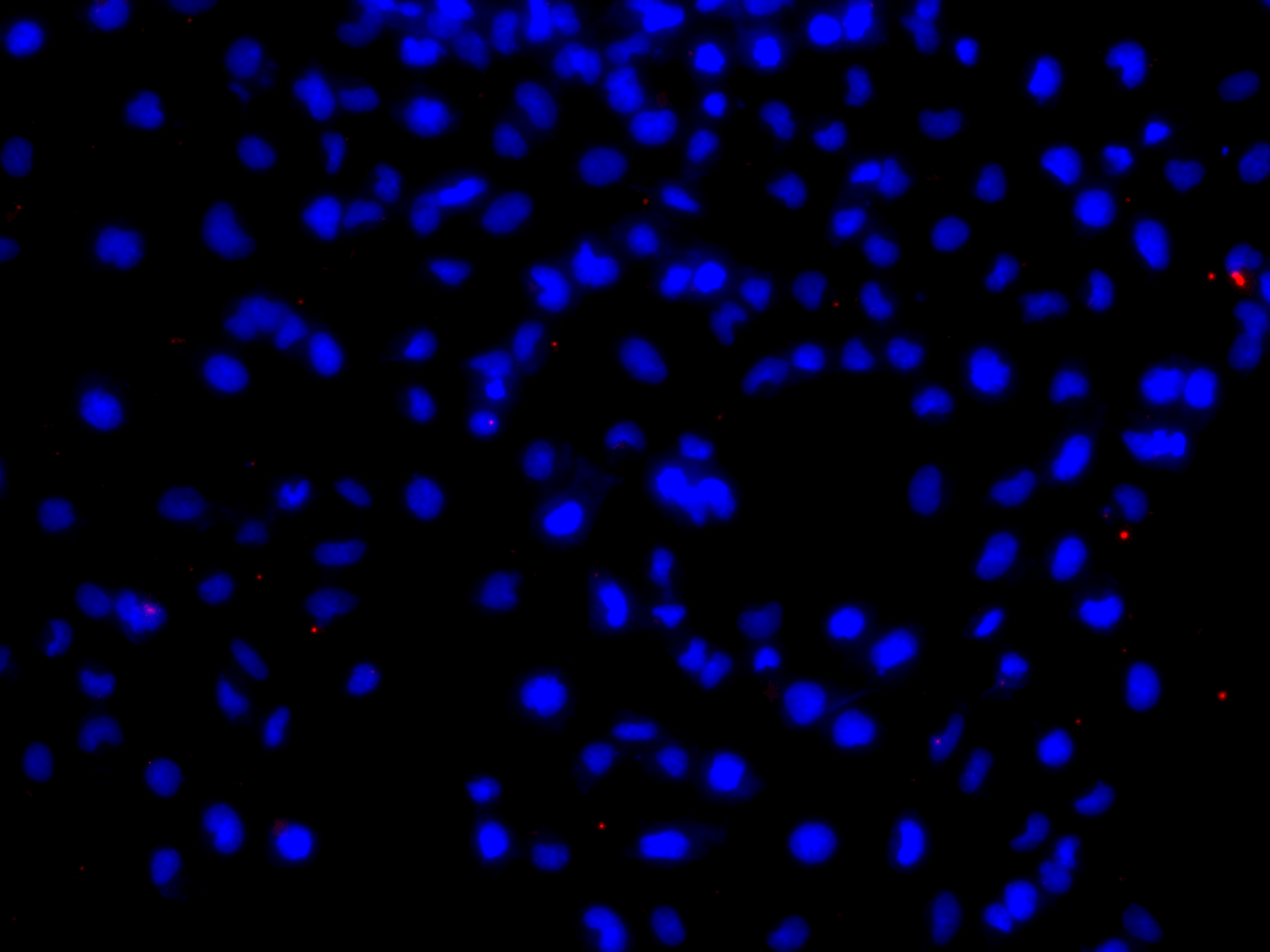

Supplement: Supplementary file 11 [file Data_Sheet_7.ZIP › Fig.5/shCtrl-AGEs-Merge-a-SMA.tif]

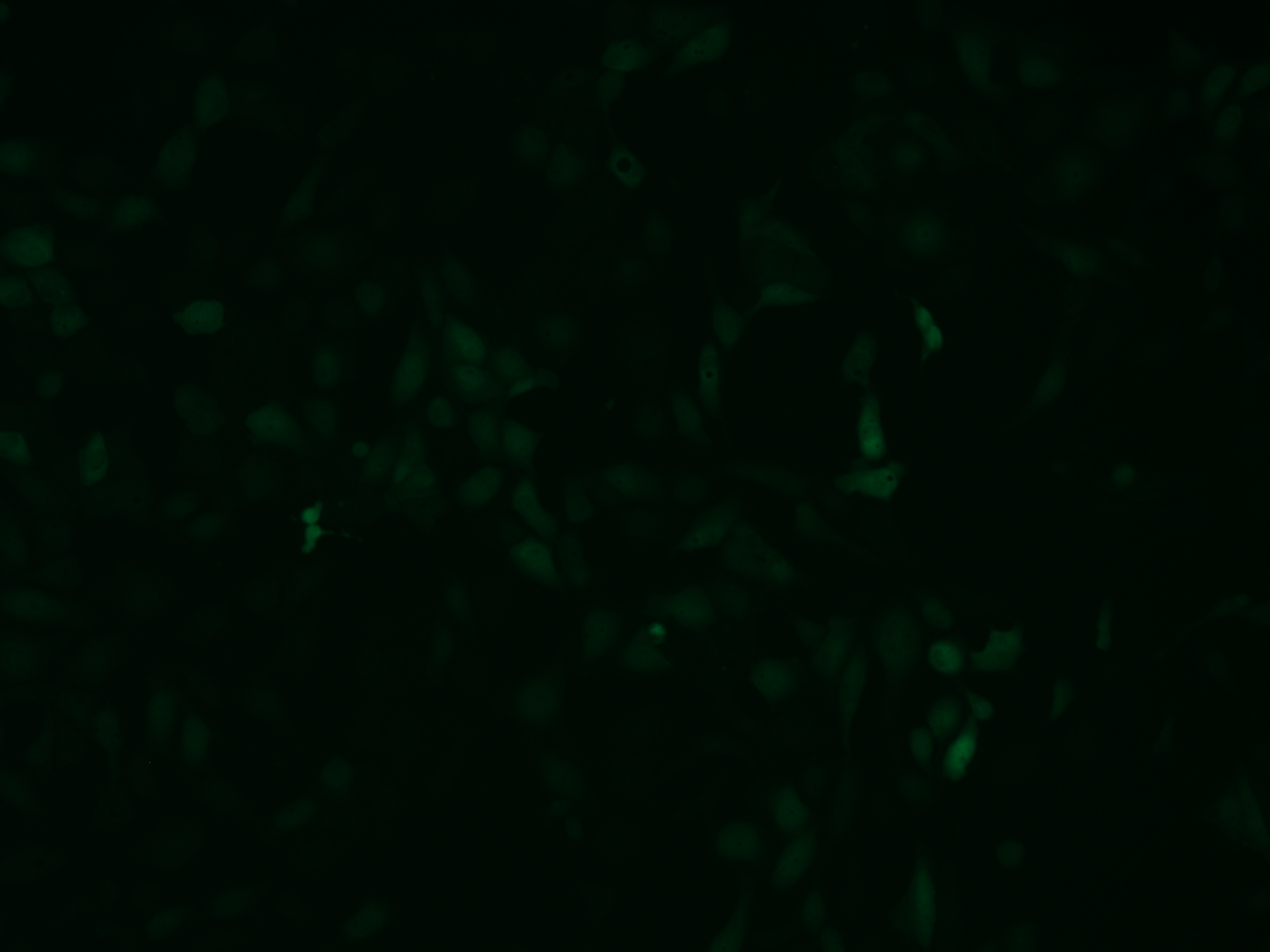

Supplement: Supplementary file 11 [file Data_Sheet_7.ZIP › Fig.5/24h-MOI=20.tif]

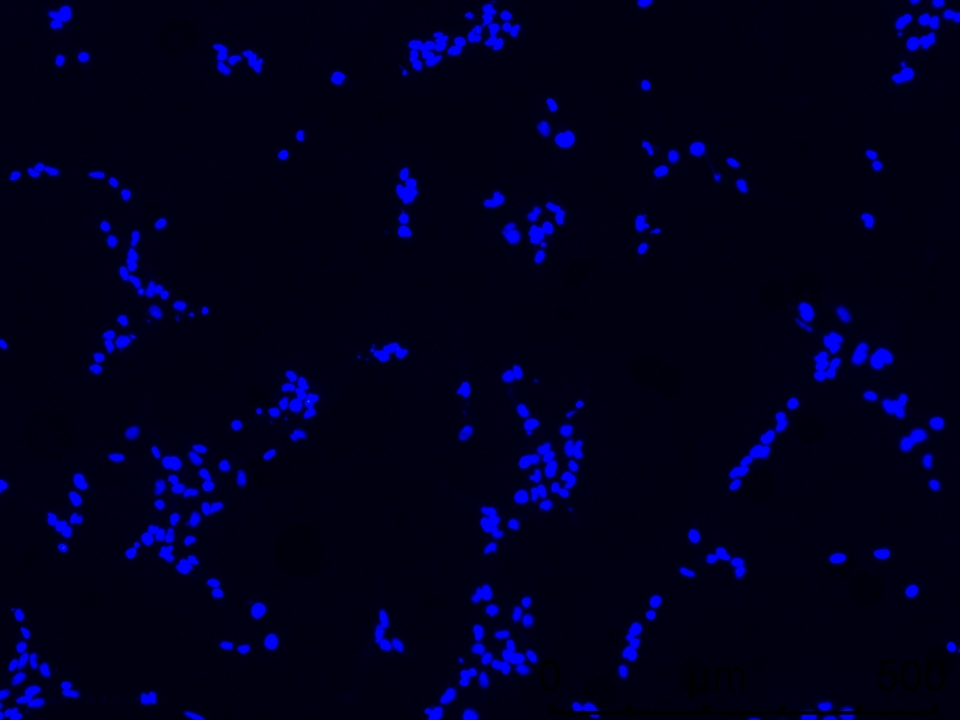

Supplement: Supplementary file 11 [file Data_Sheet_7.ZIP › Fig.5/shCtrl-Ctrl-DAPI-JMJD1A.jpg]

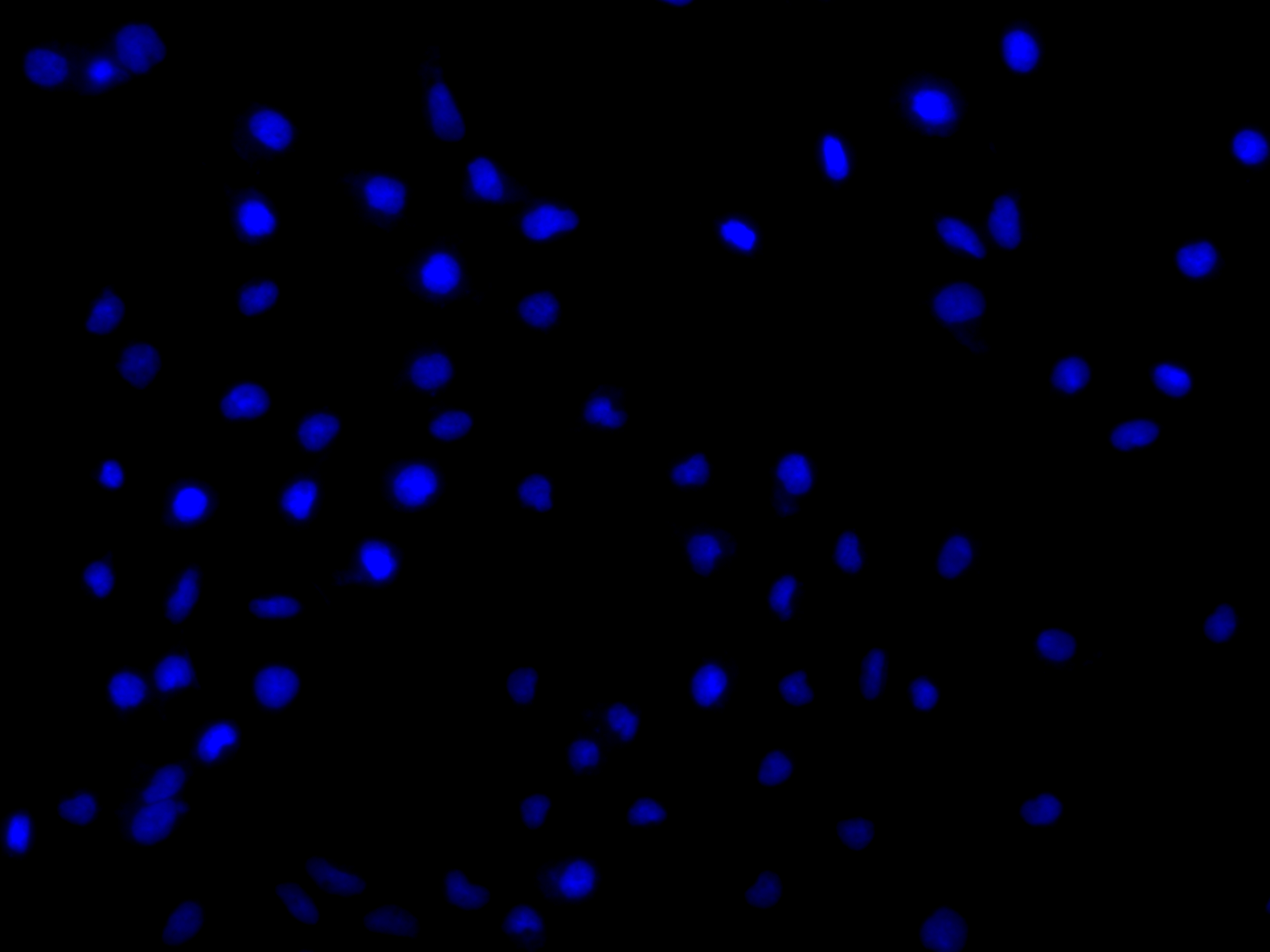

Supplement: Supplementary file 11 [file Data_Sheet_7.ZIP › Fig.5/shCtrl-Ctrl-DAPI-a-SMA.tif]

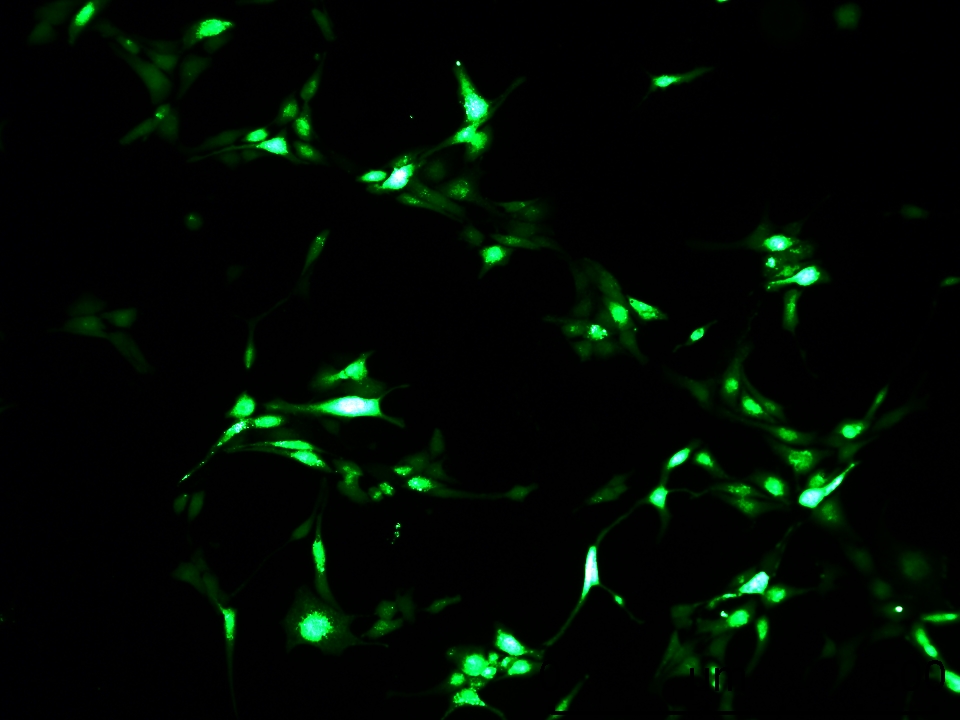

Supplement: Supplementary file 11 [file Data_Sheet_7.ZIP › Fig.5/shJMJD1A-AGEs-GFP-JMJD1A.jpg]

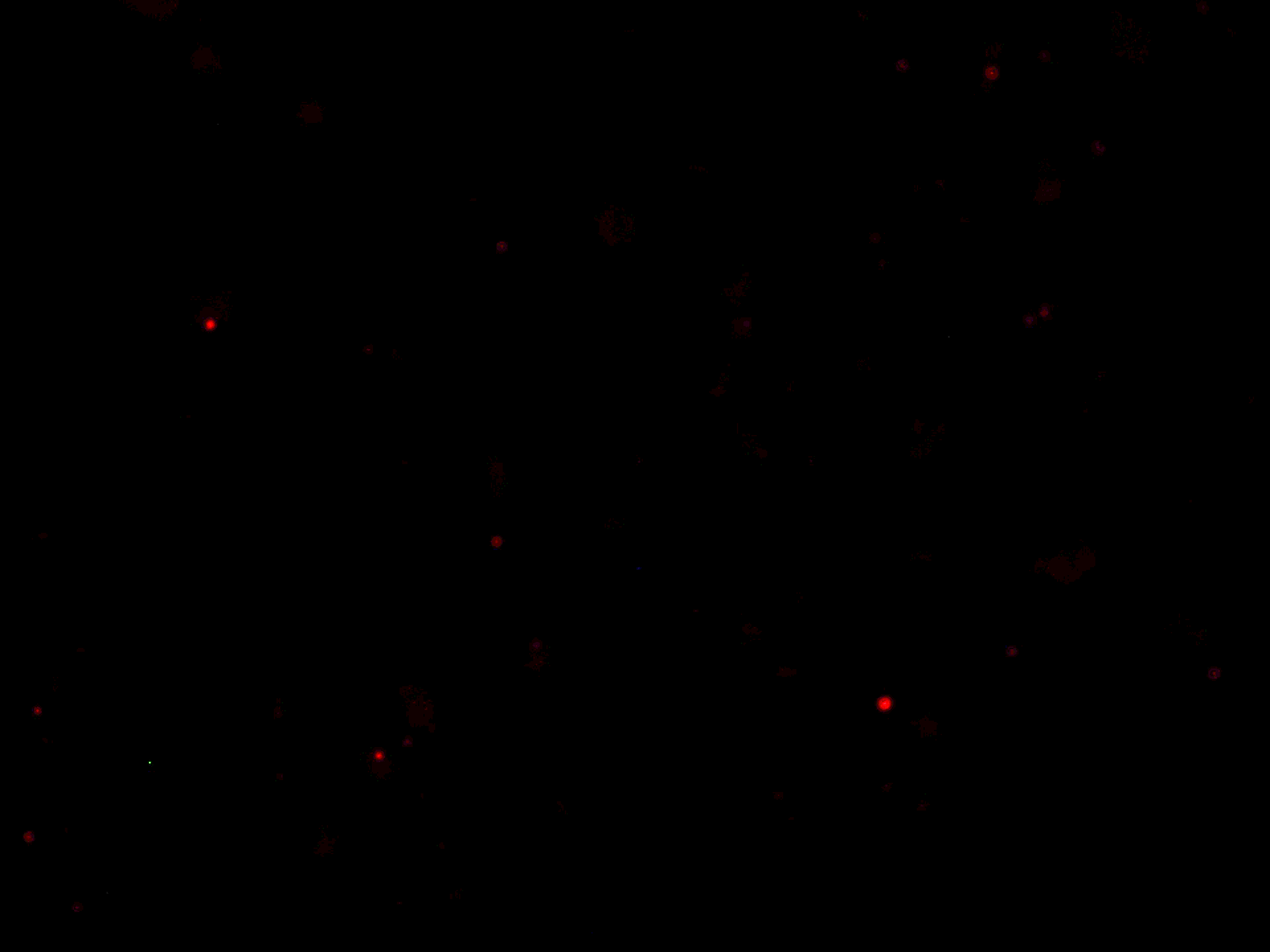

Supplement: Supplementary file 11 [file Data_Sheet_7.ZIP › Fig.5/shJMJD1A-Ctrl-a-SMA.tif]

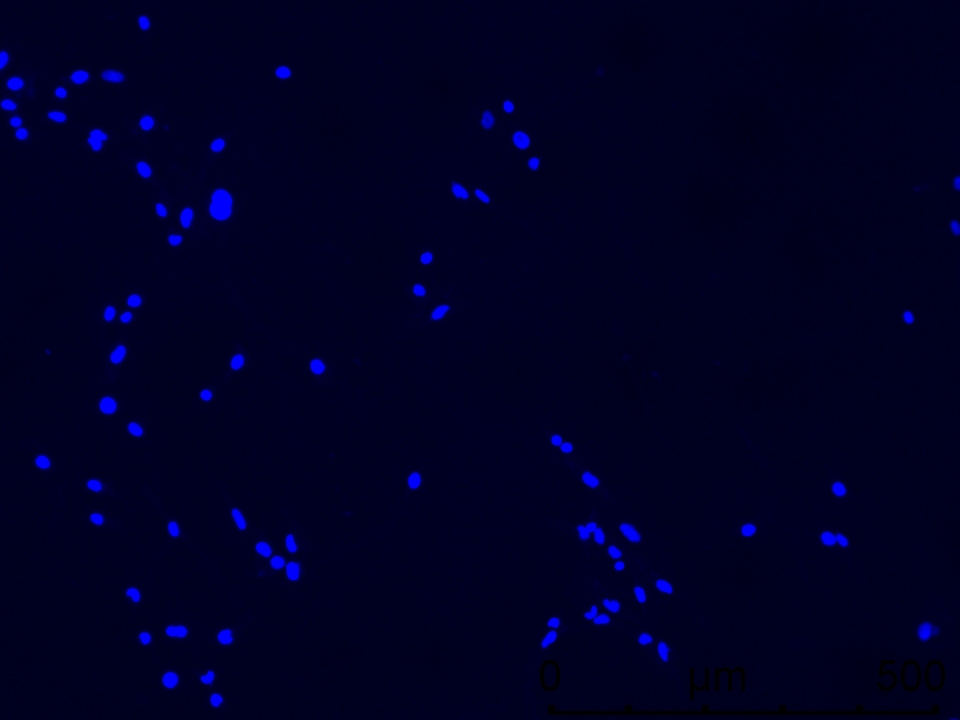

Supplement: Supplementary file 11 [file Data_Sheet_7.ZIP › Fig.5/shctrl-AGEs-DAPI-JMJD1A.jpg]

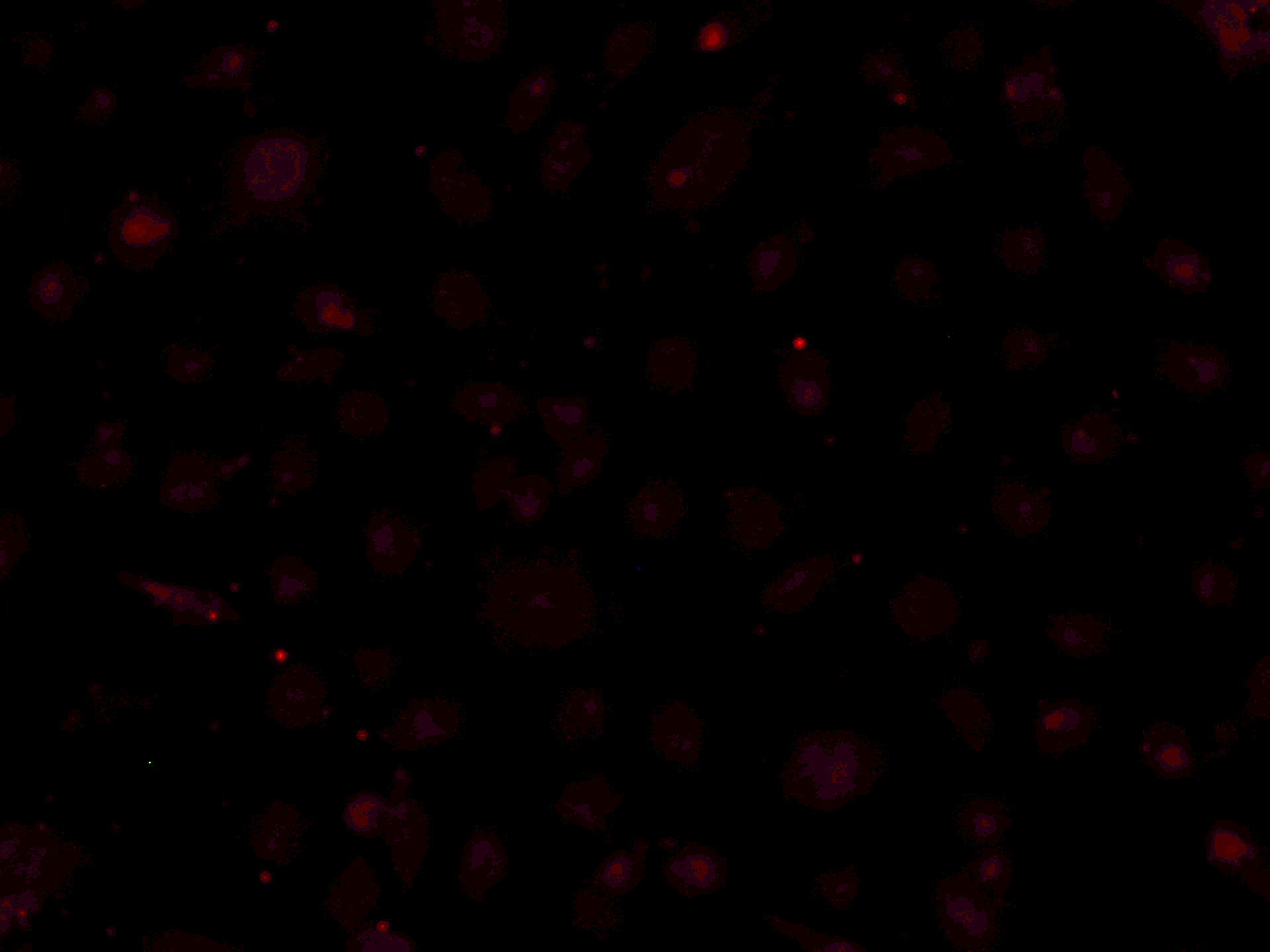

Supplement: Supplementary file 11 [file Data_Sheet_7.ZIP › Fig.5/shJMJD1A-AGEs-a-SMA.tif]

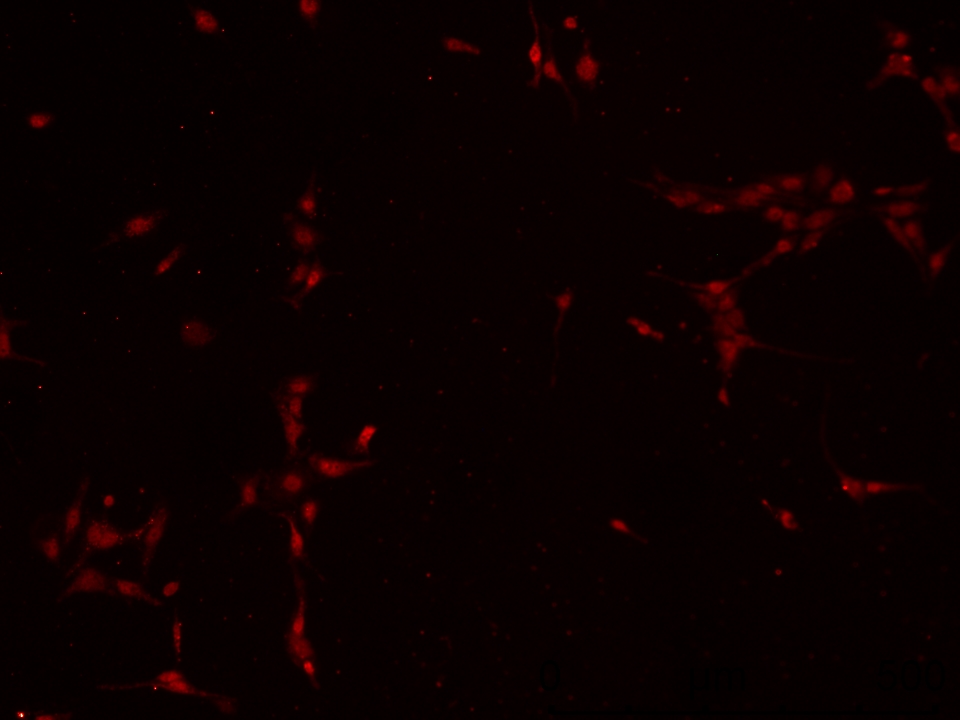

Supplement: Supplementary file 11 [file Data_Sheet_7.ZIP › Fig.5/shJMJD1A-Ctrl-JMJD1A.jpg]

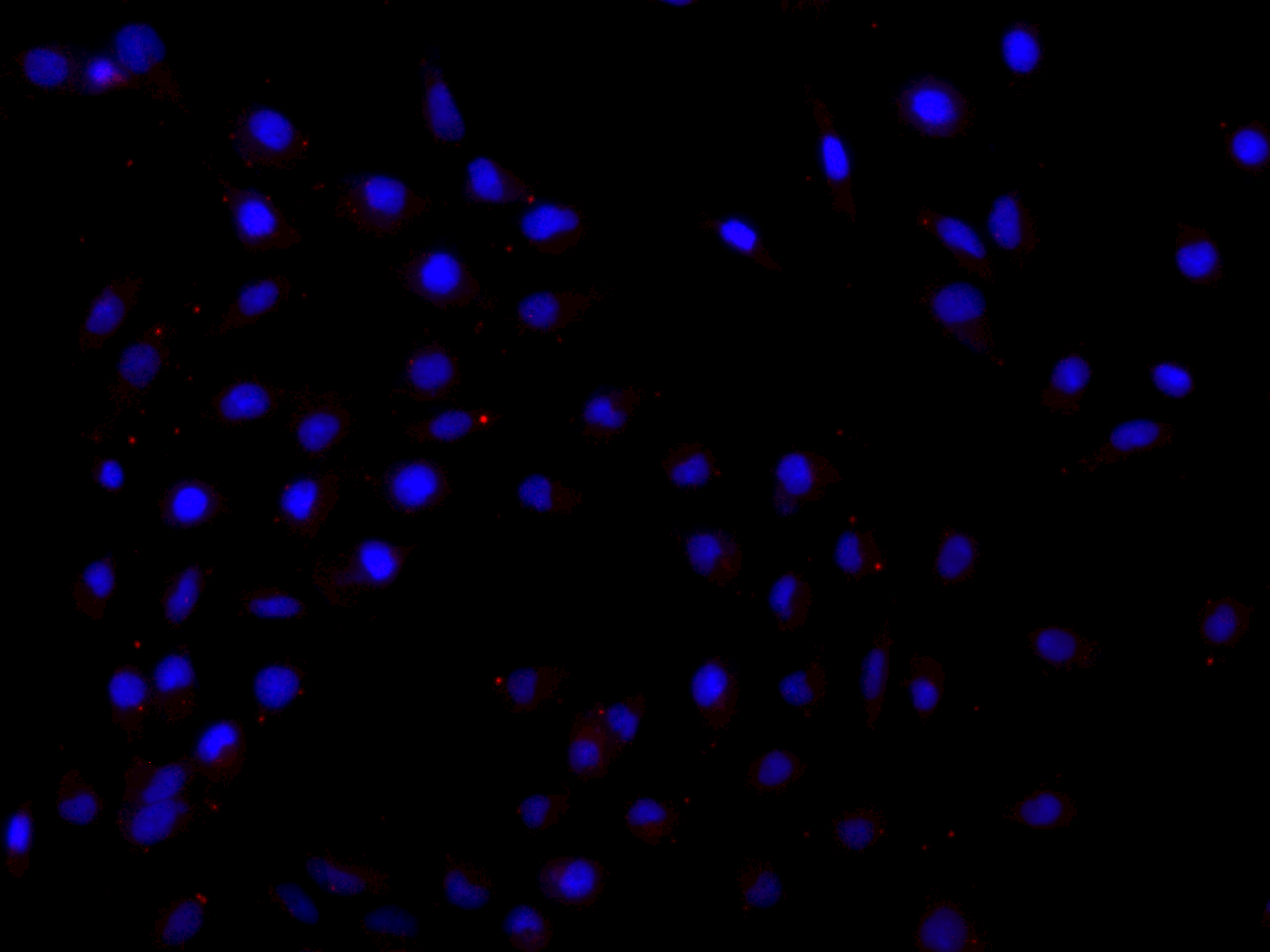

Supplement: Supplementary file 11 [file Data_Sheet_7.ZIP › Fig.5/shCtrl-Ctrl-Merge-a-SMA.tif]

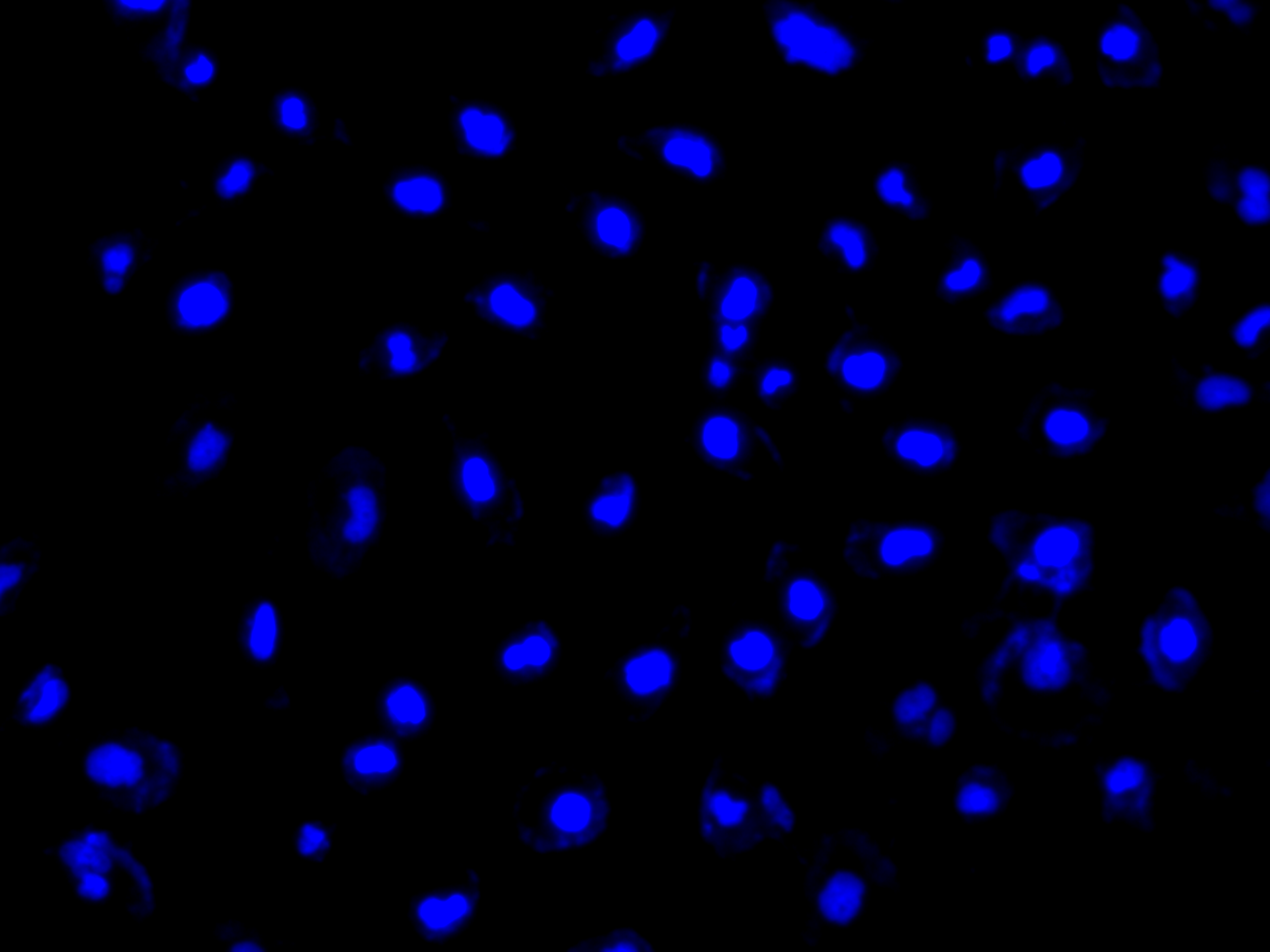

Supplement: Supplementary file 11 [file Data_Sheet_7.ZIP › Fig.5/shJMJD1A-Ctrl-DAPI-a-SMA.tif]

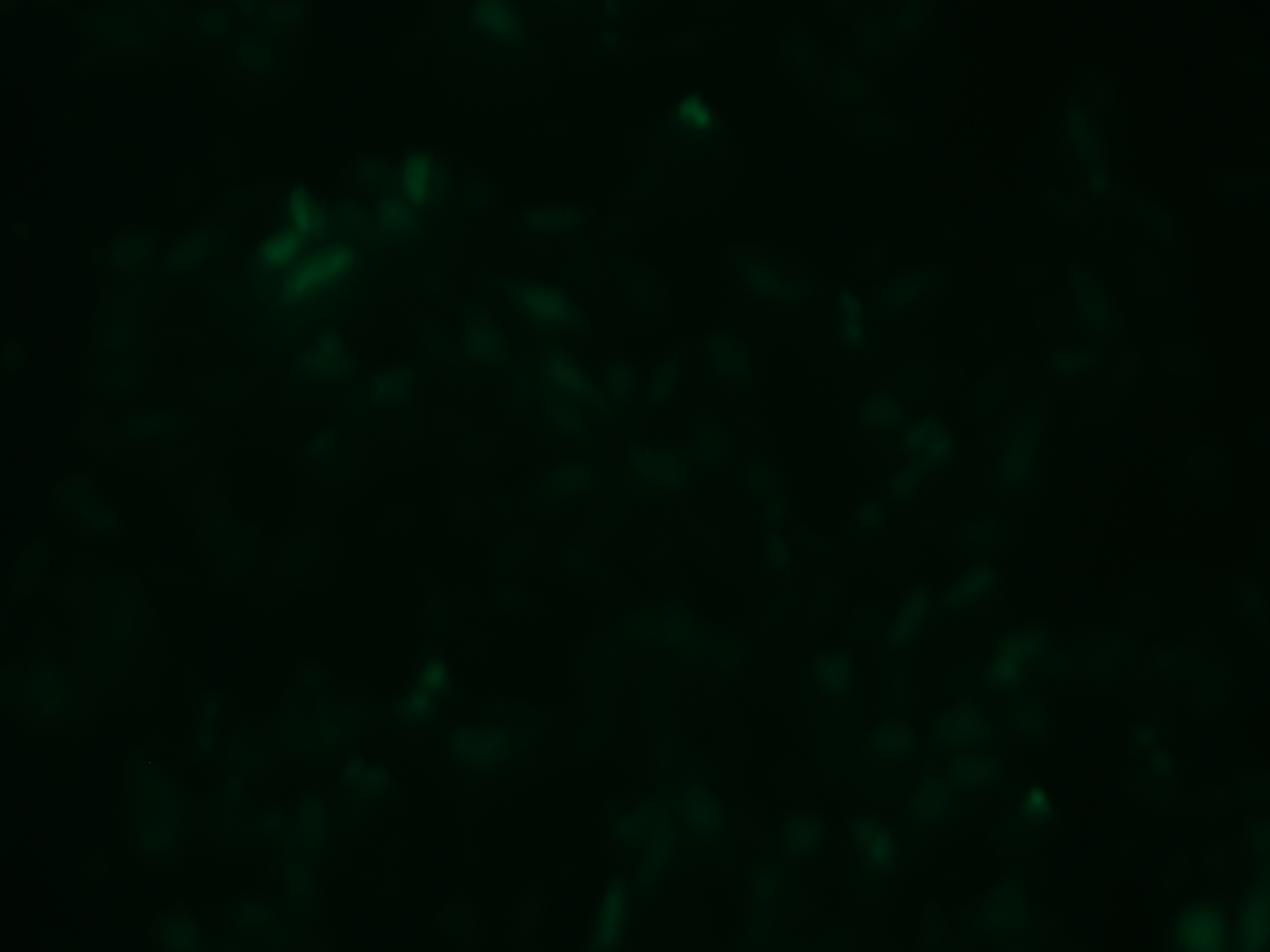

Supplement: Supplementary file 11 [file Data_Sheet_7.ZIP › Fig.5/24h-MOI=30.tif]

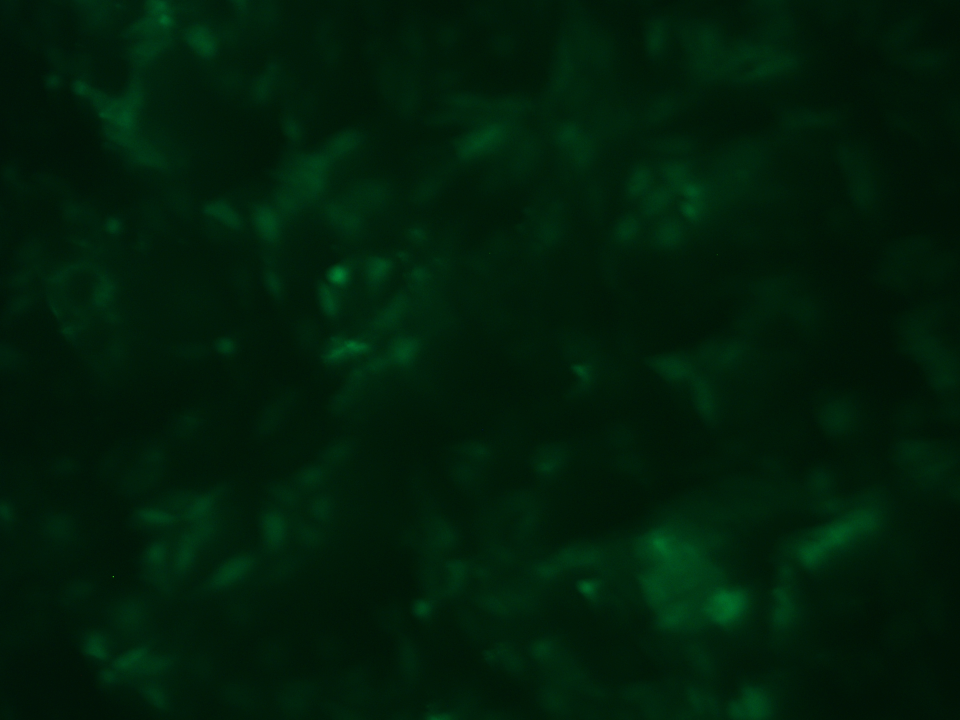

Supplement: Supplementary file 11 [file Data_Sheet_7.ZIP › Fig.5/48h-MOI=30.tif]

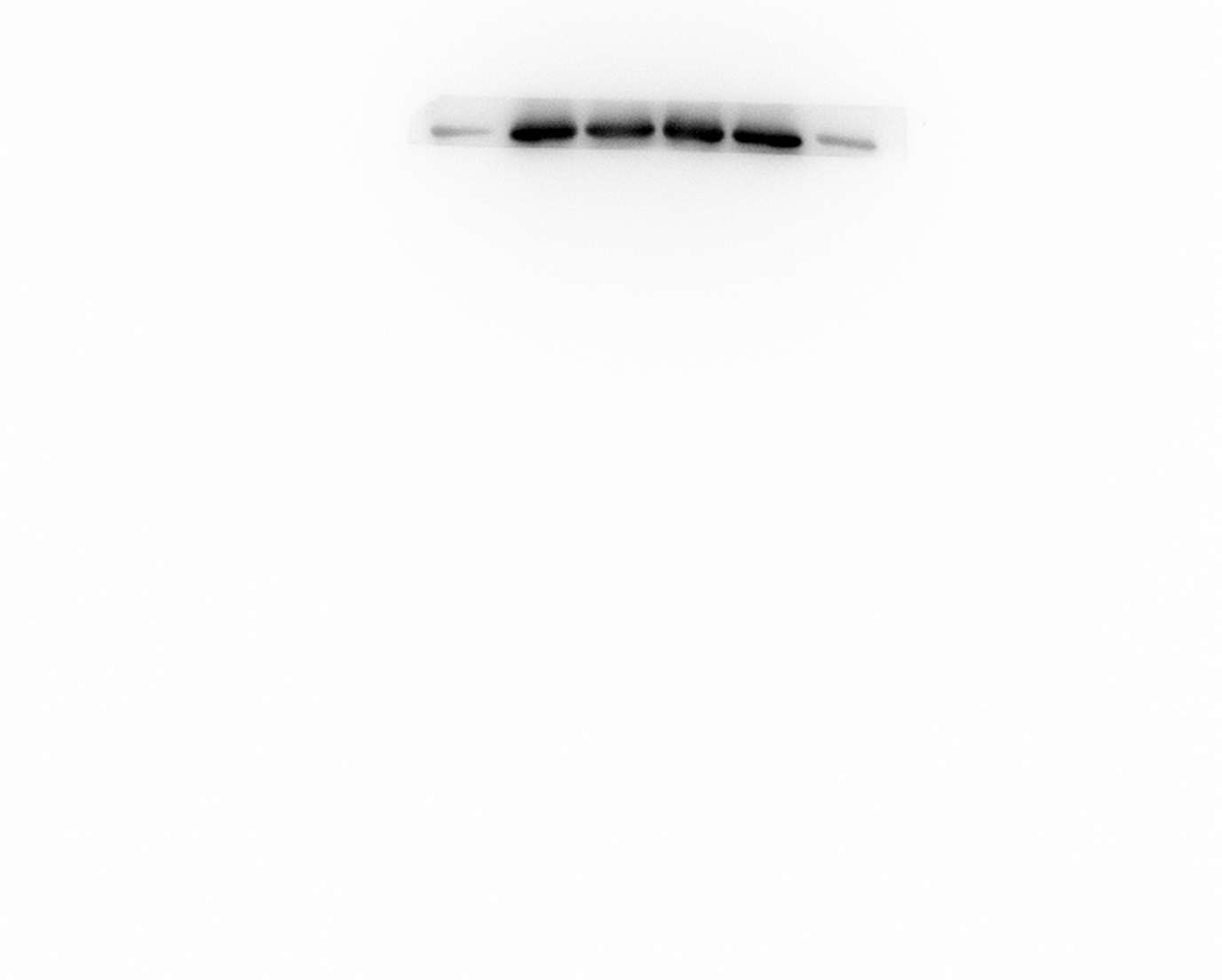

Supplement: Supplementary file 11 [file Data_Sheet_7.ZIP › Fig.5/GAPDH-μò▓.jpg]

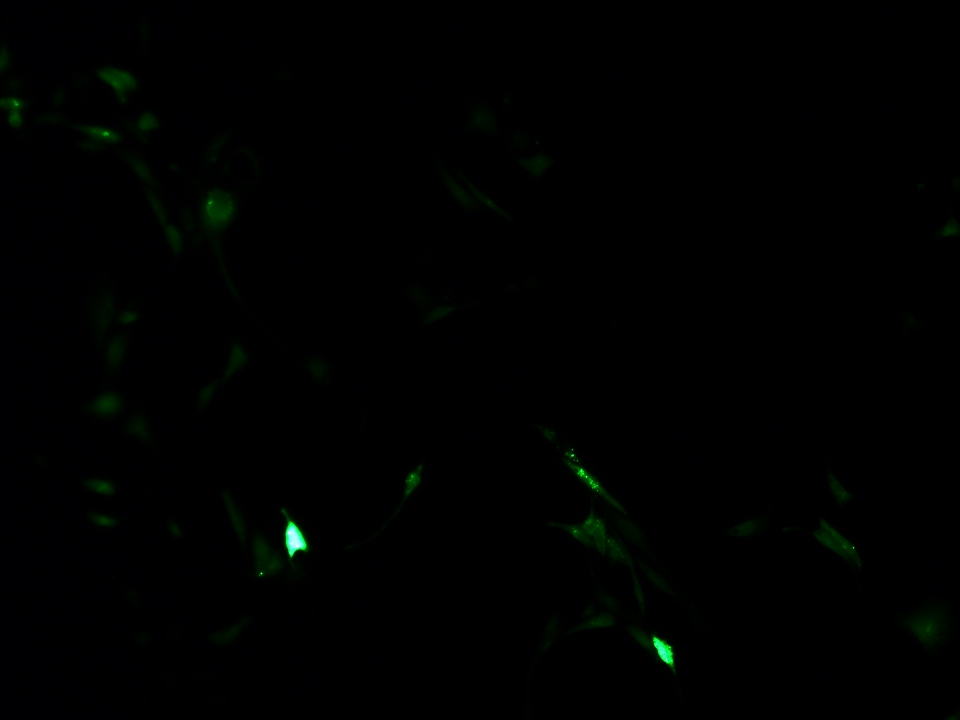

Supplement: Supplementary file 11 [file Data_Sheet_7.ZIP › Fig.5/shctrl-AGEs-GFP-JMJD1A.jpg]

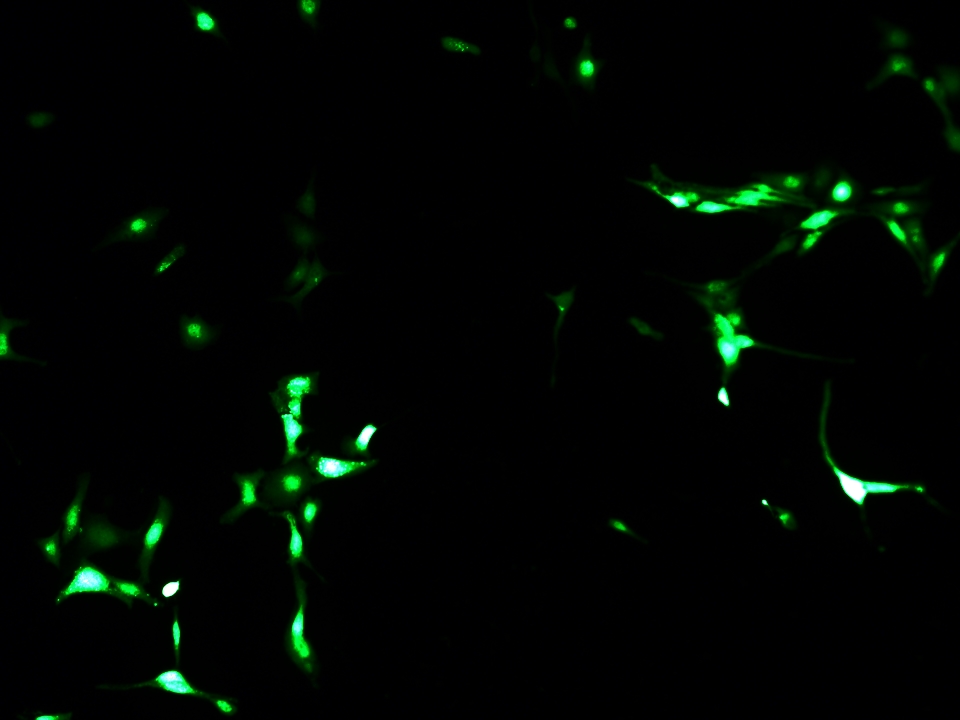

Supplement: Supplementary file 11 [file Data_Sheet_7.ZIP › Fig.5/shJMJD1A-Ctrl-GFP-JMJD1A.jpg]

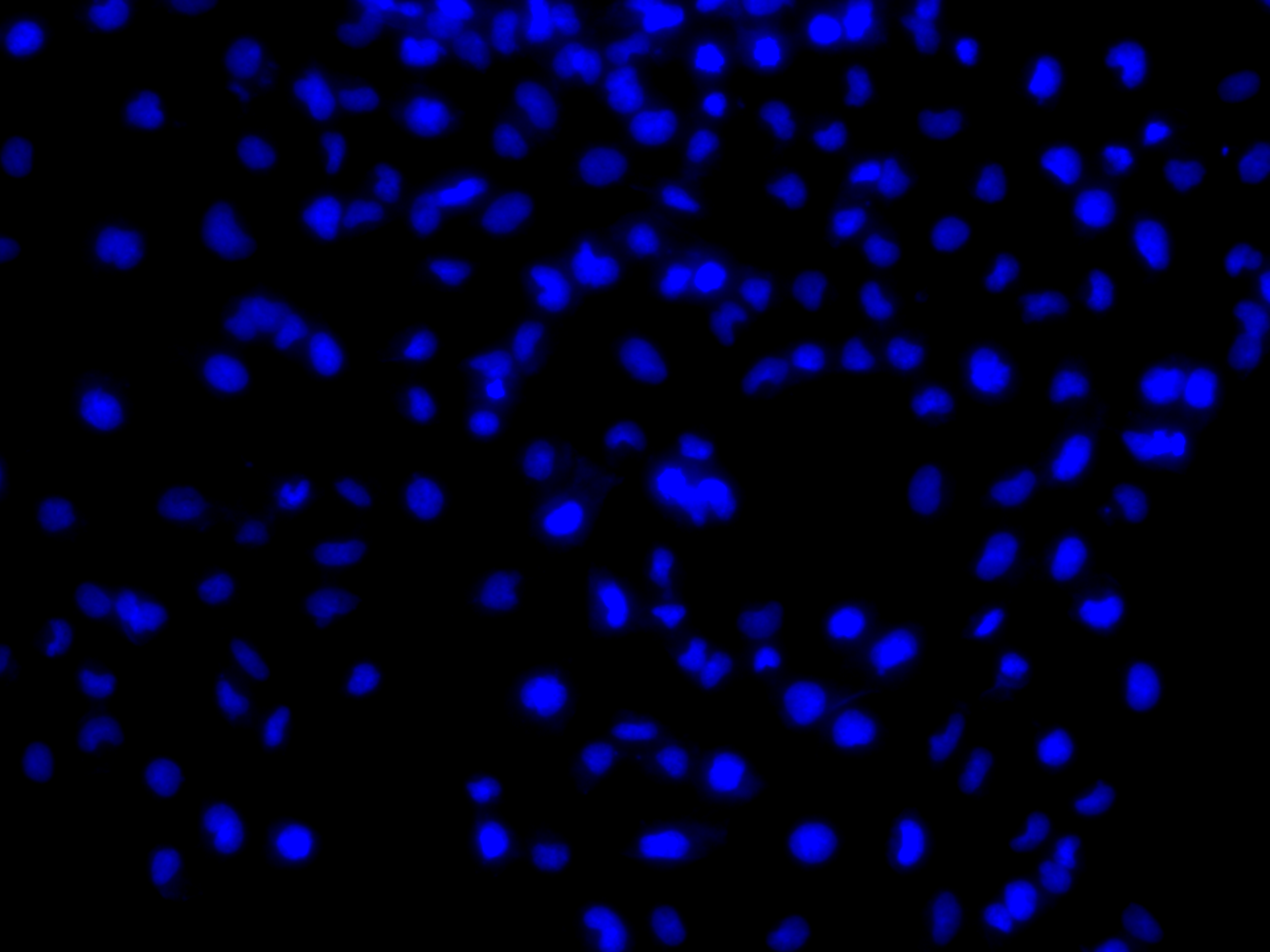

Supplement: Supplementary file 11 [file Data_Sheet_7.ZIP › Fig.5/shCtrl-AGEs-DAPI-a-SMA.tif]

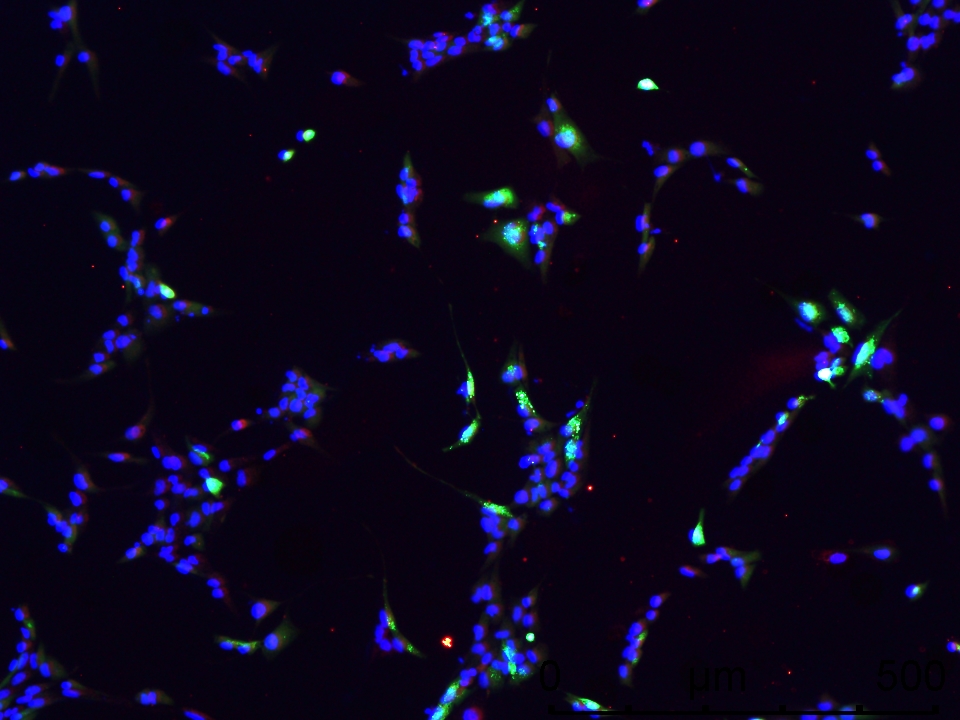

Supplement: Supplementary file 11 [file Data_Sheet_7.ZIP › Fig.5/shCtrl-Ctrl-Merge-JMJD1A.jpg]

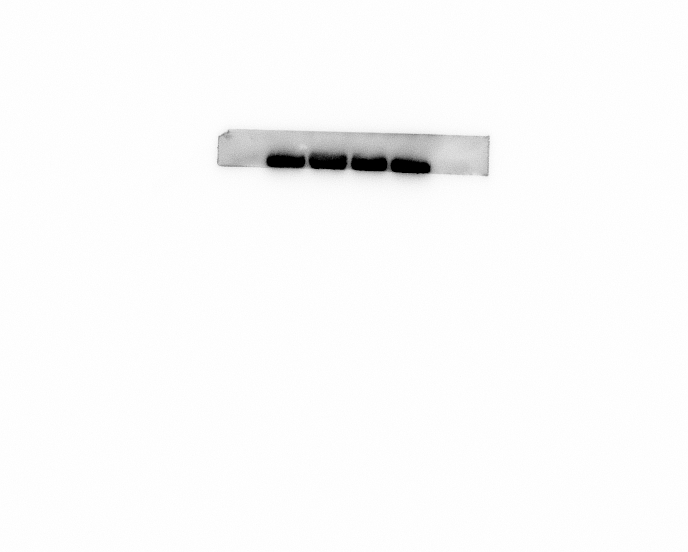

Supplement: Supplementary file 11 [file Data_Sheet_7.ZIP › Fig.5/GAPDH-Q.jpg]

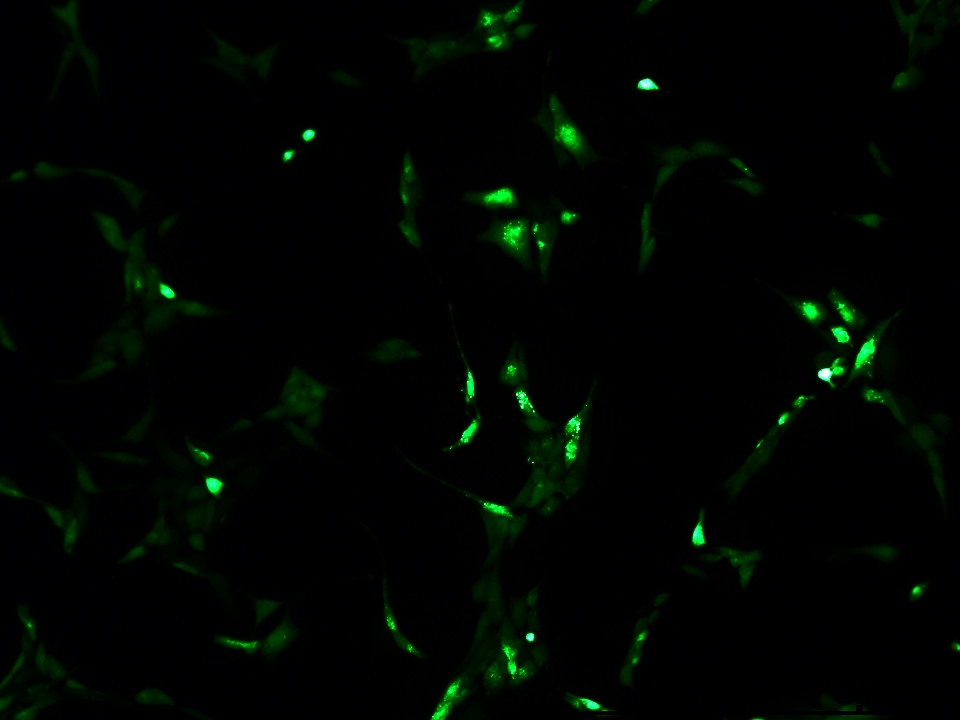

Supplement: Supplementary file 11 [file Data_Sheet_7.ZIP › Fig.5/shCtrl-Ctrl-GFP-JMJD1A.jpg]

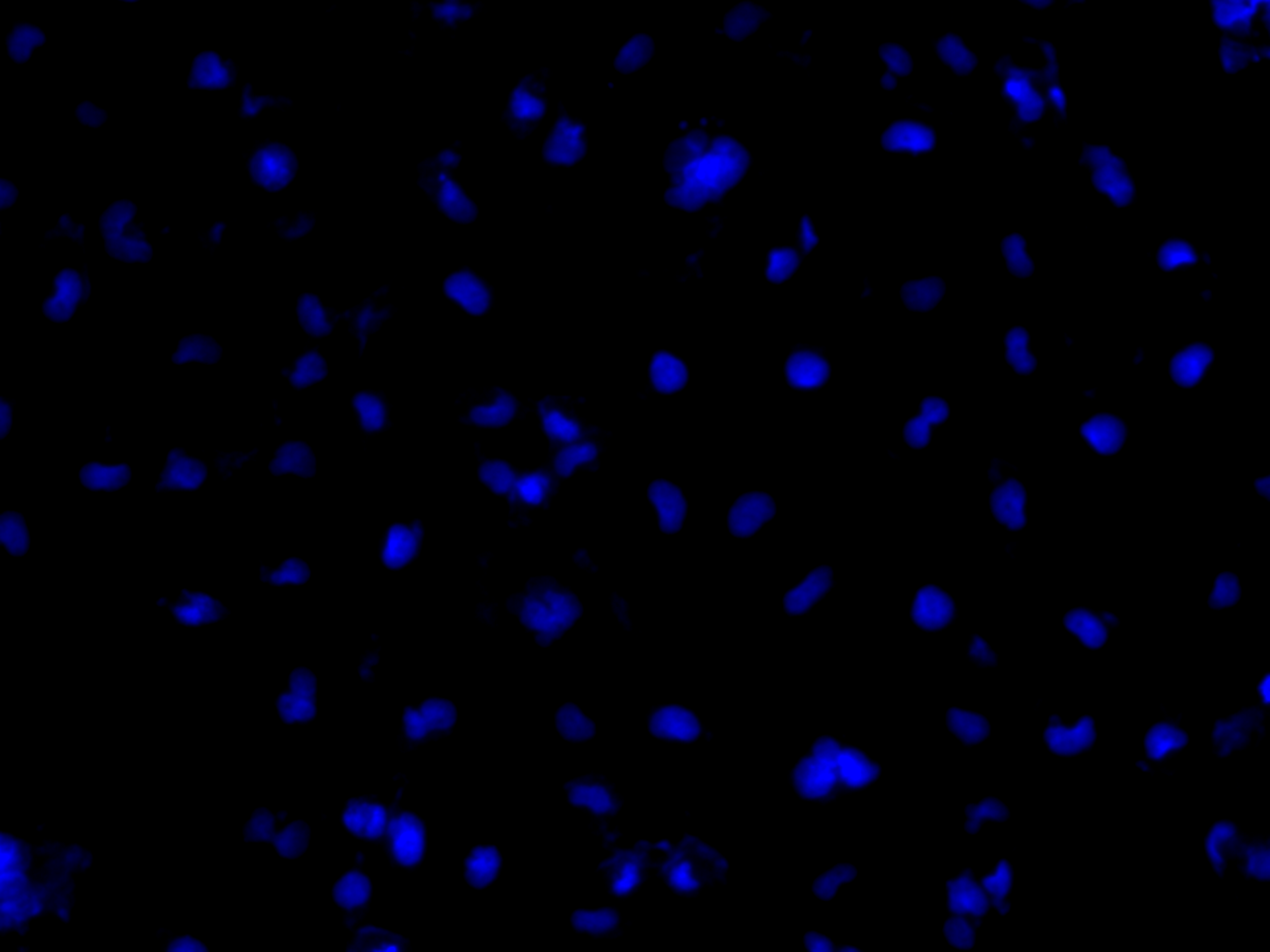

Supplement: Supplementary file 11 [file Data_Sheet_7.ZIP › Fig.5/shJMJD1A-AGEs-DAPI-a-SMA.tif]

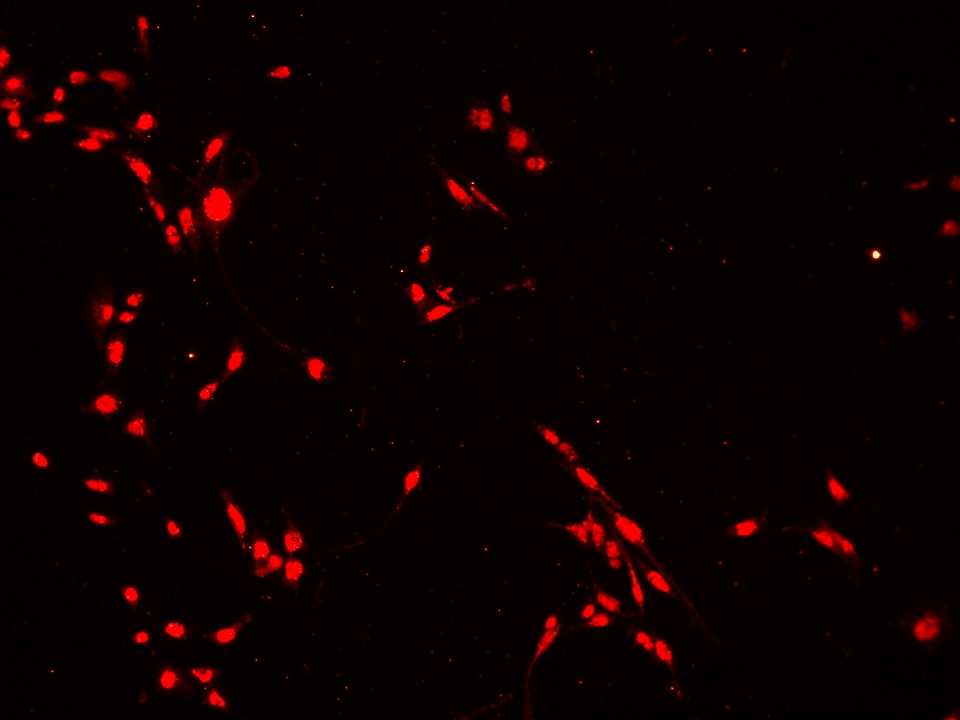

Supplement: Supplementary file 11 [file Data_Sheet_7.ZIP › Fig.5/shctrl-AGEs-JMJD1A.jpg]

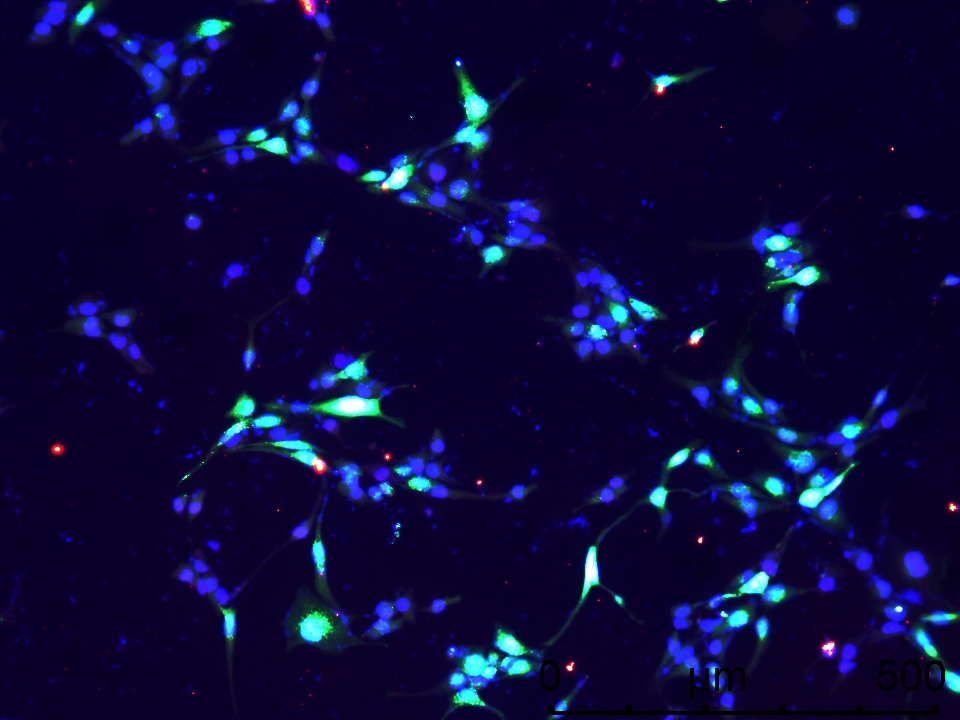

Supplement: Supplementary file 11 [file Data_Sheet_7.ZIP › Fig.5/shJMJD1A-AGEs-Merge-JMJD1A.jpg]

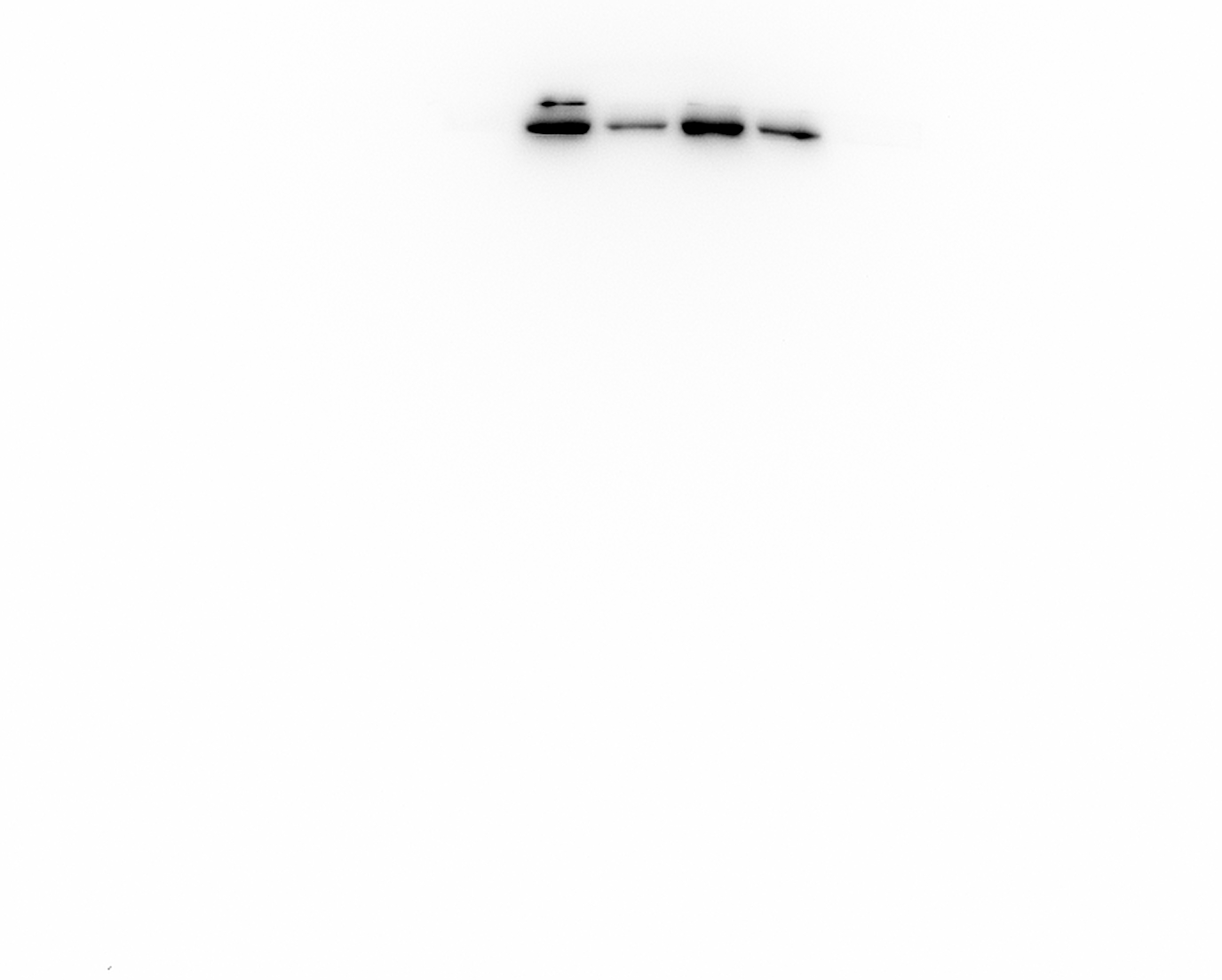

Supplement: Supplementary file 11 [file Data_Sheet_7.ZIP › Fig.5/VIM-2.jpg]

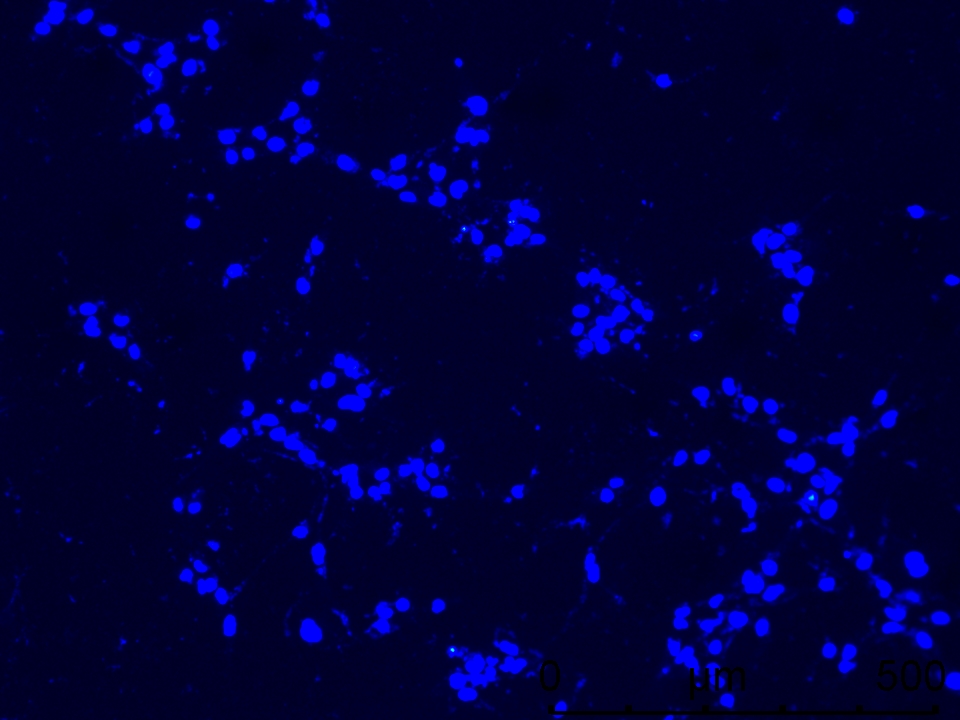

Supplement: Supplementary file 11 [file Data_Sheet_7.ZIP › Fig.5/shJMJD1A-AGEs-DAPI-JMJD1A.jpg]

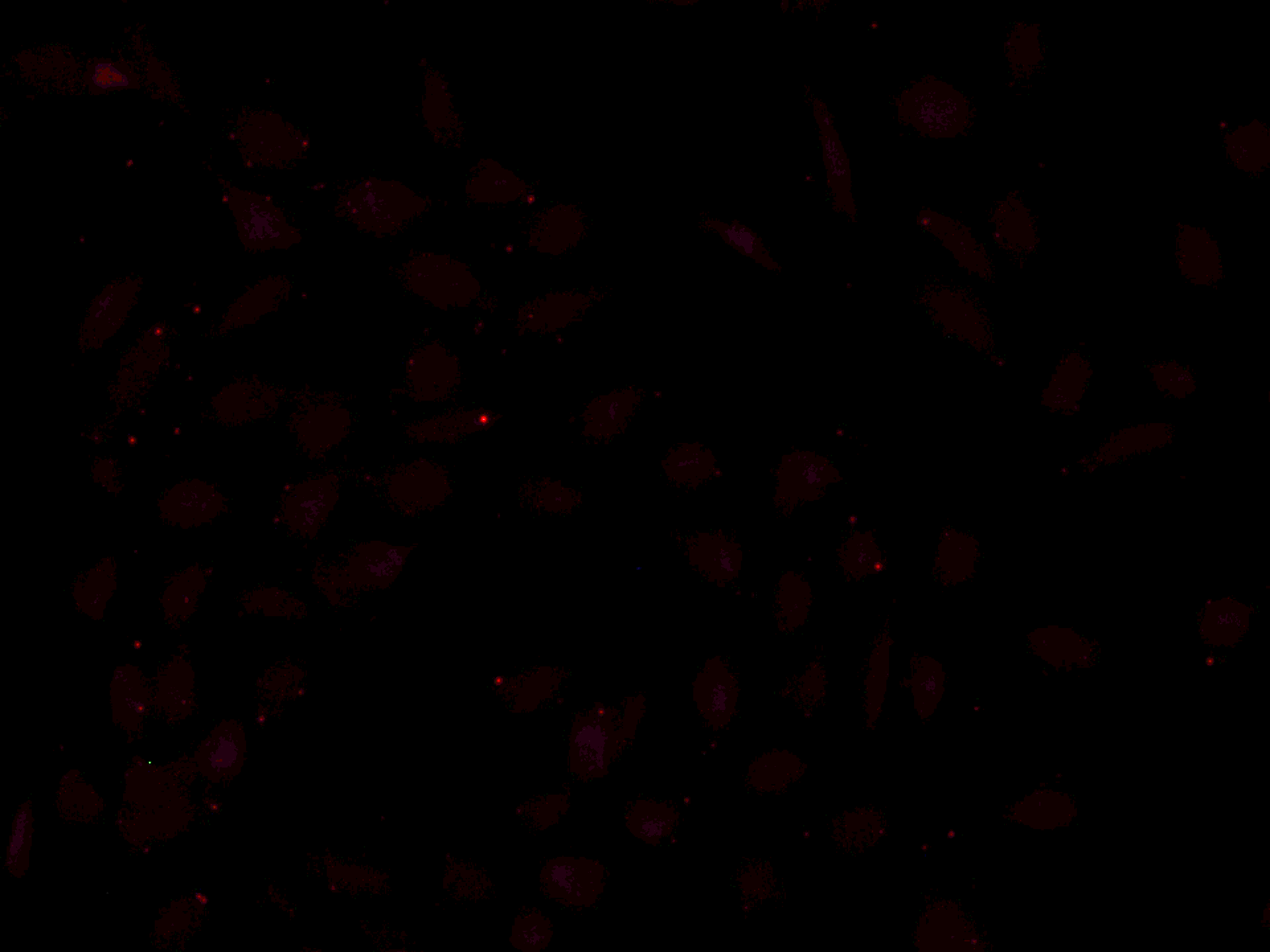

Supplement: Supplementary file 11 [file Data_Sheet_7.ZIP › Fig.5/shCtrl-Ctrl-a-SMA.tif]

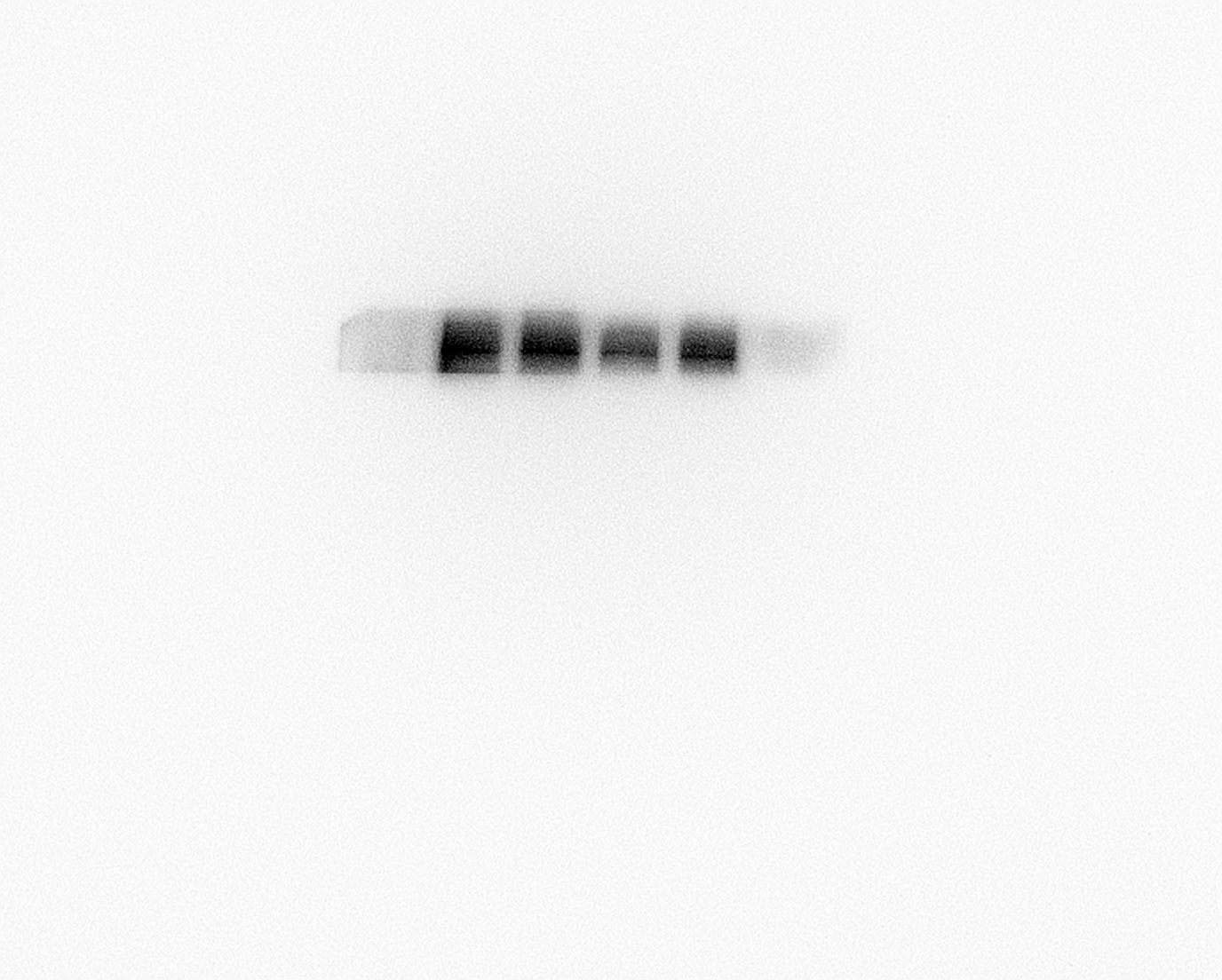

Supplement: Supplementary file 11 [file Data_Sheet_7.ZIP › Fig.5/COL-1-μò▓2.jpg]

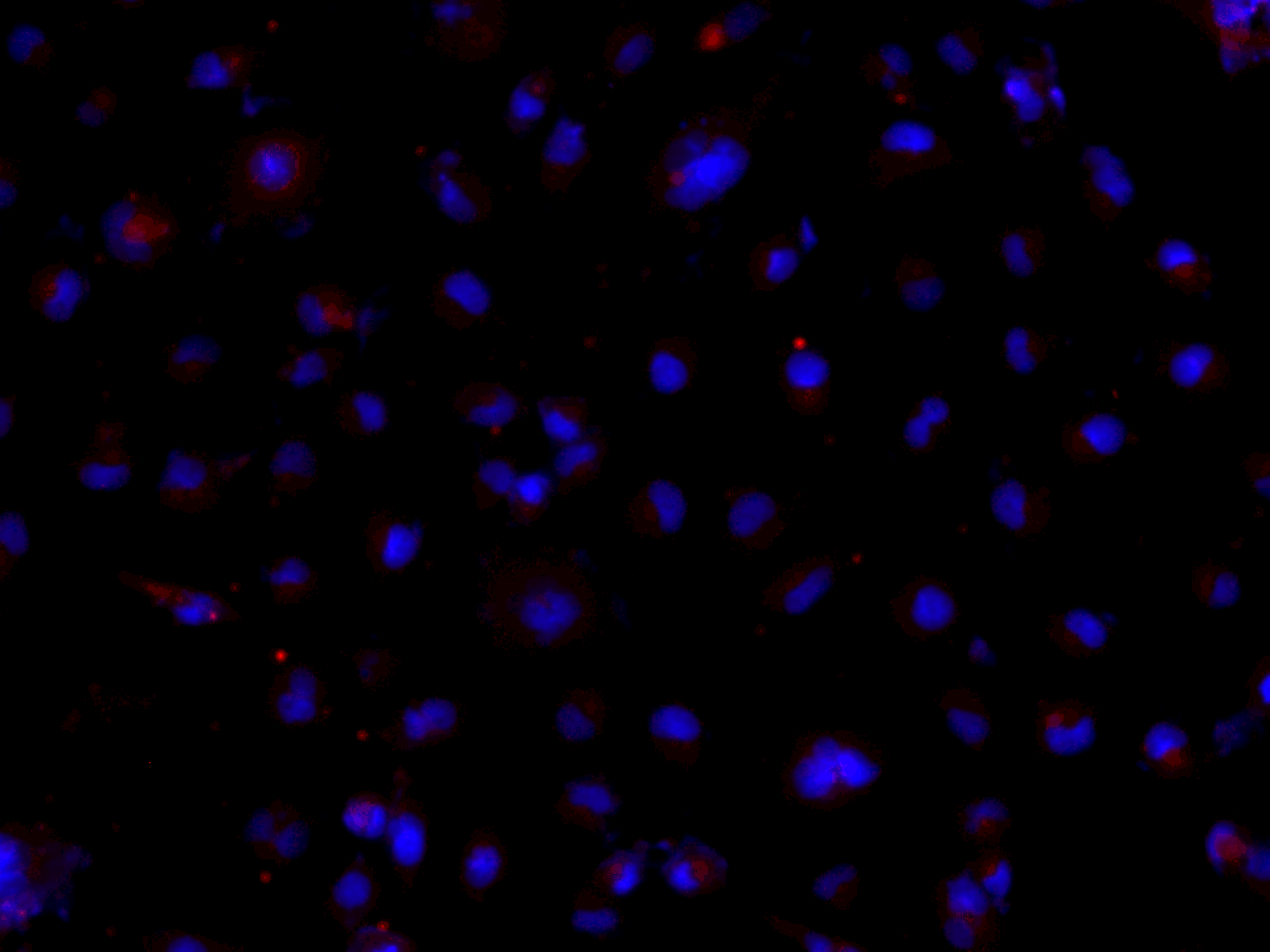

Supplement: Supplementary file 11 [file Data_Sheet_7.ZIP › Fig.5/shJMJD1A-AGEs-Merge-a-SMA.tif]

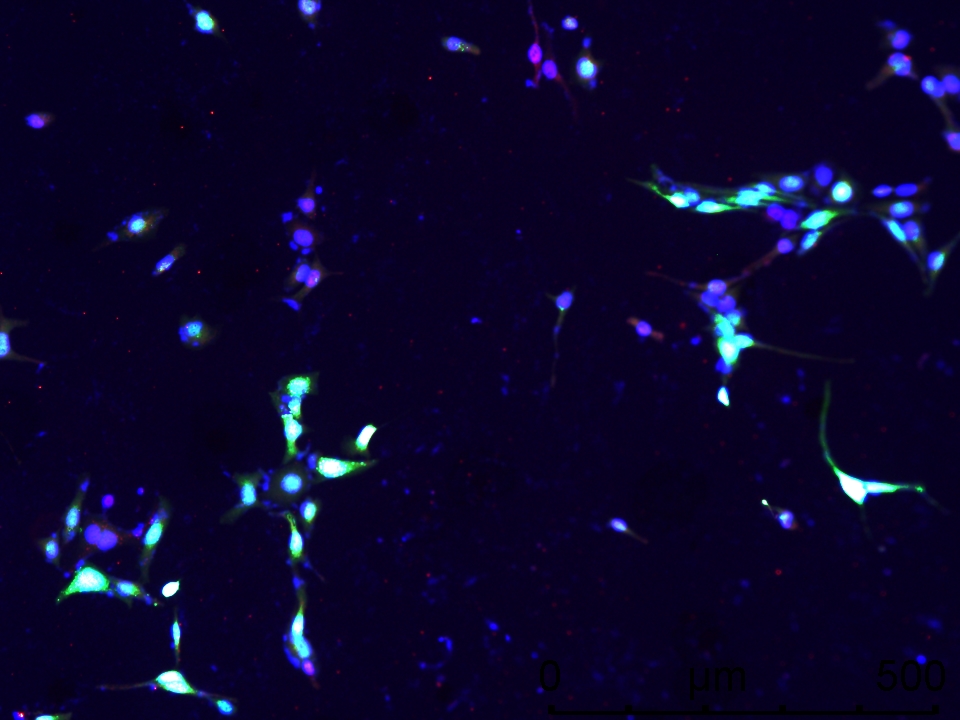

Supplement: Supplementary file 11 [file Data_Sheet_7.ZIP › Fig.5/shJMJD1A-Ctrl-Merge-JMJD1A.jpg]

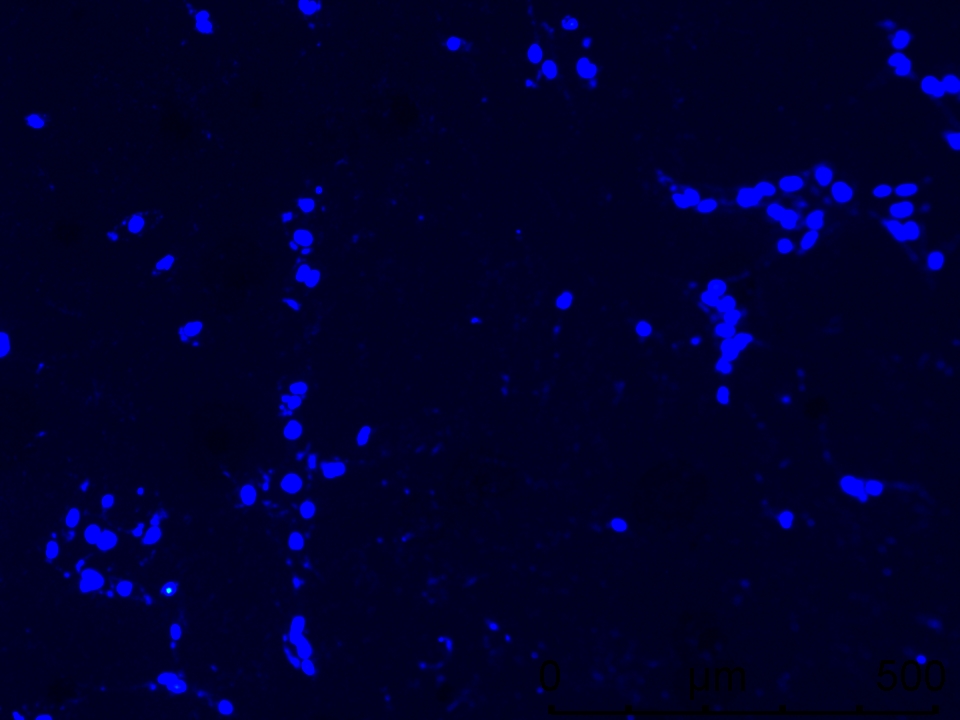

Supplement: Supplementary file 11 [file Data_Sheet_7.ZIP › Fig.5/shJMJD1A-Ctrl-DAPI-JMJD1A.jpg]

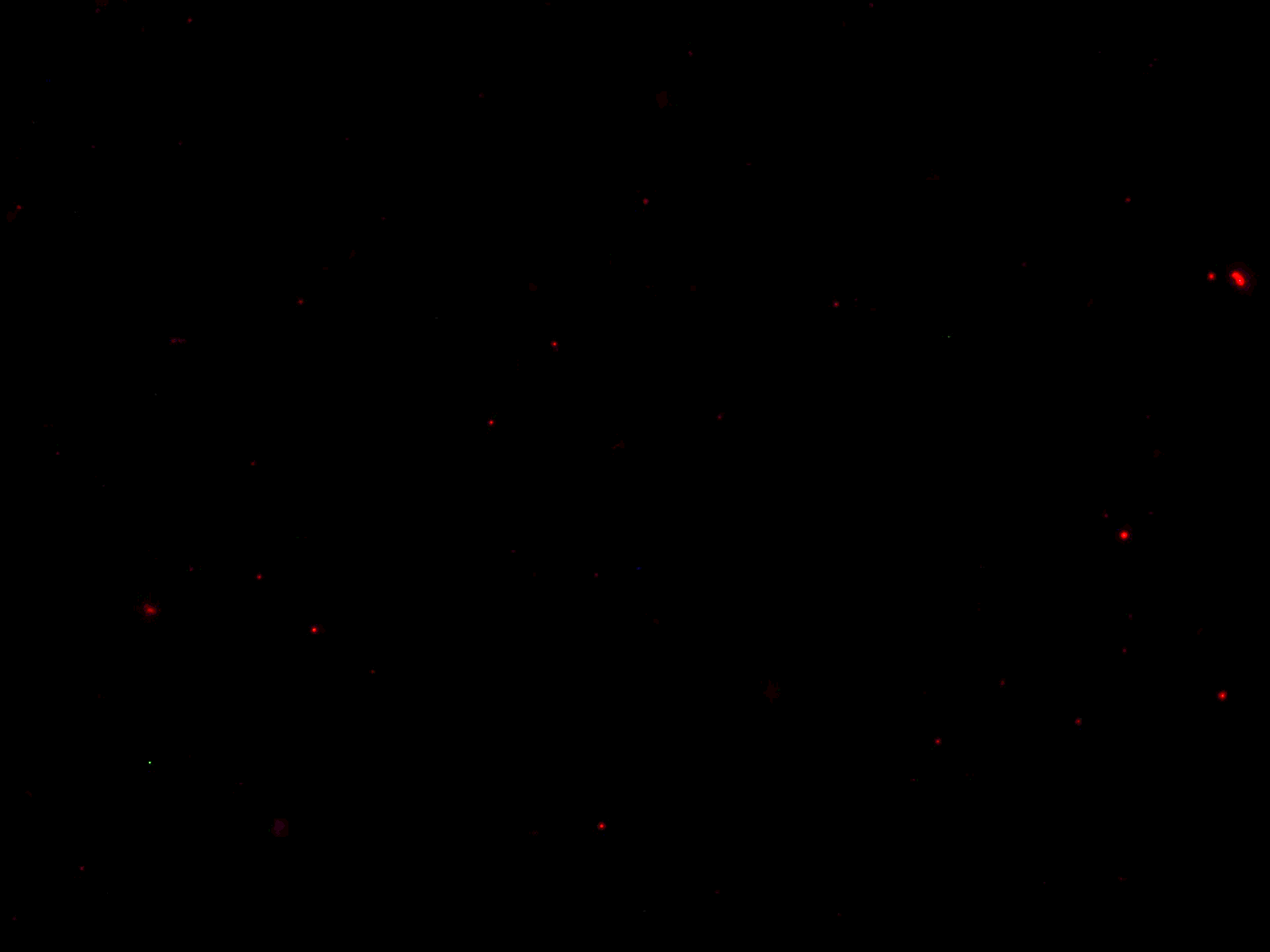

Supplement: Supplementary file 11 [file Data_Sheet_7.ZIP › Fig.5/shCtrl-AGEs-a-SMA.tif]

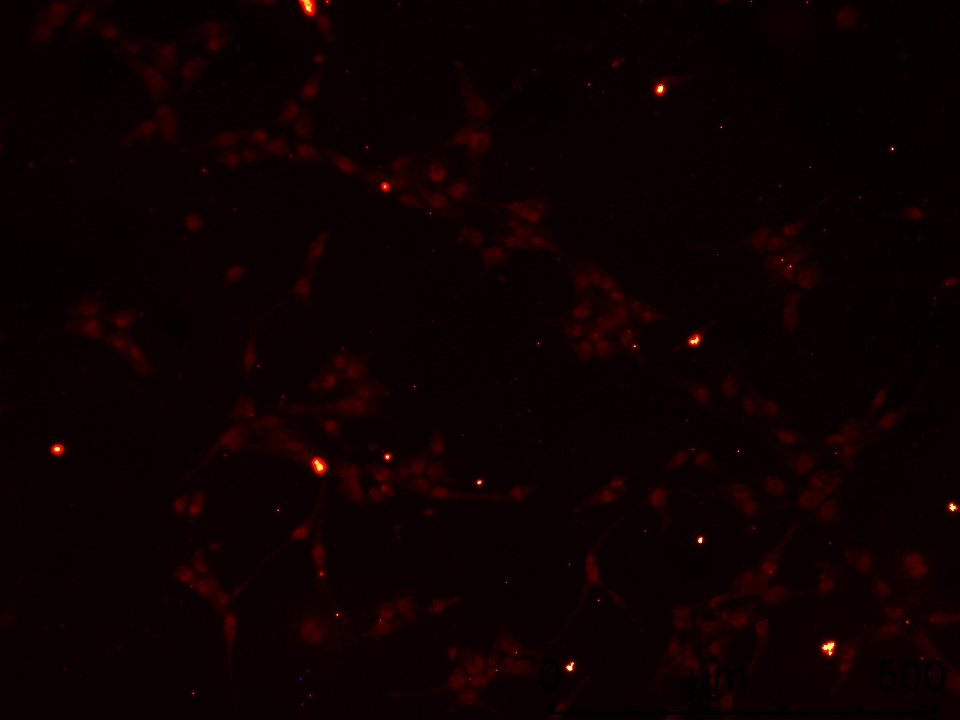

Supplement: Supplementary file 11 [file Data_Sheet_7.ZIP › Fig.5/shJMJD1A-AGEs-JMJD1A.jpg]

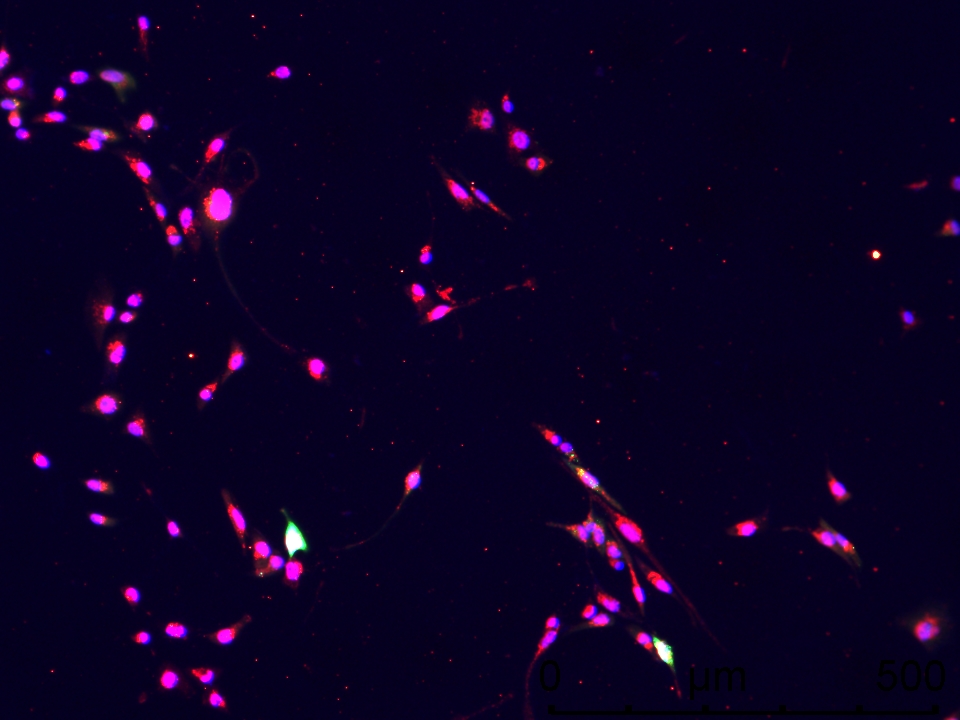

Supplement: Supplementary file 11 [file Data_Sheet_7.ZIP › Fig.5/shctrl-AGEs-Merge-JMJD1A.jpg]

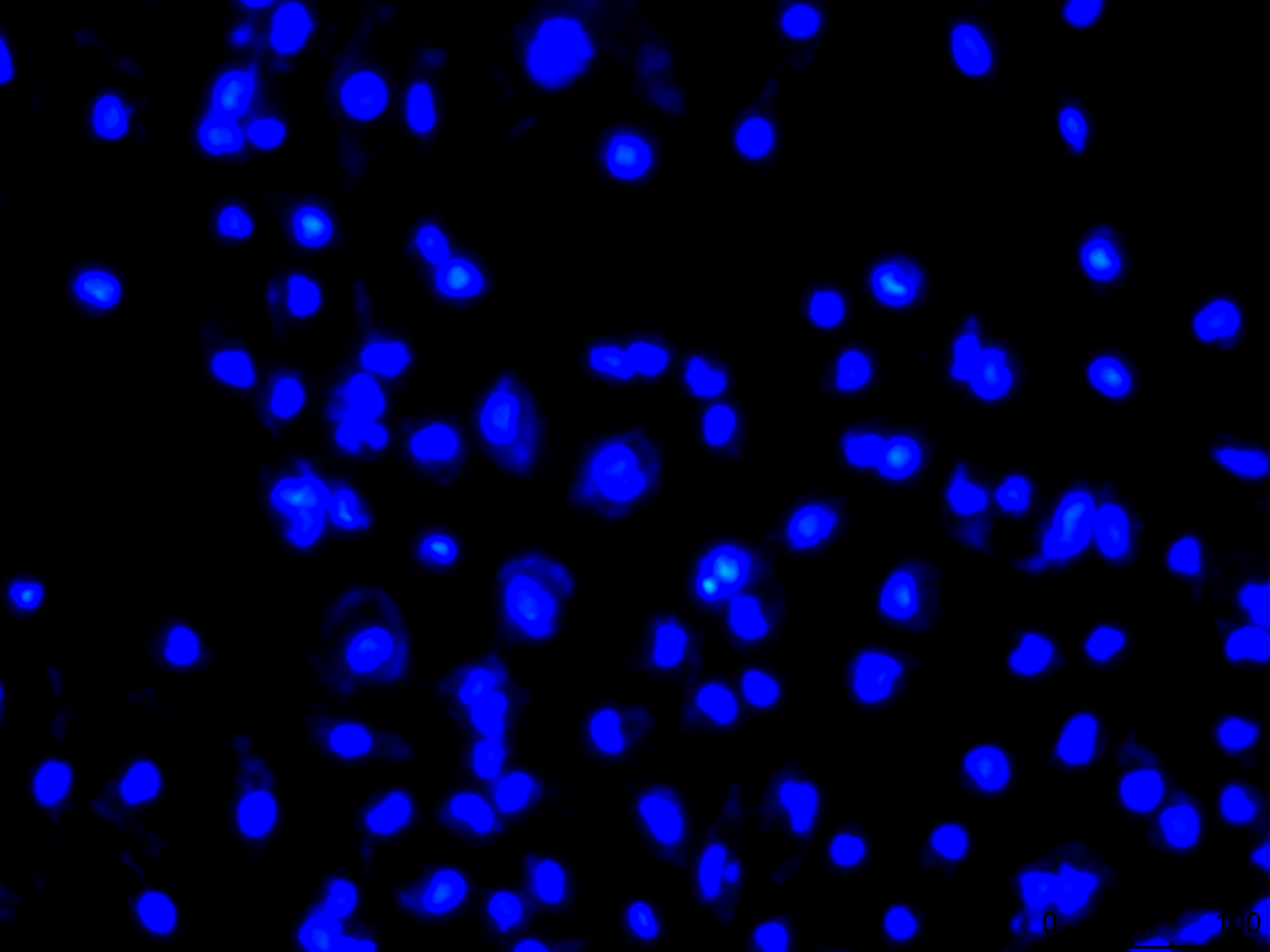

Supplement: Supplementary file 12 [file Data_Sheet_8.ZIP › Fig.6/vector group-Ctrl-DAPI-JMJD1A.tif]

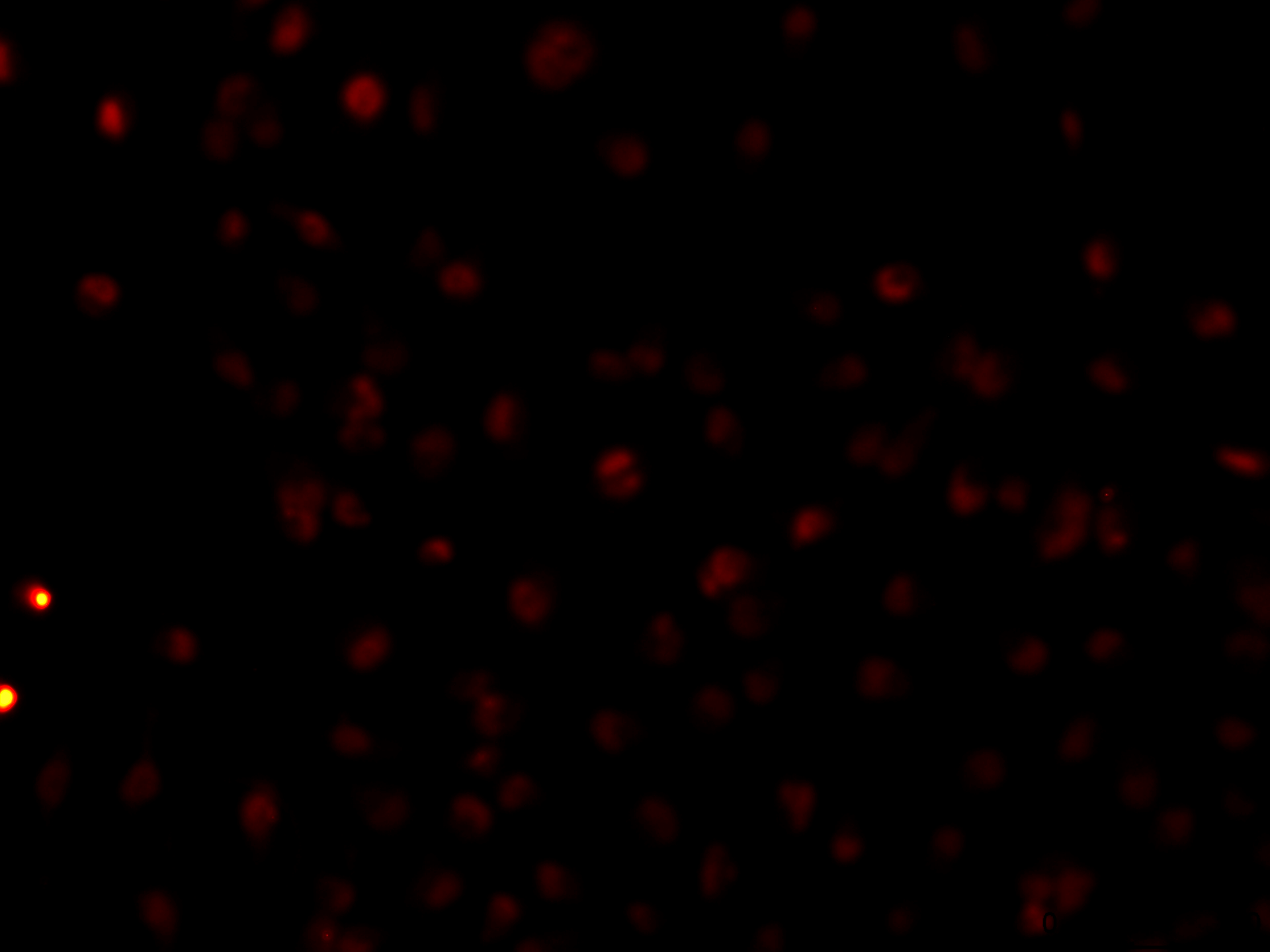

Supplement: Supplementary file 12 [file Data_Sheet_8.ZIP › Fig.6/vector group-Ctrl-JMJD1A.tif]

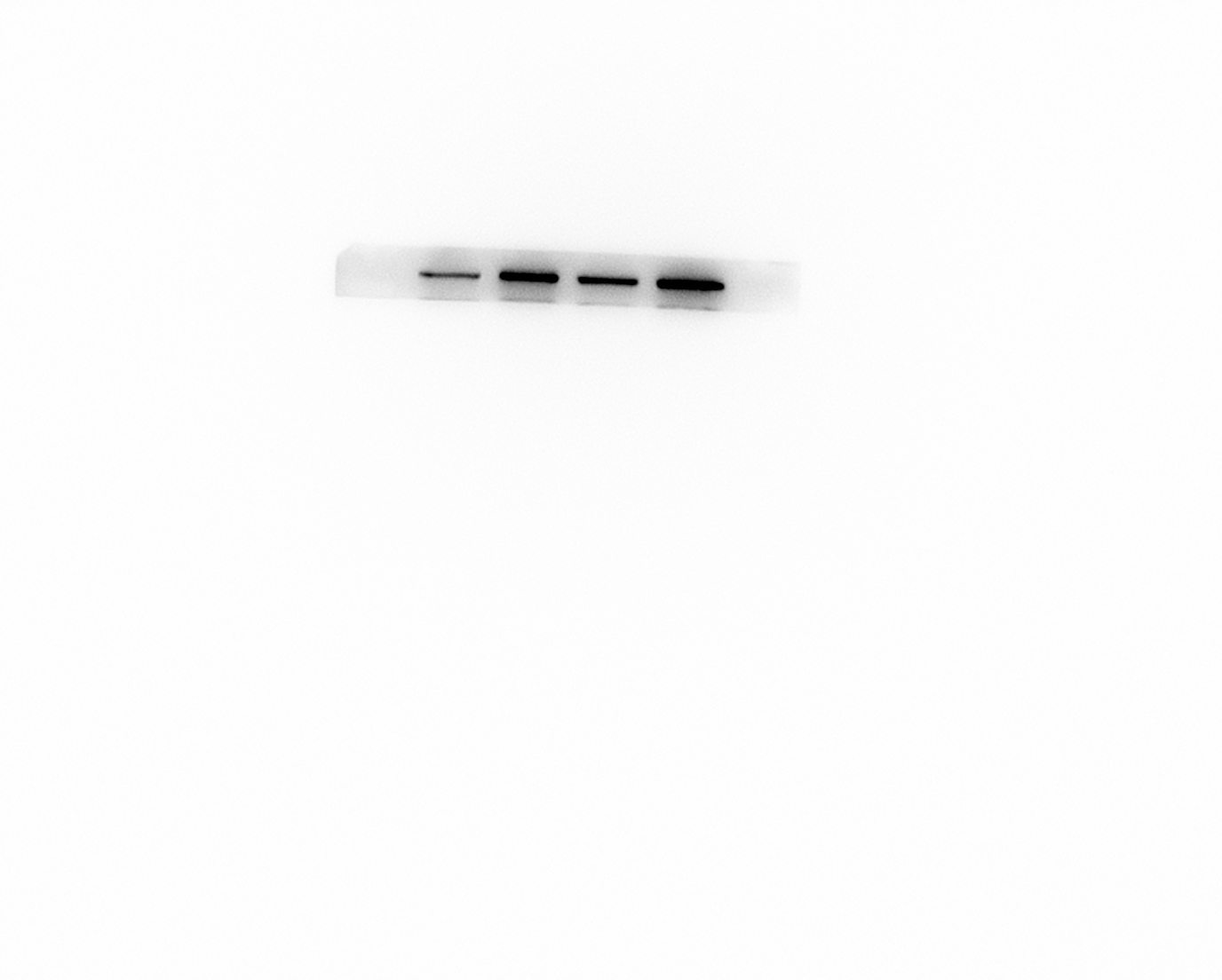

Supplement: Supplementary file 12 [file Data_Sheet_8.ZIP › Fig.6/VIM.jpg]

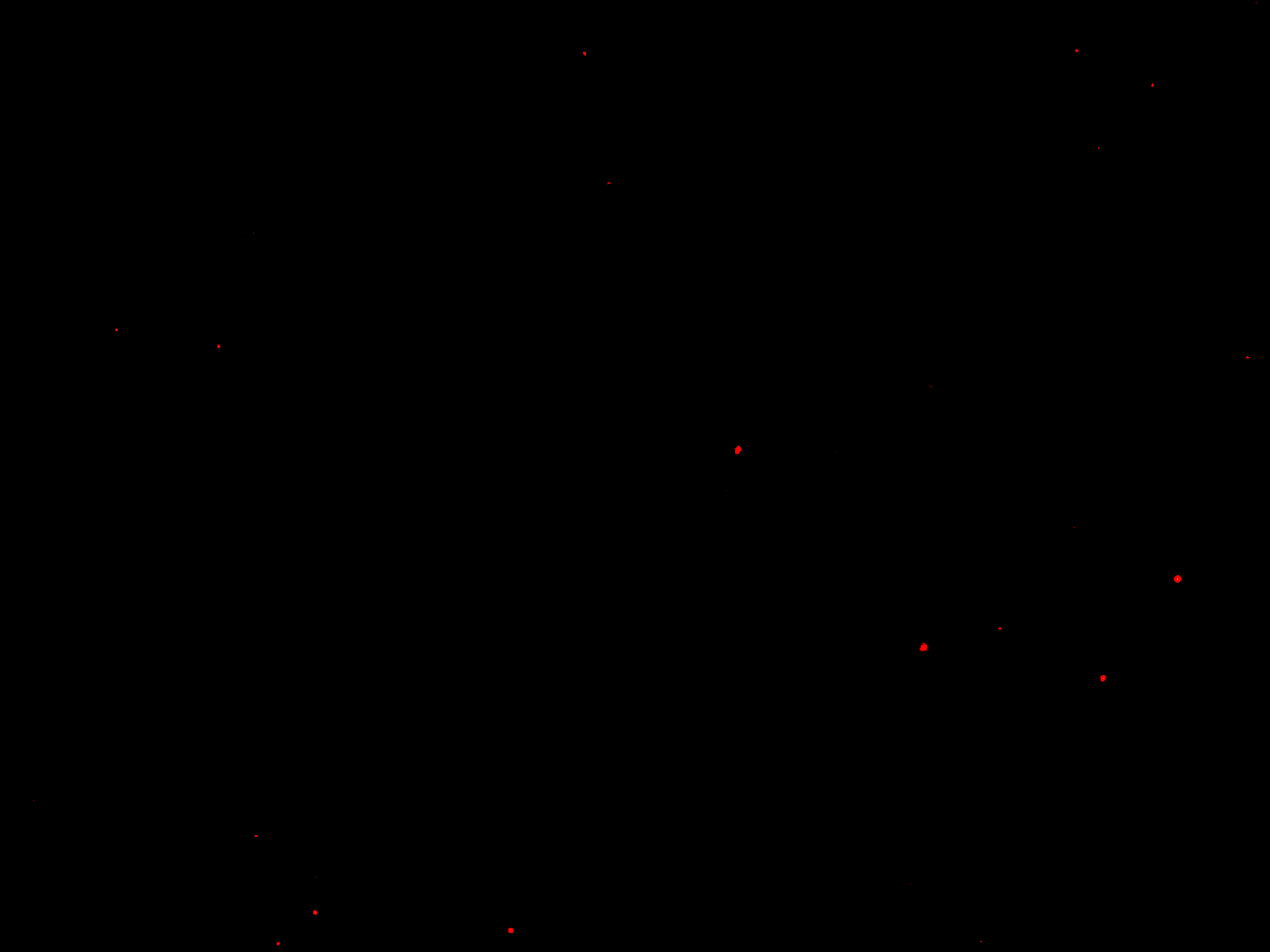

Supplement: Supplementary file 12 [file Data_Sheet_8.ZIP › Fig.6/vector group-Ctrl-a-SMA.tif]

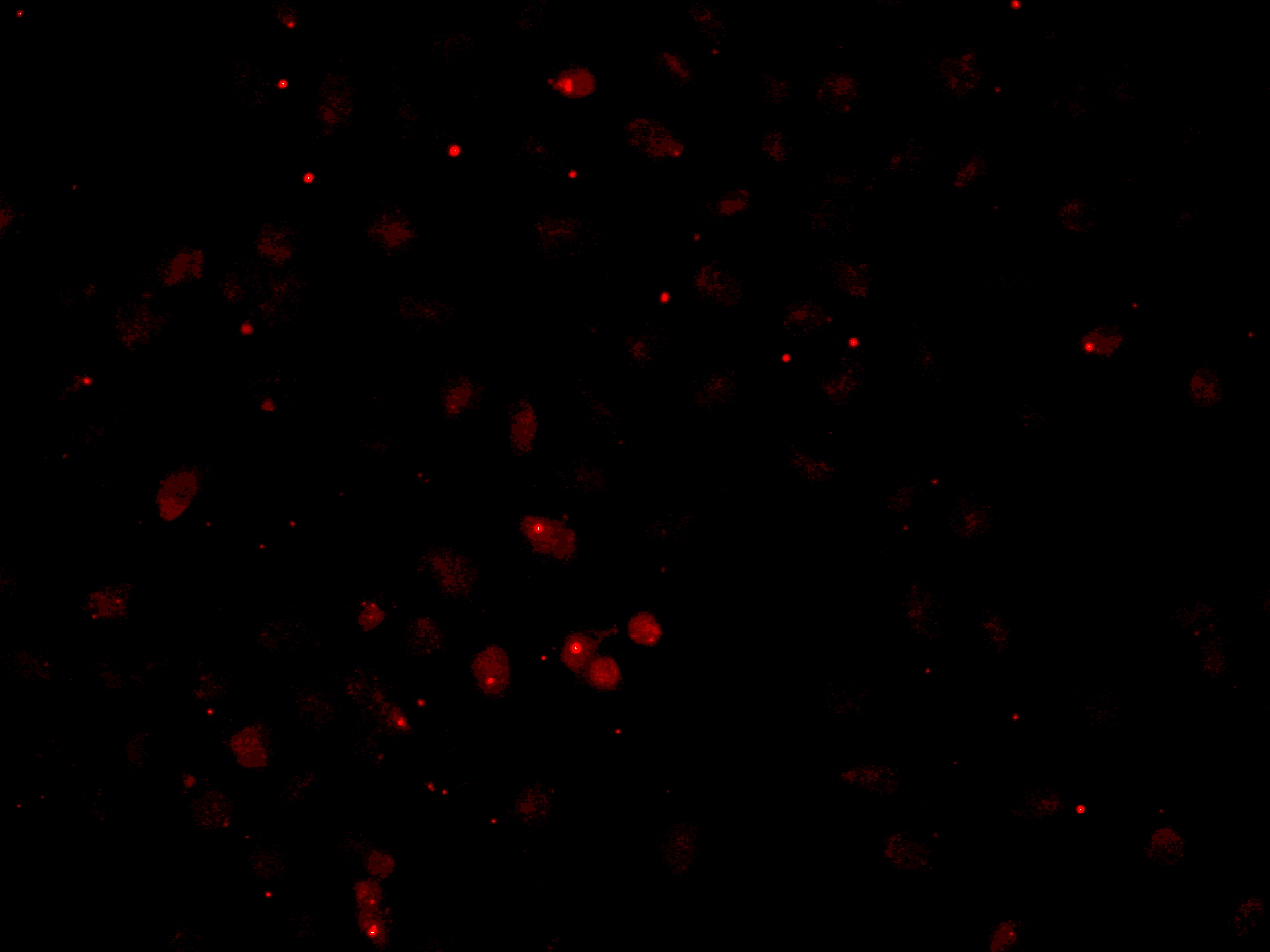

Supplement: Supplementary file 12 [file Data_Sheet_8.ZIP › Fig.6/vector group-AGEs-a-SMA.tif]

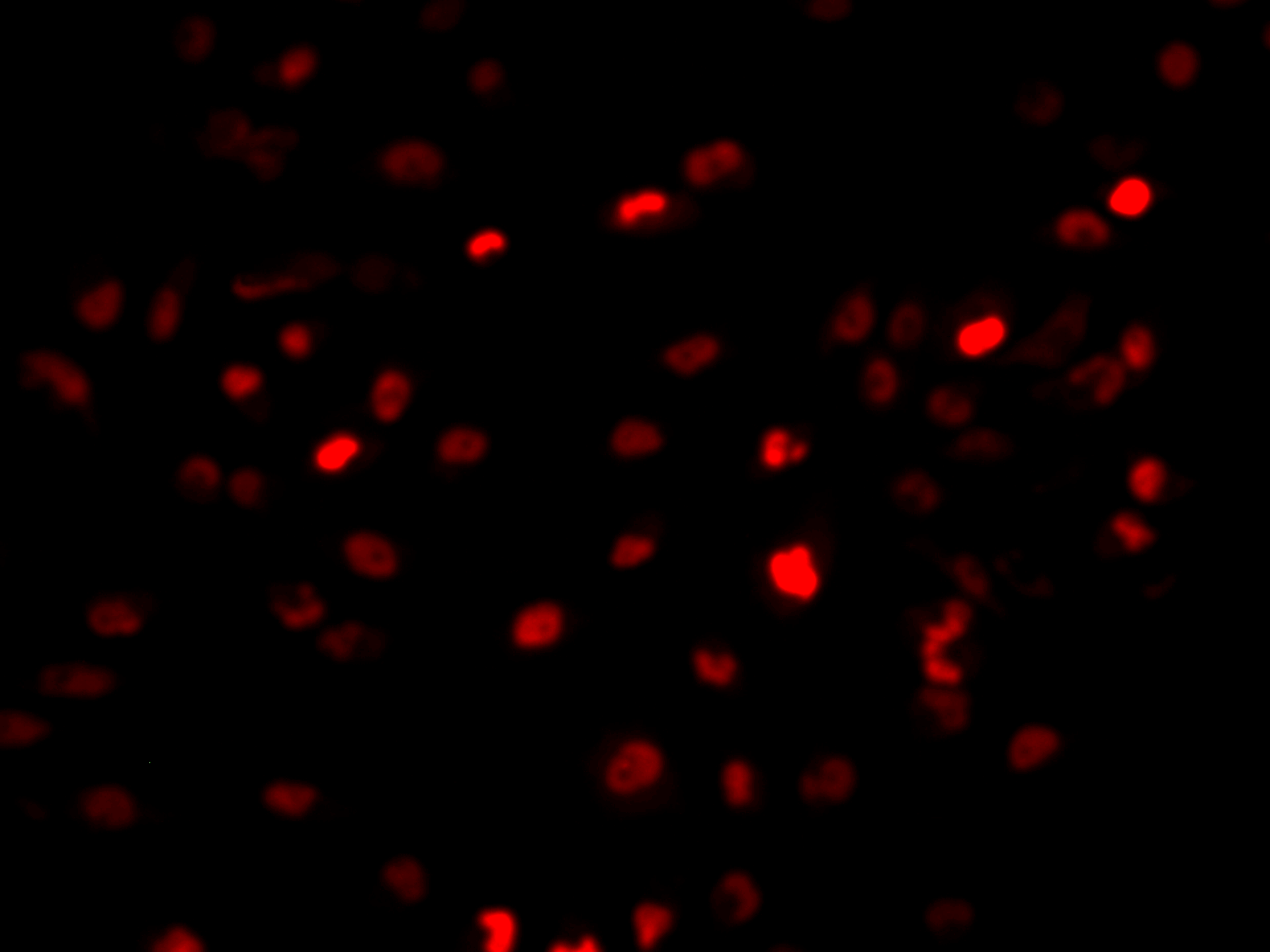

Supplement: Supplementary file 12 [file Data_Sheet_8.ZIP › Fig.6/JMJD1A group-AGEs-JMJD1A.tif]

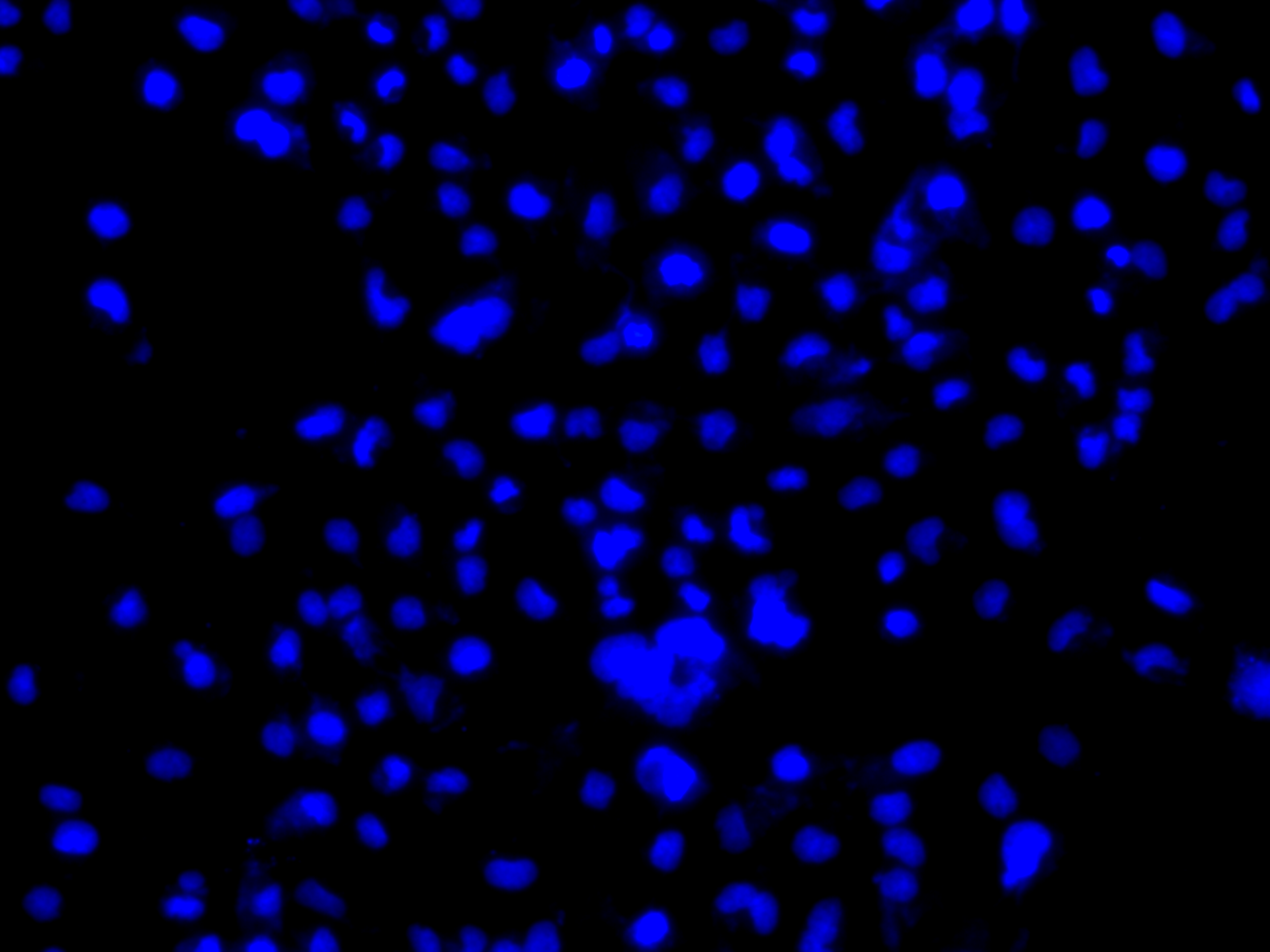

Supplement: Supplementary file 12 [file Data_Sheet_8.ZIP › Fig.6/vector group-AGEs-DAPI-JMJD1A.tif]

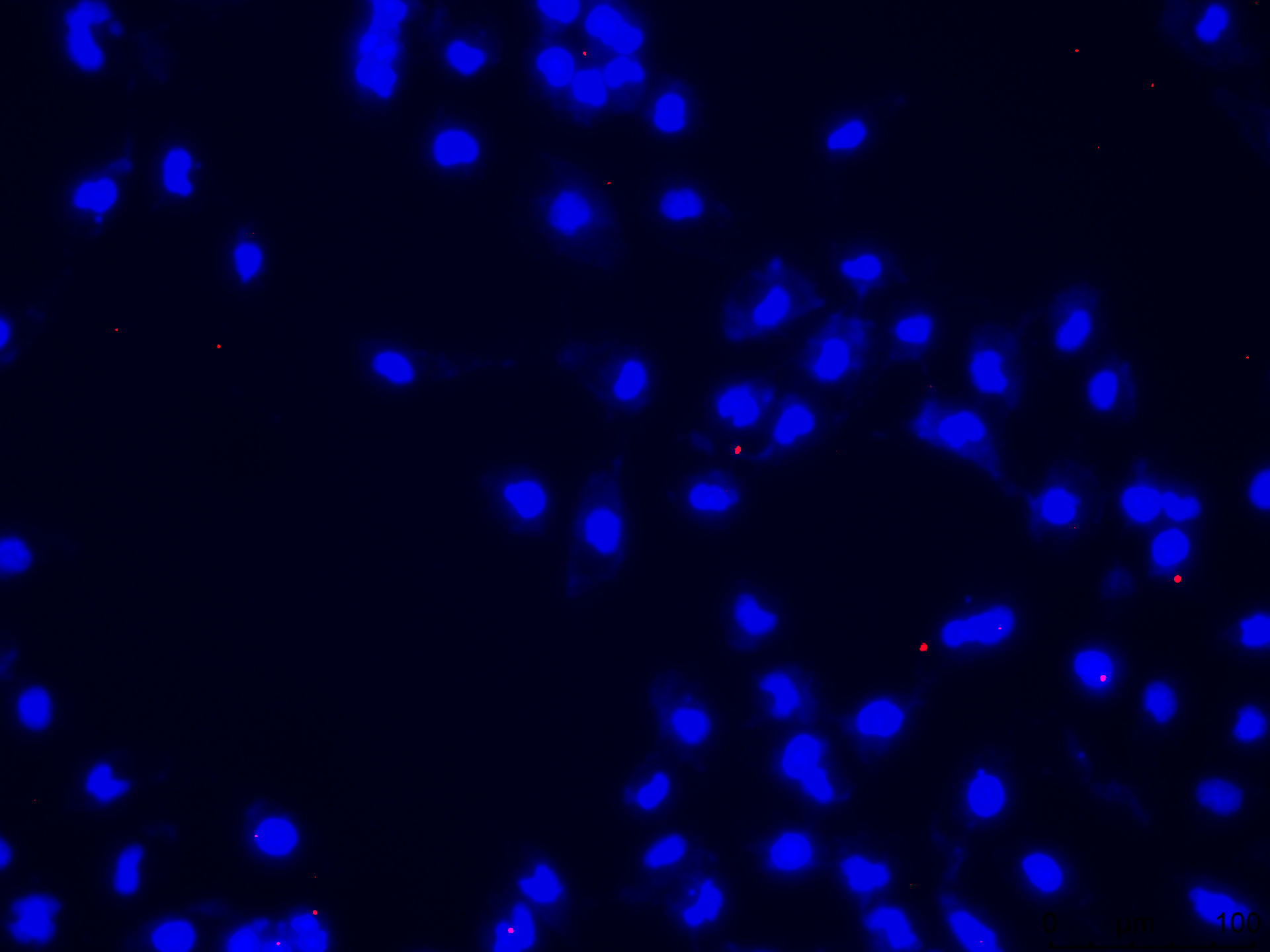

Supplement: Supplementary file 12 [file Data_Sheet_8.ZIP › Fig.6/vector group-Ctrl-Merge-a-SMA.tif]

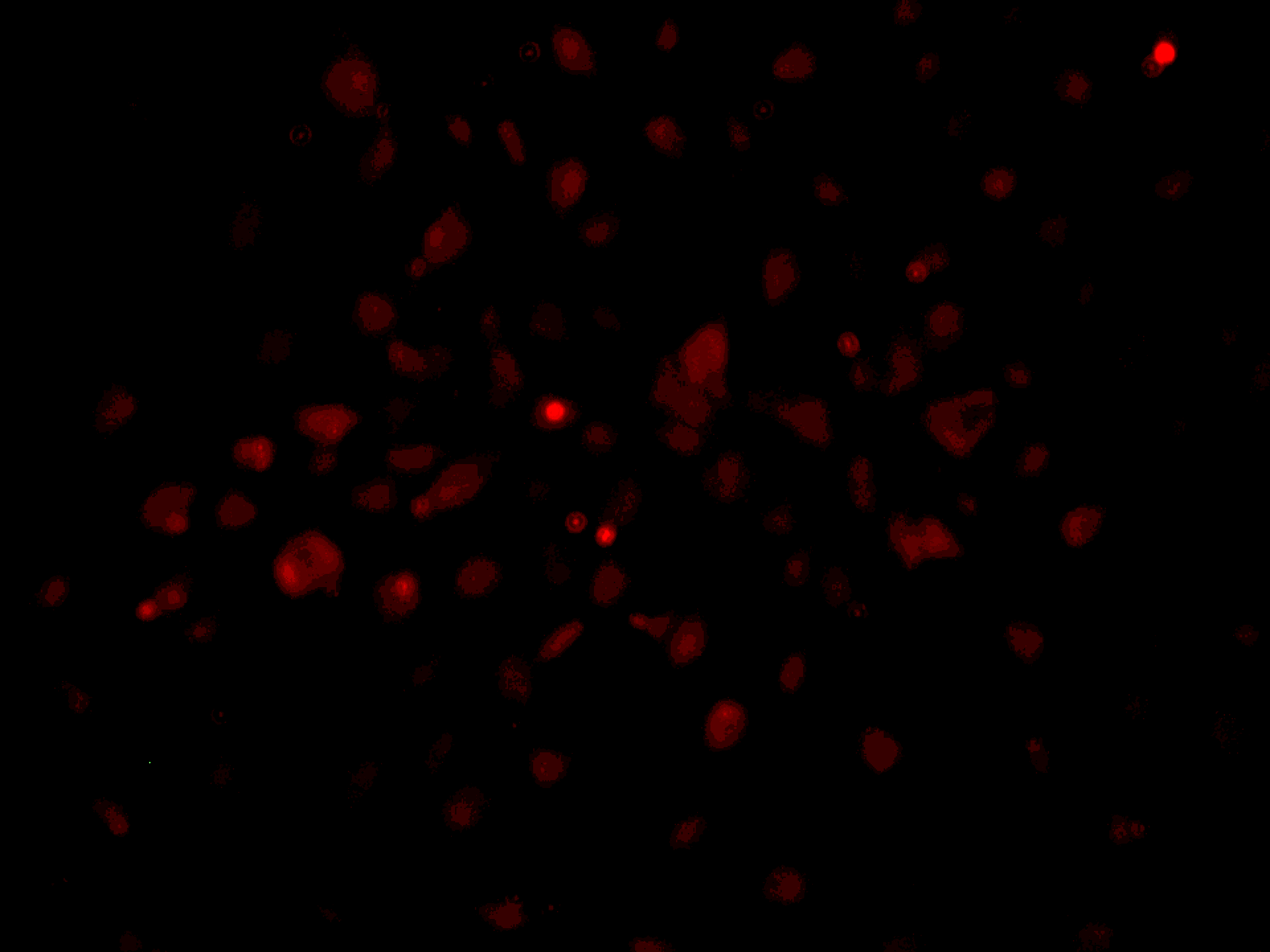

Supplement: Supplementary file 12 [file Data_Sheet_8.ZIP › Fig.6/JMJD1A group-Ctrl-a-SMA.tif]

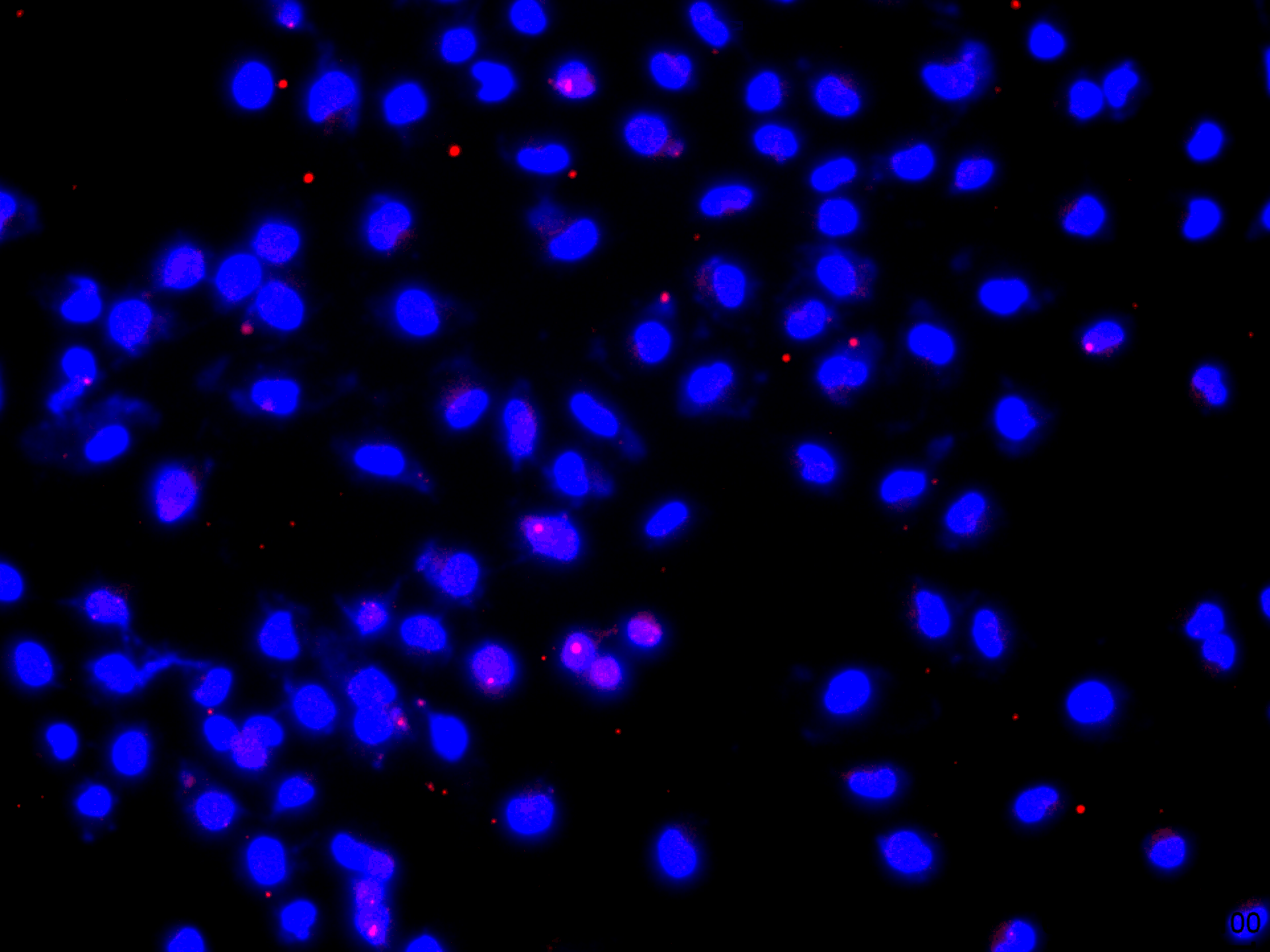

Supplement: Supplementary file 12 [file Data_Sheet_8.ZIP › Fig.6/vector group-AGEs-Merge-a-SMA.tif]

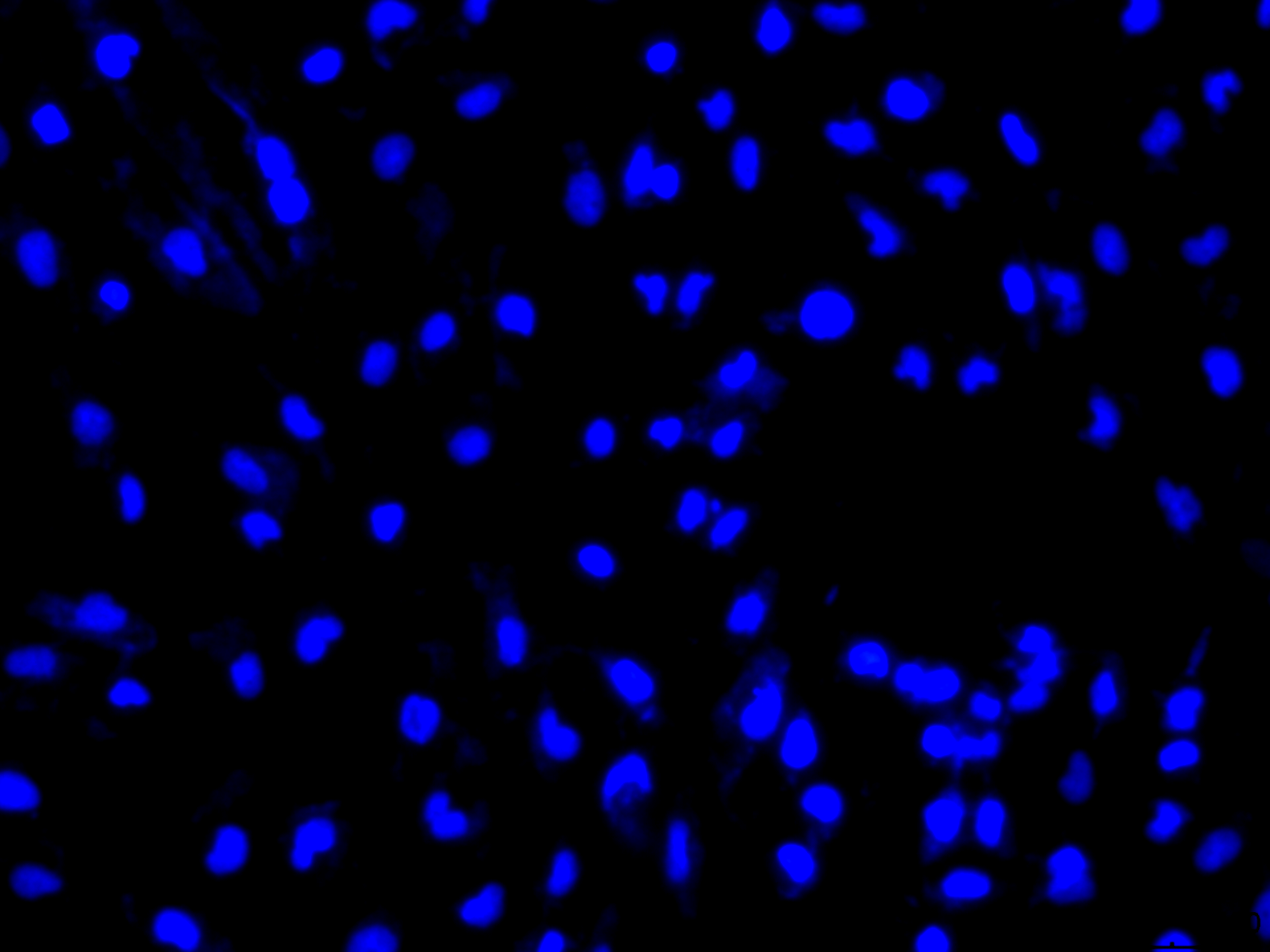

Supplement: Supplementary file 12 [file Data_Sheet_8.ZIP › Fig.6/JMJD1A group-AGEs-DAPI-a-SMA.tif]

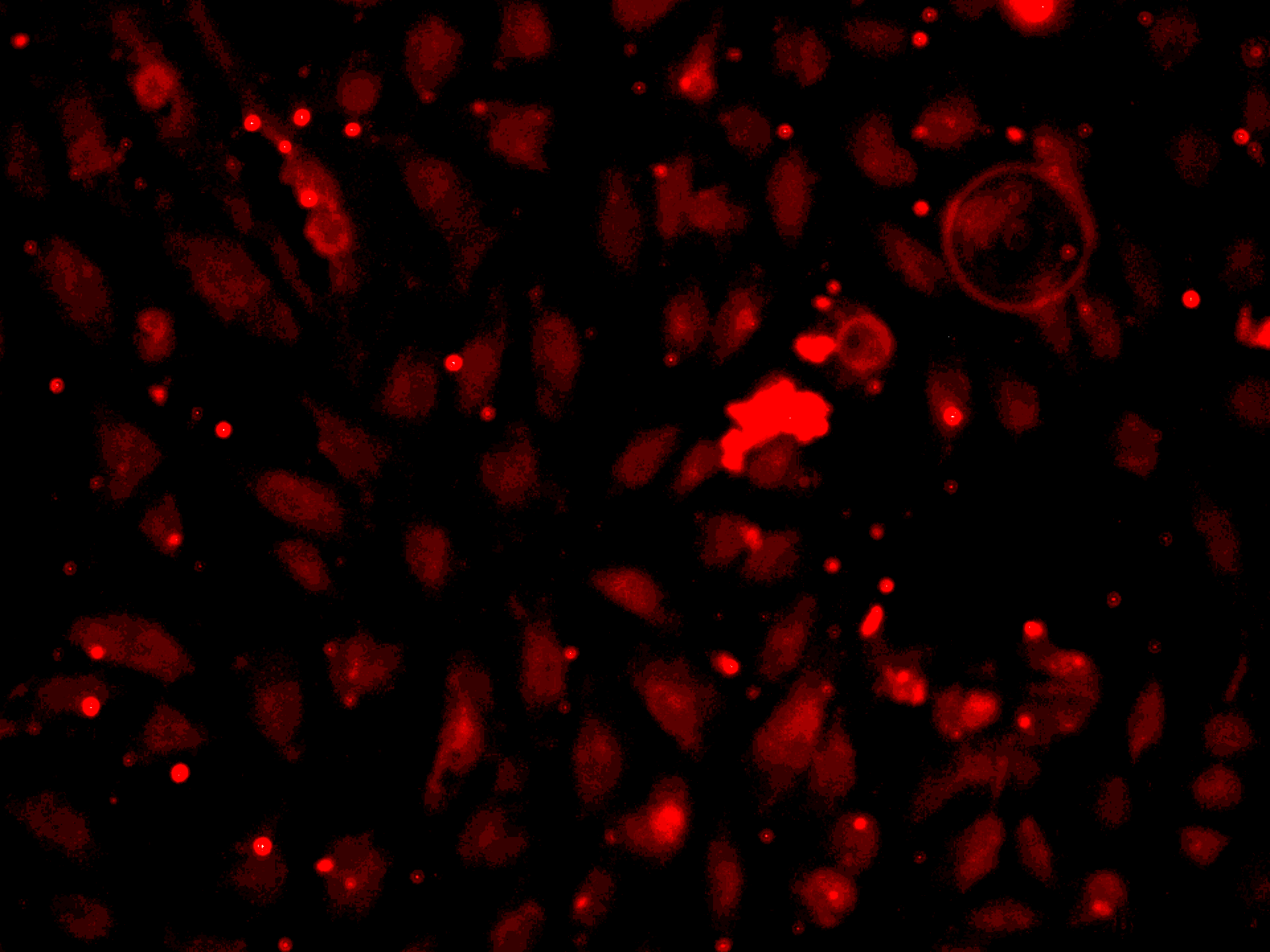

Supplement: Supplementary file 12 [file Data_Sheet_8.ZIP › Fig.6/JMJD1A group-AGEs-a-SMA.tif]

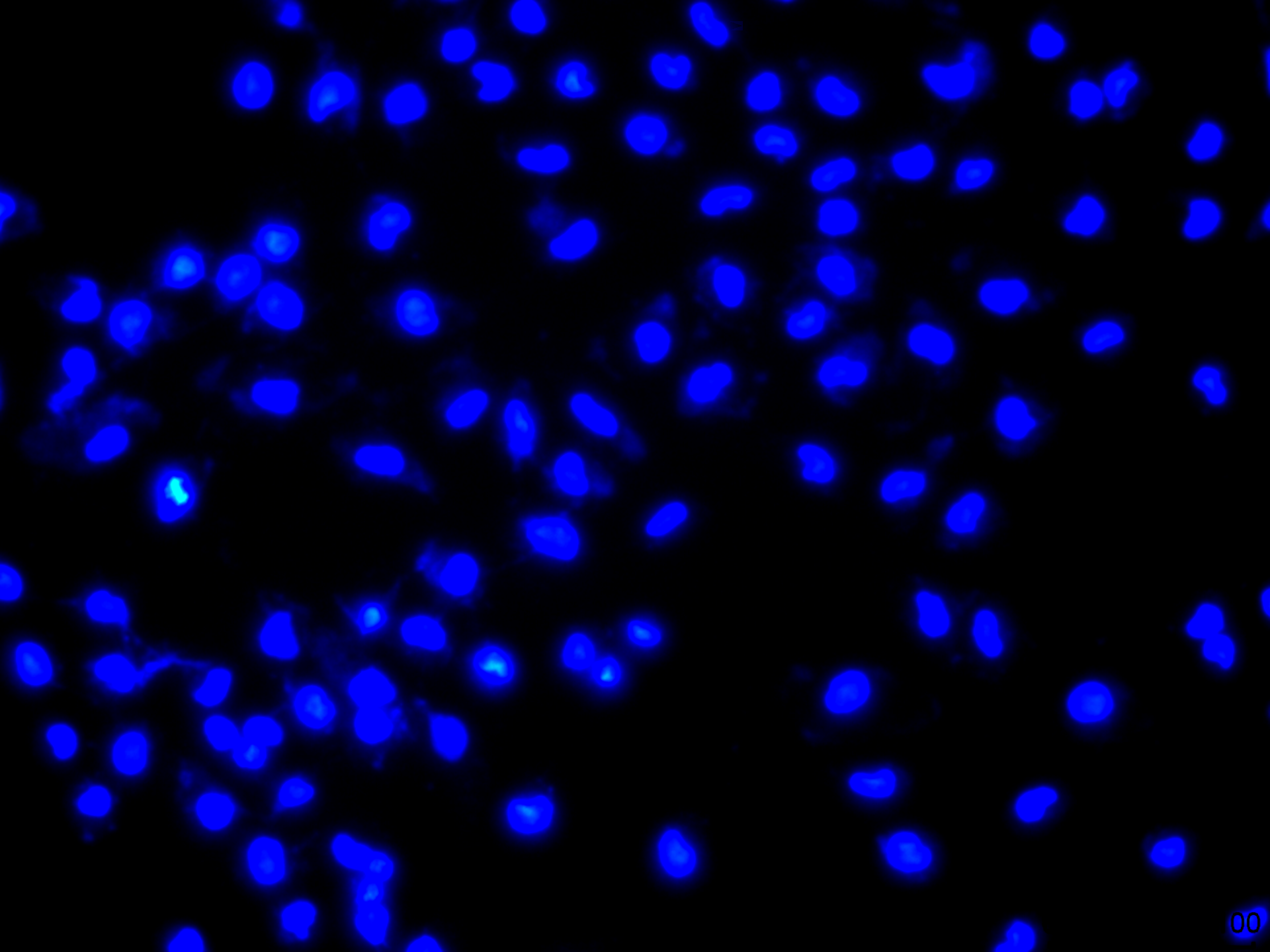

Supplement: Supplementary file 12 [file Data_Sheet_8.ZIP › Fig.6/vector group-AGEs-DAPI-a-SMA.tif]

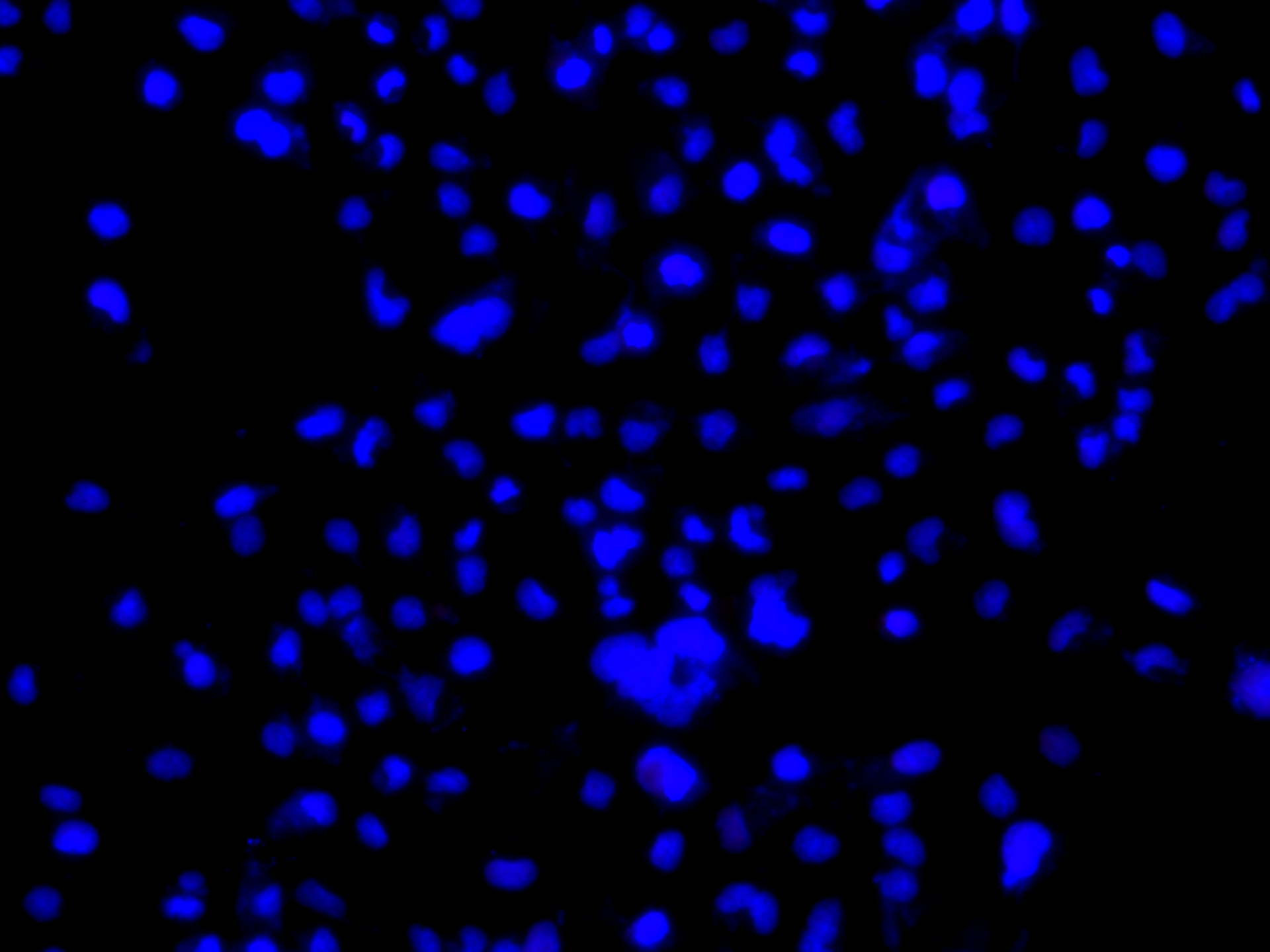

Supplement: Supplementary file 12 [file Data_Sheet_8.ZIP › Fig.6/vector group-AGEs-Merge-JMJD1A.tif]

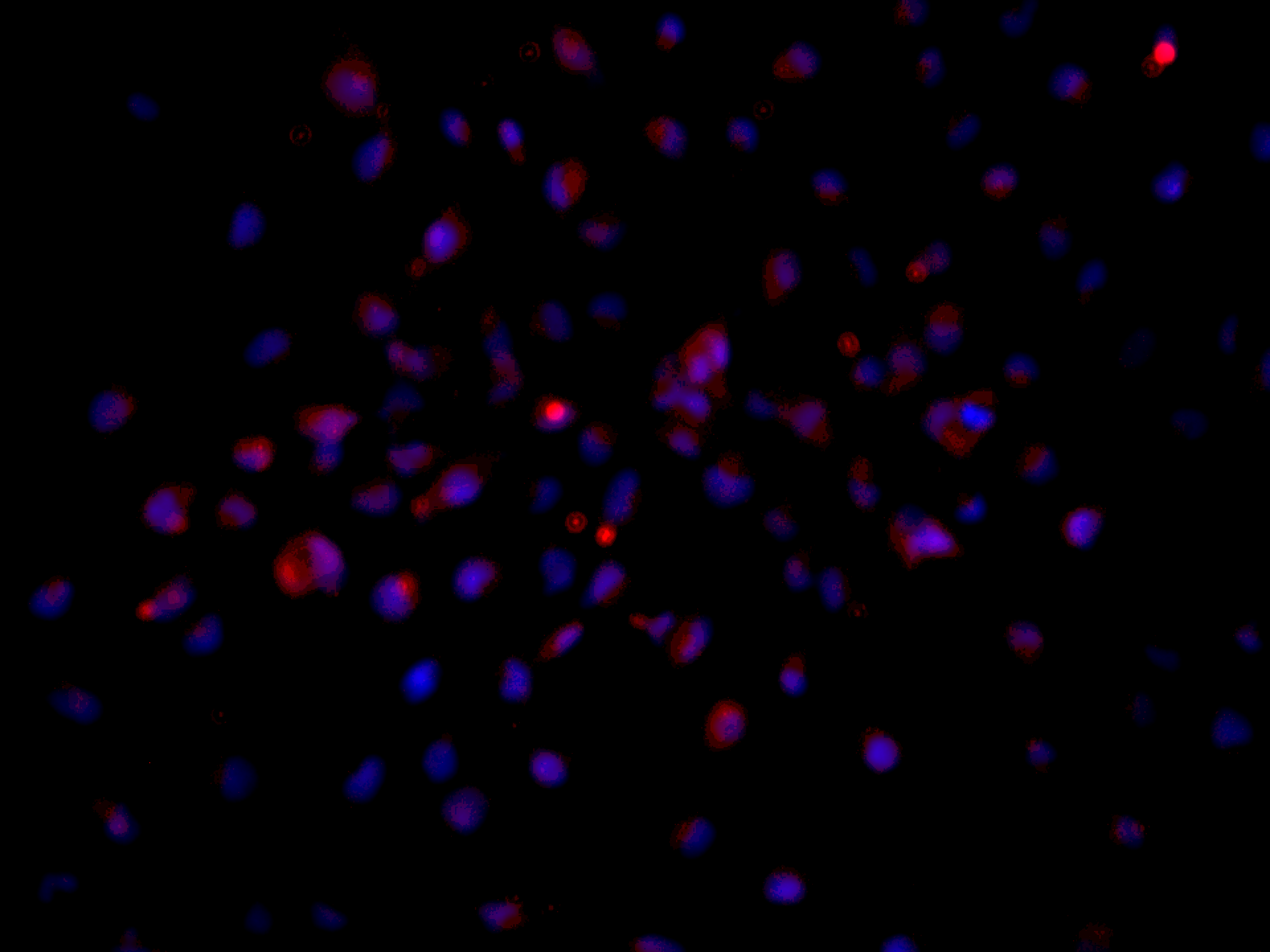

Supplement: Supplementary file 12 [file Data_Sheet_8.ZIP › Fig.6/JMJD1A group-Ctrl-Merge-a-SMA.tif]

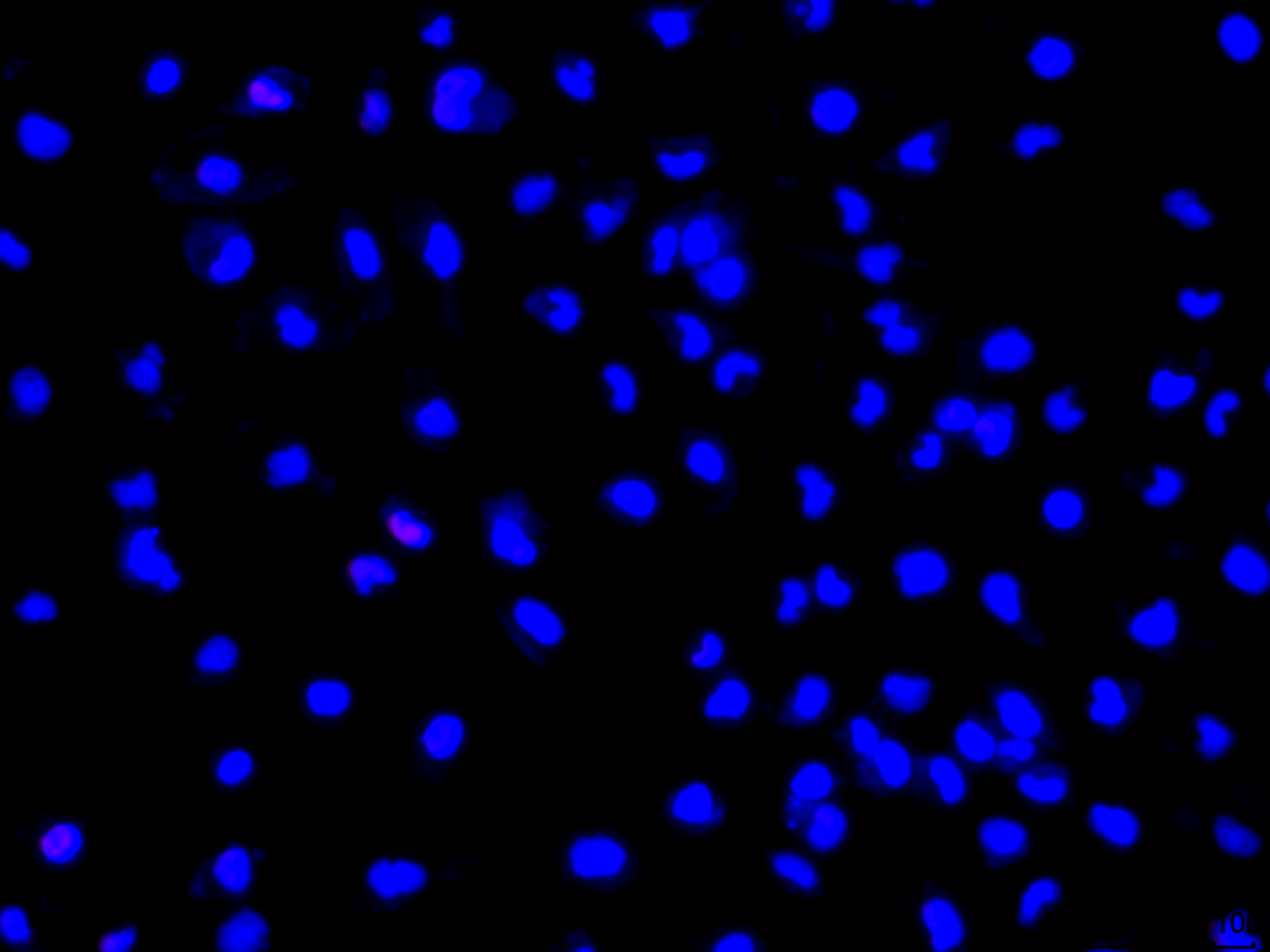

Supplement: Supplementary file 12 [file Data_Sheet_8.ZIP › Fig.6/JMJD1A group-Ctrl-Merge-JMJD1A.tif]

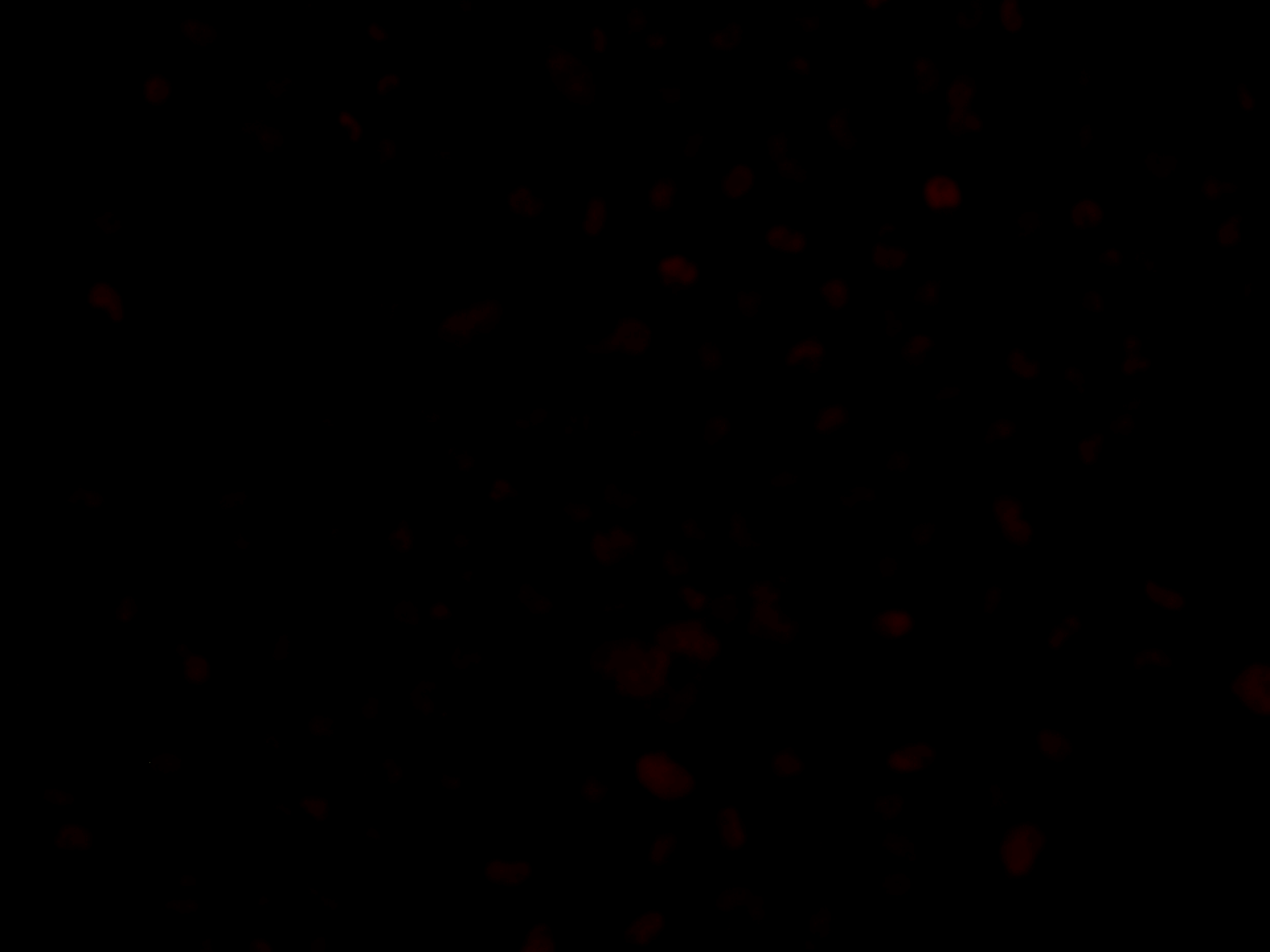

Supplement: Supplementary file 12 [file Data_Sheet_8.ZIP › Fig.6/vector group-AGEs-JMJD1A.tif]

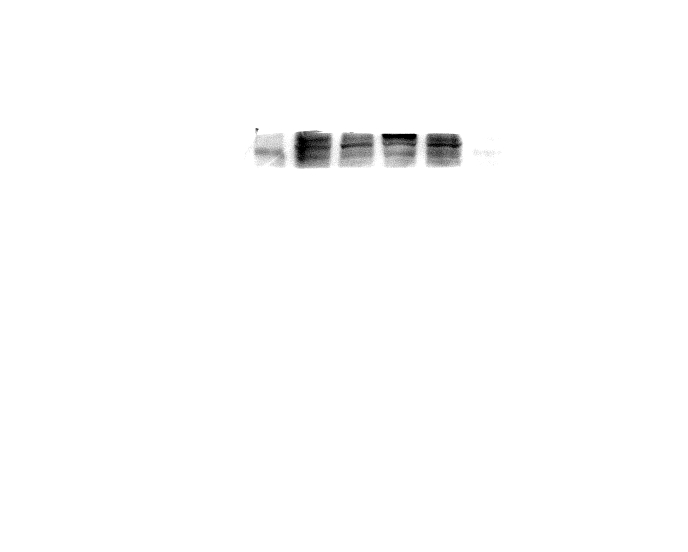

Supplement: Supplementary file 12 [file Data_Sheet_8.ZIP › Fig.6/TGF-B1-G-4.jpg]

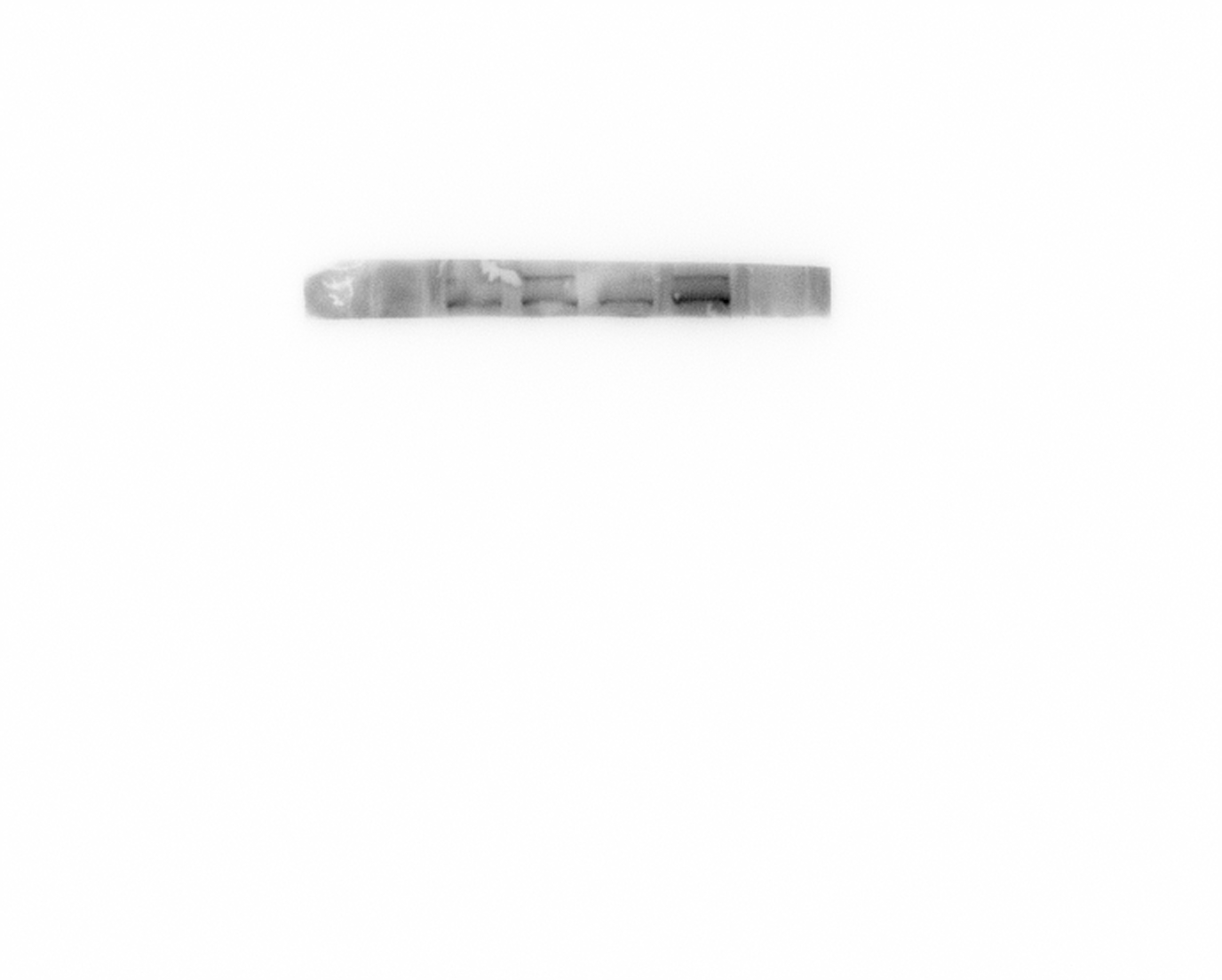

Supplement: Supplementary file 12 [file Data_Sheet_8.ZIP › Fig.6/JMJD1A-Φ┐ç.jpg]
