# Supplementary figures and images for: JMJD1A/NR4A1 Signaling Regulates the Procession of Renal Tubular Epithelial Interstitial Fibrosis Induced by AGEs in HK-2 (part 2 of 2)
Source: Front Med (Lausanne). 2022 Feb 3;8:807694. doi: 10.3389/fmed.2021.807694 (PMC8850412; doi:10.3389/fmed.2021.807694)

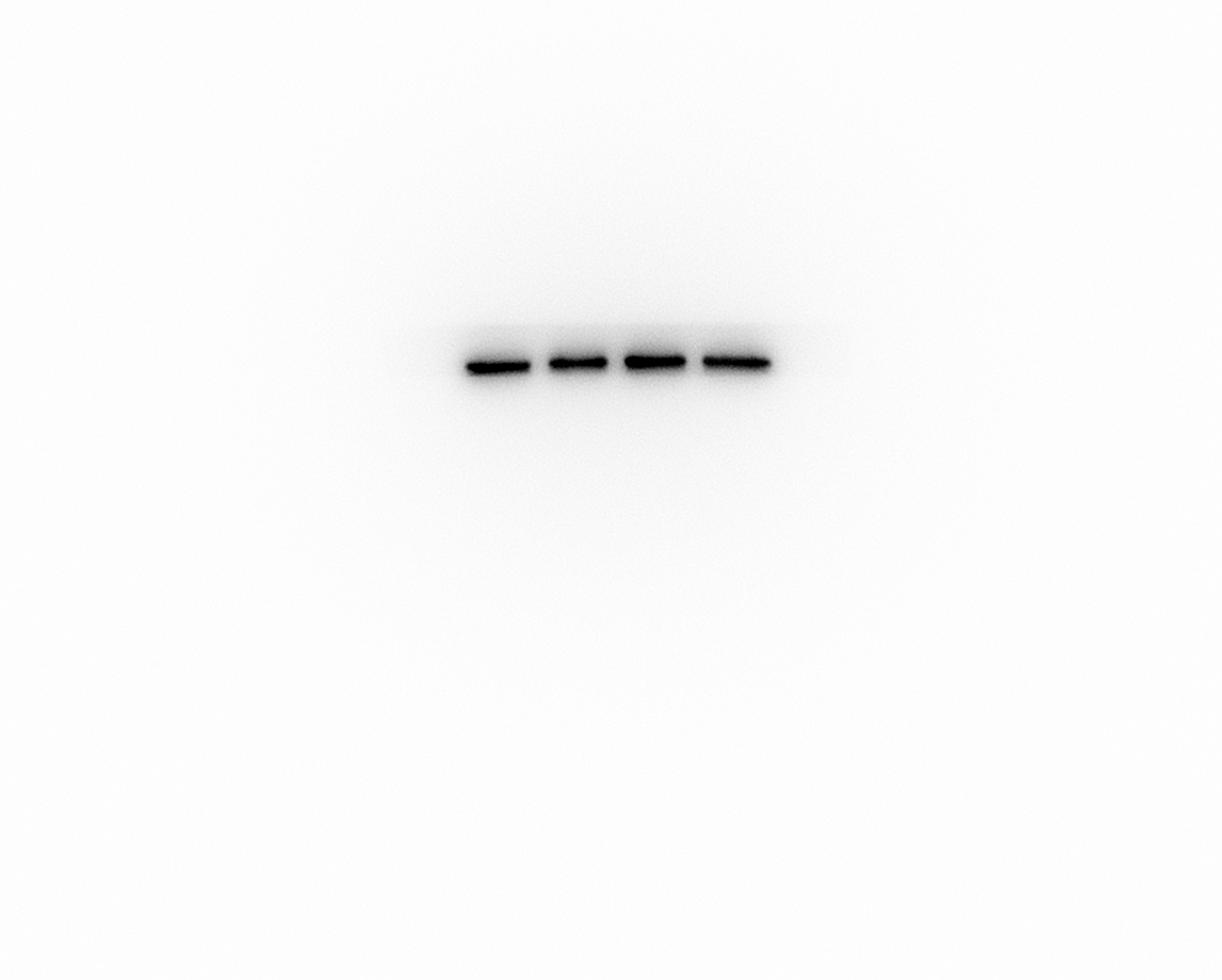

Supplement: Supplementary file 12 [file Data_Sheet_8.ZIP › Fig.6/GAPDH.jpg]

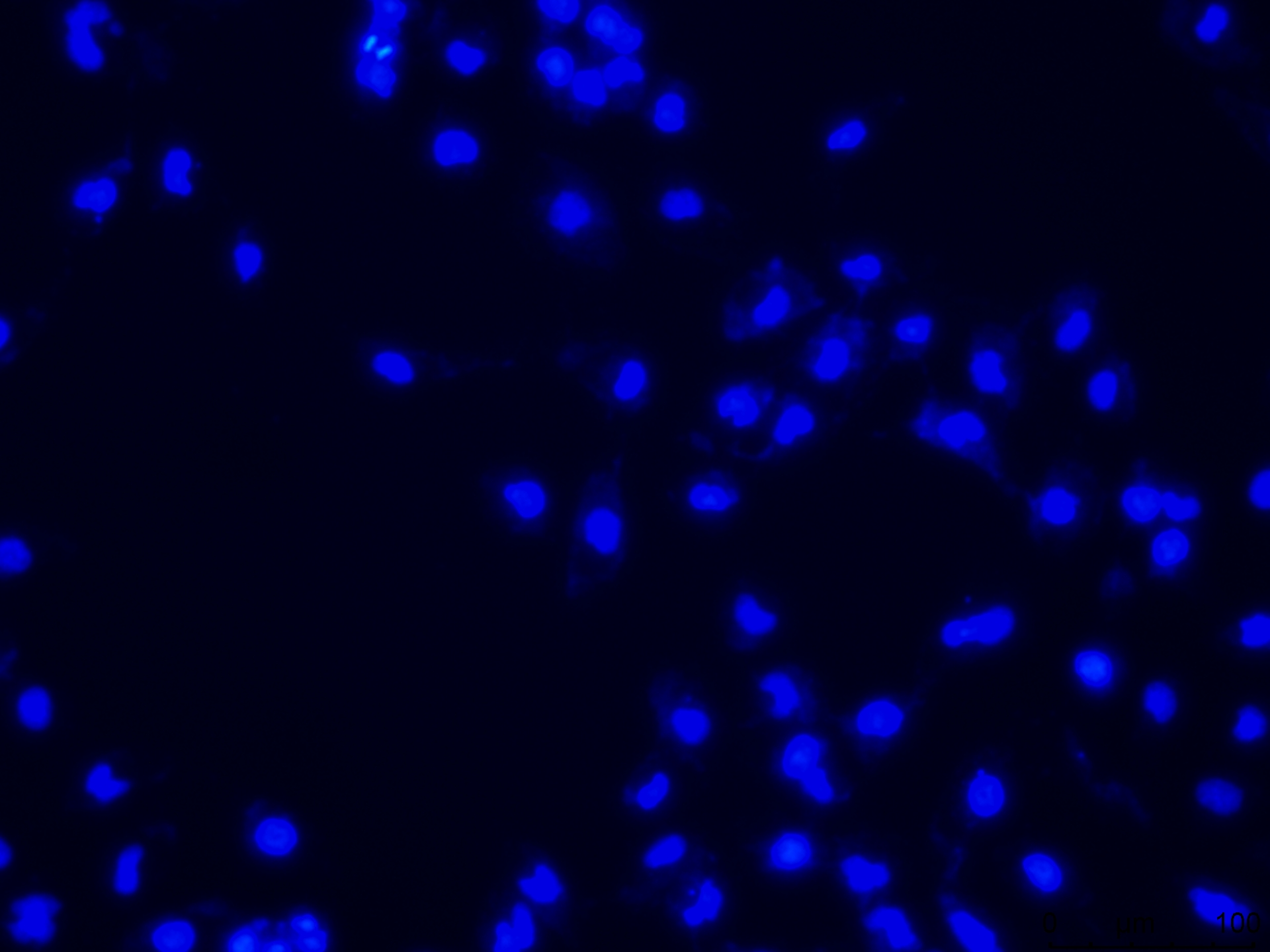

Supplement: Supplementary file 12 [file Data_Sheet_8.ZIP › Fig.6/vector group-Ctrl-DAPI-a-SMA.tif]

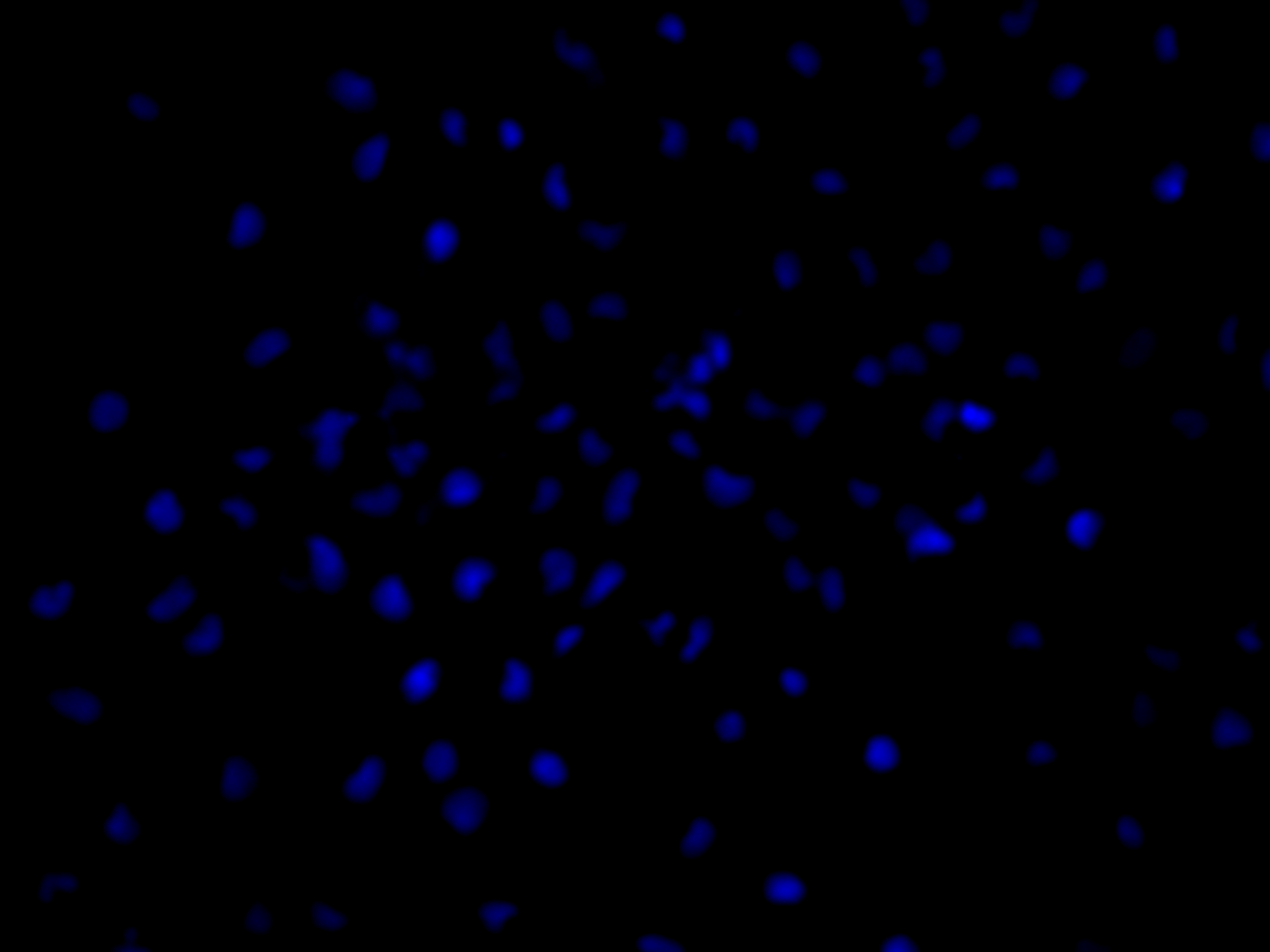

Supplement: Supplementary file 12 [file Data_Sheet_8.ZIP › Fig.6/JMJD1A group-Ctrl-DAPI-a-SMA.tif]

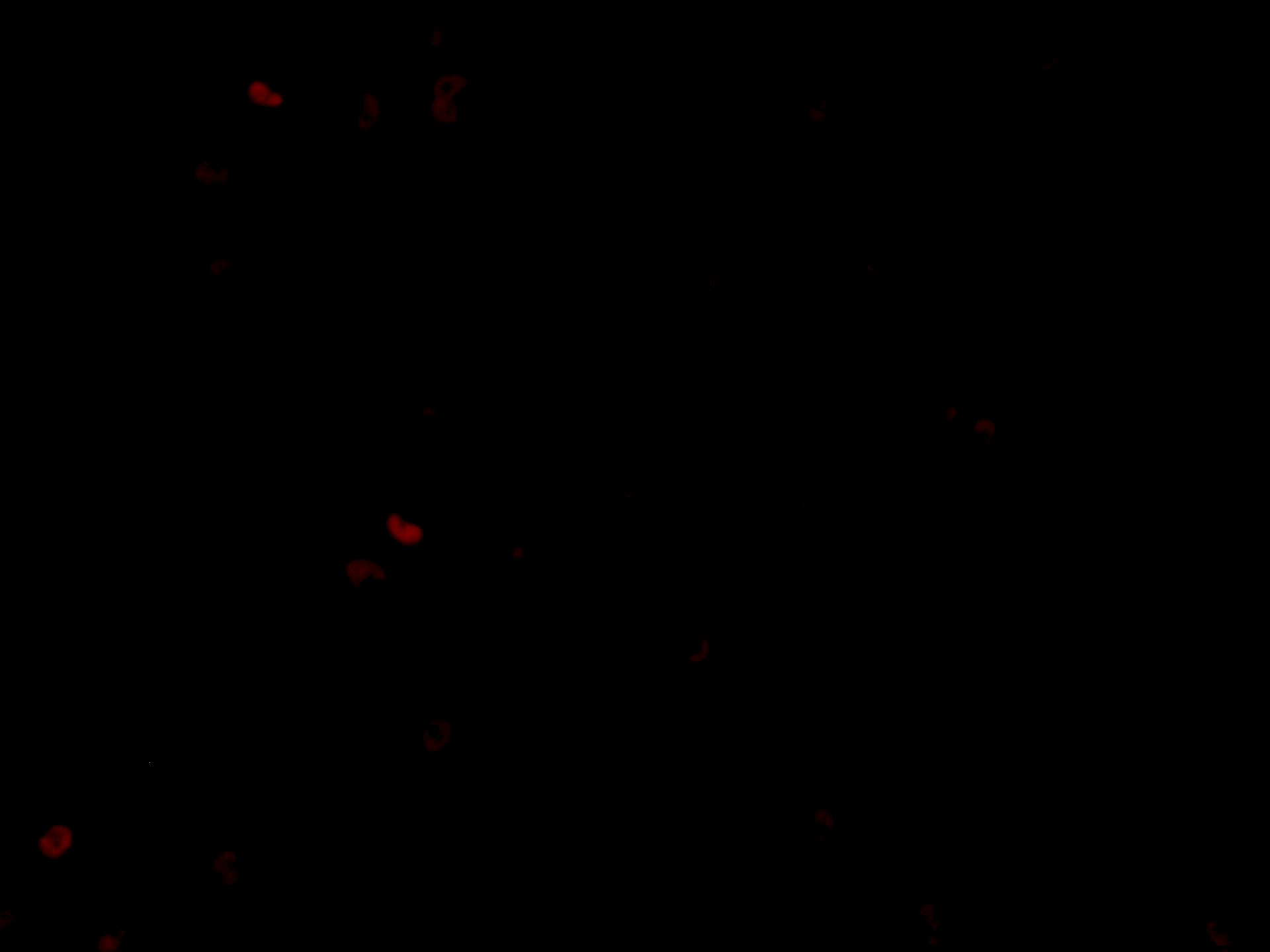

Supplement: Supplementary file 12 [file Data_Sheet_8.ZIP › Fig.6/JMJD1A group-Ctrl-JMJD1A.tif]

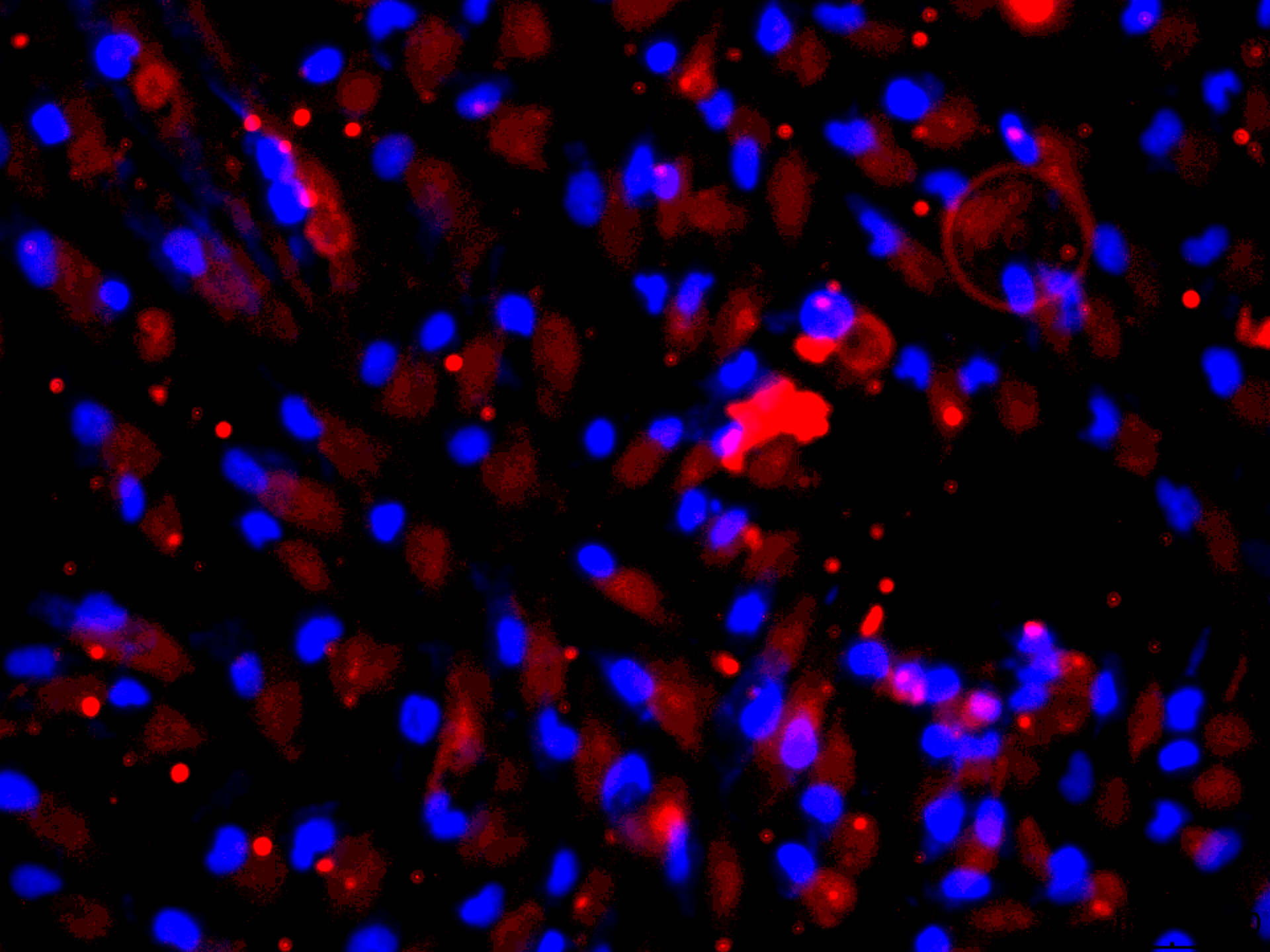

Supplement: Supplementary file 12 [file Data_Sheet_8.ZIP › Fig.6/JMJD1A group-AGEs-Merge-a-SMA.tif]

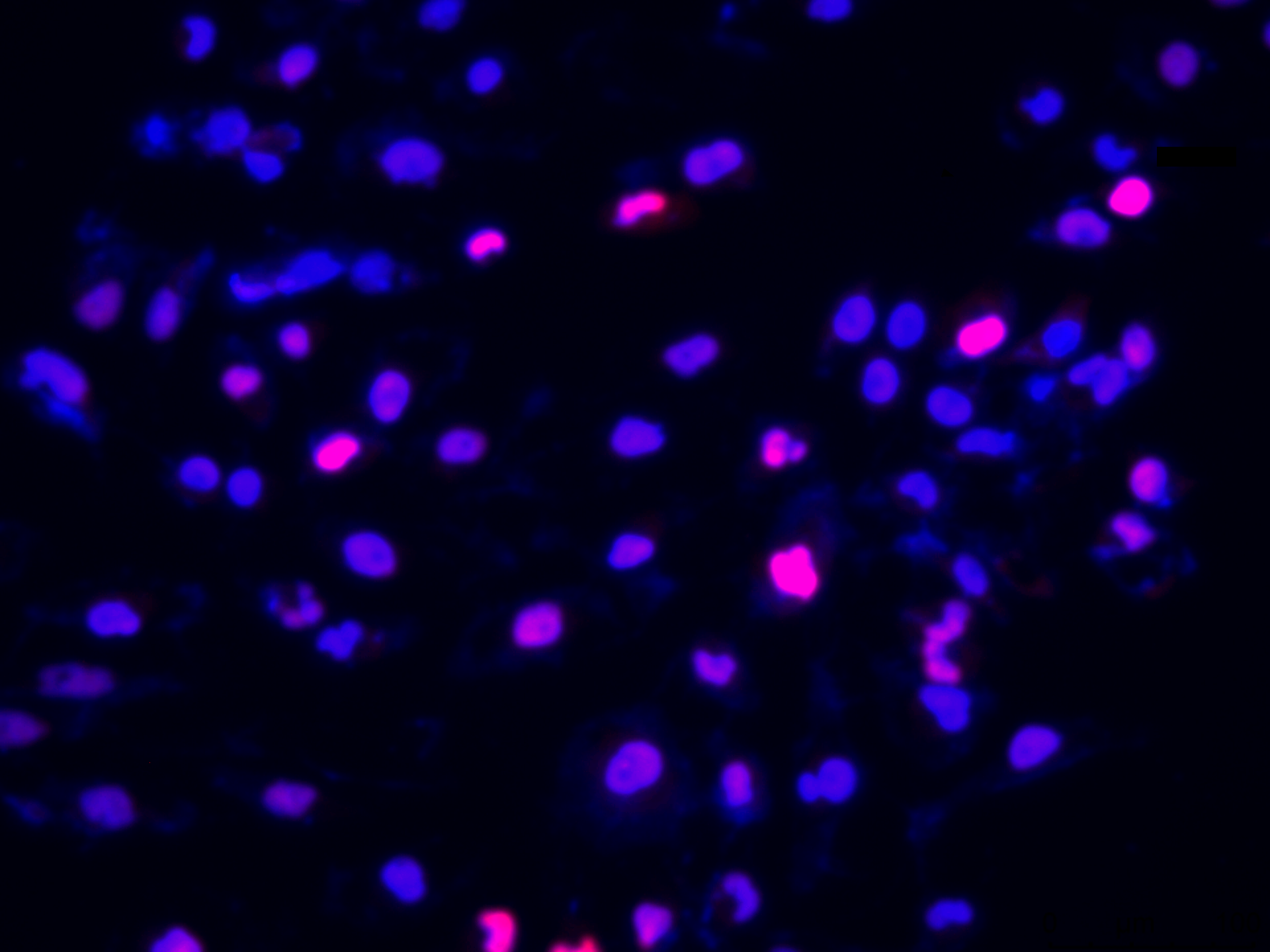

Supplement: Supplementary file 12 [file Data_Sheet_8.ZIP › Fig.6/JMJD1A group-AGEs-Merge-JMJD1A.tif]

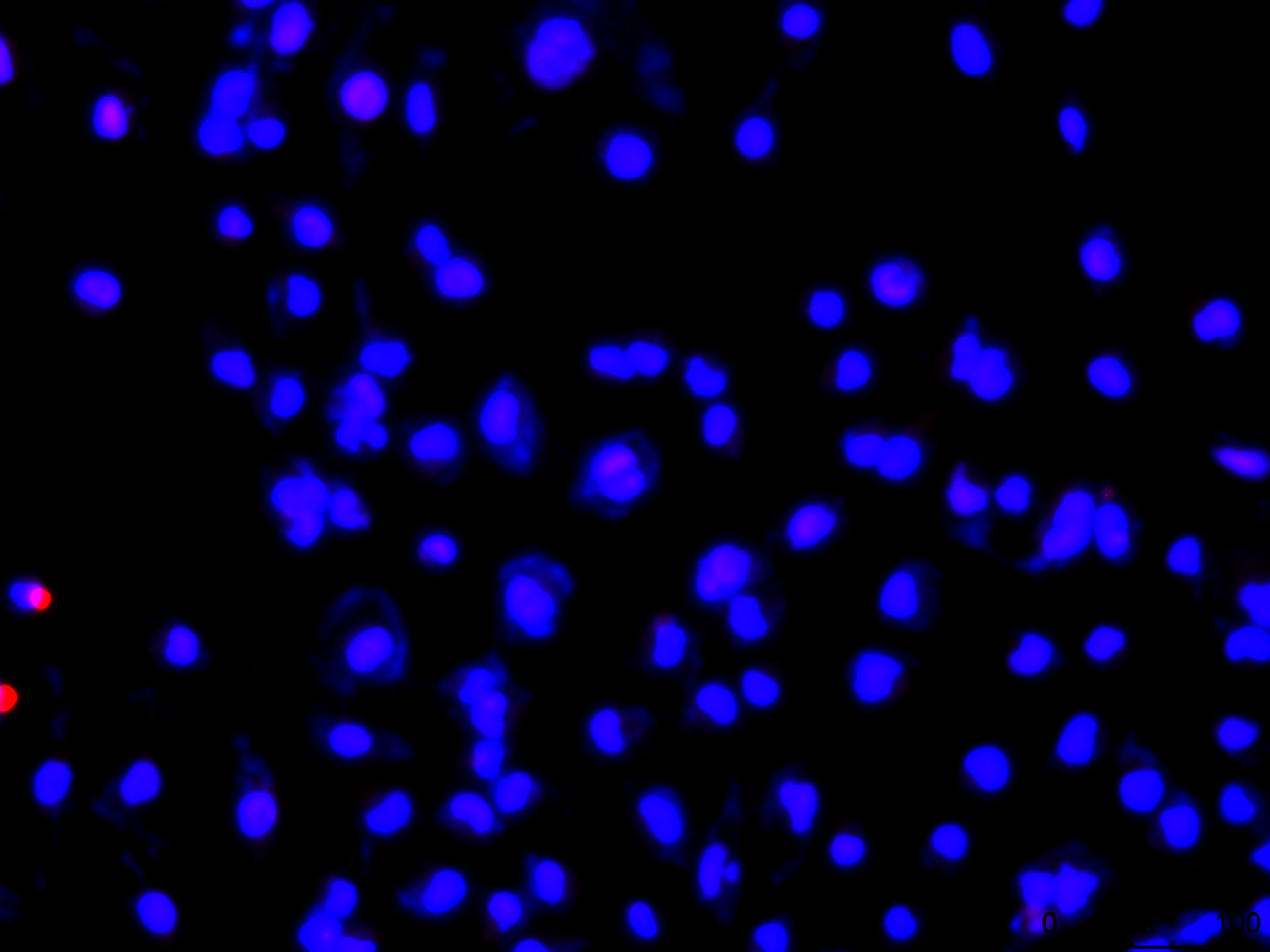

Supplement: Supplementary file 12 [file Data_Sheet_8.ZIP › Fig.6/vector group-Ctrl-Merge-JMJD1A.tif]

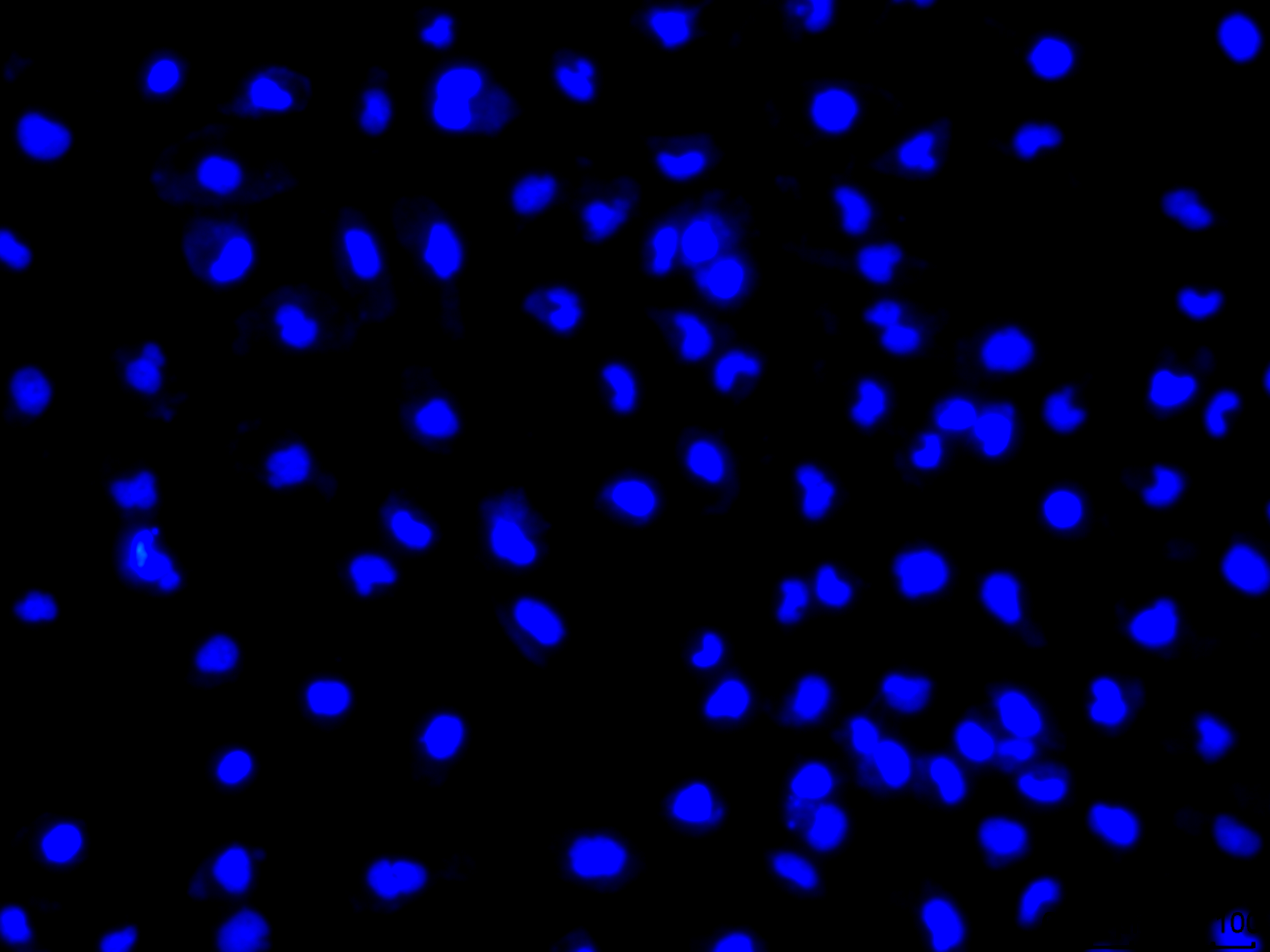

Supplement: Supplementary file 12 [file Data_Sheet_8.ZIP › Fig.6/JMJD1A group-Ctrl-DAPI-JMJD1A.tif]

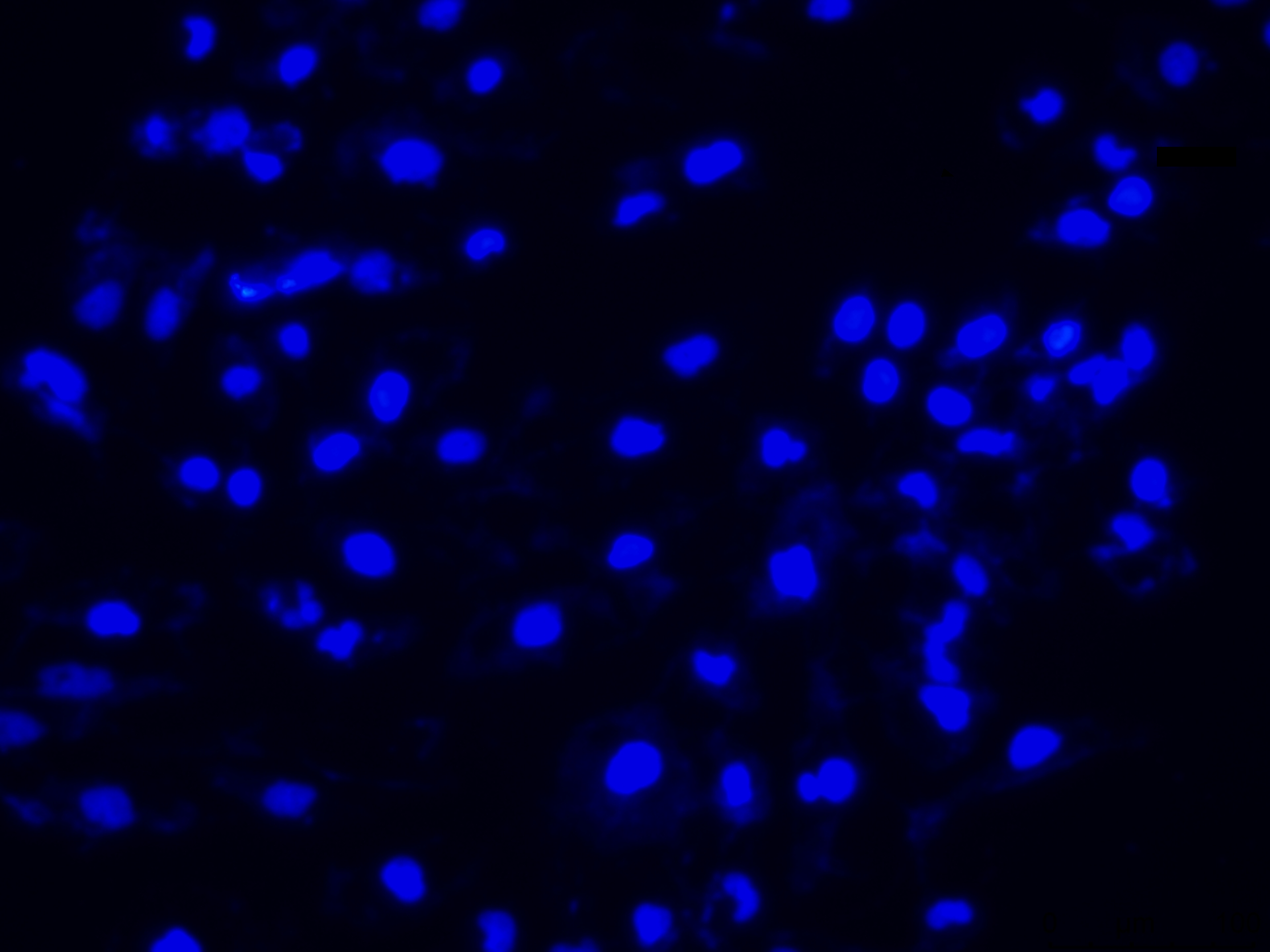

Supplement: Supplementary file 12 [file Data_Sheet_8.ZIP › Fig.6/JMJD1A group-AGEs-DAPI-JMJD1A.tif]

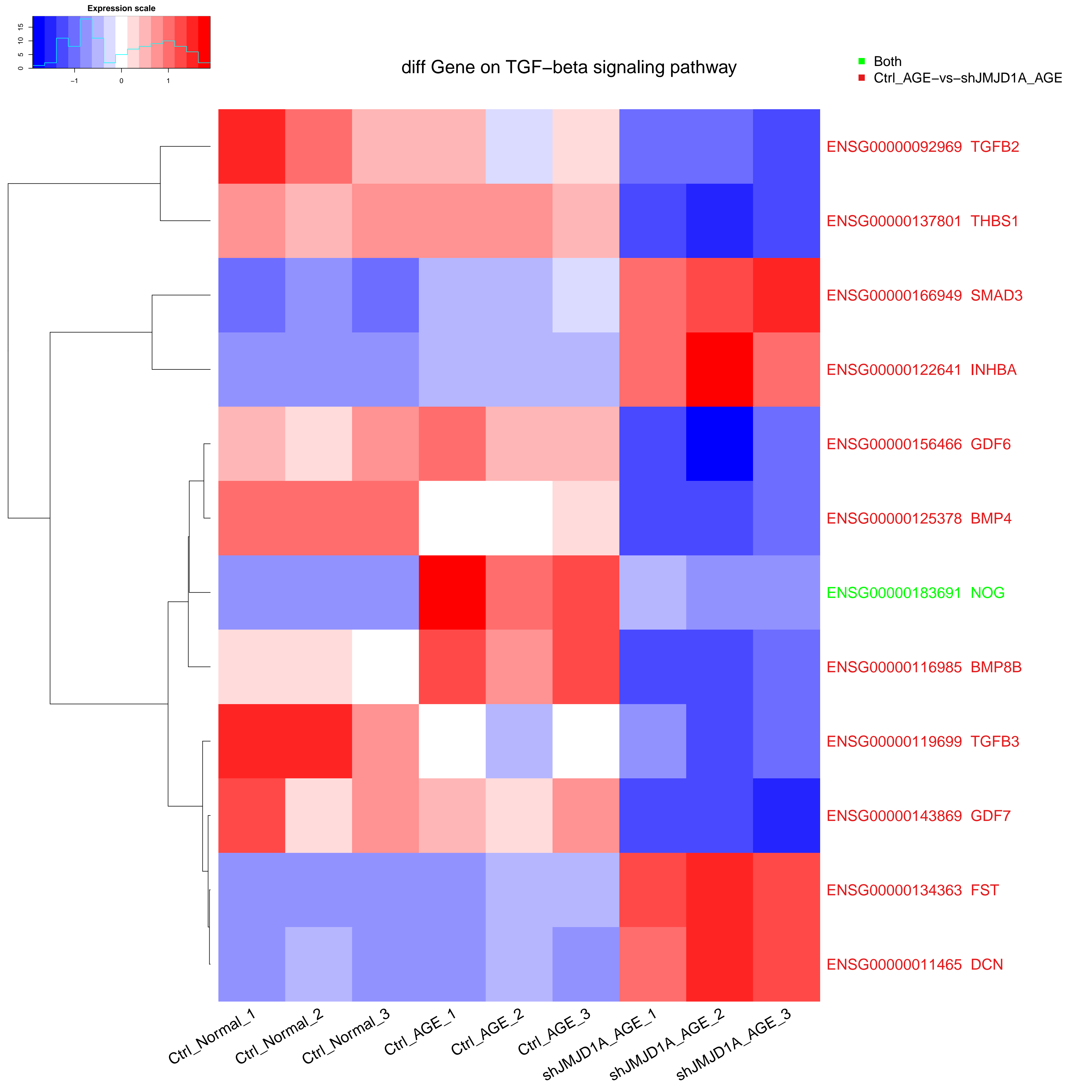

Supplement: Supplementary file 13 [file Data_Sheet_9.ZIP › Fig.7/ko04350.diffGene.expr.pdf]

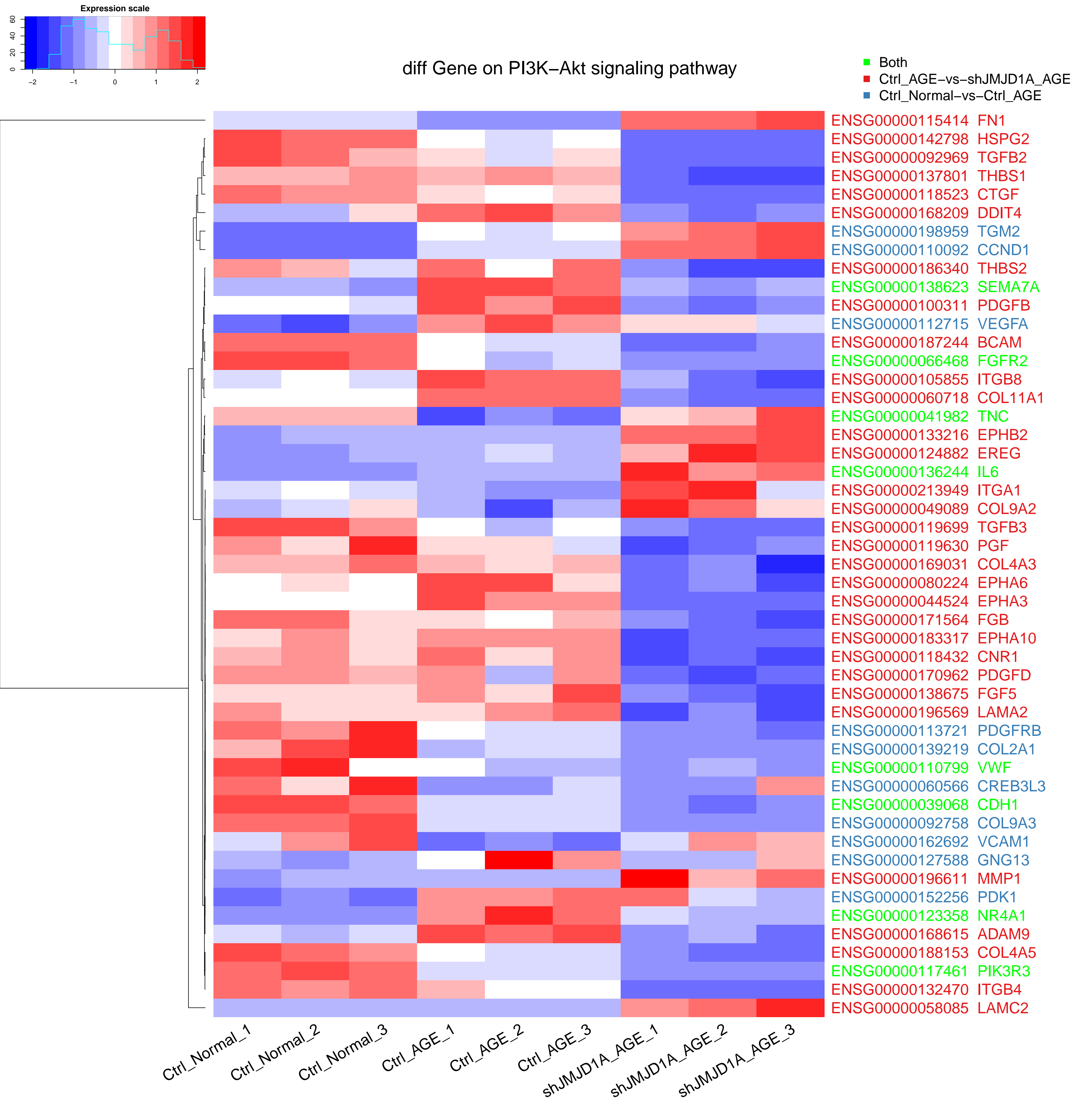

Supplement: Supplementary file 13 [file Data_Sheet_9.ZIP › Fig.7/ko04151.diffGene.expr.pdf]

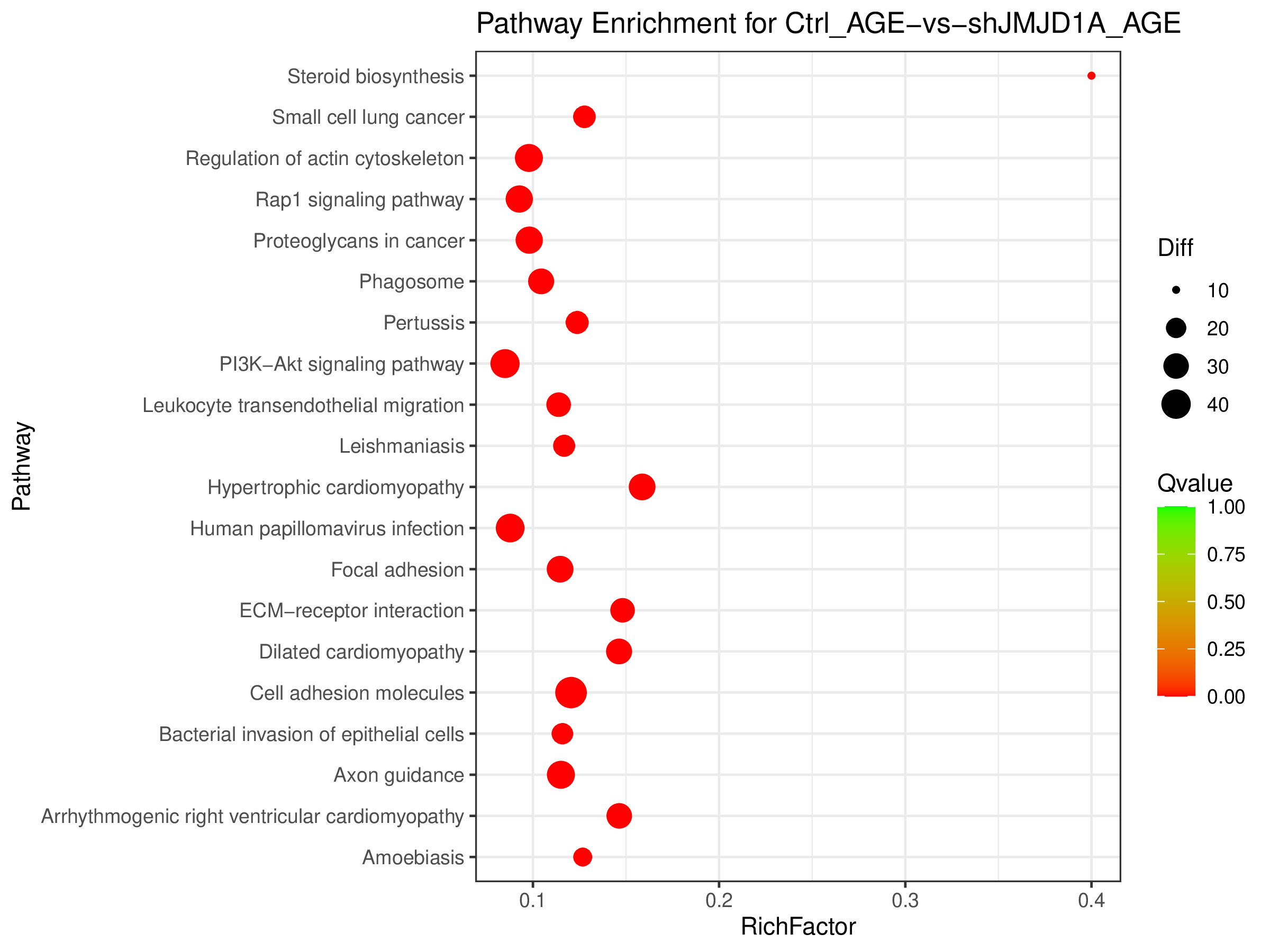

Supplement: Supplementary file 13 [file Data_Sheet_9.ZIP › Fig.7/Ctrl_AGE-vs-shJMJD1A_AGE.Pathenrich.png]

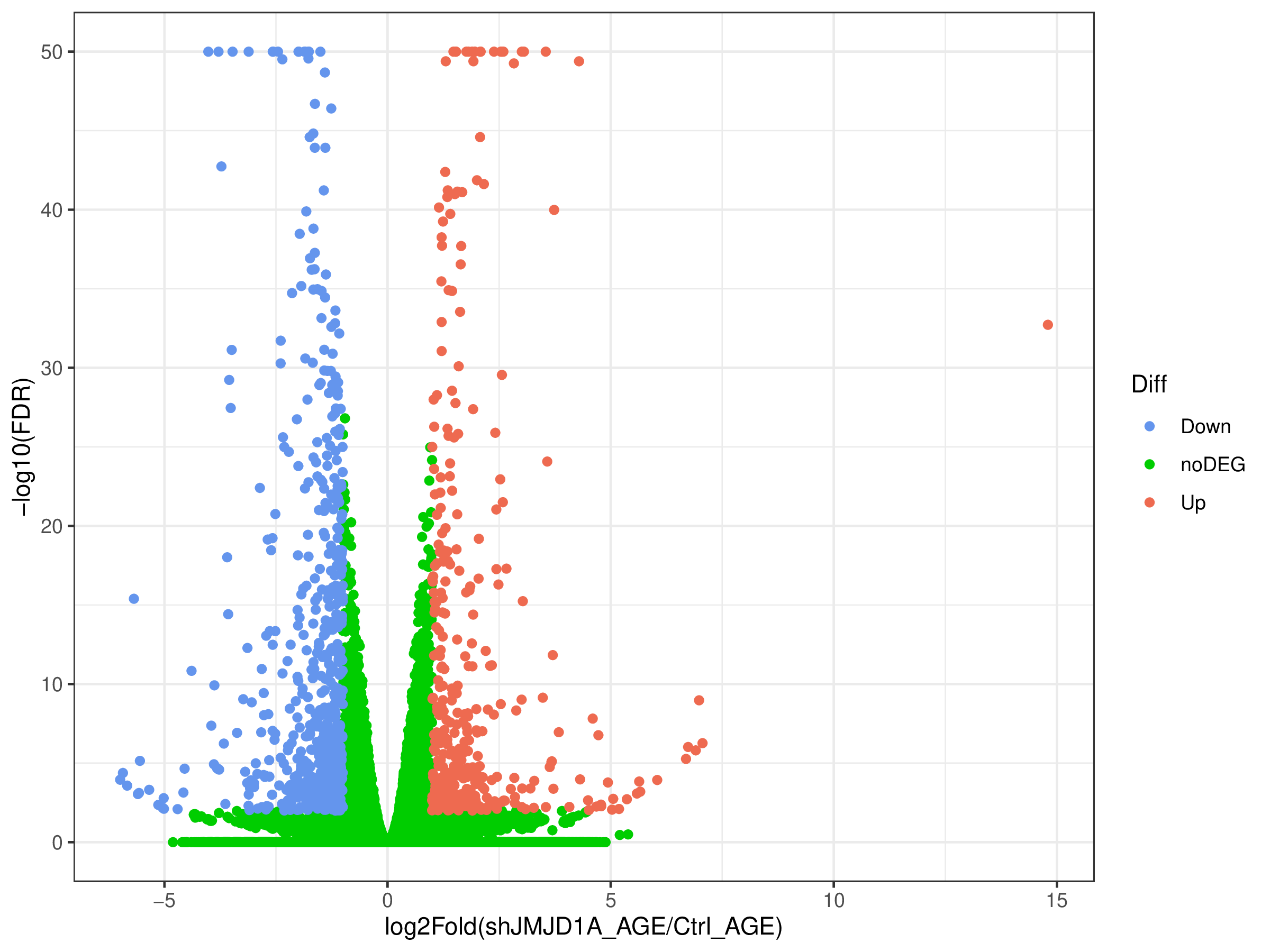

Supplement: Supplementary file 13 [file Data_Sheet_9.ZIP › Fig.7/Ctrl_AGE-vs-shJMJD1A_AGE.Volcano.png]

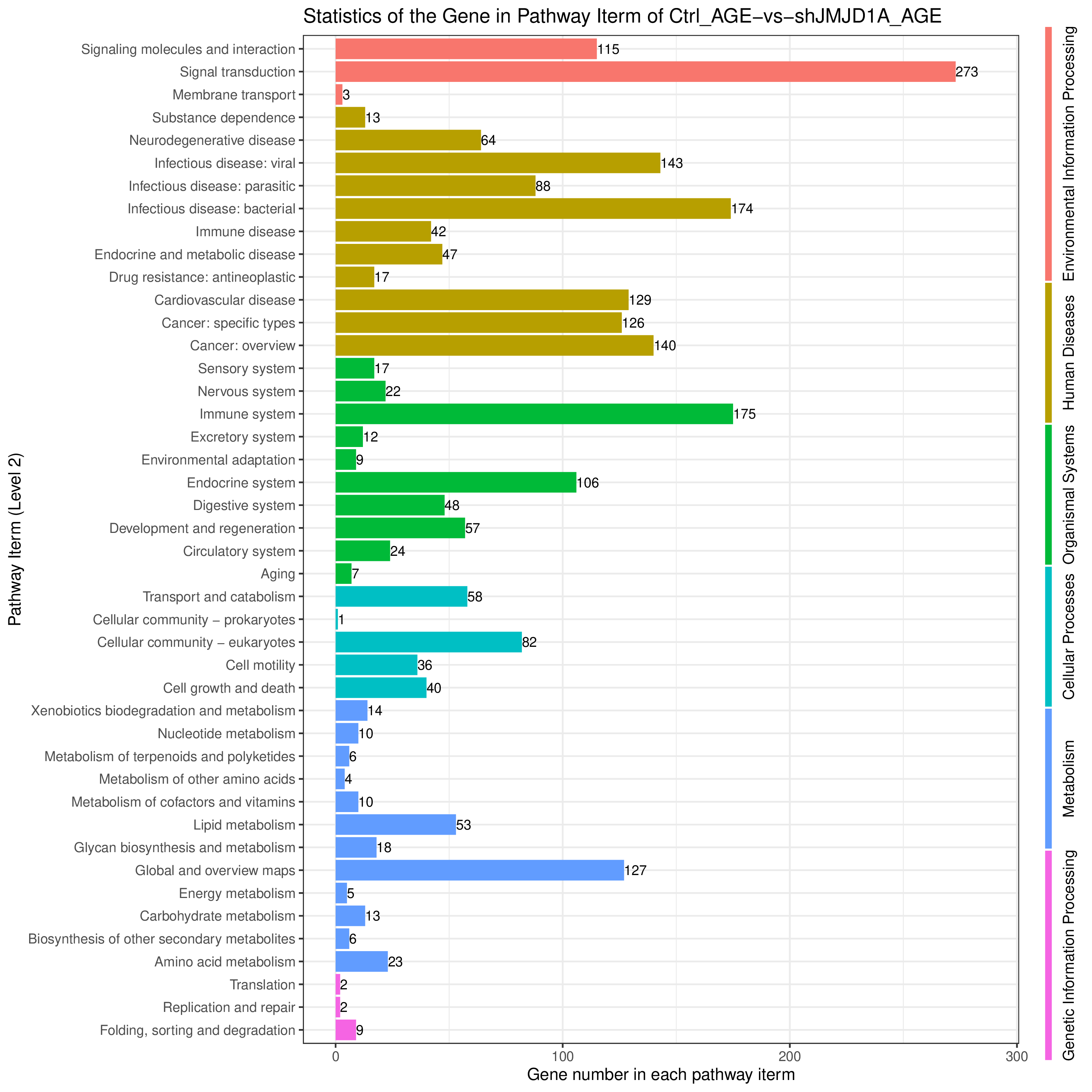

Supplement: Supplementary file 13 [file Data_Sheet_9.ZIP › Fig.7/Ctrl_AGE-vs-shJMJD1A_AGE.path_class.png]

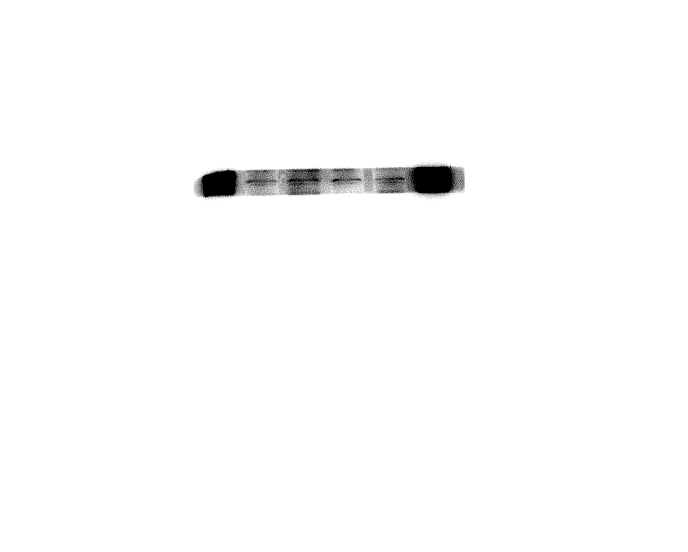

Supplement: Supplementary file 14 [file Data_Sheet_10.ZIP › Fig.8/E-CAD.jpg]

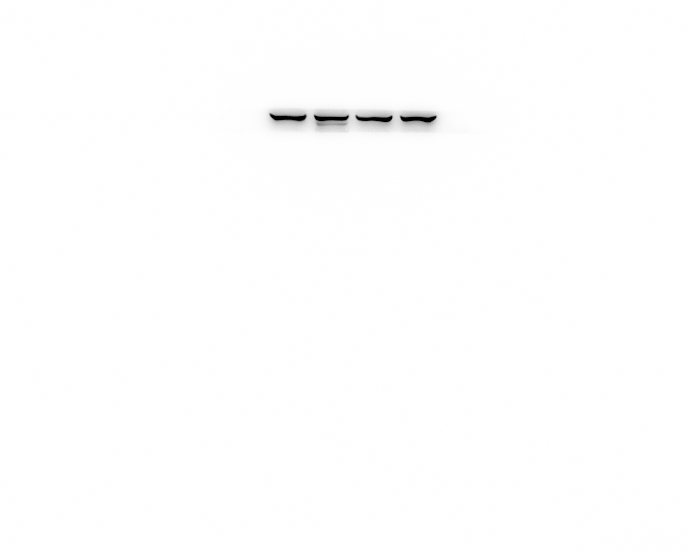

Supplement: Supplementary file 14 [file Data_Sheet_10.ZIP › Fig.8/VIM.jpg]

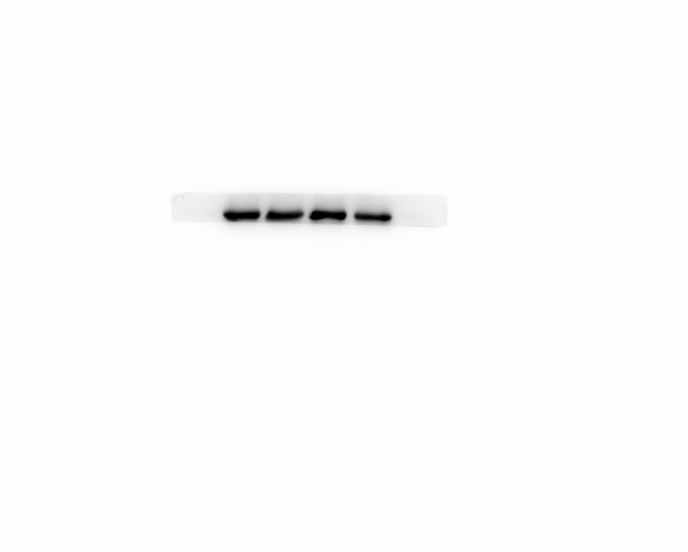

Supplement: Supplementary file 14 [file Data_Sheet_10.ZIP › Fig.8/GAPDH-Φ┐ç.jpg]

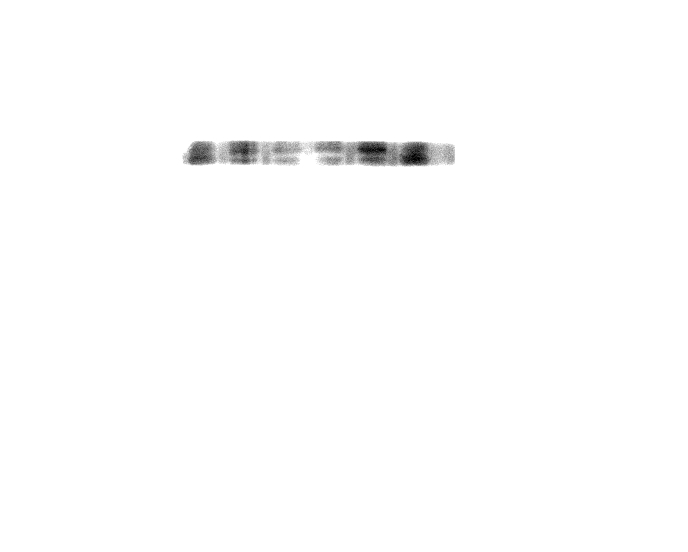

Supplement: Supplementary file 14 [file Data_Sheet_10.ZIP › Fig.8/CTGF-1.jpg]

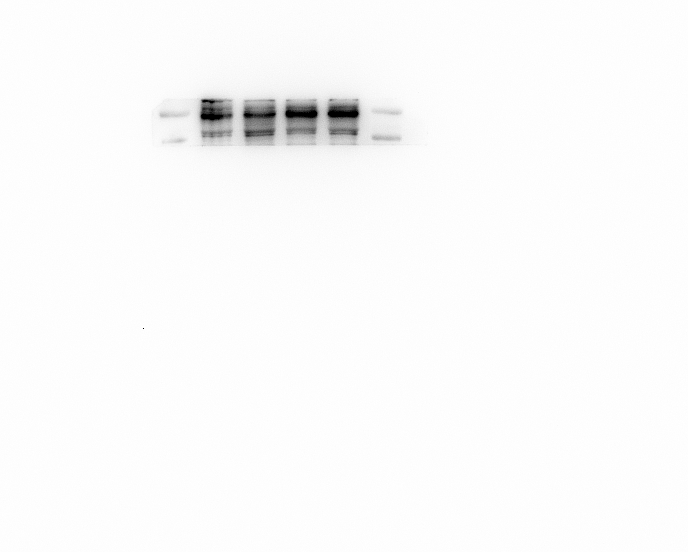

Supplement: Supplementary file 14 [file Data_Sheet_10.ZIP › Fig.8/TGF-B1.jpg]

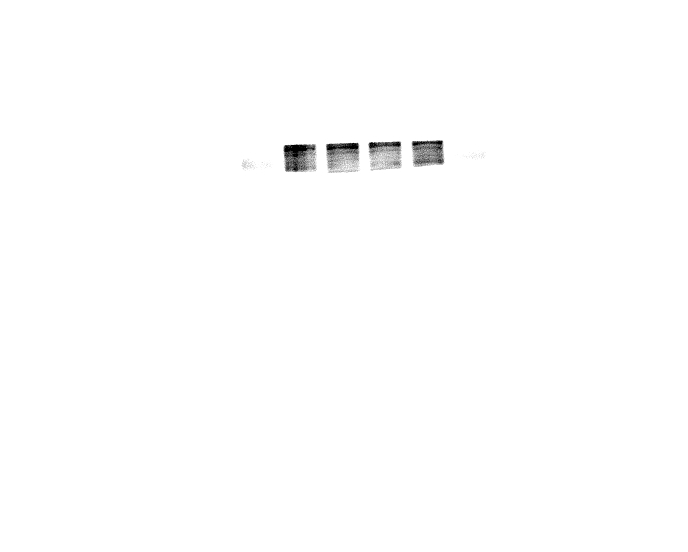

Supplement: Supplementary file 14 [file Data_Sheet_10.ZIP › Fig.8/NR4A1-5.jpg]

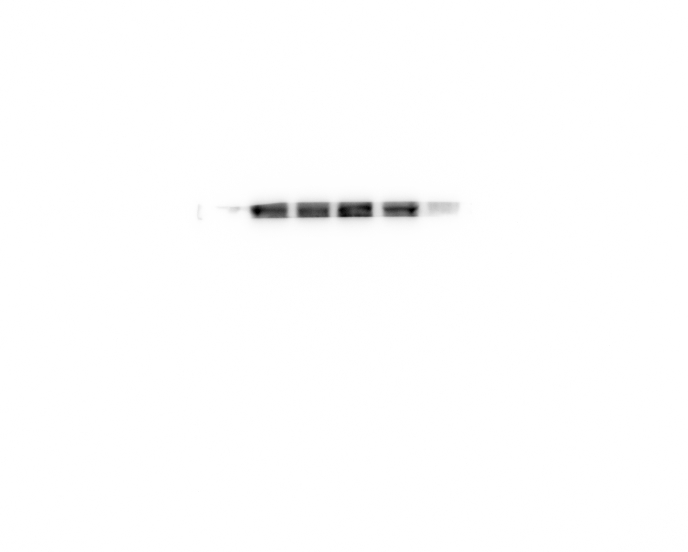

Supplement: Supplementary file 14 [file Data_Sheet_10.ZIP › Fig.8/NR4A1-G-2.jpg]

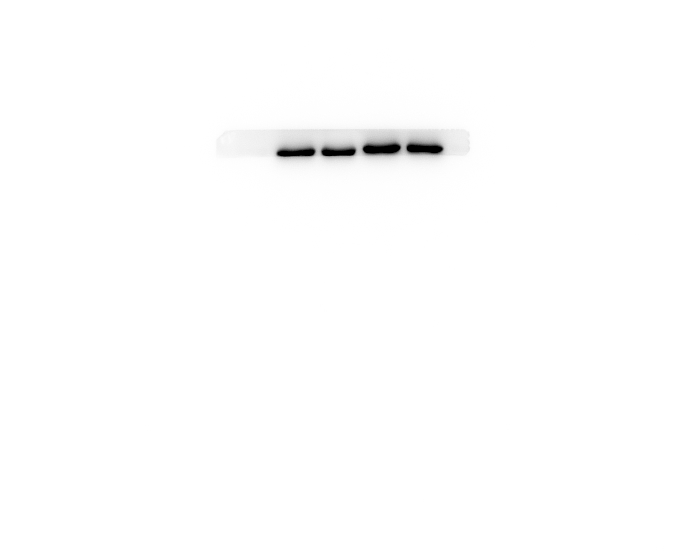

Supplement: Supplementary file 14 [file Data_Sheet_10.ZIP › Fig.8/GAPDH-μò▓-2.jpg]

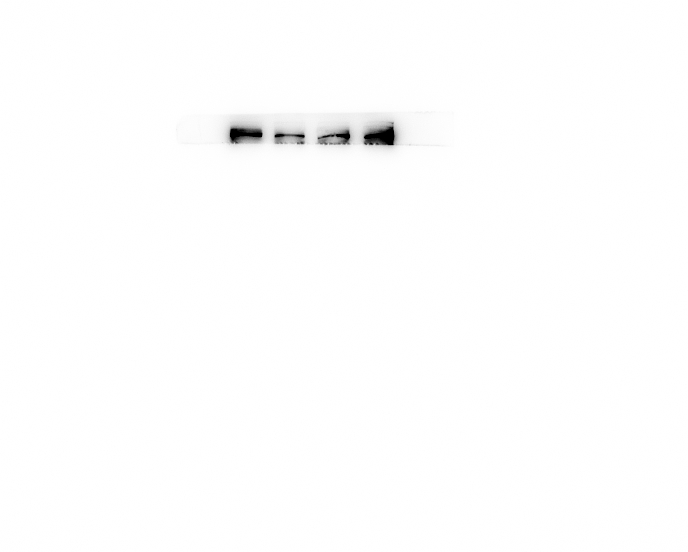

Supplement: Supplementary file 14 [file Data_Sheet_10.ZIP › Fig.8/COL-1.jpg]

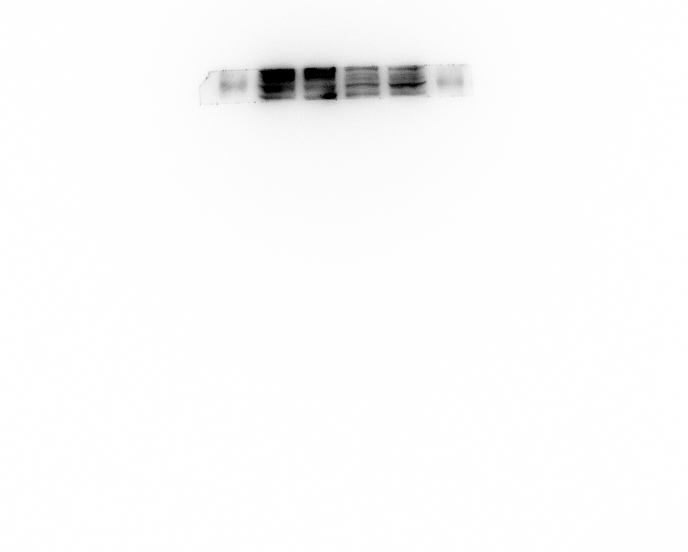

Supplement: Supplementary file 14 [file Data_Sheet_10.ZIP › Fig.8/NR4A1-2.jpg]

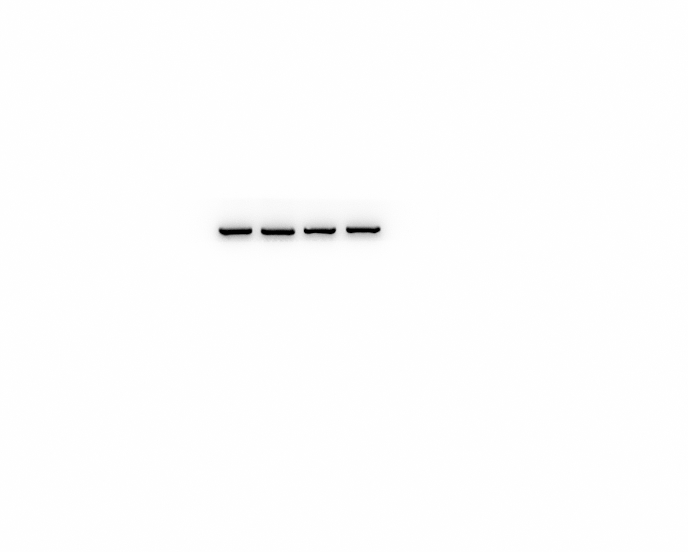

Supplement: Supplementary file 14 [file Data_Sheet_10.ZIP › Fig.8/gapdh.jpg]
